# An evolutionary analysis of lysophospholipid acyltransferases generating membrane phospholipid diversity

**DOI:** 10.3389/fimmu.2026.1838657

**Published:** 2026-08-10

**Authors:** Hideo Shindou, Yosuke Kawai

**Affiliations:** 1 Department of Lipid Life Science, National Institute of Global Health and Medicine, Japan Institute for Health Security (JIHS), Shinjuku-ku, Tokyo, Japan; 2 Department of Medical Lipid Science, Graduate School of Medicine, The University of Tokyo, Bunkyo-ku, Tokyo, Japan; 3 Human Population Genomics laboratory, Department of Informatics, National Institute of Genetics. Yata, Mishima, Shizuoka, Japan; 4 Genome Medical Science Project, National Institute of Global Health and Medicine, Japan Institute for Health Security (JIHS), Shinjuku-ku, Tokyo, Japan

**Keywords:** cellular membrane, LPLAT, membrane diversity, membrane remodeling, pathogen, phospholipid

## Abstract

All living organisms are enclosed by biological membranes composed primarily of phospholipids. The diversification of membrane lipids has shaped key membrane functions, including signaling, membrane protein regulation, and immune responses, and may also have driven organismal evolution. In humans, this diversity is mainly generated by lysophospholipid acyltransferases (LPLATs), yet the evolutionary distribution and diversity of LPLATs across organisms remain poorly understood, particularly in pathogens. Here, we systematically identified candidate LPLAT proteins belonging to two major families in representative prokaryotic and eukaryotic species and reconstructed their phylogenetic relationships. The analysis reveals a marked expansion and diversification of LPLATs in vertebrates and suggests the possibility of pathogen-specific LPLAT expansions. These findings suggest that the diversification of membrane phospholipid remodeling enzymes may accompany increasing biological complexity and pathogenicity. This study provides an evolutionary framework for understanding phospholipid metabolism and highlights pathogen LPLATs as potential targets for exploring host defense mechanisms.

## 1 Introduction

Biological membranes define the boundaries of cells and organelles and play central roles in numerous cellular processes, including signaling, membrane protein regulation, and immune responses (1–3). The principal structural components of these membranes are glycerophospholipids (hereafter referred to as phospholipids). Beyond their structural role in membrane bilayers, phospholipids can also be released as main components of alveolar surfactant and lipoproteins (4, 5). Mammalian phospholipids form a highly diverse molecular population, with more than a thousand molecular species arising from combinations of different polar head groups and fatty acyl chains attached to a glycerol backbone (6).

**Figure 1:**
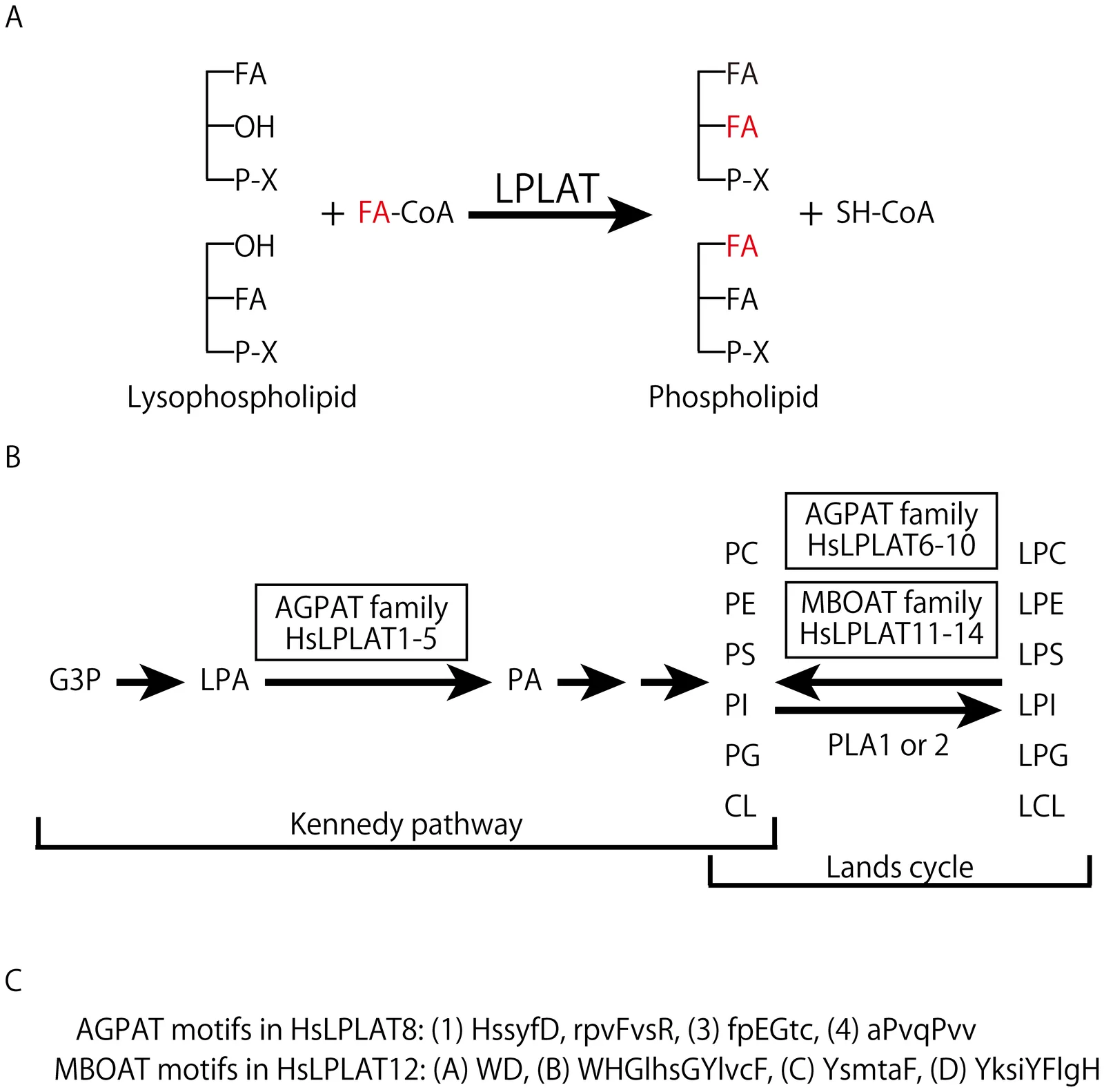
Reaction of LPLAT. LPLAT generates phospholipid from lysophospholipid and acyl-CoA (FA-CoA). **(A)** The upper and lower indicate the *sn*-2 and the *sn*-1 remodeling, respectively. X denotes the polar head groups. **(B)** The biosynthetic pathways of membrane phospholipids in human are shown. G3P, glycrol-3-phosphate. **(C)** AGPAT motifs in HsLPLAT8 and MBOAT motifs in HsLPLAT12 are shown. Lowercase letters indicate residues that are not conserved across a broad range of species. The predicted structures of both motifs are shown in the review (6).

The biosynthesis and remodeling of phospholipids have been extensively studied in mammals. Classical studies established that phospholipids are produced and matured through two major pathways: the Kennedy pathway (7) and the Lands cycle (8). Central enzymes producing phospholipid diversity in both pathways are lysophospholipid acyltransferases (LPLATs), which catalyze the esterification of lysophospholipids using acyl-CoAs (6) (**Figure 1A**). In the Kennedy pathway, phosphatidic acid (PA) is produced by LPLATs from lyso-PA (LPA) and acyl-CoAs. PA is subsequently converted into several other phospholipids, phosphatidylcholine (PC), phosphatidylethanolamine (PE), phosphatidylglycerol (PG), phosphatidylinositol (PI), phosphatidylserine (PS), and cardiolipin (CL). Fatty acids of these phospholipids are reacylated by LPLATs in the Lands cycle (**Figure 1B**). Fourteen LPLATs have been identified in human and mouse, and recent studies combining enzyme characterization with knockout mouse models have begun to reveal their roles in the regulation of membrane phospholipid compositions. These LPLATs belong to two families: 1-acylglycerol-3-phosphate *O*-acyltransferase (AGPAT) and membrane-bound *O*-acyltransferase (MBOAT) families. AGPAT motifs 1–4 and MBOAT motifs A-D, essential for LPLAT activity, have been identified (**Figure 1C**) (9–11). LPLAT structures have been predicted by homology modeling (12) and AlphaFold2 analysis (6), and several 3D structures have also been experimentally determined (13). Conserved motifs in both AGPAT- and MBOAT-family LPLATs may form pockets for substrate binding.

Phospholipid compositions vary widely across organisms, reflecting differences in metabolism, environmental adaptation, and cellular complexity. Although multiple mechanisms can influence phospholipid diversity, LPLAT-mediated remodeling is likely to play a central role in shaping membrane lipid compositions in most organisms. LPLATs have been studied in several model organisms, including *Saccharomyces cerevisiae* (14), *Caenorhabditis elegans* (15), *Drosophila melanogaster* (16), *Escherichia coli* (17, 18), and *Arabidopsis thaliana* (19), as well as in *Homo sapiens* and *Mus musculus* (6, 20). Previous studies showed that deficiency or mutation of LPLATs changed the phospholipid compositions and induced biological dysfunctions, including cell growth reduction in *S. cerevisiae* (14), impaired epithelial cell division in *C. elegans* (15), and abnormal germ cell development in *D. melanogaster* (16). In mammals, several phenotypes have been reported in association with LPLAT deficiency or mutation, including visual dysfunction (21), male infertility (22), delayed neuronal migration in the cortex (23), alleviation of neuropathic pain (24), and suppression of ferroptosis in the liver (25) in LPLAT gene-deficient mice; intellectual disability (26) and lipodystrophy (27) in humans with LPLAT gene mutations; and suppression of cell growth (28) in a human cell line. Several reviews have summarized the effects of human and mouse LPLAT mutations and deficiencies on biological functions (6, 20). The number of LPLATs has increased during evolution. For example, humans possess 14 LPLATs, whereas yeast has only two (6). Consistent with this expansion, the fatty acid composition of membrane phospholipids is more diverse in humans than in yeast, and phospholipid composition differs among species. However, systematic analyses of LPLAT distribution and evolution across diverse taxa remain limited. In addition, pathogens may expand their LPLATs to remodel their membranes in order to survive and thrive in hostile host niches, evade the host immune system, or optimize virulence. Yet, little is known about the diversity of LPLATs in pathogenic organisms. In particular, little is known about the diversity of LPLATs in pathogenic organisms.

In this study, we asked whether the diversification of membrane phospholipid composition across organisms is evolutionarily linked to LPLATs, and whether it is associated with functional changes such as pathogenicity. Here, we conducted a comprehensive survey of LPLAT proteins across representative prokaryotic and eukaryotic lineages, including bacteria and major eukaryotic supergroups such as Opisthokonta, Amoebozoa, Stramenopiles–Alveolata–Rhizaria (SAR), Archaeplastida, and Excavata, based on the classification framework proposed by Adl et al. (29, 30). The phylogenetic analyses reveal the diversification of LPLATs during evolution, with notable expansion in vertebrates. Furthermore, comparative analyses suggest that certain pathogenic species harbor an expanded repertoire of LPLAT homologs compared with their non-pathogenic relatives. These findings indicate that diversification of phospholipid remodeling enzymes may have accompanied organismal evolution and the emergence of pathogenicity. This study provides an evolutionary perspective on membrane phospholipid metabolism and establishes a framework for exploring the functional roles of LPLATs in biological diversity and host–pathogen interactions.

## 2 Methods

### 2.1 Identification of candidate LPLAT proteins

Orthologs of the fourteen known human and mouse LPLAT enzymes were searched in representative prokaryotic and eukaryotic organisms (**Supplementary Table 1**) (29, 30) using the following strategy.

(i) The amino acid sequences of SLC1 (NP_010231, AGPAT family) and ALE1 (NP_014818, MBOAT family) from *S. cerevisiae* were used individually as queries in tBLASTn searches against the NCBI RefSeq genomes database and selected organism genomes.

(ii) Additional searches were performed in the NCBI database using combinations of organism names and the keywords “1-acylglycerol-3-phosphate acyltransferase”, “AGPAT”, “membrane-bound *O*-acyltransferase”, or “MBOAT.” In addition, previously reported LPLATs in several organisms were included.

(iii) Candidate LPLAT amino acid sequences identified in each organism were subsequently used as queries for additional tBLASTn searches against the NCBI non-redundant (nr) database and organism-specific genome databases to identify related genomes.

Candidate proteins were selected if they contained the conserved motifs of AGPAT or MBOAT families, including the AGPAT motifs 1(HxxxxD) and 3(FPEG) (9, 11) or the MBOAT motif B(WHGxxxGYxxxF) (10) (**Figure 1C**). When multiple proteins with identical internal regions were identified, the protein with the longest N- or C-terminal region was selected as the representative sequence. Accession numbers (AccNo) of the proteins and LPLAT names used in this study are listed in **Supplementary Tables 1–3**. The number of clusters was shown in **Supplementary Table 1**.

### 2.2 Sequence alignment and phylogenetic analysis

Multiple sequence alignments were generated using Clustal Omega (31) with default parameters. Phylogenetic trees were inferred from the multiple sequence alignment of LPLAT homolog amino acid sequences using the maximum likelihood method implemented in PhyML (32). The LG model of amino acid substitution and bootstrap analysis with 1,000 replicates were used for the phylogenetic analysis. For phylogenetic tree inference, only the alignments of the AGPAT motif 1–4 or MBOAT motif A–D regions were used. All analyses were performed using SeaView (33), and the final outputs were generated within this software. All alignments were shown in **Supplementary Figures 1–3**.

## 3 Results

### 3.1 Identification of candidate LPLAT proteins across representative organisms

In humans and mice, LPLATs have been classified into two families: LPLAT1–10 belonging to the AGPAT family and LPLAT11–14 belonging to the MBOAT family (6). Because the AGPAT and MBOAT families are evolutionarily distinct and share low sequence homology, phylogenetic trees were constructed separately for each family. To investigate their evolutionary distribution, orthologous LPLAT candidates were identified from representative prokaryotic and eukaryotic clades using the strategy described in the Methods section. All identified candidates contained conserved motifs of either the AGPAT family (Motif 1: HxxxxD and Motif 3: FPEG) (9, 11) or the MBOAT family (Motif B: WHGxxxGYxxxF) (10).

As shown in **Supplementary Tables 1–3**, no MBOAT family LPLATs were detected in several species, including the prokaryote *E. coli* K-12, the choanoflagellate *Monosiga brevicollis*, the alveolate parasite *Plasmodium falciparum*, and the excavate *Trypanosoma cruzi*. In addition, no candidate LPLAT proteins were identified in the archaeal genomes examined.

### 3.2 Classification of LPLATs in representative organisms

**Figure 2:**
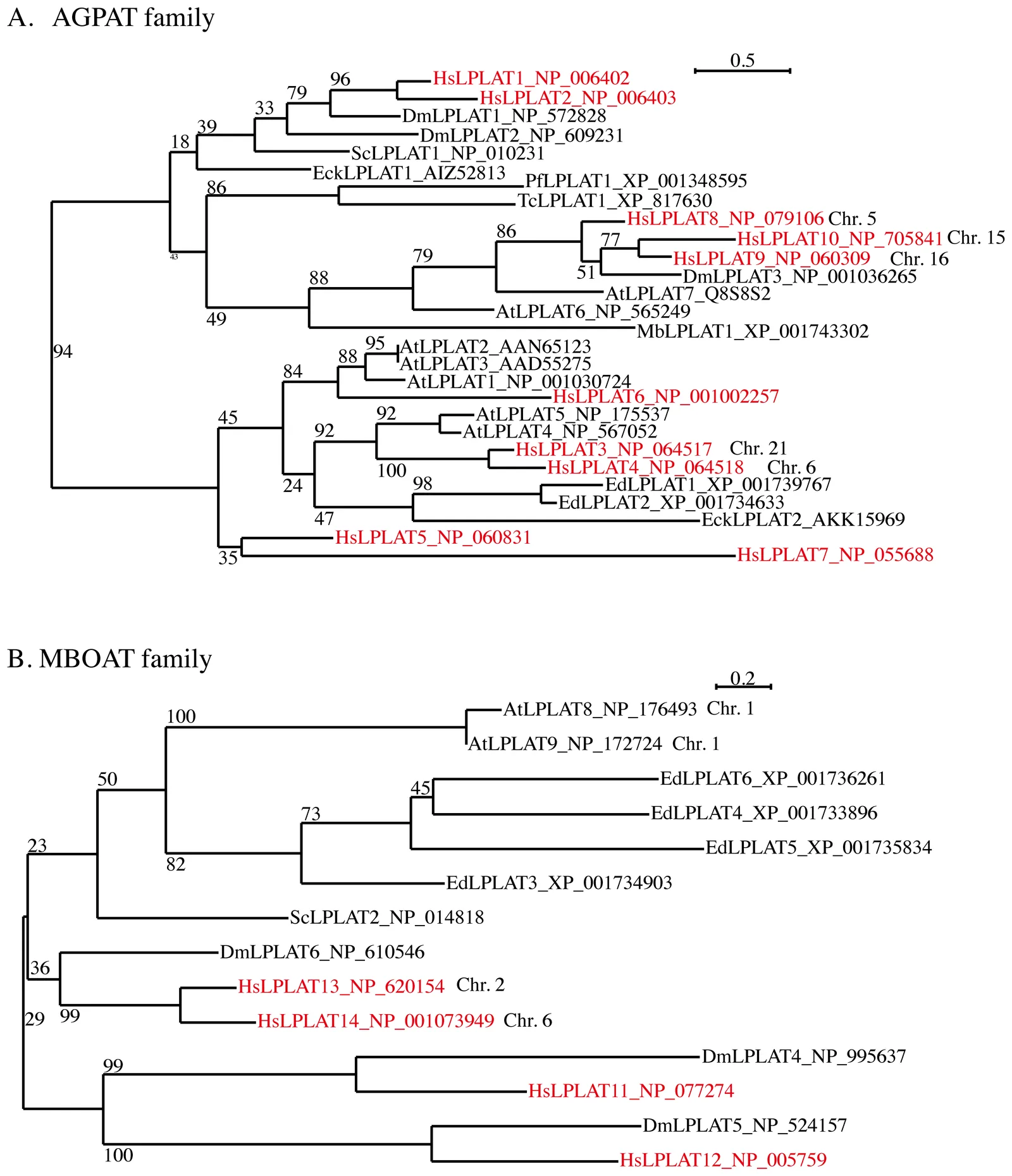
Phylogenetic tree of the representative organisms. Selected organisms and candidate LPLATs are shown in **Supplementary Tables 1–3**. The phylogenetic trees of AGPAT **(A)** and MBOAT **(B)** families were constructed separately. HsLPLATs were indicated in red. The distance scale and scale bar represent evolutionary distance, measured as the estimated number of amino acid substitutions per site.

To examine overall patterns of LPLAT diversity across major evolutionary groups, representative species were selected: *E. coli* K-12 (EckLPLAT), *D. melanogaster* (DmLPLAT), *H. sapiens* (HsLPLAT), *S. cerevisiae* S288C (ScLPLAT), *M. brevicollis* MX1 (MbLPLAT), *Entamoeba dispar* SAW760 (EdLPLAT), *P. falciparum* 3D7 (PfLPLAT), *A. thaliana* (AtLPLAT), and *T. cruzi* (TcLPLAT) (**Supplementary Tables 1–3**). Phylogenetic trees were constructed using SeaView (33) (**Figure 2**).

**Figure 2** shows phylogenetic trees depicting the evolutionary relationships among AGPAT (**Figure 2A**) and MBOAT (**Figure 2B**) LPLAT families across various species. In **Figure 2A**, the phylogenetic analysis identified 7 and 16 major clades with bootstrap values ≧90 and ≧50, respectively, whereas in **Figure 2B**, 4 and 7 major clades were identified with bootstrap values ≧90 and ≧50, respectively. Although several deep ancestral nodes exhibited low bootstrap support (<50), likely due to low sequence homology across highly divergent evolutionary clades, the distinct separation of major clusters (such as HsLPLAT1/2 vs. HsLPLAT8/9/10) remains highly supported. Further structural or synteny analyses may be required to firmly resolve these deeper evolutionary relationships. In the AGPAT family, LPLATs were grouped into three major clusters, comprising HsLPLAT1/2, HsLPLAT3/4/5/6/7, and HsLPLAT8/9/10. In contrast, the MBOAT family was divided into two major clusters, HsLPLAT11/12 and HsLPLAT13/14. Although HsLPLAT3 and HsLPLAT4 belong to the same cluster, they are located on chromosomes 21 (https://www.ncbi.nlm.nih.gov/protein/NP_064517) and 6 (https://www.ncbi.nlm.nih.gov/protein/NP_064518), respectively. Similarly, HsLPLAT8, HsLPLAT9, and HsLPLAT10, which form another cluster, are located on chromosomes 5 (https://www.ncbi.nlm.nih.gov/protein/NP_079106), 16 (https://www.ncbi.nlm.nih.gov/protein/NP_060309), and 15 (https://www.ncbi.nlm.nih.gov/protein/NP_705841), respectively. HsLPLAT13 and HsLPLAT14 also belong to the same cluster and exhibit similar substrate preferences, yet they are located on chromosomes 2 (https://www.ncbi.nlm.nih.gov/protein/NP_620154) and 6 (https://www.ncbi.nlm.nih.gov/protein/NP_001073949), respectively. The observations suggest that these gene pairs or groups are unlikely to have arisen through tandem duplication. HsLPLAT7 diverged earlier than the other LPLATs, suggesting that it may represent a distinct evolutionary lineage (**Figure 2A**). AtLPLAT8 and AtLPLAT9 show a close relationship with a high bootstrap value. Although both genes are located on chromosome 1 (https://www.ncbi.nlm.nih.gov/protein/NP_176493; https://www.ncbi.nlm.nih.gov/protein/NP_172724), they are not adjacent to each other, suggesting that they may not be products of tandem duplication (**Figure 2B**).

The reported biochemical characteristics of human and mouse LPLATs (mammalian LPLATs) were shown in **Supplementary Table 4**. HsLPLATs are not a single monophyletic group but instead split into distinct evolutionary lineages, suggesting that different HsLPLAT proteins may have evolved to handle different lipid substrates and serve distinct physiological roles within the cells. Although functional verification is required to determine whether non-human LPLATs possess identical enzymatic activities, several observations suggest potential similarities. For example, in *S. cerevisiae*, ScLPLAT1 (SLC1) incorporates C18:1 into LPA and lysophosphatidylcholine (LPC) (34, 35), whereas ScLPLAT2 (LPT1, ALE1, SLC4) can utilize a broad range of lysophospholipids and can incorporate highly unsaturated fatty acids *in vitro* (34, 36–38). In *D. melanogaster*, DmLPLAT4 (farjavit), DmLPLAT5 (nessy), and DmLPLAT6 (oysgedart) exhibit substrate preferences for LPI/C20:4-CoA, LPC/C20:4-CoA, LPC/C18:1-CoA as substrates, respectively (16). HsLPLAT11, HsLPLAT12, and HsLPLAT13/14 belong to the same clusters as DmLPLAT4, DmLPLAT5, and DmLPLAT6, respectively, and show substrate preferences (6) similar to those of the corresponding DmLPLATs (16). They may be orthologs with conserved functions across vertebrates and invertebrates. Each LPLAT in *S. cerevisiae* and *D. melanogaster* exhibits characteristics similar to those of the corresponding mammalian LPLATs (**Figure 2**). Although PfLPLAT1 was not classified into any human LPLAT group, LPA acyltransferase (LPAAT) and LPC acyltransferase (LPCAT) activities with several acyl-CoAs have been reported (39). Notably, mammalian LPLAT5 and mammalian LPLAT7, which incorporate C18:0 or C18:1 into phospholipids (6), were observed only in metazoans among the species examined. These enzymes may represent a vertebrate-specific expansion. Mammalian LPLAT6 and mammalian LPLAT7 esterify fatty acids at the *sn*-1 position of phospholipids (**Figure 1A**) (40–43). A close relationship between HsLPLAT6 and plant AtLPLAT1, AtLPLAT2, and AtLPLAT3 was observed. Further studies are needed to characterize the biochemical properties of HsLPLAT6 and AtLPLAT1–3.

### 3.3 Diversification of LPLATs in Metazoa

**Figure 3:**
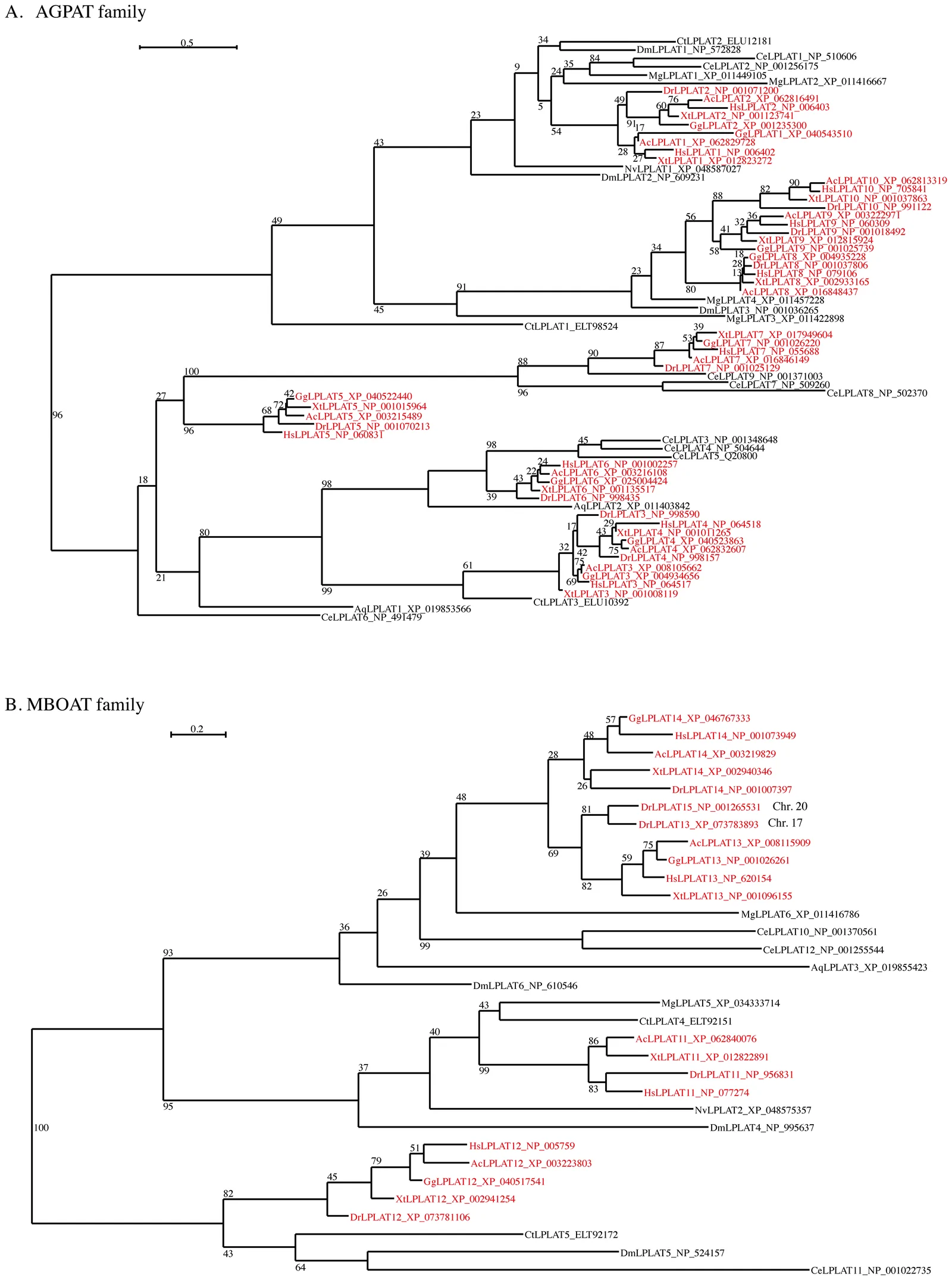
Phylogenetic tree of the representative Metazoa. Selected organisms and candidate LPLATs are shown in **Supplementary Tables 1–3**. The phylogenetic trees of AGPAT **(A)** and MBOAT **(B)** families were constructed separately. Vertebrate LPLATs were indicated in red. The distance scale and scale bar represent evolutionary distance, measured as the estimated number of amino acid substitutions per site.

To further investigate LPLAT evolution within metazoa, phylogenetic trees were constructed using representative invertebrates—*Amphimedon queenslandica* (AqLPLAT), *Nematostella vectensis* (NvLPLAT), *Magallana gigas* (MgLPLAT), *D. melanogaster* (DmLPLAT), *C. elegans* (CeLPLAT), and *Capitella teleta* (CtLPLAT)—and vertebrates—*Danio rerio* (DrLPLAT), *Xenopus tropicalis* (XtLPLAT), *Anolis carolinensis* (AcLPLAT), *Gallus gallus* (GgLPLAT), and *H. sapiens* (**Supplementary Tables 1–3**). **Figure 3** shows phylogenetic trees depicting the evolutionary relationships among AGPAT (**Figure 3A**) and MBOAT (**Figure 3B**) LPLAT families across various species. In **Figure 3A**, the phylogenetic analysis identified 11 and 30 major clades with bootstrap values ≧90 and ≧50, respectively, whereas in **Figure 3B**, 5 and 17 major clades were identified with bootstrap values ≧90 and ≧50, respectively.

Members of the LPLAT2–9 group in the AGPAT family and the LPLAT12–14 group in the MBOAT family were identified in *D. rerio*, *X. tropicalis*, *A. carolinensis*, *G. gallus*, and *H. sapiens*. However, the analysis revealed that *D. rerio* lacked an ortholog of HsLPLAT1 but contained two orthologs of HsLPLAT13 (DrLPLAT13 and DrLPLAT15), suggesting a lineage-specific duplication, because DrLPLAT13 and DrLPLAT15 are located on chromosomes 17 (https://www.ncbi.nlm.nih.gov/protein/XP_073783893.1/) and 20 (https://www.ncbi.nlm.nih.gov/protein/NP_001265531.1/), respectively. In contrast, *G. gallus* lacked the orthologs of HsLPLAT10 and HsLPLAT11. The full complement of 10 AGPAT-family LPLATs and 4 MBOAT-family LPLATs appears to have been established before the emergence of vertebrates. These findings suggest that the diversification of phospholipid remodeling enzymes had largely occurred prior to vertebrate evolution.

LPLAT1 and LPLAT2 cluster sequences may share a common ancestor and exhibit close evolutionary relationships across vertebrates and some invertebrates. LPLAT5 and LPLAT7 showed high bootstrap values of 96 and 100, respectively, and formed distinct major lineages in the phylogenetic tree. LPLAT8, 9, and 10 formed a distinct group, which may reflect relatively recent duplication events in vertebrates. Based on the substrate specificities of mouse LPLATs, these enzymes are predicted to be involved in sn-1/sn-2 remodeling; however, no clear commonality in fatty acid recognition has been identified (6). Further analyses of the substrate specificities of LPLATs from additional species will be necessary.

Vertebrate LPLATs formed tight clusters, suggesting strong conservation of these specific LPLAT isoforms across vertebrates. In particular, vertebrate LPLAT11 and LPLAT12 showed high bootstrap support values of 99 and 82, respectively, suggesting that they are orthologs. LPLAT14 and LPLAT13 groups showed strong conservation among vertebrates. LPLAT14 sequences from different vertebrate species formed a tight cluster, whereas LPLAT13 sequences formed a sister group to LPLAT14, suggesting a relatively recent duplication event in vertebrates. Although DrLPLAT13 and DrLPLAT15 are located on different chromosomes, they may have arisen through a gene duplication event rather than tandem duplication. In the LPLAT12 group, the relatively long branch lengths of the invertebrate LPLATs may reflect a greater accumulation of amino acid substitutions. The distinct clustering of vertebrate and invertebrate sequences may indicate that these lineages diverged relatively early during evolution.

Overall, vertebrates displayed a greater diversity of LPLATs than invertebrates. The diversification of LPLATs may have accompanied evolutionary processes. Nevertheless, some invertebrates showed relatively high numbers of LPLAT proteins. For example, *C. elegans*, *M. gigas*, and *D. melanogaster* possessed comparatively large LPLAT repertoires. In particular, *C. elegans* contained 12 LPLAT proteins. Previous studies reported that triple knockout of CeLPLAT3 (acl-8), CeLPLAT4 (acl-9), and CeLPLAT5 (acl-10) in *C. elegans* reduces PI species containing C18:0 (15), suggesting that these LPLATs contribute to the regulation of PI and phosphoinositide compositions.

### 3.4 Differences in LPLAT repertoires between pathogenic and non-pathogenic species

**Figure 4:**
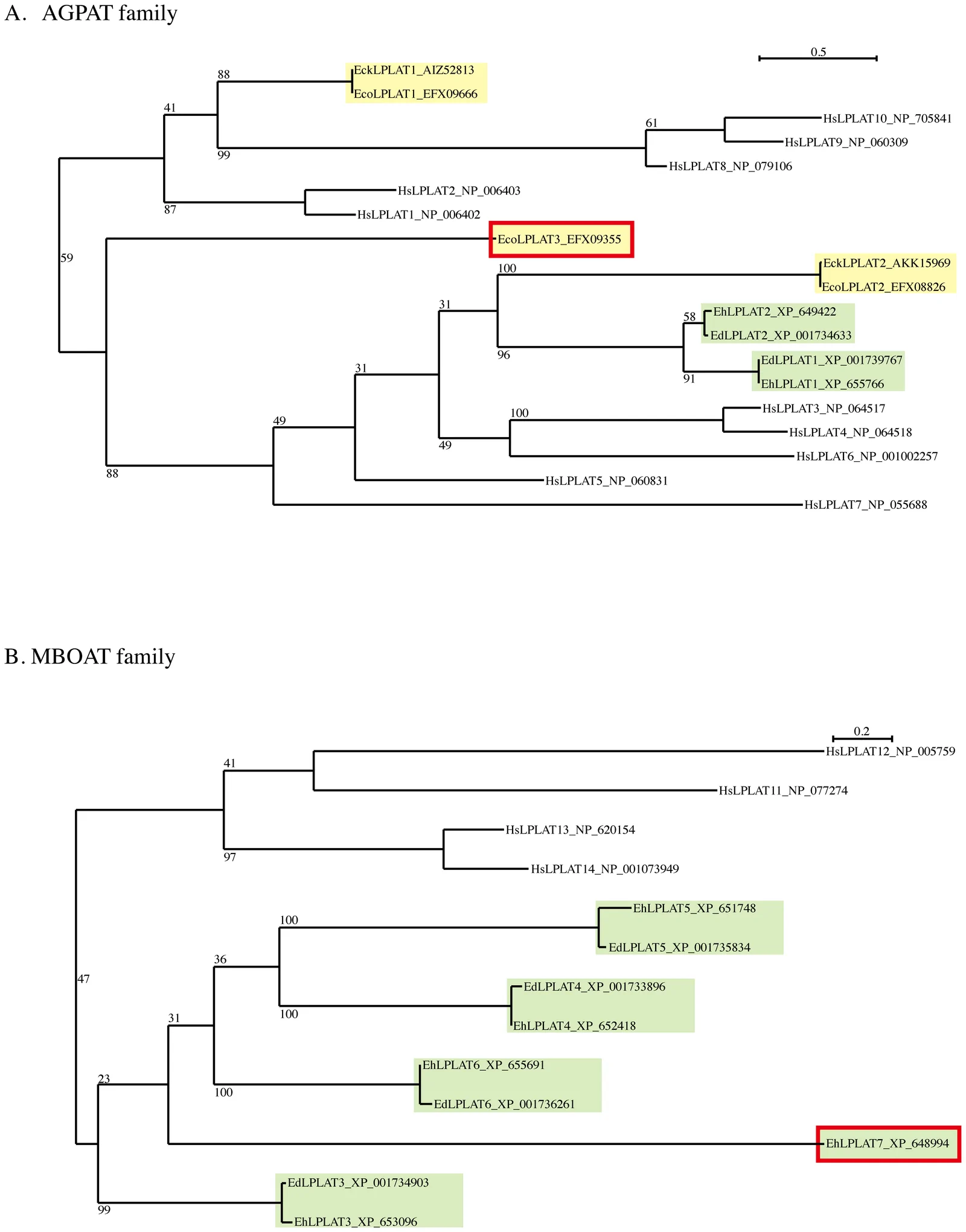
Phylogenetic tree of pathogenic and non-pathogenic strains. The phylogenetic trees of AGPAT **(A)** and MBOAT **(B)** families were constructed. Selected organisms and candidate LPLATs are shown in **Supplementary Tables 1–3**. *E. coli* and Entamoeba are shown in light yellow and light green, respectively. Red squares indicate additional LPLATs of pathogenic species. The distance scale and scale bar represent evolutionary distance, measured as the estimated number of amino acid substitutions per site.

To examine whether LPLAT compositions are associated with pathogenicity, LPLAT repertoires were compared between pathogenic and non-pathogenic species. Specifically, this study analyzed *E. coli* K-12 and the pathogenic strain *E. coli* O157:H7 (strain G5101) (EcoLPLAT), as well as the non-pathogenic amoeba *E. dispar* SAW760 and the pathogenic species *Entamoeba histolytica* HM-1:IMSS (EhLPLAT) (**Supplementary Tables 1–3**). Phylogenetic analyses were performed together with HsLPLATs (**Figure 4**). **Figure 4** shows phylogenetic trees depicting the evolutionary relationships among AGPAT (**Figure 4A**) and MBOAT (**Figure 4B**) LPLAT families across various species. In **Figure 4A**, the phylogenetic analysis identified 5 and 11 major clades with bootstrap values ≧90 and ≧50, respectively, whereas in **Figure 4B**, 5 and 5 major clades were identified with bootstrap values ≧90 and ≧50, respectively.

*E. coli* K-12 possessed two AGPAT-family LPLATs, whereas the pathogenic species *E. coli* O157:H7 contained three, including an additional protein (EcoLPLAT3) (**Figure 4A**). In *Entamoeba*, both *Entamoeba* species possessed two AGPAT-family paralogs. Unlike *E. coli*, however, *Entamoeba* species also possess MBOAT-family LPLATs, resulting in a total of four LPLAT paralogs. The very short branch lengths between EcoLPLAT and EckLPLAT suggest high conservation across these strains. EcoLPLAT1/EckLPLAT1, EcoLPLAT2/EckLPLAT2, EdLPLAT1/EhLPLAT1, EdLPLAT3/EhLPLAT3, EdLPLAT4/EhLPLAT4, EdLPLAT5/EhLPLAT5, and EdLPLAT6/EhLPLAT6 showed high bootstrap values of 88, 100, 91, 99, 100, 100, and 100, respectively. The LPLATs in *E. coli* and amoebae may suggest lineage-specific gene duplication events that occurred before divergence within each lineage. HsLPLAT3–7 formed a separate evolutionary branch from the bacterial and protozoan groups. Notably, the pathogenic species *E. histolytica* possesses an additional protein, EhLPLAT7. Within the MBOAT family tree, EhLPLAT7 forms an independent branch that clusters more closely with the Entamoeba LPLAT3/4/5/6 group than with HsLPLATs (**Figure 4B**). These observations suggest that certain pathogenic species may possess expanded LPLAT repertoires relative to their non-pathogenic relatives.

## 4 Discussion

Membrane phospholipid biosynthesis has diversified significantly during evolution. To map these relationships, we constructed phylogenetic trees based on the conserved motifs of the AGPAT and MBOAT families. Our findings suggest that the structural diversification of these LPLATs may have driven the expansion of ecological niches and the development of specialized cellular functions. This evolutionary diversity is reflected in modern mammals; for instance, the 14 human and mouse LPLAT isoforms exhibit highly distinct tissue expression patterns and physiological roles. Specifically, LPLAT3 is highly expressed in the retina and testis, and its deficiency leads to visual dysfunction (21) and male infertility (22), respectively. Ultimately, variations in LPLAT expression, substrate preferences, and enzymatic regulation act as primary determinants of cellular lipid profiles, offering new insights into both baseline physiology and host defense mechanisms.

In this study, MBOAT-family LPLATs were not detected in prokaryotes. Although AGPAT-family LPLATs were identified in the prokaryotic bacterium *E. coli*, no LPLAT candidates were detected in archaeal genomes. Archaeal membrane lipids are structurally distinct from those of bacteria and eukaryotes, as they typically contain ether-linked isoprenoid chains at both the *sn*-1 and *sn*-2 positions of the glycerol backbone (44). Therefore, the acyl-CoA–dependent esterification catalyzed by LPLATs may not be required in archaeal lipid biosynthesis. Similar to *E. coli*, MBOAT-family LPLATs were not found in *M. brevicollis*, *P. falciparum*, or *T. cruzi*. MBOAT-family LPLATs are characterized by their ability to incorporate arachidonic acid into PC and PI. These lipids are involved in lipid mediator production and phosphoinositides (PIPs) signaling. Such features may have evolved to enable greater diversity in membrane lipid compositions. During evolution, MBOAT-family LPLATs and metazoan-specific AGPAT-family LPLATs such as mammalian LPLAT5, mammalian LPLAT6, and mammalian LPLAT7 appear to have arisen, potentially reflecting the increasing complexity of membrane lipid remodeling. These enzymes may be particularly important for processes such as polyunsaturated fatty acids (PUFA)s incorporation and remodeling of *sn*-1 fatty acids.

PA serves as the central precursor for the synthesis of many other phospholipids in the *de novo* pathway (Kennedy pathway). PA synthesis using a broad range of fatty acids may therefore represent a conserved and essential feature of membrane phospholipid metabolism across diverse organisms. In addition, the phylogenetic analysis identified several LPLAT candidates that could not be clearly classified into mammalian LPLAT groups (**Figure 2**). As with most LPLATs, enzymatic *in vitro* analyses combined with lipidomic studies of LPLAT knockout organisms will be essential to clarify the phospholipid species regulated by these LPLATs.

This study suggests possible functional links between LPLAT diversification and specific phospholipid metabolism. HsLPLAT11, which incorporates arachidonic acid into LPI, contributes to PI metabolism and phosphoinositide signaling pathways. A similar enzyme, DmLPLAT4 (farjavit), was identified in the arthropod *D. melanogaster*. DmLPLAT4 (farjavit) has been reported to synthesize arachidonic-acid–containing PI (16), suggesting functional similarity to HsLPLAT11. In addition, the mouse ortholog of HsLPLAT6 catalyzes the esterification of stearic acid at the *sn*-1 position of PI (41). In this study, orthologs of HsLPLAT6 were restricted to A. thaliana (AtLPLATs) and not widely detected among other examined species. This activity may therefore represent a relatively recent evolutionary innovation that contributes to phosphoinositide regulation.

Other LPLAT groups may also have contributed to the evolution of lipid mediator pathways. HsLPLAT12 and DmLPLAT5 (nessy) incorporate arachidonic acid into LPC (16, 45, 46). Arachidonic acid is not only a structural component of membrane phospholipids but also the precursor of lipid mediators, eicosanoids (47), indicating that these enzymes may have contributed to the evolution of lipid mediator production. However, because the physiological roles of lipid mediators differ widely among organisms, and their biosynthetic mechanisms cannot be directly compared across species, further studies are needed to evaluate the relationship between LPLAT evolution and lipid mediators.

Among phospholipids, platelet-activating factor (PAF) is a potent phospholipid mediator (48, 49). PAF is rapidly synthesized in response to extracellular stimuli (50, 51), and the PAF biosynthetic enzyme lyso-PAF acetyltransferase (52) is regulated through three distinct mechanisms (53). Mammalian LPLAT9 catalyzes the lyso-PAF acetyltransferase reaction responsible for PAF biosynthesis (54, 55). Notably, mammalian LPLAT9 is the only member among the fourteen LPLATs whose phosphorylation and expression are induced by innate immune stimuli, such as toll-like receptor (TLR)4 or TLR9 ligands (24, 56, 57). These observations suggest that diversification of the LPLAT family may have contributed not only to membrane phospholipid remodeling but also to the evolution of lipid-mediated signaling systems involved in host defense. This group in the phylogenetic tree also includes mammalian LPLAT8 and mammalian LPLAT10, which also have distinctive characters. Mammalian LPLAT8 contributes to the synthesis of pulmonary surfactant phospholipids essential for respiration and increases the saturated fatty acids in PC (58–62). Mammalian LPLAT10 incorporates very long chain fatty acid into the *sn*-1 position of PC (63, 64). Although similar physiological functions cannot be assumed in non-mammal organisms, these enzymes highlight the potential importance of LPLAT-mediated phospholipid remodeling in evolutionary adaptations.

Docosahexaenoic acid (DHA, C22:6) is a very long-chain fatty acid containing six double bonds and plays multiple functional roles in mammals (21, 22, 26, 65). Several studies have reported LPLAT activities that generate DHA-containing phospholipids, mediated by mammalian LPLAT3 (66), mammalian LPLAT4 (67), and mammalian LPLAT10 (63, 64, 68–70). In addition to serving as membrane components, DHA-containing phospholipids function as a reservoir for lipid mediators (71), similar to arachidonic acid (C20:4)-containing phospholipids (47). Considering the number of double bonds in fatty acids may provide important insights into the evolution of phospholipid biosynthesis.

Other enzymes such as mammalian LPLAT13 and mammalian LPLAT14, which preferentially utilize oleoyl-CoA (72, 73), and DmLPLAT6 (oysgedart) (16) and ScLPLAT2 (LPT1, ALE1, SLC4) (34, 36–38) in this cluster also possess similar preferences. Although ScLPLAT2 has been reported to recognize PUFA substrates *in vitro*, yeast membranes contain relatively low levels of PUFAs (74). Similar discrepancies have been reported in mouse LPLAT studies (6), in which *in vitro* enzymatic activity does not always correlate with the phospholipid species altered *in vivo* following LPLAT deficiency or overexpression.

Membrane phospholipids play important roles in immune function. Membrane nanodomains (also called microdomains), which are enriched in specific phospholipids, are involved in various cellular functions, including immunoreceptor signaling. These nanodomains serve as platforms for the accumulation of signaling molecules involved in immune receptor pathways, such as Src-family kinases, G proteins, and PIPs, thereby influencing T cell receptor (TCR) and B cell receptor (BCR) signaling (75). PI is a precursor of PIPs. In addition, fatty acids contained in phospholipids, such as arachidonic acid, DHA, and linoleic acid, serve as precursors for lipid mediators. These fatty acid-derived lipid mediators are produced in response to extracellular stimuli, originating from membrane phospholipids in immune cells. Thus, membrane phospholipids and LPLATs that regulate their remodeling play critical roles in the modulation of immune signaling. Furthermore, understanding pathogen-derived phospholipids and LPLATs will provide important insights into pathogen control (39). Elucidating LPLATs in both pathogens and hosts will also contribute to advances in immunological research.

Interestingly, differences in LPLAT repertoires were also observed between pathogenic and non-pathogenic species within the same genus. The pathogenic bacterium *E. coli* O157 contains one additional AGPAT-family LPLAT, EcoLPLAT3, compared with the non-pathogenic strain *E. coli* K-12. Similarly, the pathogenic amoeba *E. histolytica* contains one additional MBOAT-family LPLAT, EhLPLAT7, compared with the non-pathogenic species *E. dispar*. These additional enzymes may contribute to the synthesis of phospholipids involved in pathogenicity or may compensate for other LPLATs. The phospholipid biosynthetic pathway in *E. histolytica* has also been proposed, highlighting the importance of future studies (76). Functional studies, including gene disruption analysis, will be required to further clarify their roles in virulence. Comparing only two pairs of organisms (*E. coli* and *Entamoeba*), which are isolated data points, is insufficient to suggest a broader evolutionary or biological trend linking lipid remodeling to virulence. Expanding such analyses to additional pathogens may provide important insights into host–pathogen interactions.

To inhabit diverse environments rather than a specific one, organisms likely require a broad range of membrane properties to ensure adaptability. Furthermore, it can be inferred that diverse membrane phospholipid compositions were advantageous for the formation of complex tissues from multiple cell types and for the acquisition of specialized functions. Evaluating the effects of phospholipids on physicochemical properties is also an important issue. For example, LPLAT3-deficient mice, which show reduced levels of DHA-containing phospholipids, exhibit disruption of photoreceptor disc structures and abnormalities in sperm morphology. However, the molecular mechanisms by which phospholipid properties regulate these processes remain unclear. The observation that LPLATs are most abundant in animals, particularly in mammals, suggests a link between organismal evolution and the diversification of phospholipids and LPLATs. It can be inferred that diverse phospholipid compositions are advantageous for generating the wide range of cellular and organelle morphologies. In addition, the presence of highly unsaturated fatty acids may contribute not only to the diversification of lipid mediators but also to a potential role as a sink for lipid oxidation. Elucidating these mechanisms, including their possible involvement in the acquisition of pathogenicity, remains an important direction for future research.

## 5 Limitations

This study is based primarily on publicly available genome annotations, and therefore, some species may lack the information. BLAST-based searches can be influenced by the selected query sequence and the search parameters.

Phospholipid compositions in cells are regulated not only by LPLATs but also by multiple additional factors, such as acyl-CoA synthetases (77), phospholipase As (78, 79), transacylases (78), substrate availability including lipid nutrients, and intracellular localization of LPLATs. These regulatory mechanisms may vary among organisms. For example, the bacterial enzyme Aas in *E. coli* can synthesize PA using acyl-acyl carrier protein (acyl-ACP) rather than acyl-CoA (80). Although Aas was not included in the current analysis because of its distinct enzymatic mechanism, such enzymes indicate that additional pathways may contribute to membrane phospholipid remodeling. Therefore, LPLATs alone cannot fully explain the diversity of membrane phospholipid compositions.

In this study, phylogenetic analyses were performed using newly identified candidate LPLAT proteins. Further biochemical and biological characterization of LPLATs from diverse organisms beyond humans and mice, both *in vitro* and *in vivo*, will contribute to a deeper understanding of the evolution of LPLATs and membrane phospholipids. Additionally, the phospholipid compositions of individual organisms should be analyzed to better understand the evolution of phospholipid biosynthesis. While sequence conservation represents potential enzymatic capabilities, the known variations between *in vitro* activity and *in vivo* phospholipid profiles highlight that sequence alone does not dictate cellular phenotype. Thus, these phylogenetic clusters should be viewed as predictive bases needing *in vivo* lipidomic studies.

Our results contained the weak nodes. While the deeper backbone nodes of the AGPAT tree lack strong bootstrap support, the functional divergence of these lineages is independently supported by distinct chromosomal distributions (e.g., chromosomes 5, 16, and 15 for HsLPLAT8/9/10) and diverging substrate preferences observed in experimental models.

## 6 Conclusion

Comparative analyses of LPLATs across diverse organisms suggest that membrane phospholipid remodeling diversified during organismal evolution in parallel with LPLAT diversification. Expansion of the LPLAT family, along with regulation of their tissue distribution and enzymatic activities, may have contributed to the generation of diverse membrane environments in living organisms. These findings support the idea that LPLAT diversification is closely associated with organismal evolution. The identification of pathogen-specific LPLAT expansions further indicates that these enzymes may contribute to the emergence of pathogenic traits, although further biochemical characterization of these LPLATs will be necessary. Even if certain LPLATs are not directly involved in pathogenicity, inhibitors targeting pathogen-specific LPLATs may represent potential targets for antimicrobial drug development. Thus, the evolutionary study of phospholipid metabolism provides insights not only into membrane biology and evolutionary processes but also into host defense and the control of infectious diseases.

## Data Availability

Publicly available datasets were analyzed in this study. This data can be found here: Amino acid sequences from NCBI database. Only accession numbers are listed in the **Supplementary Tables 2 and 3**.

## Supplementary material

Supplementary Figure 1 **(A)** Sequence alignment utilized in **Figure 2A**. AGPAT motifs in HsLPLAT8 were shown. **(B)** Sequence alignment utilized in **Figure 2B**. MBOAT motifs in HsLPLAT12 were shown.

Supplementary Figure 2 **(A)** Sequence alignment utilized in **Figure 4A**. AGPAT motifs in HsLPLAT8 were shown. **(B)** Sequence alignment utilized in **Figure 4B**. MBOAT motifs in HsLPLAT12 were shown.

Supplementary Figure 3 **(A)** Sequence alignment utilized in **Figure 3A**. AGPAT motifs in HsLPLAT8 were shown. **(B)** Sequence alignment utilized in **Figure 3B**. MBOAT motifs in HsLPLAT12 were shown.

## References

[1] van Meer G Voelker DR Feigenson GW . Membrane lipids: where they are and how they behave. Nat Rev Mol Cell Biol. (2008) 9:112–24. doi: 10.1038/nrm2330 18216768 PMC2642958

[2] Antonny B Vanni S Shindou H Ferreira T . From zero to six double bonds: phospholipid unsaturation and organelle function. Trends Cell Biol. (2015) 25:427–36. doi: 10.1016/j.tcb.2015.03.004 25906908

[3] Hishikawa D Valentine WJ Iizuka-Hishikawa Y Shindou H Shimizu T . Metabolism and functions of docosahexaenoic acid-containing membrane glycerophospholipids. FEBS Lett. (2017) 591:2730–44. doi: 10.1002/1873-3468.12825 28833063 PMC5639365

[4] Numata M Voelker DR . Anti-inflammatory and anti-viral actions of anionic pulmonary surfactant phospholipids. Biochim Biophys Acta Mol Cell Biol Lipids. (2022) 1867:159139. doi: 10.1016/j.bbalip.2022.159139 35240310 PMC9050941

[5] Chen A Zhang C Ding WX Ni HM . Mechanisms of scramblases in regulating hepatic lipoprotein secretion and autophagy. J Lipid Res. (2026) 67:100979. doi: 10.1016/j.jlr.2026.100979 41539605 PMC12887103

[6] Valentine WJ Yanagida K Kawana H Kono N Noda NN Aoki J . Update and nomenclature proposal for mammalian lysophospholipid acyltransferases, which create membrane phospholipid diversity. J Biol Chem. (2022) 298:101470. doi: 10.1016/j.jbc.2021.101470 34890643 PMC8753187

[7] Kennedy EP Weiss SB . The function of cytidine coenzymes in the biosynthesis of phospholipides. J Biol Chem. (1956) 222:193–214. doi: 10.1016/s0021-9258(19)50785-2 13366993

[8] Lands WE . Metabolism of glycerolipides; a comparison of lecithin and triglyceride synthesis. J Biol Chem. (1958) 231:883–8. doi: 10.1016/s0021-9258(18)70453-5 13539023

[9] Harayama T Shindou H Ogasawara R Suwabe A Shimizu T . Identification of a novel noninflammatory biosynthetic pathway of platelet-activating factor. J Biol Chem. (2008) 283:11097–106. doi: 10.1074/jbc.m708909200 18285344

[10] Shindou H Eto M Morimoto R Shimizu T . Identification of membrane O-acyltransferase family motifs. Biochem Biophys Res Commun. (2009) 383:320–5. doi: 10.1016/j.bbrc.2009.04.013 19361486

[11] Yamashita A Hayashi Y Matsumoto N Nemoto-Sasaki Y Oka S Tanikawa T . Glycerophosphate/acylglycerophosphate acyltransferases. Biology. (2014) 3:801–30. doi: 10.3390/biology3040801 25415055 PMC4280512

[12] Hamano F Matoba K Hashidate-Yoshida T Suzuki T Miura K Hishikawa D . Mutagenesis and homology modeling reveal a predicted pocket of lysophosphatidylcholine acyltransferase 2 to catch acyl-CoA. FASEB Journal: Off Publ Fed Am Societies For Exp Biol. (2021) 35:e21501. doi: 10.1096/fj.202002591R 33956375 PMC12315963

[13] Zhang Q Yao D Rao B Jian L Chen Y Hu K . The structural basis for the phospholipid remodeling by lysophosphatidylcholine acyltransferase 3. Nat Commun. (2021) 12:6869. doi: 10.1038/s41467-021-27244-1 34824256 PMC8617236

[14] Oelkers P Pokhrel K . Four acyltransferases uniquely contribute to phospholipid heterogeneity in Saccharomyces cerevisiae. Lipid Insights. (2016) 9:31–41. doi: 10.4137/LPI.S40597 27920551 PMC5127605

[15] Imae R Inoue T Kimura M Kanamori T Tomioka NH Kage-Nakadai E . Intracellular phospholipase A1 and acyltransferase, which are involved in Caenorhabditis elegans stem cell divisions, determine the sn-1 fatty acyl chain of phosphatidylinositol. Mol Biol Cell. (2010) 21:3114–24. doi: 10.1091/mbc.E10-03-0195 20668164 PMC2938378

[16] Steinhauer J Gijon MA Riekhof WR Voelker DR Murphy RC Treisman JE . Drosophila lysophospholipid acyltransferases are specifically required for germ cell development. Mol Biol Cell. (2009) 20:5224–35. doi: 10.1091/mbc.e09-05-0382 19864461 PMC2793297

[17] Coleman J . Characterization of Escherichia coli cells deficient in 1-acyl-sn-glycerol-3-phosphate acyltransferase activity. J Biol Chem. (1990) 265:17215–21. doi: 10.1016/s0021-9258(17)44891-5 2211622

[18] Suwanawat N Ogawa T Toyotake Y Kawamoto J Kurihara T . Biochemical characterization and mutational analysis of lysophosphatidic acid acyltransferases of Escherichia coli highlighting their involvement in the generation of membrane phospholipid diversity. J Biochem. (2025) 177:259–72. doi: 10.1093/jb/mvae093 39727331

[19] Stalberg K Stahl U Stymne S Ohlrogge J . Characterization of two Arabidopsis thaliana acyltransferases with preference for lysophosphatidylethanolamine. BMC Plant Biol. (2009) 9:60. doi: 10.1186/1471-2229-9-60 19445718 PMC2690597

[20] Valentine WJ Shimizu T Shindou H . Lysophospholipid acyltransferases orchestrate the compositional diversity of phospholipids. Biochimie. (2023) 215:24–33. doi: 10.1016/j.biochi.2023.08.012 37611890

[21] Shindou H Koso H Sasaki J Nakanishi H Sagara H Nakagawa KM . Docosahexaenoic acid preserves visual function by maintaining correct disc morphology in retinal photoreceptor cells. J Biol Chem. (2017) 292:12054–64. doi: 10.1074/jbc.M117.790568 28578316 PMC5519357

[22] Iizuka-Hishikawa Y Hishikawa D Sasaki J Takubo K Goto M Nagata K . Lysophosphatidic acid acyltransferase 3 tunes the membrane status of germ cells by incorporating docosahexaenoic acid during spermatogenesis. J Biol Chem. (2017) 292:12065–76. doi: 10.1074/jbc.M117.791277 28578315 PMC5519358

[23] Lee HC Inoue T Sasaki J Kubo T Matsuda S Nakasaki Y . LPIAT1 regulates arachidonic acid content in phosphatidylinositol and is required for cortical lamination in mice. Mol Biol Cell. (2012) 23:4689–700. doi: 10.1091/mbc.E12-09-0673 23097495 PMC3521678

[24] Shindou H Shiraishi S Tokuoka SM Takahashi Y Harayama T Abe T . Relief from neuropathic pain by blocking of the platelet-activating factor-pain loop. FASEB Journal: Off Publ Fed Am Societies For Exp Biol. (2017) 31:2973–80. doi: 10.1096/fj.201601183R 28341636 PMC5471516

[25] Inagaki NF Nakanishi H Ohto T Shindou H Shimizu T . LPCAT3/LPLAT12 deficiency in the liver ameliorates acetaminophen-induced acute liver injury. FASEB Journal: Off Publ Fed Am Societies For Exp Biol. (2024) 38:e23328. doi: 10.1096/fj.202301744R 38019192

[26] Malik MA Saqib MAN Mientjes E Acharya A Alam MR Wallaard I . A loss of function variant in AGPAT3 underlies intellectual disability and retinitis pigmentosa (IDRP) syndrome. Eur J Hum Genetics: EJHG. (2023) 31:1447–54. doi: 10.1038/s41431-023-01475-w 37821758 PMC10689475

[27] Agarwal AK Arioglu E De Almeida S Akkoc N Taylor SI Bowcock AM . AGPAT2 is mutated in congenital generalized lipodystrophy linked to chromosome 9q34. Nat Genet. (2002) 31:21–3. doi: 10.1038/ng880 11967537

[28] Tanosaki T Mikami Y Shindou H Suzuki T Hashidate-Yoshida T Hosoki K . Lysophosphatidylcholine acyltransferase 1 deficiency promotes pulmonary emphysema via apoptosis of alveolar epithelial cells. Inflammation. (2022) 45:1765–79. doi: 10.1007/s10753-022-01659-4 35338433

[29] Adl SM Simpson AG Farmer MA Andersen RA Anderson OR Barta JR . The new higher level classification of eukaryotes with emphasis on the taxonomy of protists. J Eukaryot Microbiol. (2005) 52:399–451. doi: 10.1111/j.1550-7408.2005.00053.x 16248873

[30] Adl SM Bass D Lane CE Lukes J Schoch CL Smirnov A . Revisions to the classification, nomenclature, and diversity of eukaryotes. J Eukaryot Microbiol. (2019) 66:4–119. doi: 10.1111/jeu.12691 30257078 PMC6492006

[31] Madeira F Madhusoodanan N Lee J Eusebi A Niewielska A Tivey ARN . The EMBL-EBI Job Dispatcher sequence analysis tools framework in 2024. Nucleic Acids Res. (2024) 52:W521–5. doi: 10.1093/nar/gkae241 38597606 PMC11223882

[32] Guindon S Dufayard JF Lefort V Anisimova M Hordijk W Gascuel O . New algorithms and methods to estimate maximum-likelihood phylogenies: assessing the performance of PhyML 3.0. Syst Biol. (2010) 59:307–21. doi: 10.1093/sysbio/syq010 20525638

[33] Gouy M Guindon S Gascuel O . SeaView version 4: a multiplatform graphical user interface for sequence alignment and phylogenetic tree building. Mol Biol Evol. (2010) 27:221–4. doi: 10.1093/molbev/msp259 19854763

[34] Benghezal M Roubaty C Veepuri V Knudsen J Conzelmann A . SLC1 and SLC4 encode partially redundant acyl-coenzyme A 1-acylglycerol-3-phosphate O-acyltransferases of budding yeast. J Biol Chem. (2007) 282:30845–55. doi: 10.1074/jbc.M702719200 17675291

[35] Jasieniecka-Gazarkiewicz K Demski K Lager I Stymne S Banas A . Possible role of different yeast and plant lysophospholipid:acyl-CoA acyltransferases (LPLATs) in acyl remodelling of phospholipids. Lipids. (2016) 51:15–23. doi: 10.1007/s11745-015-4102-0 26643989 PMC4700060

[36] Tamaki H Shimada A Ito Y Ohya M Takase J Miyashita M . LPT1 encodes a membrane-bound O-acyltransferase involved in the acylation of lysophospholipids in the yeast Saccharomyces cerevisiae. J Biol Chem. (2007) 282:34288–98. doi: 10.1074/jbc.M704509200 17890783

[37] Riekhof WR Wu J Jones JL Voelker DR . Identification and characterization of the major lysophosphatidylethanolamine acyltransferase in Saccharomyces cerevisiae. J Biol Chem. (2007) 282:28344–52. doi: 10.1074/jbc.m705256200 17652094

[38] Jain S Stanford N Bhagwat N Seiler B Costanzo M Boone C . Identification of a novel lysophospholipid acyltransferase in Saccharomyces cerevisiae. J Biol Chem. (2007) 282:30562–6. doi: 10.1074/jbc.m706326200 17726007

[39] Fukumoto J Yoshida M Tokuoka SM ES HH Miyazaki S Sakura T . Pivotal roles of Plasmodium falciparum lysophospholipid acyltransferase 1 in cell cycle progression and cytostome internalization. Commun Biol. (2025) 8:142. doi: 10.1038/s42003-025-07564-4 39880906 PMC11779973

[40] Kawana H Kano K Shindou H Inoue A Shimizu T Aoki J . An accurate and versatile method for determining the acyl group-introducing position of lysophospholipid acyltransferases. Biochim Biophys Acta Mol Cell Biol Lipids. (2019) 1864:1053–60. doi: 10.1016/j.bbalip.2019.02.008 30853650

[41] Imae R Inoue T Nakasaki Y Uchida Y Ohba Y Kono N . LYCAT, a homologue of C. elegans acl-8, acl-9, and acl-10, determines the fatty acid composition of phosphatidylinositol in mice. J Lipid Res. (2012) 53:335–47. doi: 10.1194/jlr.M018655 22172515 PMC3276457

[42] Kawana H Ozawa M Shibata T Onishi H Sato Y Kano K . Identification and characterization of LPLAT7 as an sn-1-specific lysophospholipid acyltransferase. J Lipid Res. (2022) 63:100271. doi: 10.1016/j.jlr.2022.100271 36049524 PMC9587406

[43] Sato T Umebayashi S Senoo N Akahori T Ichida H Miyoshi N . LPGAT1/LPLAT7 regulates acyl chain profiles at the sn-1 position of phospholipids in murine skeletal muscles. J Biol Chem. (2023) 299:104848. doi: 10.1016/j.jbc.2023.104848 37217003 PMC10285227

[44] Caforio A Driessen AJM . Archaeal phospholipids: structural properties and biosynthesis. Biochim Biophys Acta Mol Cell Biol Lipids. (2017) 1862:1325–39. doi: 10.1016/j.bbalip.2016.12.006 28007654

[45] Hashidate-Yoshida T Harayama T Hishikawa D Morimoto R Hamano F Tokuoka SM . Fatty acid remodeling by LPCAT3 enriches arachidonate in phospholipid membranes and regulates triglyceride transport. eLife. (2015) 21:6328. doi: 10.7554/eLife.06328 25898003 PMC4436788

[46] Rong X Wang B Dunham MM Hedde PN Wong JS Gratton E . Lpcat3-dependent production of arachidonoyl phospholipids is a key determinant of triglyceride secretion. eLife. (2015) 4:6557. doi: 10.7554/eLife.06557 25806685 PMC4400582

[47] Shimizu T . Lipid mediators in health and disease: enzymes and receptors as therapeutic targets for the regulation of immunity and inflammation. Annu Rev Pharmacol. (2009) 49:123–50. doi: 10.1146/annurev.pharmtox.011008.145616 18834304

[48] Prescott SM Zimmerman GA McIntyre TM . Platelet-activating factor. J Biol Chem. (1990) 265:17381–4. doi: 10.1016/S0021-9258(18)38167-5 2170377

[49] Ishii S Shimizu T . Platelet-activating factor (PAF) receptor and genetically engineered PAF receptor mutant mice. Prog Lipid Res. (2000) 39:41–82. doi: 10.1016/s0163-7827(99)00016-8 10729607

[50] Snyder F . Platelet-activating factor and its analogs: metabolic pathways and related intracellular processes. Biochim Biophys Acta. (1995) 1254:231–49. doi: 10.1016/0005-2760(94)00192-2 7857964

[51] Shindou H Ishii S Uozumi N Shimizu T . Roles of cytosolic phospholipase A(2) and platelet-activating factor receptor in the Ca-induced biosynthesis of PAF. Biochem Biophys Res Commun. (2000) 271:812–7. doi: 10.1006/bbrc.2000.2723 10814544

[52] Wykle RL Malone B Snyder F . Enzymatic synthesis of 1-alkyl-2-acetyl-sn-glycero-3-phosphocholine, a hypotensive and platelet-aggregating lipid. J Biol Chem. (1980) 255:10256–60. doi: 10.1016/s0021-9258(19)70457-8 7430122

[53] Shindou H Ishii S Yamamoto M Takeda K Akira S Shimizu T . Priming effect of lipopolysaccharide on acetyl-coenzyme A:lyso-platelet-activating factor acetyltransferase is MyD88 and TRIF independent. J Immunol. (2005) 175:1177–83. doi: 10.4049/jimmunol.175.2.1177 16002720

[54] Shindou H Hishikawa D Nakanishi H Harayama T Ishii S Taguchi R . A single enzyme catalyzes both platelet-activating factor production and membrane biogenesis of inflammatory cells. Cloning and characterization of acetyl-CoA:LYSO-PAF acetyltransferase. J Biol Chem. (2007) 282:6532–9. doi: 10.1074/jbc.m609641200 17182612

[55] Suzuki T Taketomi Y Yanagida K Yoshida-Hashidate T Nagase T Murakami M . Re-evaluation of the canonical PAF pathway in cutaneous anaphylaxis. Biochim Biophys Acta Mol Cell Biol Lipids. (2025) 1870:159563. doi: 10.1016/j.bbalip.2024.159563 39332666

[56] Morimoto R Shindou H Oda Y Shimizu T . Phosphorylation of lysophosphatidylcholine acyltransferase 2 at Ser34 enhances platelet-activating factor production in endotoxin-stimulated macrophages. J Biol Chem. (2010) 285:29857–62. doi: 10.1074/jbc.M110.147025 20663880 PMC2943291

[57] Morimoto R Shindou H Tarui M Shimizu T . Rapid production of platelet-activating factor is induced by protein kinase Calpha-mediated phosphorylation of lysophosphatidylcholine acyltransferase 2 protein. J Biol Chem. (2014) 289:15566–76. doi: 10.1074/jbc.M114.558874 24742674 PMC4140912

[58] Nakanishi H Shindou H Hishikawa D Harayama T Ogasawara R Suwabe A . Cloning and characterization of mouse lung-type acyl-CoA:lysophosphatidylcholine acyltransferase 1 (LPCAT1): expression in alveolar type II cells and possible involvement in surfactant production. J Biol Chem. (2006) 281:20140–7. doi: 10.1074/jbc.m600225200 16704971

[59] Chen X Hyatt BA Mucenski ML Mason RJ Shannon JM . Identification and characterization of a lysophosphatidylcholine acyltransferase in alveolar type II cells. PNAS. (2006) 103:11724–9. doi: 10.1073/pnas.0604946103 16864775 PMC1544237

[60] Harayama T Eto M Shindou H Kita Y Otsubo E Hishikawa D . Lysophospholipid acyltransferases mediate phosphatidylcholine diversification to achieve the physical properties required *in vivo*. Cell Metab. (2014) 20:295–305. doi: 10.1016/j.cmet.2014.05.019 24981836

[61] Bridges JP Ikegami M Brilli LL Chen X Mason RJ Shannon JM . LPCAT1 regulates surfactant phospholipid synthesis and is required for transitioning to air breathing in mice. J Clin Invest. (2010) 120:1736–48. doi: 10.1172/JCI38061 20407208 PMC2860922

[62] Harayama T Shindou H Shimizu T . Biosynthesis of phosphatidylcholine by human lysophosphatidylcholine acyltransferase 1. J Lipid Res. (2009) 50:1824–31. doi: 10.1194/jlr.M800500-JLR200 19383981 PMC2724784

[63] Hama K Fujiwara Y Imai K Kusakabe Y Hayashi Y Takashima S . Phosphatidylcholine with C26:0 moiety, a precursor of a diagnostic marker for X-ALD, is synthesized by LPLAT10/LPEAT2. J Lipid Res. (2026) 67:100973. doi: 10.1016/j.jlr.2025.100973 41478358 PMC12859765

[64] Kawana H Kataoka R Sato Y Ohnishi H Iwama T Shimanaka Y . LPLAT10/LPEAT2 produces atypical phospholipids with an unsaturated fatty acid at the sn-1 position. J Lipid Res. (2026) 67(4):101007. doi: 10.1016/j.jlr.2026.101007 41740885 PMC13051972

[65] Hishikawa D Yanagida K Nagata K Kanatani A Iizuka Y Hamano F . Hepatic levels of DHA-containing phospholipids instruct SREBP1-mediated synthesis and systemic delivery of polyunsaturated fatty acids. iScience. (2020) 23:101495. doi: 10.1016/j.isci.2020.101495 32891885 PMC7481256

[66] Koeberle A Shindou H Harayama T Shimizu T . Role of lysophosphatidic acid acyltransferase 3 for the supply of highly polyunsaturated fatty acids in TM4 Sertoli cells. FASEB Journal: Off Publ Fed Am Societies For Exp Biol. (2010) 24:4929–38. doi: 10.1096/fj.10-162818 20705908

[67] Eto M Shindou H Shimizu T . A novel lysophosphatidic acid acyltransferase enzyme (LPAAT4) with a possible role for incorporating docosahexaenoic acid into brain glycerophospholipids. Biochem Biophys Res Commun. (2014) 443:718–24. doi: 10.1016/j.bbrc.2013.12.043 24333445

[68] Cao J Shan D Revett T Li D Wu L Liu W . Molecular identification of a novel mammalian brain isoform of acyl-CoA:lysophospholipid acyltransferase with prominent ethanolamine lysophospholipid acylating activity, LPEAT2. J Biol Chem. (2008) 283:19049–57. doi: 10.1074/jbc.m800364200 18458083

[69] Eto M Shindou H Yamamoto S Tamura-Nakano M Shimizu T . Lysophosphatidylethanolamine acyltransferase 2 (LPEAT2) incorporates DHA into phospholipids and has possible functions for fatty acid-induced cell death. Biochem Biophys Res Commun. (2020) 526:246–52. doi: 10.1016/j.bbrc.2020.03.074 32204912

[70] Shimizu K Ono M Mikamoto T Urayama Y Yoshida S Hase T . Overexpression of lysophospholipid acyltransferase, LPLAT10/LPCAT4/LPEAT2, in the mouse liver increases glucose-stimulated insulin secretion. FASEB Journal: Off Publ Fed Am Societies For Exp Biol. (2024) 38:e23425. doi: 10.1096/fj.202301594RR 38226852

[71] Serhan CN . Pro-resolving lipid mediators are leads for resolution physiology. Nature. (2014) 510:92–101. doi: 10.1038/nature13479 24899309 PMC4263681

[72] Hishikawa D Shindou H Kobayashi S Nakanishi H Taguchi R Shimizu T . Discovery of a lysophospholipid acyltransferase family essential for membrane asymmetry and diversity. PNAS. (2008) 105:2830–5. doi: 10.1073/pnas.0712245105 18287005 PMC2268545

[73] Gijón M Riekhof W Zarini S Murphy R Voelker D . Lysophospholipid acyltransferases and arachidonate recycling in human neutrophils. J Biol Chem. (2008) 283:30235–45. doi: 10.1074/jbc.M806194200 PMC2573059 18772128

[74] Kelso C Maccarone AT de Kroon A Mitchell TW Renne MF . Temperature adaptation of yeast phospholipid molecular species at the acyl chain positional level. FEBS Lett. (2025) 599:530–44. doi: 10.1002/1873-3468.15060 39673166 PMC11848023

[75] Horejsi V Hrdinka M . Membrane microdomains in immunoreceptor signaling. FEBS Lett. (2014) 588:2392–7. doi: 10.1016/j.febslet.2014.05.047 24911201

[76] Mi-Ichi F Tsugawa H Yoshida H Arita M . Unique features of Entamoeba histolytica glycerophospholipid metabolism; has the E. histolytica lipid metabolism network evolved through gene loss and gain to enable parasitic life cycle adaptation? mSphere. (2023) 8:e0017423. doi: 10.1128/msphere.00174-23 37584599 PMC10597341

[77] Grevengoed TJ Klett EL Coleman RA . Acyl-CoA metabolism and partitioning. Annu Rev Nutr. (2014) 34:1–30. doi: 10.1146/annurev-nutr-071813-105541 24819326 PMC5881898

[78] Murakami M Sato H Taketomi Y . Updating phospholipase A2 biology. Biomolecules. (2020) 10:1–32. doi: 10.3390/biom10101457 33086624 PMC7603386

[79] Kita Y Shindou H Shimizu T . Cytosolic phospholipase A2 and lysophospholipid acyltransferases. Biochim Biophys Acta Mol Cell Biol Lipids. (2019) 1864:838–45. doi: 10.1016/j.bbalip.2018.08.006 30905348

[80] Niu W Vu T Du G Bogdanov M Zheng L . Lysophospholipid remodeling mediated by the LplT and Aas protein complex in the bacterial envelope. J Biol Chem. (2024) 300:107704. doi: 10.1016/j.jbc.2024.107704 39173951 PMC11416262

