# Supplementary material for: An evolutionary analysis of lysophospholipid acyltransferases generating membrane phospholipid diversity
Source: Front Immunol. 2026 Aug 10;17:1838657. doi: 10.3389/fimmu.2026.1838657 (PMC13500770; doi:10.3389/fimmu.2026.1838657)
Supplement: Supplementary file 1 [file DataSheet1.pdf]

					Organisms	Name	Fig. 2	Fig. 3	Fig. 4	Number of clusters	
										AGPAT	MBOAT
Prokaryotes	Bacteria				Escherichia coli K-12	EcKLPLAT	○		○	2	0
					Escherichia coli O157:H7 str. G5101	EcOLPLAT			○	3	0
Eukaryotes	Opisthokonta	Metazoa	Invertebrates	Porifera	Amphimedon queenslandica	AqLPLAT		○		2	1
				Cnidaria	Nematostella vectensis	NvLPLAT		○		1	1
				Mollusca	Magallana gigas	MgLPLAT		○		4	2
				Arthropoda	Drosophila melanogaster	DmLPLAT	○	○		3	3
				Nematoda	Caenorhabditis elegans	CeLPLAT		○		9	3
				Annelida	Capitella teleta	CtLPLAT		○		3	2
			Vertebrates	Fishes	Danio rerio	DrLPLAT		○		9	5
				Amphibians	Xenopus tropicalis	XtLPLAT		○		10	4
				Reptiles	Anolis carolinensis	AcLPLAT		○		10	4
				Birds	Gallus gallus	GgLPLAT		○		9	3
				Mammals	Homo sapiens	HsLPLAT	○	○	○	10	4
		Fungi		Ascomycota	Saccharomyces cerevisiae S288C	ScLPLAT	○			1	1
		Choanoflagellata			Monosiga brevicollis MX1	MbLPLAT	○			1	0
	Amoebozoa				Entamoeba dispar SAW760	EdLPLAT	○		○	2	4
					Entamoeba histolytica HM-1:IMSS	EhLPLAT			○	2	5
	SAR	Alveolata			Plasmodium falciparum 3D7	PfLPLAT	○			1	0
	Archaeplastida	Angiosperms			Arabidopsis thaliana	AtLPLAT	○			7	2
	Excavata				Trypanosoma cruzi	TcLPLAT	○			1	0

Supplementary Table 1. Selected organisms and LPLAT names.

LPLAT candidates were identified across prokaryotic and eukaryotic lineages, including bacteria and the major eukaryotic supergroups (Opisthokonta, Amoebozoa, SAR, Archaeplastida, and Excavata), based on the classification framework of Adl et al. (14, 15). LPLAT nomenclature used in this study is indicated. Organisms highlighted by circles were analyzed in Figs. 2–4. LPLATs were not found in Archaea.

Name	No	Acc. No.	No	Acc. No.	No	Acc. No.	No	Acc. No.	No	Acc. No.	No	Acc. No.	No	Acc. No.	No	Acc. No.	No	Acc. No.	No	Acc. No.
EcKLPLAT	1	AIZ52813	2	AKK15969																
EcOLPLAT	1	EFX09666	2	EFX08826	3	EFX09355														
AqLPLAT	1	XP_019853566	2	XP_011403842																
NvLPLAT	1	XP_048587027																		
MgLPLAT	1	XP_011449105	2	XP_011416667	3	XP_011422898	4	XP_011457228												
DmLPLAT	1	NP_572828	2	NP_609231	3	NP_001036265														
CeLPLAT	1	NP_510606	2	NP_001256175	3	NP_001348648	4	NP_504644	5	Q20800	6	NP_491479	7	NP_509260	8	NP_502370	9	NP_001371003		
CtLPLAT	1	ELT98524	2	ELU12181	3	ELU10392														
DrLPLAT			2	NP_001071200	3	NP_998590	4	NP_998157	5	NP_001070213	6	NP_998435	7	NP_001025129	8	NP_001037806	9	NP_001018492	10	NP_991122
XtLPLAT	1	XP_012823272	2	NP_001123741	3	NP_001008119	4	NP_001011265	5	NP_001015964	6	NP_001135517	7	XP_017949604	8	XP_002933165	9	XP_012815924	10	NP_001037863
AcLPLAT	1	XP_062829728	2	XP_062816491	3	XP_008105662	4	XP_062832607	5	XP_003215489	6	XP_003216108	7	XP_016846149	8	XP_016848437	9	XP_003222971	10	XP_062813319
GgLPLAT	1	XP_040543510	2	XP_001235300	3	XP_004934656	4	XP_040523863	5	XP_040522440	6	XP_025004424	7	NP_001026220	8	XP_004935228	9	NP_001025739	10	
HsLPLAT	1	NP_006402	2	NP_006403	3	NP_064517	4	NP_064518	5	NP_060831	6	NP_001002257	7	NP_055688	8	NP_079106	9	NP_060309	10	NP_705841
ScLPLAT	1	NP_010231																		
MbLPLAT	1	XP_001743302																		
EdLPLAT	1	XP_001739767	2	XP_001734633																
EhLPLAT	1	XP_655766	2	XP_649422																
PfLPLAT	1	XP_001348595																		
AtLPLAT	1	NP_001030724	2	AAN65123	3	AAD55275	4	NP_567052	5	NP_175537	6	NP_565249	7	Q8S8S2						
TcLPLAT	1	XP_817630																		

Supplement Table 2. Candidate LPLATs from the AGPAT family

Name	No	Acc. No.	No	Acc. No.	No	Acc. No.	No	Acc. No.	No	Acc. No.
EcKLPLAT										
EcOLPLAT										
AqLPLAT	3	XP_019855423								
NvLPLAT	2	XP_048575357								
MgLPLAT	5	XP_034333714	6	XP_011416786						
DmLPLAT	4	NP_995637	5	NP_524157	6	NP_610546				
CeLPLAT	10	NP_001370561	11	NP_001022735	12	NP_001255544				
CtLPLAT	4	ELT92151	5	ELT92172						
DrLPLAT	11	NP_956831	12	XP_073781106	13	XP_073783893	14	NP_001007397	15	NP_001265531
XtLPLAT	11	XP_012822891	12	XP_002941254	13	NP_001096155	14	XP_002940346		
AcLPLAT	11	XP_062840076	12	XP_003223803	13	XP_008115909	14	XP_003219829		
GgLPLAT	11		12	XP_040517541	13	NP_001026261	14	XP_046767333		
HsLPLAT	11	NP_077274	12	NP_005759	13	NP_620154	14	NP_001073949		
ScLPLAT	2	NP_014818								
MbLPLAT										
EdLPLAT	3	XP_001734903	4	XP_001733896	5	XP_001735834	6	XP_001736261		
EhLPLAT	3	XP_653096	4	XP_652418	5	XP_651748	6	XP_655691	7	XP_648994
PfLPLAT										
AtLPLAT	8	NP_176493	9	NP_172724						
TcLPLAT										

Supplement Table 3. Candidate LPLATs from the MBOAT family

Family	LPLAT	Substrates	
		LPL (Lyso-PL)	Fatty acid of Acyl-CoA
AGPAT	LPLAT1	LPA	broad
	LPLAT2	LPA	broad
	LPLAT3	LPA,LPI	C22:6
	LPLAT4	LPA	C18:1
	LPLAT5	LPA	C18:1
	LPLAT6	LPI, LCL	C18:0
	LPLAT7	LPC, LPE	C18:0
	LPLAT8	LPC	C16:0
	LPLAT9	LPC	C2:0
	LPLAT10	LPC, LPE	unsaturated, C26:0
MBOAT	LPLAT11	LPI, LCL	C20:4
	LPLAT12	LPC, LPS, LPE	C18:2, C20:4
	LPLAT13	LPC, LPE	C18:1
	LPLAT14	LPS, LPE	C18:1

Supplementary Table 4. Substrate preferences of mouse and human LPLATs.

Representative examples are shown. LPA, lyso-PA; LPC, lyso-PC; LPE, lyso-PE; LPI, lyso-PI; LPS, lyso-PS, and LCL, lyso-CL.

Supplementary Fig. 1A

```
                        1
EckLPLAT1_AIZ52813      ---------- ---------- ---------- ---------- ---------- ----------
EckLPLAT2_AKK15969      ---------- ---------- ---------- ---------- ---------- ----------
DmLPLAT1_NP_572828      ---------- ---------- ---------- ---------- ---------- -MI-------
DmLPLAT2_NP_609231      ---------- ---------- ---------- ---------- ---------- ----------
DmLPLAT3_NP_001036265   MTTSTIKP-- TG--GDEVPP A--GVVGN-- -VADVGV--- AASLTKRDS- -----EED--
ScLPLAT1_NP_010231      ---------- ---------- ---------- ---------- ---------- ----------
MbLPLAT1_XP_001743302   ---------- ---------- ---------- ---------- ---------- ----------
EdLPLAT1_XP_001739767   ---------- ---------- ---------- ---------- ---------- -MILYFRQPS
EdLPLAT2_XP_001734633   ---------- ---------- ---------- ---------- ---------- ----------
PfLPLAT1_XP_001348595   ---------- ---------- ---------- ---------- ---------- -MEIDVKKKY
AtLPLAT1_NP_001030724   ---------- ---------- ---------- ---------- ---------- ----------
AtLPLAT2_AAN65123       ---------- ---------- ---------- ---------- ---------- ----------
AtLPLAT3_AAD55275       ---------- ---------- ---------- ---------- ---------- ----------
AtLPLAT4_NP_567052      ---------- ---------- ---------- ---------- ---------- ----------
AtLPLAT5_NP_175537      ---------- ---------- ---------- ---------- ---------- ----------
AtLPLAT6_NP_565249      MES-ELKD-- LN--SNSNPP S--SKEDRPL LKSESDL--- AAAIEELDK- -----KFAPY
AtLPLAT7_Q8S8S2         MADPDLSSPL IHHQSSDQPE VVISIADDD- -DDESGLNLL PAVVDPRVSR GFEFDHLNPY
TcLPLAT1_XP_817630      ---------- ---------- ---------- ---------- ---------- ----------
HsLPLAT1_NP_006402      ---------- ---------- ---------- ---------- ---------- -MDL------
HsLPLAT2_NP_006403      ---------- ---------- ---------- ---------- ---------- -MEL------
HsLPLAT3_NP_064517      ---------- ---------- ---------- ---------- ---------- ----------
HsLPLAT4_NP_064518      ---------- ---------- ---------- ---------- ---------- ----------
HsLPLAT5_NP_060831      ---------- ---------- ---------- ---------- ---------- ----------
HsLPLAT6_NP_001002257   ---------- ---------- ---------- ---------- ---------- ----------
HsLPLAT7_NP_055688      ---------- ---------- ---------- ---------- ---------- ----------
HsLPLAT8_NP_079106      MRLRGCGPRA AP-------- ---------- ---------- ASSAGASDA- -----R----
HsLPLAT9_NP_060309      --MSRCAQAA EV--AATVPG A--G------ -VGNVGL--- RPPMVPRQA- -----S----
HsLPLAT10_NP_705841     ---------- ---------- ---------- --MSQGS--- PGDWAPLDP- -----T----

                       61
EckLPLAT1_AIZ52813      ---------- ---------- ---------- ---------- ---------- -----MLYIF
EckLPLAT2_AKK15969      MRFFS---AL S---MA---- --------NL LNKFIMTRIL A--AITLLLS IVLT------
DmLPLAT1_NP_572828      ---------- ---------- ----TMTSFI ELLGL--FLL LMLPFL--YE -TNHIFRYYF
DmLPLAT2_NP_609231      ---------- ---------- -----MACTC EVIGL--ACV VA--LI--LS -ATAKAPYQM
DmLPLAT3_NP_001036265   -TW-----AS KGYNYINPFV HRIE-IDSHI EVAKIYVLTV LLLPIRVVGC VLSLISAWMF
ScLPLAT1_NP_010231      ---------- ---------- ---------- ---------- --------MS -VIGRFLYYL
MbLPLAT1_XP_001743302   ---------- ---------- ---------- --MY------ ---------- ----------
EdLPLAT1_XP_001739767   KRVKK---MI R---GE---- --------SS ERMVDTTGIK D-------KI ILILKYYFAY
EdLPLAT2_XP_001734633   ---------- ---------- ---------- ---MKCNELI N-------KI CWGLKIYFAY
PfLPLAT1_XP_001348595   KHS------- ---------- ---NILIRLI LTIYI--YIV MFLTLS--F- ----SLICQL
AtLPLAT1_NP_001030724   --------ME K---KSVP-- -----NSDKL SLIRVLRGII C--LMVLVST AFMMLIFWGF
AtLPLAT2_AAN65123       MEVCG---DL K---SDNL-- -----KNRPL TPLRILRGLM I--LLVFLST AFMFLLYFAP
AtLPLAT3_AAD55275       MEVCG---DL K---SDNL-- -----KNRPL TPLRILRGLM I--LLVFLST AFMFLLYFAP
AtLPLAT4_NP_567052      ---------- ---------- ---------- ---------- -------MVI AAAVIVPLGL
AtLPLAT5_NP_175537      ---------- ---------- ---------- ---------- ------MKIP AALVFIPVGV
AtLPLAT6_NP_565249      ART-----DL YGTMGLGPFP MT-------- ENIKLAVALV TLVPLRFLLS MSILLLYYLI
AtLPLAT7_Q8S8S2         GFLSESEPPV LGPTTVDPFR NNTPGVSGLY EAIKLVIC-L PIALIRLVLF AASLAVGYLA
TcLPLAT1_XP_817630      MSY------- ---------- ---HTVIRSI VTAYV----I LLLAVH--W- ----IIAWIL
HsLPLAT1_NP_006402      --W------- ---------- ---PGAWMLL LLLFL--LLL FLLPTL--WF -CSPSAKYFF
HsLPLAT2_NP_006403      --W------- ---------- ---PCLAAAL LLLLL--LVQ ---------- -LSRAAEFYA
HsLPLAT3_NP_064517      ---------- ---------- ---------- ---------M G--LLAFLKT QFVLHLLVGF
HsLPLAT4_NP_064518      ---------- ---------- ---------- ---------M D--LAGLLKS QFLCHLVFCY
HsLPLAT5_NP_060831      --------ML L---SLVL-- ---------- -HTYSMRYLL P--SVVLLGT APTYVLAWGV
HsLPLAT6_NP_001002257   ---------- ---------- ---------- ---------- ---MVSWKGI YFILTLFWGS
HsLPLAT7_NP_055688      ---------- ---------- ---------- --MAITLEEA P--WLGWLLV KALMRFAF--
HsLPLAT8_NP_079106      ---------L LAPPGRNPFV HELR-LSAL- QKAQVALMTL TLFPVRLLVA AAMMLLAWPL
HsLPLAT9_NP_060309      ---------F FPPPVPNPFV QQTQ-IGSA- RRVQIVLLGI ILLPIRVLLV ALILLLAWPF
HsLPLAT10_NP_705841     ---------P GPPASPNPFV HELH-LSRL- QRVKFCLLGA LLAPIRVLLA FIVLFLLWPF
```

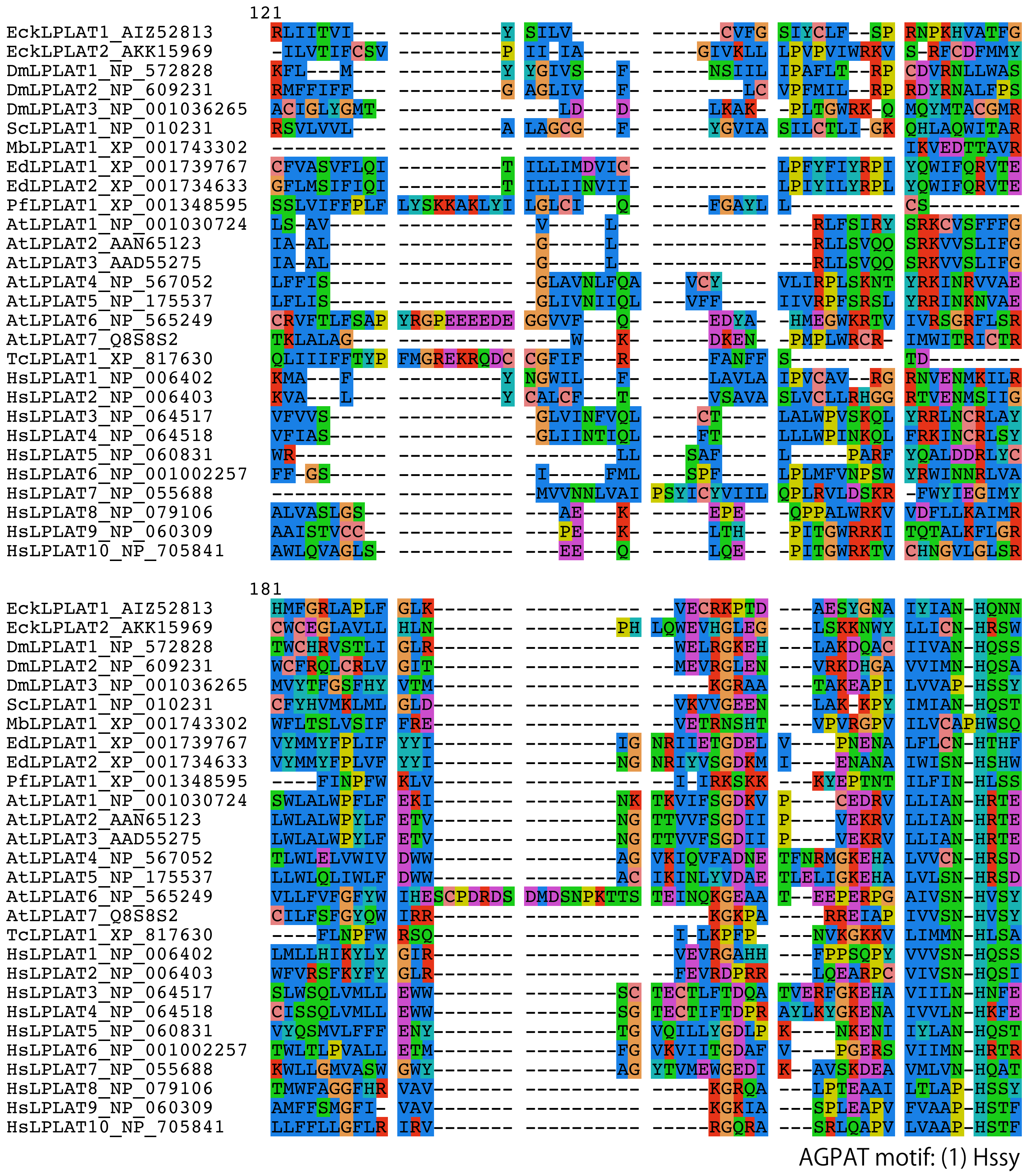
121
EckLPLAT1_AIZ52813 RLIITVI--- ----------Y SILV------ -------CVFG SIYCLF--SP RNPKHVATFG
EckLPLAT2_AKK15969 -ILVTIFCSV ----------P II-IA----- -----GIVKLL LPVPVIWRKV S-RFCDFMMY
DmLPLAT1_NP_572828 KFL---M--- ----------Y YGIVS---F- ------NSIIL IPAFLT--RP CDVRNLLWAS
DmLPLAT2_NP_609231 RMFFIFF--- ----------G AGLIV---F- ---------LC VPFMIL--RP RDYRNALFPS
DmLPLAT3_NP_001036265 ACIGLYGMT- ----------- ---LD---D- ------LKAK- -PLTGWRK-Q MQYMTACGMR
ScLPLAT1_NP_010231 RSVLVVL--- ----------A LAGCG---F- ------YGVIA SILCTLI-GK QHLAQWITAR
MbLPLAT1_XP_001743302 ---------- ----------- ---------- ----------- ---------- IKVEDTTAVR
EdLPLAT1_XP_001739767 CFVASVFLQI ----------T ILLIMDVIC- ----------- LPFYFIYRPI YQWIFQRVTE
EdLPLAT2_XP_001734633 GFLMSIFIQI ----------T ILLIINVII- ----------- LPIYILYRPL YQWIFQRVTE
PfLPLAT1_XP_001348595 SSLVIFFPLF LYSKKAKLYI LGLCI---Q- ------FGAYL L--------- CS--------
AtLPLAT1_NP_001030724 LS-AV----- ----------- -V-----L-- ----------- ---RLFSIRY SRKCVSFFFG
AtLPLAT2_AAN65123 IA-AL----- ----------- -G-----L-- ----------- ---RLLSVQQ SRKVVSLIFG
AtLPLAT3_AAD55275 IA-AL----- ----------- -G-----L-- ----------- ---RLLSVQQ SRKVVSLIFG
AtLPLAT4_NP_567052 LFFIS----- ----------- -GLAVNLFQA ---VCY----- VLIRPLSKNT YRKINRVVAE
AtLPLAT5_NP_175537 LFLIS----- ----------- -GLIVNIIQL ---VFF----- IIVRPFSRSL YRRINKNVAE
AtLPLAT6_NP_565249 CRVFTLFSAP YRGPEEEEDE GGVVF---Q- ------EDYA- -HMEGWKRTV IVRSGRFLSR
AtLPLAT7_Q8S8S2 TKLALAG--- ----------- ----W---K- ------DKEN- -PMPLWRCR- IMWITRICTR
TcLPLAT1_XP_817630 QLIIIFFTYP FMGREKRQDC CGFIF---R- ------FANFF S--------- TD--------
HsLPLAT1_NP_006402 KMA---F--- ----------Y NGWIL---F- ------LAVLA IPVCAV--RG RNVENMKILR
HsLPLAT2_NP_006403 KVA---L--- ----------Y CALCF---T- ------VSAVA SLVCLLRHGG RTVENMSIIG
HsLPLAT3_NP_064517 VFVVS----- ----------- -GLVINFVQL ----CT----- LALWPVSKQL YRRLNCRLAY
HsLPLAT4_NP_064518 VFIAS----- ----------- -GLIINTIQL ----FT----- LLLWPINKQL FRKINCRLSY
HsLPLAT5_NP_060831 WR-------- ----------- --------LL ---SAF----- L-----PARF YQALDDRLYC
HsLPLAT6_NP_001002257 FF-GS----- ----------- -I-----FML ---SPF----- LPLMFVNPSW YRWINNRLVA
HsLPLAT7_NP_055688 ---------- ----------- -MVVNNLVAI PSYICYVIIL QPLRVLDSKR -FWYIEGIMY
HsLPLAT8_NP_079106 ALVASLGS-- ----------- ---AE---K- ------EPE-- -QPPALWRKV VDFLLKAIMR
HsLPLAT9_NP_060309 AAISTVCC-- ----------- ---PE---K- ------LTH-- -PITGWRRKI TQTALKFLGR
HsLPLAT10_NP_705841 AWLQVAGLS- ----------- ---EE---Q- ------LQE-- -PITGWRKTV CHNGVLGLSR
181
EckLPLAT1_AIZ52813 HMFGRLAPLF GLK------- ----------- --VECRKPTD ---AESYGNA IYIAN-HQNN
EckLPLAT2_AKK15969 CWCEGLAVLL HLN------- ---------PH LQWEVHGLEG ---LSKKNWY LLICN-HRSW
DmLPLAT1_NP_572828 TWCHRVSTLI GLR------- ----------- --WELRGKEH ---LAKDQAC IIVAN-HQSS
DmLPLAT2_NP_609231 WCFRQLCRLV GIT------- ----------- --MEVRGLEN ---VRKDHGA VVIMN-HQSA
DmLPLAT3_NP_001036265 MVYTFGSFHY VTM------- ----------- ------KGRAA ---TAKEAPI LVVAP-HSSY
ScLPLAT1_NP_010231 CFYHVMKLML GLD------- ----------- --VKVVGEEN ---LAK-KPY IMIAN-HQST
MbLPLAT1_XP_001743302 WFLTSLVSIF FRE------- ----------- --VETRNSHT ---VPVRGPV ILVCAPHWSQ
EdLPLAT1_XP_001739767 VYMMYFPLIF YYI------- ---------IG NRIIETGDEL V----PNENA LFLCN-HTHF
EdLPLAT2_XP_001734633 VYMMYFPLVF YYI------- ---------NG NRIYVSGDKM I----ENANA IWISN-HSHW
PfLPLAT1_XP_001348595 ----FINPFW KLV------- ----------- --I-IRKSKK ---KYEPTNT ILFIN-HLSS
AtLPLAT1_NP_001030724 SWLALWPFLF EKI------- ---------NK TKVIFSGDKV P----CEDRV LLIAN-HRTE
AtLPLAT2_AAN65123 LWLALWPYLF ETV------- ---------NG TTVVFSGDII P----VEKRV LLIAN-HRTE
AtLPLAT3_AAD55275 LWLALWPYLF ETV------- ---------NG TTVVFSGDII P----VEKRV LLIAN-HRTE
AtLPLAT4_NP_567052 TLWLELVWIV DWW------- ---------AG VKIQVFADNE TFNRMGKEHA LVVCN-HRSD
AtLPLAT5_NP_175537 LLWLQLIWLF DWW------- ---------AC IKINLYVDAE TLELIGKEHA LVLSN-HRSD
AtLPLAT6_NP_565249 VLLFVFGFYW IHESCPDRDS DMDSNPKTTS TEINQKGEAA T--EEPERPG AIVSN-HVSY
AtLPLAT7_Q8S8S2 CILFSFGYQW IRR------- ----------- ------KGKPA ----RREIAP IVVSN-HVSY
TcLPLAT1_XP_817630 ----FLNPFW RSQ------- ----------- --I-LKPFP- ---NVKGKKV LIMMN-HLSA
HsLPLAT1_NP_006402 LMLLHIKYLY GIR------- ----------- --VEVRGAHH ---FPPSQPY VVVSN-HQSS
HsLPLAT2_NP_006403 WFVRSFKYFY GLR------- ----------- --FEVRDPRR ---LQEARPC VIVSN-HQSI
HsLPLAT3_NP_064517 SLWSQLVMLL EWW------- ---------SC TECTLFTDQA TVERFGKEHA VIILN-HNFE
HsLPLAT4_NP_064518 CISSQLVMLL EWW------- ---------SG TECTIFTDPR AYLKYGKENA IVVLN-HKFE
HsLPLAT5_NP_060831 VYQSMVLFFF ENY------- ---------TG VQILLYGDLP K----NKENI IYLAN-HQST
HsLPLAT6_NP_001002257 TWLTLPVALL ETM------- ---------FG VKVIITGDAF V----PGERS VIIMN-HRTR
HsLPLAT7_NP_055688 KWLLGMVASW GWY------- ---------AG YTVMEWGEDI K--AVSKDEA VMLVN-HQAT
HsLPLAT8_NP_079106 TMWFAGGFHR VAV------- ----------- ------KGRQA ---LPTEAAI LTLAP-HSSY
HsLPLAT9_NP_060309 AMFFSMGFI- VAV------- ----------- ------KGKIA ---SPLEAPV FVAAP-HSTF
HsLPLAT10_NP_705841 LLFFLLGFLR IRV------- ----------- ------RGQRA ---SRLQAPV LVAAP-HSTF
AGPAT motif: (1) Hssy

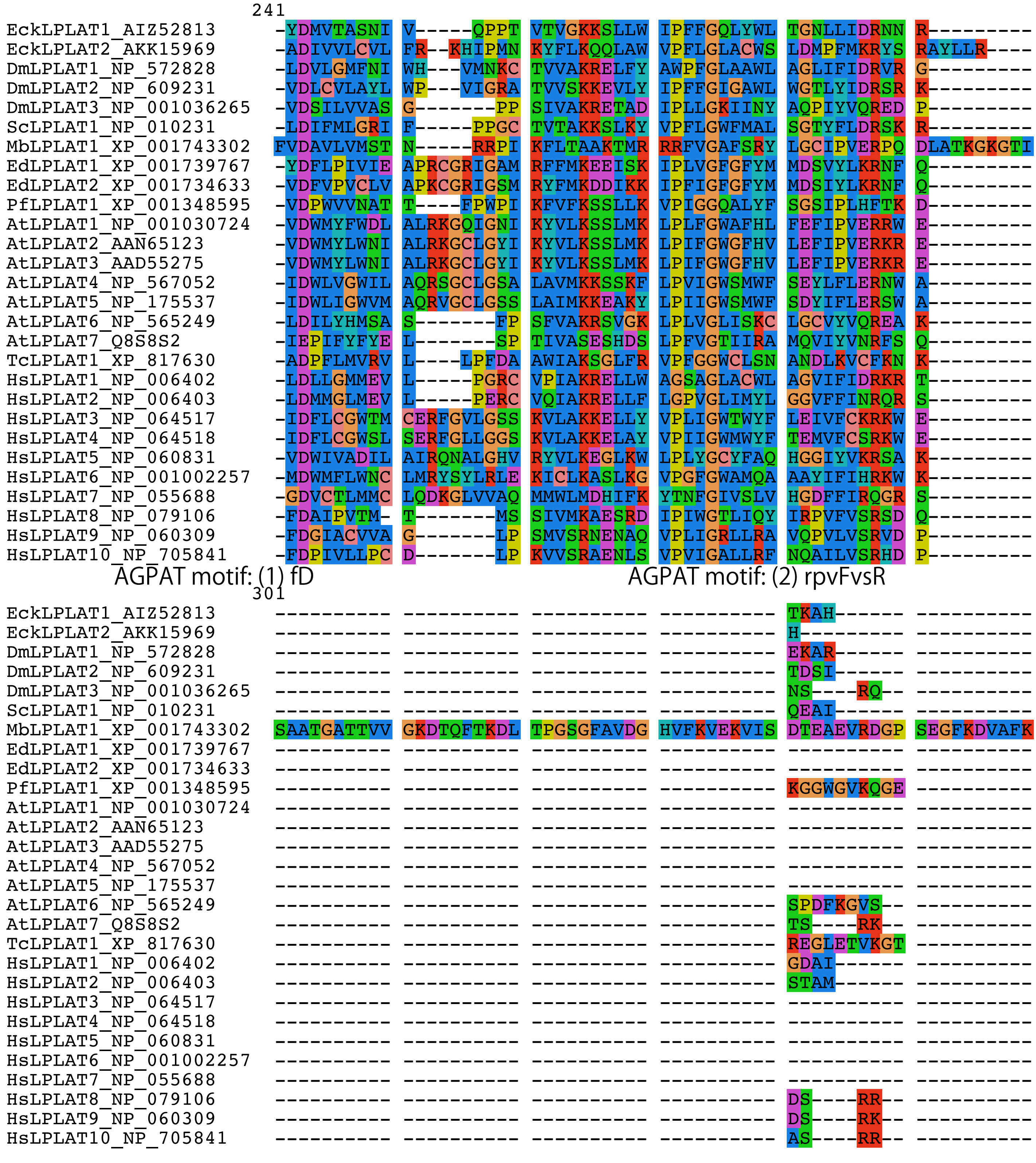

241
EckLPLAT1_AIZ52813 -YDMVTASNI V-----QPPT VTVGKKSLLW IPFFGQLYWL TGNLLIDRNN R---------
EckLPLAT2_AKK15969 -ADIVVLCVL FR--KHIPMN KYFLKQQLAW VPFLGLACWS LDMPFMKRYS RAYLLR----
DmLPLAT1_NP_572828 -LDVLGMFNI WH---VMNKC TVVAKRELFY AWPFGLAAWL AGLIFIDRVR G---------
DmLPLAT2_NP_609231 -VDLCVLAYL WP---VIGRA TVVSKKEVLY IPFFGIGAWL WGTLYIDRSR K---------
DmLPLAT3_NP_001036265 -VDSILVVAS G-------PP SIVAKRETAD IPLLGKIINY AQPIYVQRED P---------
ScLPLAT1_NP_010231 -LDIFMLGRI F-----PPGC TVTAKKSLKY VPFLGWFMAL SGTYFLDRSK R---------
MbLPLAT1_XP_001743302 FVDAVLVMST N-----RRPI KFLTAAKTMR RRFVGAFSRY LGCIPVERPQ DLATKGKGTI
EdLPLAT1_XP_001739767 -YDFLPIVIE APRCGRIGAM RFFMKEEISK IPLVGFGFYM MDSVYLKRNF Q---------
EdLPLAT2_XP_001734633 -VDFVPVCLV APKCGRIGSM RYFMKDDIKK IPFIGFGFYM MDSIYLKRNF Q---------
PfLPLAT1_XP_001348595 -VDPWVVNAT T----FPWPI KFVFKSSLLK VPIGGQALYF SGSIPLHFTK D---------
AtLPLAT1_NP_001030724 -VDWMYFWDL ALRKGQIGNI KYVLKSSLMK LPLFGWAFHL FEFIPVERRW E---------
AtLPLAT2_AAN65123 -VDWMYLWNI ALRKGCLGYI KYVLKSSLMK LPIFGWGFHV LEFIPVERKR E---------
AtLPLAT3_AAD55275 -VDWMYLWNI ALRKGCLGYI KYVLKSSLMK LPIFGWGFHV LEFIPVERKR E---------
AtLPLAT4_NP_567052 -IDWLVGWIL AQRSGCLGSA LAVMKKSSKF LPVIGWSMWF SEYLFLERNW A---------
AtLPLAT5_NP_175537 -IDWLIGWVM AQRVGCLGSS LAIMKKEAKY LPIIGWSMWF SDYIFLERSW A---------
AtLPLAT6_NP_565249 -LDILYHMSA S-------FP SFVAKRSVGK LPLVGLISKC LGCVYVQREA K---------
AtLPLAT7_Q8S8S2 -IEPIFYFYE L-------SP TIVASESHDS LPFVGTIIRA MQVIYVNRFS Q---------
TcLPLAT1_XP_817630 -ADPFLMVRV L----LPFDA AWIAKSGLFR VPFGGWCLSN ANDLKVCFKN K---------
HsLPLAT1_NP_006402 -LDLLGMMEV L-----PGRC VPIAKRELLW AGSAGLACWL AGVIFIDRKR T---------
HsLPLAT2_NP_006403 -LDMMGLMEV L-----PERC VQIAKRELLF LGPVGLIMYL GGVFFINRQR S---------
HsLPLAT3_NP_064517 -IDFLCGWTM CERFGVLGSS KVLAKKELLY VPLIGWTWYF LEIVFCKRKW E---------
HsLPLAT4_NP_064518 -IDFLCGWSL SERFGLLGGS KVLAKKELAY VPIIGWMWYF TEMVFCSRKW E---------
HsLPLAT5_NP_060831 -VDWIVADIL AIRQNALGHV RYVLKEGLKW LPLYGCYFAQ HGGIYVKRSA K---------
HsLPLAT6_NP_001002257 -MDWMFLWNC LMRYSYLRLE KICLKASLKG VPGFGWAMQA AAYIFIHRKW K---------
HsLPLAT7_NP_055688 -GDVCTLMMC LQDKGLVVAQ MMWLMDHIFK YTNFGIVSLV HGDFFIRQGR S---------
HsLPLAT8_NP_079106 -FDAIPVTM- T-------MS SIVMKAESRD IPIWGTLIQY IRPVFVSRSD Q---------
HsLPLAT9_NP_060309 -FDGIACVVA G-------LP SMVSRNENAQ VPLIGRLLRA VQPVLVSRVD P---------
HsLPLAT10_NP_705841 -FDPIVLLPC D-------LP KVVSRAENLS VPVIGALLRF NQAILVSRHD P---------
AGPAT motif: (1) fD
AGPAT motif: (2) rpvFvsR
301
EckLPLAT1_AIZ52813 ---------- ---------- ---------- ---------- TKAH------ ----------
EckLPLAT2_AKK15969 ---------- ---------- ---------- ---------- H--------- ----------
DmLPLAT1_NP_572828 ---------- ---------- ---------- ---------- EKAR------ ----------
DmLPLAT2_NP_609231 ---------- ---------- ---------- ---------- TDSI------ ----------
DmLPLAT3_NP_001036265 ---------- ---------- ---------- ---------- NS----RQ-- ----------
ScLPLAT1_NP_010231 ---------- ---------- ---------- ---------- QEAI------ ----------
MbLPLAT1_XP_001743302 SAATGATTVV GKDTQFTKDL TPGSGFAVDG HVFKVEKVIS DTEAEVRDGP SEGFKDVAFK
EdLPLAT1_XP_001739767 ---------- ---------- ---------- ---------- ---------- ----------
EdLPLAT2_XP_001734633 ---------- ---------- ---------- ---------- ---------- ----------
PfLPLAT1_XP_001348595 ---------- ---------- ---------- ---------- KGGWGVKQGE ----------
AtLPLAT1_NP_001030724 ---------- ---------- ---------- ---------- ---------- ----------
AtLPLAT2_AAN65123 ---------- ---------- ---------- ---------- ---------- ----------
AtLPLAT3_AAD55275 ---------- ---------- ---------- ---------- ---------- ----------
AtLPLAT4_NP_567052 ---------- ---------- ---------- ---------- ---------- ----------
AtLPLAT5_NP_175537 ---------- ---------- ---------- ---------- ---------- ----------
AtLPLAT6_NP_565249 ---------- ---------- ---------- ---------- SPDFKGVS-- ----------
AtLPLAT7_Q8S8S2 ---------- ---------- ---------- ---------- TS----RK-- ----------
TcLPLAT1_XP_817630 ---------- ---------- ---------- ---------- REGLETVKGT ----------
HsLPLAT1_NP_006402 ---------- ---------- ---------- ---------- GDAI------ ----------
HsLPLAT2_NP_006403 ---------- ---------- ---------- ---------- STAM------ ----------
HsLPLAT3_NP_064517 ---------- ---------- ---------- ---------- ---------- ----------
HsLPLAT4_NP_064518 ---------- ---------- ---------- ---------- ---------- ----------
HsLPLAT5_NP_060831 ---------- ---------- ---------- ---------- ---------- ----------
HsLPLAT6_NP_001002257 ---------- ---------- ---------- ---------- ---------- ----------
HsLPLAT7_NP_055688 ---------- ---------- ---------- ---------- ---------- ----------
HsLPLAT8_NP_079106 ---------- ---------- ---------- ---------- DS----RR-- ----------
HsLPLAT9_NP_060309 ---------- ---------- ---------- ---------- DS----RK-- ----------
HsLPLAT10_NP_705841 ---------- ---------- ---------- ---------- AS----RR-- ----------

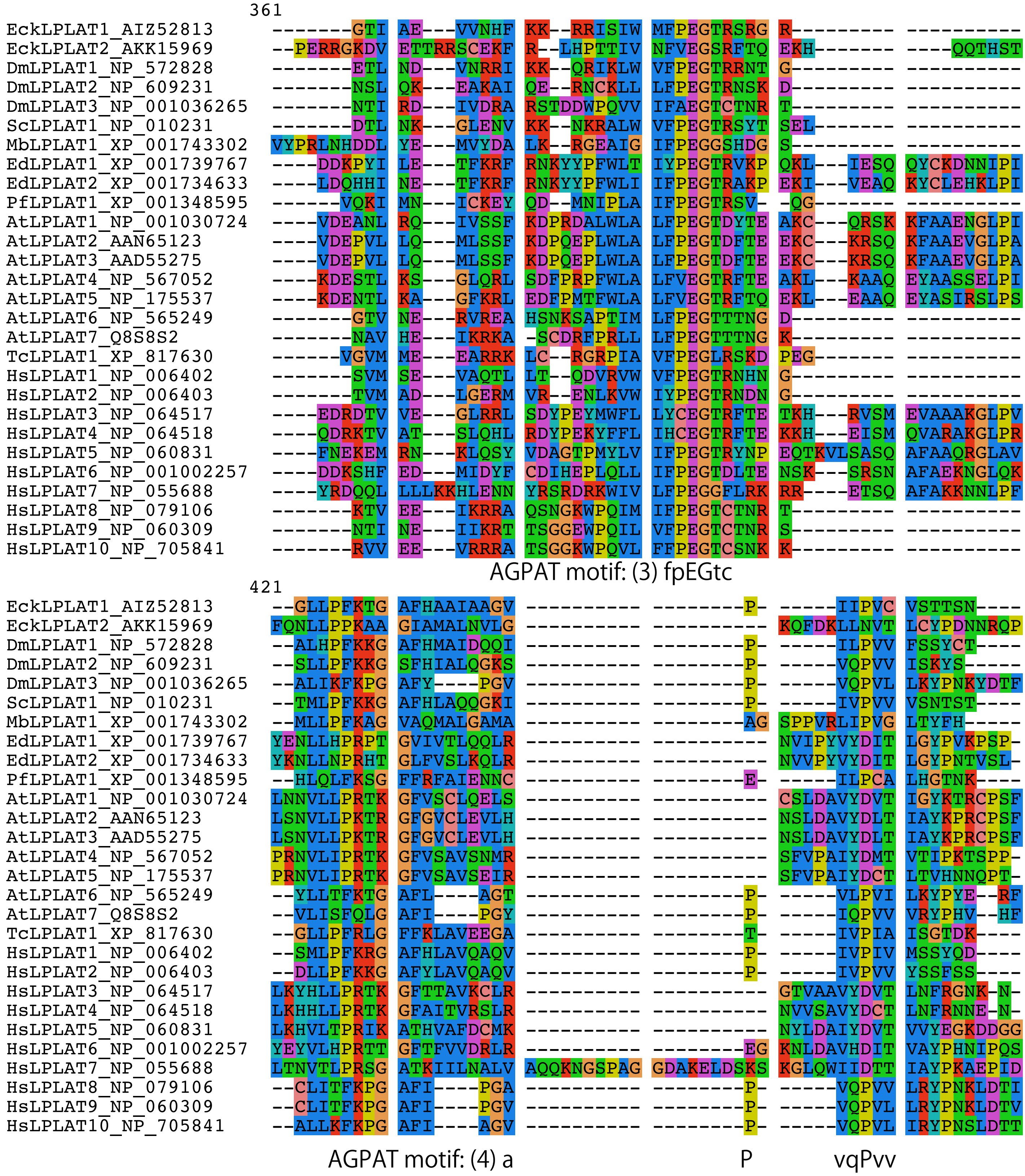
361
EckLPLAT1_AIZ52813 -------GTI AE---VVNHF KK--RRISIW MFPEGTRSRG R--------- ----------
EckLPLAT2_AKK15969 --PERRGKDV ETTRRSCEKF R--LHPTTIV NFVEGSRFTQ EKH------- ----QQTHST
DmLPLAT1_NP_572828 -------ETL ND---VNRRI KK--QRIKLW VFPEGTRRNT G--------- ----------
DmLPLAT2_NP_609231 -------NSL QK---EAKAI QE--RNCKLL LFPEGTRNSK D--------- ----------
DmLPLAT3_NP_001036265 -------NTI RD---IVDRA RSTDDWPQVV IFAEGTCTNR T--------- ----------
ScLPLAT1_NP_010231 -------DTL NK---GLENV KK--NKRALW VFPEGTRSYT SEL------- ----------
MbLPLAT1_XP_001743302 VYPRLNHDDL YE---MVYDA LK--RGEAIG IFPEGGSHDG S--------- ----------
EdLPLAT1_XP_001739767 ----DDKPYI LE---TFKRF RNKYYPFWLT IYPEGTRVKP QKL---IESQ QYCKDNNIPI
EdLPLAT2_XP_001734633 ----LDQHHI NE---TFKRF RNKYYPFWLI IFPEGTRAKP EKI---VEAQ KYCLEHKLPI
PfLPLAT1_XP_001348595 ------VQKI MN---ICKEY QD--MNIPLA IFPEGTRSV- -QG------- ----------
AtLPLAT1_NP_001030724 ----VDEANL RQ---IVSSF KDPRDALWLA LFPEGTDYTE AKC---QRSK KFAAENGLPI
AtLPLAT2_AAN65123 ----VDEPVL LQ---MLSSF KDPQEPLWLA LFPEGTDFTE EKC---KRSQ KFAAEVGLPA
AtLPLAT3_AAD55275 ----VDEPVL LQ---MLSSF KDPQEPLWLA LFPEGTDFTE EKC---KRSQ KFAAEVGLPA
AtLPLAT4_NP_567052 ----KDESTL KS---GLQRL SDFPRPFWLA LFVEGTRFTE AKL---KAAQ EYAASSELPI
AtLPLAT5_NP_175537 ----KDENTL KA---GFKRL EDFPMTFWLA LFVEGTRFTQ EKL---EAAQ EYASIRSLPS
AtLPLAT6_NP_565249 -------GTV NE---RVREA HSNKSAPTIM LFPEGTTTNG D--------- ----------
AtLPLAT7_Q8S8S2 -------NAV HE---IKRKA -SCDRFPRLL LFPEGTTTNG K--------- ----------
TcLPLAT1_XP_817630 ------VGVM ME---EARRK LC--RGRPIA VFPEGLRSKD PEG------- ----------
HsLPLAT1_NP_006402 -------SVM SE---VAQTL LT--QDVRVW VFPEGTRNHN G--------- ----------
HsLPLAT2_NP_006403 -------TVM AD---LGERM VR--ENLKVW IYPEGTRNDN G--------- ----------
HsLPLAT3_NP_064517 ----EDRDTV VE---GLRRL SDYPEYMWFL LYCEGTRFTE TKH---RVSM EVAAAKGLPV
HsLPLAT4_NP_064518 ----QDRKTV AT---SLQHL RDYPEKYFFL IHCEGTRFTE KKH---EISM QVARAKGLPR
HsLPLAT5_NP_060831 ----FNEKEM RN---KLQSY VDAGTPMYLV IFPEGTRYNP EQTKVLSASQ AFAAQRGLAV
HsLPLAT6_NP_001002257 ----DDKSHF ED---MIDYF CDIHEPLQLL IFPEGTDLTE NSK---SRSN AFAEKNGLQK
HsLPLAT7_NP_055688 ----YRDQQL LLLKKHLENN YRSRDRKWIV LFPEGGFLRK RR----ETSQ AFAKKNNLPF
HsLPLAT8_NP_079106 -------KTV EE---IKRRA QSNGKWPQIM IFPEGTCTNR T--------- ----------
HsLPLAT9_NP_060309 -------NTI NE---IIKRT TSGGEWPQIL VFPEGTCTNR S--------- ----------
HsLPLAT10_NP_705841 -------RVV EE---VRRRA TSGGKWPQVL FFPEGTCSNK K--------- ----------
AGPAT motif: (3) fpEGtc
421
EckLPLAT1_AIZ52813 --GLLPFKTG AFHAAIAAGV ---------- --------P- -----IIPVC VSTTSN----
EckLPLAT2_AKK15969 FQNLLPPKAA GIAMALNVLG ---------- ---------- KQFDKLLNVT LCYPDNNRQP
DmLPLAT1_NP_572828 --ALHPFKKG AFHMAIDQQI ---------- --------P- -----ILPVV FSSYCT----
DmLPLAT2_NP_609231 --SLLPFKKG SFHIALQGKS ---------- --------P- -----VQPVV ISKYS-----
DmLPLAT3_NP_001036265 --ALIKFKPG AFY----PGV ---------- --------P- -----VQPVL LKYPNKYDTF
ScLPLAT1_NP_010231 --TMLPFKKG AFHLAQQGKI ---------- --------P- -----IVPVV VSNTST----
MbLPLAT1_XP_001743302 --MLLPFKAG VAQMALGAMA ---------- --------AG SPPVRLIPVG LTYFH-----
EdLPLAT1_XP_001739767 YENLLHPRPT GVIVTLQQLR ---------- ---------- NVIPYVYDIT LGYPVKPSP-
EdLPLAT2_XP_001734633 YKNLLNPRHT GLFVSLKQLR ---------- ---------- NVVPYVYDIT LGYPNTVSL-
PfLPLAT1_XP_001348595 --HLQLFKSG FFRFAIENNC ---------- --------E- -----ILPCA LHGTNK----
AtLPLAT1_NP_001030724 LNNVLLPRTK GFVSCLQELS ---------- ---------- CSLDAVYDVT IGYKTRCPSF
AtLPLAT2_AAN65123 LSNVLLPKTR GFGVCLEVLH ---------- ---------- NSLDAVYDLT IAYKPRCPSF
AtLPLAT3_AAD55275 LSNVLLPKTR GFGVCLEVLH ---------- ---------- NSLDAVYDLT IAYKPRCPSF
AtLPLAT4_NP_567052 PRNVLIPRTK GFVSAVSNMR ---------- ---------- SFVPAIYDMT VTIPKTSPP-
AtLPLAT5_NP_175537 PRNVLIPRTK GFVSAVSEIR ---------- ---------- SFVPAIYDCT LTVHNNQPT-
AtLPLAT6_NP_565249 --YLLTFKTG AFL----AGT ---------- --------P- -----VLPVI LKYPYE--RF
AtLPLAT7_Q8S8S2 --VLISFQLG AFI----PGY ---------- --------P- -----IQPVV VRYPHV--HF
TcLPLAT1_XP_817630 --GLLPFRLG FFKLAVEEGA ---------- --------T- -----IVPIA ISGTDK----
HsLPLAT1_NP_006402 --SMLPFKRG AFHLAVQAQV ---------- --------P- -----IVPIV MSSYQD----
HsLPLAT2_NP_006403 --DLLPFKKG AFYLAVQAQV ---------- --------P- -----IVPVV YSSFSS----
HsLPLAT3_NP_064517 LKYHLLPRTK GFTTAVKCLR ---------- ---------- GTVAAVYDVT LNFRGNK-N-
HsLPLAT4_NP_064518 LKHHLLPRTK GFAITVRSLR ---------- ---------- NVVSAVYDCT LNFRNNE-N-
HsLPLAT5_NP_060831 LKHVLTPRIK ATHVAFDCMK ---------- ---------- NYLDAIYDVT VVYEGKDDGG
HsLPLAT6_NP_001002257 YEYVLHPRTT GFTFVVDRLR ---------- --------EG KNLDAVHDIT VAYPHNIPQS
HsLPLAT7_NP_055688 LTNVTLPRSG ATKIILNALV AQQKNGSPAG GDAKELDSKS KGLQWIIDTT IAYPKAEPID
HsLPLAT8_NP_079106 --CLITFKPG AFI----PGA ---------- --------P- -----VQPVV LRYPNKLDTI
HsLPLAT9_NP_060309 --CLITFKPG AFI----PGV ---------- --------P- -----VQPVL LRYPNKLDTV
HsLPLAT10_NP_705841 --ALLKFKPG AFI----AGV ---------- --------P- -----VQPVL IRYPNSLDTT
AGPAT motif: (4) a
P
vqPvv

```
                   481
EckLPLAT1_AIZ52813      ---------- -KIN--LN-- -------RLH NGLVIVEMLP PIDVSQ---- ---YGKDQVR
EckLPLAT2_AKK15969      ------F-FD -MLSGKLT-- ---------- RIVVHVDLQP IADELHGDYI NDKSFKRHFQ
DmLPLAT1_NP_572828      ---------- -FLNDKKK-- -------ILN SGRIVITTLP PVSTEG---- ---LTKDDID
DmLPLAT2_NP_609231      ---------- -FMDDEKK-- -------TFR PGHALIHILP EVSTEK---- ---YKREDVQ
DmLPLAT3_NP_001036265   --------TW -TWDGPGVLR LLWLTMTQF- YNRCEIEYLP VYTPSE---- ---DEVADAN
ScLPLAT1_NP_010231      ---------- -LVSPKYG-- -------VFN RGCMIVRILK PISTEN---- ---LTKDKIG
MbLPLAT1_XP_001743302   ---------- ------GH-- -------RF- RSRAMVEFGD PISIEQ---- ---DMVEAYR
EdLPLAT1_XP_001739767   --------SC CFFPGEGI-- ---------- TIHMNIHKIS IKDIPE---- ----DEESLK
EdLPLAT2_XP_001734633   --------AS CFCPGEGV-- ---------- NIHMNVNRID VKDIPE---- ----DENEFK
PfLPLAT1_XP_001348595   ---------- -VWPLKSS-- -------LFD KGTIYFSFGE PFKPTP---- ---GM--TYE
AtLPLAT1_NP_001030724   -------LDN -VYGIEPS-- ---------- EVHIHIRRIN LTQIPN---- ----QEKDIN
AtLPLAT2_AAN65123       -------MDN -VFGTDPS-- ---------- EVHIHVRRVL LKEIPA---- ----NEAESS
AtLPLAT3_AAD55275       -------MDN -VFGTDPS-- ---------- EVHIHVRRVL LKEIPA---- ----NEAESS
AtLPLAT4_NP_567052      -----PTMLR -LFKGQPS-- ---------- VVHVHIKCHS MKDLPE---- ----SDDAIA
AtLPLAT5_NP_175537      -----PTLLR -MFSGQSS-- ---------- EINLQMRRHK MSELPE---- ----TDDGIA
AtLPLAT6_NP_565249      --------SV -AWDTISGAR HILFLLCQV- VNHLEVIRLP VYYPSQ---- ---EEKDDPK
AtLPLAT7_Q8S8S2         --------DQ -SWGNISLLT LMFRMFTQF- HNFMEVEYLP VIYPSE---- ---KQKQNAV
TcLPLAT1_XP_817630      ---------- -CWPLGSV-- -------LMD SSTAYFSCGD PVDAST---- ---FR--TAE
HsLPLAT1_NP_006402      ---------- -FYCKKER-- -------RFT SGQCQVRVLP PVPTEG---- ---LTPDDVP
HsLPLAT2_NP_006403      ---------- -FYNTKKK-- -------FFT SGTVTVQVLE AIPTSG---- ---LTAADVP
HsLPLAT3_NP_064517      -----PSLLG -ILYGKKY-- ---------- EADMCVRRFP LEDIPL---- ----DEKEAA
HsLPLAT4_NP_064518      -----PTLLG -VLNGKKY-- ---------- HADLYVRRIP LEDIPE---- ----DDDECS
HsLPLAT5_NP_060831      QRRESPTMTE -FLCKECP-- ---------- KIHIHIDRID KKDVPE---- ----EQEHMR
HsLPLAT6_NP_001002257   -------EKH LLQGDFPR-- ---------- EIHFHVHRYP IDTLPT---- ----SKEDLQ
HsLPLAT7_NP_055688      ------IQTW ILGYRKPT-- ---------- VTHVHYRIFP IKDVPL---- ----ETDDLT
HsLPLAT8_NP_079106      --------TW -TWQGPGALE ILWLTLCQF- HNQVEIEFLP VYSPSE---- ---EEKRNPA
HsLPLAT9_NP_060309      --------TW -TWQGYTFIQ LCMLTFCQL- FTKVEVEFMP VQVPND---- ---EEKNDPV
HsLPLAT10_NP_705841     --------SW -AWRGPGVLK VLWLTASQP- CSIVDVEFLP VYHPSP---- ---EESRDPT

                   541
EckLPLAT1_AIZ52813      ELAAHCRSIM EQKIAEL--- ----DKEVAE REAAGK---- -----V---- ----------
EckLPLAT2_AKK15969      QWLNSLWQEK DRLLTSLMSS QR-------- ---------- ---------- ------QNK-
DmLPLAT1_NP_572828      VLMERVRSQM IETFKVT--- ----SAEALH RYKPIK---- -----KVGIP AASSASTSST
DmLPLAT2_NP_609231      LLIDECQSIM QTEYTKL--- ----SKEGQA LSSKKQ---- -----A---- ----------
DmLPLAT3_NP_001036265   LYANNVREVM AKALGVPTSD YSF------- -EDVIVMSRA R--------D MKIPFPGDIV
ScLPLAT1_NP_010231      EFAEKVRDQM VDTLKEI--- ----GYSPAI NDTTLP---- -----PQAIE YAALQHDKKV
MbLPLAT1_XP_001743302   NGGDAKRGAF DKLLKRVQEA VEAVTITAPD YETLKLLWAG RRLYKPENLE LSTEQTQQLT
EdLPLAT1_XP_001739767   RWLDDLWVEK DKLMSYFKEH KE-------- ---------- ---------- ----------
EdLPLAT2_XP_001734633   QWLSNIWKHK DELVDYYKEN GH-------- ---------- ---------- ----------
PfLPLAT1_XP_001348595   ELRDKTRMIM YEMIKEF--- --------PD YDPEVD---- -----TLAKE FSQARGH---
AtLPLAT1_NP_001030724   AWLMNTFQLK DQLLNDFYSN GH-------- ---------- ---------- ----------
AtLPLAT2_AAN65123       AWLMDSFKLK DKLLSDFNAQ GK-------- ---------- ---------- ----------
AtLPLAT3_AAD55275       AWLMDSFKLK DKLLSDFNAQ GK-------- ---------- ---------- ----------
AtLPLAT4_NP_567052      QWCRDQFVAK DALLDKHIAA DT-------- ---------- ---------- ----------
AtLPLAT5_NP_175537      QWCQDLFITK DAQLEKYFTK DV-------- ---------- ---------- ----------
AtLPLAT6_NP_565249      LYASNVRKLM ATEGNLILSE LGL------- -SDKRIYHAT L--------N GNLSQTRDFH
AtLPLAT7_Q8S8S2         RLSQKTSHAI ATSLNVVQTS HSF------- -ADLMLLNKA T--------E LKLENPSNYM
TcLPLAT1_XP_817630      ELRDHVWQVI TDLRESH--- --------PD RRGKLK---- -----SQ--- ----------
HsLPLAT1_NP_006402      ALADRVRHSM LTVFREI--- ----STDGRG GGDYLK---- -----KPGGG ----------
HsLPLAT2_NP_006403      ALVDTCHRAM RTTFLHI--- ----SKTPQE NGAT------ -------AGS ----------
HsLPLAT3_NP_064517      QWLHKLYQEK DALQEIYNQK GM-------- ---------- ---------- ----------
HsLPLAT4_NP_064518      AWLHKLYQEK DAFQEEYYRT GT-------- ---------- ---------- ----------
HsLPLAT5_NP_060831      RWLHERFEIK DKMLIEFYES PD-------- ---------- ---------- ------PERR
HsLPLAT6_NP_001002257   LWCHKRWEEK EERLRSFYQG EK-------- ---------- ---------- ----------
HsLPLAT7_NP_055688      TWLYQRFVEK EDLLSHFYET GA-------- ---------- ---------- ----------
HsLPLAT8_NP_079106      LYASNVRRVM AEALGVSVTD YTF------- -EDCQLALAE G--------Q LRLPADTCLL
HsLPLAT9_NP_060309      LFANKVRNLM AEALGIPVTD HTY------- -EDCRLMISA G--------Q LTLPMEAGLV
HsLPLAT10_NP_705841     LYANNVQRVM AQALGIPATE CEF------- -VGSLPVIVV G--------R LKVALEPQLW
```

```
                          601
EckLPLAT1_AIZ52813      ---------- ---------- ---------- ---------- ---------- ----------
EckLPLAT2_AKK15969      ---------- ---------- ---------- ---------- ---------- ----------
DmLPLAT1_NP_572828      TTTTLASPGT AVTPVADAAV AAAAAVASSS SAYGQPPGYE AGAAKASLLK TL--------
DmLPLAT2_NP_609231      ---------- ---------- ---------- ---------- ---------- ----------
DmLPLAT3_NP_001036265   E-IERTIEKL GLNESQR--- DAEL--CKGF -L---RLSNT DRLDIITFGE LLQVDLKNT-
ScLPLAT1_NP_010231      NKKIK----- -NEPVPSVSI SNDVNTH--- -----NE--- -GS----SVK KMH-------
MbLPLAT1_XP_001743302   IRFSEAYVQL KNEPEVQAL- RSDLA----- -VYNDAL-RA AGLHDHEVVQ TTVLQESEIF
EdLPLAT1_XP_001739767   --F----PGE PRKPQFKF-- -----T---- ---------- ---------- ---------W
EdLPLAT2_XP_001734633   --F----PGH EELYPLKF-- -----T---- ---------- ---------- ---------W
PfLPLAT1_XP_001348595   ---------- GF-------- ---------- ---------- ---------- ----------
AtLPLAT1_NP_001030724   --F----PNE GTEKEFNT-- KKYLIN---- ---------- ---------- --CLAVIAFT
AtLPLAT2_AAN65123       --F----PNQ RPEEELSV-- LKCIAT---- ---------- ---------- --FAGVISLT
AtLPLAT3_AAD55275       --F----PNQ RPEEELSV-- LKCIAT---- ---------- ---------- --FAGKQQQV
AtLPLAT4_NP_567052      --F----PGQ QEQNIGRP-- IKSLAV---- ---------- ---------- --VLS----W
AtLPLAT5_NP_175537      --F----SDL EVHQINRP-- IKPLIV---- ---------- ---------- --VII----W
AtLPLAT6_NP_565249      Q-KEE----- ---------- ---------- ---------- ---------- ----------
AtLPLAT7_Q8S8S2         VEMARVESLF HVSSLEA--- TRFLDTFVSM -----IPDSS GRVRLHDFLR GLKLKPCP--
TcLPLAT1_XP_817630      ---------- ---------- ---------- ---------- ---------- ----------
HsLPLAT1_NP_006402      ---------- G--------- ---------- ---------- ---------- ----------
HsLPLAT2_NP_006403      ---------- GVQPAQ---- ---------- ---------- ---------- ----------
HsLPLAT3_NP_064517      --F----PGE QFKPARRP-- WT-LLN---- ---------- ---------- --FLS----W
HsLPLAT4_NP_064518      --F----PET PMVPPRRP-- WT-LVN---- ---------- ---------- --WLF----W
HsLPLAT5_NP_060831      KRF----PGK SVNSKLSI-- KKTLPS---- ---------- ---------- --MLILSGLT
HsLPLAT6_NP_001002257   --N----FYF TGQSVI---- PPCKSE---- ---------- ---------- --LRVLVVKL
HsLPLAT7_NP_055688      --F----PPS KGHKEAVS-- -----R---- ---------- ---------- --EMTLSNLW
HsLPLAT8_NP_079106      E-FARLVRGL GLKPEKL--- EKDLDRYSER -A---RMKGG EKIGIAEFAA SLEVPVSD--
HsLPLAT9_NP_060309      E-FTKISRKL KLDWDGV--- RKHLDEYASI -A---SSSKG GRIGIEEFAK YLKLPVSD--
HsLPLAT10_NP_705841     E-LGKVLRKA GLSAGYV--- DAGA------ -----EPGRS RMISQEEFAR QLQLSDPQ--

                          661
EckLPLAT1_AIZ52813      ---------- ---------- ---------- ---------- ---------- ----------
EckLPLAT2_AKK15969      ---------- ---------- ---------- ---------- ---------- ----------
DmLPLAT1_NP_572828      ---------- ---------- ---------- ---------- ---------- ----------
DmLPLAT2_NP_609231      ---------- ---------- ---------- ---------- ---------- ----------
DmLPLAT3_NP_001036265   --DLH-KLFA LL-------- ----DHRRSG TVSLKSFLLC -SLFCKLKNS DLLTFLRALI
ScLPLAT1_NP_010231      ---------- ---------- ---------- ---------- ---------- ----------
MbLPLAT1_XP_001743302   SGVLQ-KGFL VL-------- -----IQTVV LIPFMVFGLP ILFACRVVST F-KARAAAAS
EdLPLAT1_XP_001739767   ADFT---GYM TPECFTKP-- ---------- ---------- ---------- ----------
EdLPLAT2_XP_001734633   ADFT---SYM SQECFSLP-- ---------- ---------- ---------- ----------
PfLPLAT1_XP_001348595   ---------- ---------- ---------- ---------- ---------- ----------
AtLPLAT1_NP_001030724   TICTHLTFFS SMIWF--RIY V-SLACVYL- -TSATHFNL- ---------- ----------
AtLPLAT2_AAN65123       VVFIYLTLYS H-SCF--KVY A-CLSGTYL- ---------- ---------- ----------
AtLPLAT3_AAD55275       T--------K P-SCQ--KVF L-LLNQSSD- -EKESKKAV- ---------- ----------
AtLPLAT4_NP_567052      ACVLTLGAIK FLHWA----- --QLFSSWKG ITISALGLGI -------ITL ----------
AtLPLAT5_NP_175537      LGFLVFGGFK LLQWL----- --SIVASWKI ILLFVFFLVI -------ATI ----------
AtLPLAT6_NP_565249      ---------- ---------- ---------- ---------- ---------- ----------
AtLPLAT7_Q8S8S2         --LSK-RIFE FI-------- ----DVEKVG SITFKQFLFA SGHVLTQPLF --KQTCELAF
TcLPLAT1_XP_817630      ---------- ---------- ---------- ---------- ---------- ----------
HsLPLAT1_NP_006402      ---------- ---------- ---------- ---------- ---------- ----------
HsLPLAT2_NP_006403      ---------- ---------- ---------- ---------- ---------- ----------
HsLPLAT3_NP_064517      ATILLSPLFS FVLGV----- --FASGSPLL ILTFLGFVGA -------ASF ----------
HsLPLAT4_NP_064518      ASLVLYPFFQ FLVSM----- --IRSGSSLT LASFILVFFV -------ASV ----------
HsLPLAT5_NP_060831      AGML-MTDAG RKLYVNTWIY GTLLGCLWVT IKA------- ---------- ----------
HsLPLAT6_NP_001002257   LSILYWTLFS PAMCL--LIY L-YSLVKWYF IITIVIFVL- ---------- ----------
HsLPLAT7_NP_055688      IFLIQ--SFA F--------- --LSGYMWYN IIQYFYHCLF ---------- ----------
HsLPLAT8_NP_079106      --LLE-DMFS LF-------- ----DESGSG EVDLRECVVA LSVVCRPART --LDTIQLAF
HsLPLAT9_NP_060309      --VLR-QLFA LF-------- ----DRNHDG SIDFREYVIG LAVLCNPSNT --EEIIQVAF
HsLPLAT10_NP_705841     --TVA-GAFG YF-------- ----QQDTKG LVDFRDVALA LAALDGGRSL --EELTRLAF
```

```
                       721
EckLPLAT1_AIZ52813     ---------- ---------- ---------- ---------- ---------- ----------
EckLPLAT2_AKK15969     ---------- ---------- ---------- ---------- ---------- ----------
DmLPLAT1_NP_572828     ---------- ---------- ---------- ---------- ---------- ----------
DmLPLAT2_NP_609231     ---------- ---------- ---------- ---------- ---------- ----------
DmLPLAT3_NP_001036265  HLYSESSQ-- ---QIDRESF V--------- RLMRHAGGKL NEQKAQALFY ALDTD----N
ScLPLAT1_NP_010231     ---------- ---------- ---------- ---------- ---------- ----------
MbLPLAT1_XP_001743302  S---AVKEHG RDVVATWKVL TALMIVPICL VLYSILLGQL IHPRLTGIS- ----------
EdLPLAT1_XP_001739767  ---------- ---------- ---------- ---------- ---------- ----------
EdLPLAT2_XP_001734633  ---------- ---------- ---------- ---------- ---------- ----------
PfLPLAT1_XP_001348595  ---------- ---------- ---------- ---------- ---------- ----------
AtLPLAT1_NP_001030724  ---------- ---------- ---------- -R-----S-V PLVETAKN-- ----------
AtLPLAT2_AAN65123      ---------- ---------- ---------- ---------- ----TFATYY KFQPS---PG
AtLPLAT3_AAD55275      ---------- ---------- ---------- -A-----Q-H PFTDTLDHLF QVQMS---SG
AtLPLAT4_NP_567052     ---------- ---------- ---------- -CMQILIR-S SQSERSTPAK VVPAK---PK
AtLPLAT5_NP_175537     ---------- ---------- ---------- -TMQILIQ-S SESQRSTPAK RPLQE---QL
AtLPLAT6_NP_565249     ---------- ---------- ---------- ---------- ---------- ----------
AtLPLAT7_Q8S8S2        SHCDADGD-- --GYITIQEL G--------- EALKNTIPNL NKDEIRGMYH LLDDD-QDQR
TcLPLAT1_XP_817630     ---------- ---------- ---------- ---------- ---------- ----------
HsLPLAT1_NP_006402     ---------- ---------- ---------- ---------- ---------- ----------
HsLPLAT2_NP_006403     ---------- ---------- ---------- ---------- ---------- ----------
HsLPLAT3_NP_064517     ---------- ---------- ---------- -GVRRLIG-V TEIEKGSSYG NQEFK---KK
HsLPLAT4_NP_064518     ---------- ---------- ---------- -GVRWMIG-V TEIDKGSAYG NSDSK---QK
HsLPLAT5_NP_060831     ---------- ---------- ---------- ---------- ---------- ----------
HsLPLAT6_NP_001002257  ---------- ---------- ---------- -QERIFGG-L EIIE-LACYR LLHKQ---PH
HsLPLAT7_NP_055688     ---------- ---------- ---------- ---------- ---------- ----------
HsLPLAT8_NP_079106     KMYGAQED-- --GSVGEGDL S--------- CILKTALG-V AELTVTDLFR AIDQE-EKGK
HsLPLAT9_NP_060309     KLFDVDED-- --GYITEEEF S--------- TILQASLG-V PDLDVSGLFK EIAQG-D--S
HsLPLAT10_NP_705841    ELFAEEQAEG PNRLLYKDGF S--------- TILHLLLG-S PHPAATALHA ELCQAGSSQG

                       781
EckLPLAT1_AIZ52813     ---------- ---------- ---------- ---------- ---------- ----------
EckLPLAT2_AKK15969     ---------- ---------- ---------- ---------- ---------- ----------
DmLPLAT1_NP_572828     ---------- ---------- ---------- ---------- ---------- ----------
DmLPLAT2_NP_609231     ---------- ---------- ---------- ---------- ---------- ----------
DmLPLAT3_NP_001036265  LGYVSFDSFV ELTEKQKSSY KFL-YHK--- ---------- ---------S EH--IR-RPK
ScLPLAT1_NP_010231     ---------- ---------- ---------- ---------- ---------- ----------
MbLPLAT1_XP_001743302  --WCLLPII- ----MLGSLH LWDHYTDLVK QLRGLLALMI SKQQTVELYY QRCKLKRRIR
EdLPLAT1_XP_001739767  ---------- ---------- ---------- ---------- ---------- ----------
EdLPLAT2_XP_001734633  ---------- ---------- ---------- ---------- ---------- ----------
PfLPLAT1_XP_001348595  ---------- ---------- ---------- ---------- ---------- ----------
AtLPLAT1_NP_001030724  -------SLK LVNK------ ---------- ---------- ---------- ----------
AtLPLAT2_AAN65123      --CFREDSCK VKNH------ ---------- ---------- ---------- ----------
AtLPLAT3_AAD55275      ---------- ---------- ---------- ---------- ---------- ----------
AtLPLAT4_NP_567052     --DNHHPESS SQTETEKEK- ---------- ---------- ---------- ----------
AtLPLAT5_NP_175537     --ISA----- ---------- ---------- ---------- ---------- ----------
AtLPLAT6_NP_565249     ---------- ---------- ---------- ---------- ---------- ----------
AtLPLAT7_Q8S8S2        ISQNDLLSCL RRNPLLIAIF A--------- ---------- ---------- ----------
TcLPLAT1_XP_817630     ---------- ---------- ---------- ---------- ---------- ----------
HsLPLAT1_NP_006402     ---------- ---------- ---------- ---------- ---------- ----------
HsLPLAT2_NP_006403     ---------- ---------- ---------- ---------- ---------- ----------
HsLPLAT3_NP_064517     --E------- ---------- ---------- ---------- ---------- ----------
HsLPLAT4_NP_064518     --LND----- ---------- ---------- ---------- ---------- ----------
HsLPLAT5_NP_060831     ---------- ---------- ---------- ---------- ---------- ----------
HsLPLAT6_NP_001002257  --LNSK---- -KNE------ ---------- ---------- ---------- ----------
HsLPLAT7_NP_055688     ---------- ---------- ---------- ---------- ---------- ----------
HsLPLAT8_NP_079106     ITFADFHRFA EMYPAFAEEY LYPDQTH--- ---------- ---------F ESCAETSP--
HsLPLAT9_NP_060309     ISYEEFKSFA LKHPEYAKIF T--TYLD--- ---------- ---------L QTCHVFSLPK
HsLPLAT10_NP_705841    LSLCQFQNFS LHDPLYGKLF S--TYLR--- ---------- ---------P PHTSRG----
```

```
                         841
EckLPLAT1_AIZ52813      ---------- ---------- ---------- ---------- -
EckLPLAT2_AKK15969      ---------- ---------- ---------- ---------- -
DmLPLAT1_NP_572828      ---------- ---------- ---------- ---------- -
DmLPLAT2_NP_609231      ---------- ---------- ---------- ---------- -
DmLPLAT3_NP_001036265   AVVTTAEN-- ---------- ---------- ---------- -
ScLPLAT1_NP_010231      ---------- ---------- ---------- ---------- -
MbLPLAT1_XP_001743302   AL---VNRAA DDMGIDRMFD EKDFMGEA-D HGLELDELLS M
EdLPLAT1_XP_001739767   ---------- ---------- ---------- ---------- -
EdLPLAT2_XP_001734633   ---------- ---------- ---------- ---------- -
PfLPLAT1_XP_001348595   ---------- ---------- ---------- ---------- -
AtLPLAT1_NP_001030724   ---------- ---------- ---------- ---------- -
AtLPLAT2_AAN65123       ---------- ---------- ---------- ---------- -
AtLPLAT3_AAD55275       ---------- ---------- ---------- ---------- -
AtLPLAT4_NP_567052      ---------- ---------- ---------- ---------- -
AtLPLAT5_NP_175537      ---------- ---------- ---------- ---------- -
AtLPLAT6_NP_565249      ---------- ---------- ---------- ---------- -
AtLPLAT7_Q8S8S2         ---------- PDLAPT---- ---------- ---------- -
TcLPLAT1_XP_817630      ---------- ---------- ---------- ---------- -
HsLPLAT1_NP_006402      ---------- ---------- ---------- ---------- -
HsLPLAT2_NP_006403      ---------- ---------- ---------- ---------- -
HsLPLAT3_NP_064517      ---------- ---------- ---------- ---------- -
HsLPLAT4_NP_064518      ---------- ---------- ---------- ---------- -
HsLPLAT5_NP_060831      ---------- ---------- ---------- ---------- -
HsLPLAT6_NP_001002257   ---------- ---------- ---------- ---------- -
HsLPLAT7_NP_055688      ---------- ---------- ---------- ---------- -
HsLPLAT8_NP_079106      --APIPNGFC ADFSPENS-- ---DAGRK-P VRKKLD---- -
HsLPLAT9_NP_060309      EVQTTPSTAS NKVSPEKH-- ---EESTS-D KKD--D---- -
HsLPLAT10_NP_705841     -TSQTPNASS PG-NPTAL-- ---ANGTVQA PKQKGD---- -
```

Supplement Figure 1A. Sequence alignment utilized in Figure 2A.
AGPAT motifs in HsLPLAT8 were shown.

Supplementary Fig. 1B

```
                       1
DmLPLAT4_NP_995637     ---------- ---------- ---------- -------MSI DDVIYVICLL GCIGAGSYVK
DmLPLAT5_NP_524157     ---------- -----MAEFE EDLPHNGLMD GIASGVGVPV EALRLLLTIL AGYPVAALYQ
DmLPLAT6_NP_610546     -MLEP----- --PKFIE--N DCYNGSRTFT WLADMVGLSV DLVNFLICQI SALFLASLFR
HsLPLAT11_NP_077274    ---------- ---------- ---------- -------MSP EEWTYLVVLL ISIPIGFLFK
HsLPLAT12_NP_005759    -MASSAEGDE GTVVALAGVL QSGFQELSLN KLATSLGASE QALRLIISIF LGYPFALFYR
HsLPLAT13_NP_620154    ---------- -----MA--T TSTTGSTLLQ PLSNAVQLPI DQVNFVVCQL FALLAAIWFR
HsLPLAT14_NP_001073949 MAAEP----- --QPSSL--S YRTTGSTYLH PLSELLGIPL DQVNFVVCQL VALFAAFWFR
ScLPLAT2_NP_014818     -MYNP----- --VD------ ------AVLT KIITNYGIDS FTLRYAICLL GSFPLNAILK
EdLPLAT3_XP_001734903  -MKEK----- --ISDSRSYK FKRMLDSFVT TSAQLIGFPE DMFRFLFAFI LEVPISIICR
EdLPLAT4_XP_001733896  ---------- ---------- -MSVIDFILE PISQIVHVPV YLLKYFSSFV LSLPMTYYCR
EdLPLAT5_XP_001735834  ---------- -------MFS NYSVVDEWVQ PISKGLNVPI DQVKYFTNAI IAAPICFGLR
EdLPLAT6_XP_001736261  ---------- ---------- ----MFTVIE SLAEKVHVPA DQIRYLLAFF IELPLCYILR
AtLPLAT8_NP_176493     ---------- ---------- ---MELLDMN SMAASIGVSV AVLRFLLCFV ATIPISFLWR
AtLPLAT9_NP_172724     ---------- ---------- ------MDMS SMAGSIGVSV AVLRFLLCFV ATIPVSFACR

                      61
DmLPLAT4_NP_995637     KI------AD EGQRKLVSTG LGVLVVVIVS GLHS-----L HCFVSLALGT AAVLLVH--P
DmLPLAT5_NP_524157     KFISV--IAD KTVHHMFFAG CGAGLCYFNY GLDT-----Y HSLIAILTTY FLVLLLRKKT
DmLPLAT6_NP_610546     SMLHPSKVSS K-LRHTFALS IGLAFGYFCF GQQA-----I HIAGLPAICY IVIRTQD--P
HsLPLAT11_NP_077274    K-------AG PGLKRWGAAA VGLGLTLFTC GPHT-----L HSLVT-ILGT WALIQAQ--P
HsLPLAT12_NP_005759    HYLFY--K-E TYLIHLFHTF TGLSIAYFNF GNQL-----Y HSLLCIVLQF LILRLMG-RT
HsLPLAT13_NP_620154    TYLHSSKTSS F-IRHVVATL LGLYLALFCF GWYA-----L HFLVQSGISY CIMIIIG--V
HsLPLAT14_NP_001073949 IYLRPGTTSS D-VRHAVATI FGIYFVIFCF GWYS-----V HLFVLVLMCY AIMVTAS--V
ScLPLAT2_NP_014818     RIP----EKR IGLKCCFIIS MSMF---YLF GVLNLVSGFR TLFISTMFTY LISRFYR--S
EdLPLAT3_XP_001734903  YI-----SMS TDMKYFIYGI IGIFISFFVY STMT-----Y CVLITMIPVY IIMKYMP--T
EdLPLAT4_XP_001733896  YL-----PNN PSLKHKVYGS IGITMSLIVF GPIT-----F SIIFLGLPIY YIAKTYP--T
EdLPLAT5_XP_001735834  YL-----PNN KNIKHIVYGT LGMIFSYLLF GQES-----I TVIFTTIPVY LIMKYNK--T
EdLPLAT6_XP_001736261  YL-----PSN PRLKHFVYST VGVIMLLFVF GINS-----L IIMIPALITY YFMKWKK--S
AtLPLAT8_NP_176493     FI-----PSR L-GKHIYSAA SGAFLSYLSF GFSS---N-L HFLVPMTIGY ASMAIYR--P
AtLPLAT9_NP_172724     IV-----PSR L-GKHLYAAA SGAFLSYLSF GFSS---N-L HFLVPMTIGY ASMAIYR--P

                     121
DmLPLAT4_NP_995637     SKGHLVTFAV MFGYLVFFRI FDFYFGIP-G H---TNMIQM ILTLKVSGIA FEKTAAWKRL
DmLPLAT5_NP_524157     QIFLAINFVF HMSYLLLGYF YT----SSND YDILWTMPHC ILVLRMIGYG FDITDGLKEE
DmLPLAT6_NP_610546     RIVQRAVLLV AMSYLLCVHL MR-QLYDYGS YALDITGPLM IITQKVTSLA FSIHDGFVRG
HsLPLAT11_NP_077274    CSCHALALAW TFSYLLFFRA LSL-LGLP-T PTPFTNAVQL LLTLKLVSLA SEVQDL----
HsLPLAT12_NP_005759    ITAVLTTFCF QMAYLLAGYY YT----ATGN YDIKWTMPHC VLTLKLIGLA VDYFDGGKDQ
HsLPLAT13_NP_620154    ENMHNYCFVF ALGYLTVCQV TRVYIFDYGQ YSADFSGPMM IITQKITSLA CEIHDGMFRK
HsLPLAT14_NP_001073949 SNIHRYSFFV AMGYLTICHI SRIYIFHYGI LTTDFSGPLM IVTQKITTLA FQVHDGLGRR
ScLPLAT2_NP_014818     KFMPHLNFMF VMGHLAINHI HAQFLNEQTQ TTVDITSSQM VLAMKLTSFA WSYYDGSCTS
EdLPLAT3_XP_001734903  RKGAYLCFGI SLGYLLTIHI KR-MIDNYLG YDLDFSSVQM VLTIKFTTFA FSVCNAYDKE
EdLPLAT4_XP_001733896  RIMAIITTTM LILHLACIHL YK-ILHTGMV YNLSFSTVHM MYVIKFSTFV WSVVDAHENN
EdLPLAT5_XP_001735834  QKAANICFII TFGYLLYRHI YL-YFNKYLI WTVEFTTTHM ILTLKLTGFA FSVANSNKKE
EdLPLAT6_XP_001736261  LHMALLMFSM NLIFLTLLQI YK-YINYYLQ WYLDITTLQM IVVIKLGNFV FNVAKGSNTN
AtLPLAT8_NP_176493     LS-GFITFFL GFAYLIGCHV FYMSGDAWKE GGIDSTGALM VLTLKVISCS INYNDGMLK-
AtLPLAT9_NP_172724     KC-GIITFFL GFAYLIGCHV FYMSGDAWKE GGIDSTGALM VLTLKVISCS MNYNDGMLK-

                     181
DmLPLAT4_NP_995637     QAHDEQKKND QRDVHQESPI EITDYDVELQ SLSAAEILHY SFNYIGVLTG PYYRYRTYRD
DmLPLAT5_NP_524157     S-------EL SKD------- --QKETALKK PPSLLELLAF SYFPSGFLVG PQFPFRRYKA
DmLPLAT6_NP_610546     DE------EL TKA------- --QQYHAIRK MPSALEYFSY VWHFQSILAG PLVFYKDYIE
HsLPLAT11_NP_077274    --HLAQRKEM ASGFSKGP-- ---TLGLLPD VPSLMETLSY SYCYVGIMTG PFFRYRTYLD
HsLPLAT12_NP_005759    N-------SL SSE------- --QQKYAIRG VPSLLEVAGF SYFYGAFLVG PQFSMNHYMK
HsLPLAT13_NP_620154    DE------EL TSS------- --QRDLAVRR MPSLLEYLSY NCNFMGILAG PLCSYKDYIT
HsLPLAT14_NP_001073949 AE------DL SAE------- --QHRLAIKV KPSFLEYLSY LLNFMSVIAG PCNNFKDYIA
ScLPLAT2_NP_014818     ESDF---KDL TEH------- --QKSRAVRG HPPLLKFLAY AFFYSTLLTG PSFDYADFDS
EdLPLAT3_XP_001734903  YK-C---S-- -EH------- --TEKHKIYN YPSILQFFGY TFFFPAFFSG PSMEYTDFVS
EdLPLAT4_XP_001733896  TK-L---C-- -EH------- --RKQMMIKT YPSLLEFFGY TFFFPGIFSG PSLEYIDYIK
EdLPLAT5_XP_001735834  LT-K---YLE ---------- ----IHKINK YPNIIEYYGF IFFYPGLFSG PTLEYNDYMS
EdLPLAT6_XP_001736261  II-R---QTD YNK------- --MNNIPRDQ FPSLLEFIGY FYFFPSIFSG PCIEYQTYKK
AtLPLAT8_NP_176493     EE------GL REA------- --QKKNRLIQ MPSLIEYFGY CLCCGSHFAG PVFEMKDYLE
AtLPLAT9_NP_172724     EE------GL REA------- --QKKNRLIQ MPSLIEYFGY CLCCGSHFAG PVYEMKDYLE
```

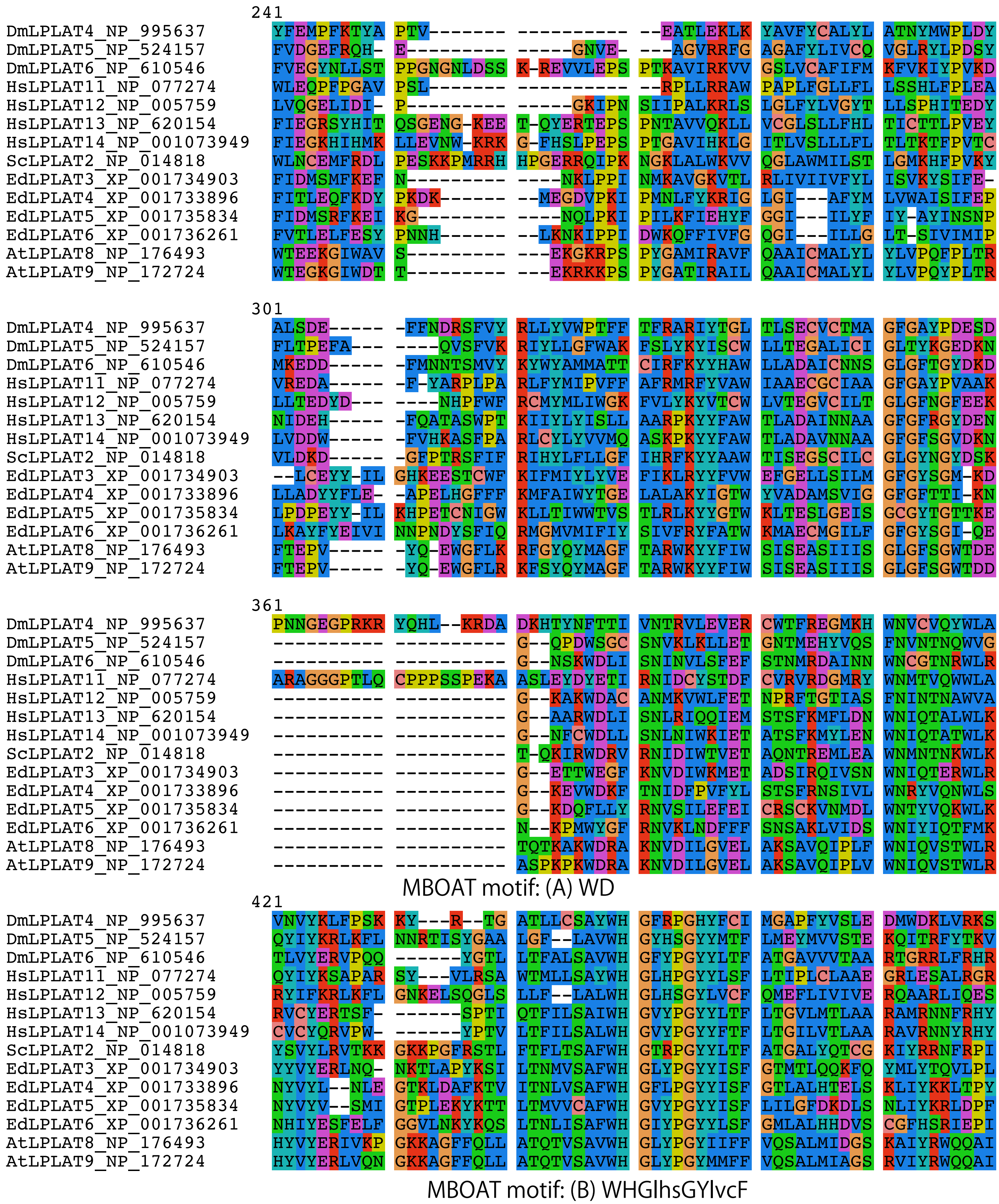
241
DmLPLAT4_NP_995637 YFEMPFKTYA PTV------- ---------- --EATLEKLK YAVFYCALYL ATNYMWPLDY
DmLPLAT5_NP_524157 FVDGEFRQH- E--------- -----GNVE- ---AGVRRFG AGAFYLIVCQ VGLRYLPDSY
DmLPLAT6_NP_610546 FVEGYNLLST PPGNGNLDSS K-REVVLEPS PTKAVIRKVV GSLVCAFIFM KFVKIYPVKD
HsLPLAT11_NP_077274 WLEQPFPGAV PSL------- ---------- --RPLLRRAW PAPLFGLLFL LSSHLFPLEA
HsLPLAT12_NP_005759 LVQGELIDI- P--------- -----GKIPN SIIPALKRLS LGLFYLVGYT LLSPHITEDY
HsLPLAT13_NP_620154 FIEGRSYHIT QSGENG-KEE T-QYERTEPS PNTAVVQKLL VCGLSLLFHL TICTTLPVEY
HsLPLAT14_NP_001073949 FIEGKHIHMK LLEVNW-KRK G-FHSLPEPS PTGAVIHKLG ITLVSLLLFL TLTKTFPVTC
ScLPLAT2_NP_014818 WLNCEMFRDL PESKKPMRRH HPGERRQIPK NGKLALWKVV QGLAWMILST LGMKHFPVKY
EdLPLAT3_XP_001734903 FIDMSMFKEF N--------- ----NKLPPI NMKAVGKVTL RLIVIIVFYL ISVKYSIFE-
EdLPLAT4_XP_001733896 FITLEQFKDY PKDK------ --MEGDVPKI PMNLFYKRIG LGI---AFYM LVWAISIFEP
EdLPLAT5_XP_001735834 FIDMSRFKEI KG-------- ----NQLPKI PILKFIEHYF GGI---ILYF IY-AYINSNP
EdLPLAT6_XP_001736261 FVTLELFESY PNNH------ --LKNKIPPI DWKQFFIVFG QGI---ILLG LT-SIVIMIP
AtLPLAT8_NP_176493 WTEEKGIWAV S--------- ---EKGKRPS PYGAMIRAVF QAAICMALYL YLVPQFPLTR
AtLPLAT9_NP_172724 WTEGKGIWDT T--------- ---EKRKKPS PYGATIRAIL QAAICMALYL YLVPQYPLTR
301
DmLPLAT4_NP_995637 ALSDE----- -FFNDRSFVY RLLYVWPTFF TFRARIYTGL TLSECVCTMA GFGAYPDESD
DmLPLAT5_NP_524157 FLTPEFA--- ----QVSFVK RIYLLGFWAK FSLYKYISCW LLTEGALICI GLTYKGEDKN
DmLPLAT6_NP_610546 MKEDD----- -FMNNTSMVY KYWYAMMATT CIRFKYYHAW LLADAICNNS GLGFTGYDKD
HsLPLAT11_NP_077274 VREDA----- -F-YARPLPA RLFYMIPVFF AFRMRFYVAW IAAECGCIAA GFGAYPVAAK
HsLPLAT12_NP_005759 LLTEDYD--- ----NHPFWF RCMYMLIWGK FVLYKYVTCW LVTEGVCILT GLGFNGFEEK
HsLPLAT13_NP_620154 NIDEH----- -FQATASWPT KIIYLYISLL AARPKYYFAW TLADAINNAA GFGFRGYDEN
HsLPLAT14_NP_001073949 LVDDW----- -FVHKASFPA RLCYLYVVMQ ASKPKYYFAW TLADAVNNAA GFGFSGVDKN
ScLPLAT2_NP_014818 VLDKD----- -GFPTRSFIF RIHYLFLLGF IHRFKYYAAW TISEGSCILC GLGYNGYDSK
EdLPLAT3_XP_001734903 --LCEYY-IL GHKEESTCWF KIFMIYLYVE FIKLRYYFVW EFGELLSILM GFGYSGM-KD
EdLPLAT4_XP_001733896 LLADYYFLE- -APELHGFFF KMFAIWYTGE LALAKYIGTW YVADAMSVIG GFGFTTI-KN
EdLPLAT5_XP_001735834 LPDPEYY-IL KHPETCNIGW KLLTIWWTVS TLRLKYYGTW KLTESLGEIS GCGYTGTTKE
EdLPLAT6_XP_001736261 LKAYFYEIVI NNPNDYSFIQ RMGMVMIFIY SIVFRYFATW KMAECMGILF GFGYSGI-QE
AtLPLAT8_NP_176493 FTEPV----- -YQ-EWGFLK RFGYQYMAGF TARWKYYFIW SISEASIIIS GLGFSGWTDE
AtLPLAT9_NP_172724 FTEPV----- -YQ-EWGFLR KFSYQYMAGF TARWKYYFIW SISEASIIIS GLGFSGWTDD
361
DmLPLAT4_NP_995637 PNNGEGPRKR YQHL--KRDA DKHTYNFTTI VNTRVLEVER CWTFREGMKH WNVCVQYWLA
DmLPLAT5_NP_524157 ---------- ---------- G--QPDWSGC SNVKLKLLET GNTMEHYVQS FNVNTNQWVG
DmLPLAT6_NP_610546 ---------- ---------- G--NSKWDLI SNINVLSFEF STNMRDAINN WNCGTNRWLR
HsLPLAT11_NP_077274 ARAGGGPTLQ CPPPSSPEKA ASLEYDYETI RNIDCYSTDF CVRVRDGMRY WNMTVQWWLA
HsLPLAT12_NP_005759 ---------- ---------- G--KAKWDAC ANMKVWLFET NPRFTGTIAS FNINTNAWVA
HsLPLAT13_NP_620154 ---------- ---------- G--AARWDLI SNLRIQQIEM STSFKMFLDN WNIQTALWLK
HsLPLAT14_NP_001073949 ---------- ---------- G--NFCWDLL SNLNIWKIET ATSFKMYLEN WNIQTATWLK
ScLPLAT2_NP_014818 ---------- ---------- T-QKIRWDRV RNIDIWTVET AQNTREMLEA WNMNTNKWLK
EdLPLAT3_XP_001734903 ---------- ---------- G--ETTWEGF KNVDIWKMET ADSIRQIVSN WNIQTERWLR
EdLPLAT4_XP_001733896 ---------- ---------- G--KEVWDKF TNIDFPVFYL STSFRNSIVL WNRYVQNWLS
EdLPLAT5_XP_001735834 ---------- ---------- G--KDQFLLY RNVSILEFEI CRSCKVNMDL WNTYVQKWLK
EdLPLAT6_XP_001736261 ---------- ---------- N--KPMWYGF RNVKLNDFFF SNSAKLVIDS WNIYIQTFMK
AtLPLAT8_NP_176493 ---------- ---------- TQTKAKWDRA KNVDILGVEL AKSAVQIPLF WNIQVSTWLR
AtLPLAT9_NP_172724 ---------- ---------- ASPKPKWDRA KNVDILGVEL AKSAVQIPLV WNIQVSTWLR
MBOAT motif: (A) WD
421
DmLPLAT4_NP_995637 VNVYKLFPSK KY---R--TG ATLLCSAYWH GFRPGHYFCI MGAPFYVSLE DMWDKLVRKS
DmLPLAT5_NP_524157 QYIYKRLKFL NNRTISYGAA LGF--LAVWH GYHSGYYMTF LMEYMVVSTE KQITRFYTKV
DmLPLAT6_NP_610546 TLVYERVPQQ -------YGTL LTFALSAVWH GFYPGYYLTF ATGAVVVTAA RTGRRLFRHR
HsLPLAT11_NP_077274 QYIYKSAPAR SY---VLRSA WTMLLSAYWH GLHPGYYLSF LTIPLCLAAE GRLESALRGR
HsLPLAT12_NP_005759 RYIFKRLKFL GNKELSQGLS LLF--LALWH GLHSGYLVCF QMEFLIVIVE RQAARLIQES
HsLPLAT13_NP_620154 RVCYERTSF- -------SPTI QTFILSAIWH GVYPGYYLTF LTGVLMTLAA RAMRNNFRHY
HsLPLAT14_NP_001073949 CVCYQRVPW- -------YPTV LTFILSALWH GVYPGYYFTF LTGILVTLAA RAVRNNYRHY
ScLPLAT2_NP_014818 YSVYLRVTKK GKKPGFRSTL FTFLTSAFWH GTRPGYYLTF ATGALYQTCG KIYRRNFRPI
EdLPLAT3_XP_001734903 YYVYERLNQ- NKTLAPYKSI LTNMVSAFWH GLYPGYYISF GTMTLQQKFQ YMLYTQVLPL
EdLPLAT4_XP_001733896 NYVYL--NLE GTKLDAFKTV ITNLVSAFWH GFLPGYYISF GTLALHTELS KLIYKKLTPY
EdLPLAT5_XP_001735834 NYVYV--SMI GTPLEKYKTT LTMVVCAFWH GVYPGYYISF LILGFDKDLS NLIYKRLDPF
EdLPLAT6_XP_001736261 NHIYESFELF GGVLNKYKQS LTNLISAFWH GIYPGYYLSF GMLALHHDVS CGFHSRIEPI
AtLPLAT8_NP_176493 HYVYERIVKP GKKAGFFQLL ATQTVSAVWH GLYPGYIIFF VQSALMIDGS KAIYRWQQAI
AtLPLAT9_NP_172724 HYVYERLVQN GKKAGFFQLL ATQTVSAVWH GLYPGYMMFF VQSALMIAGS RVIYRWQQAI
MBOAT motif: (B) WHGlhsGYlvcF

```
                       481
DmLPLAT4_NP_995637     ATGTS----- -----RRVID VIFWIFKWFA FSYLGEAFLL SSFGNIWRFY SSVYHIGYIS
DmLPLAT5_NP_524157     VLPQWGHILN NSDIYKLLYF ITLKSYNVVY MGWCLTAFVF LKYERWIVVY GAVSYYGFTF
DmLPLAT6_NP_610546     FQSTQ----- ---VTRMFYD ILTCLITRVV LGYATFPFVL LEFMGSIKLY LRFYLCLHII
HsLPLAT11_NP_077274    LSPGG----- -----QKAWD WVHWFLKMRA YDYMCMGFVL LSLADTLRYW ASIYFCI--H
HsLPLAT12_NP_005759    PT--LS-KLA AITVLQPFYY LVQQTIHWLF MGYSMTAFCL FTWDKWLKVY KSIYFLGHIF
HsLPLAT13_NP_620154    FIEPS----- ---QLKLFYD VITWIVTQVA ISYTVVPFVL LSIKPSLTFY SSWYYCLHIL
HsLPLAT14_NP_001073949 FLSSR----- ---ALKAVYD AGTWAVTQLA VSYTVAPFVM LAVEPTISLY KSMYFYLHII
ScLPLAT2_NP_014818     FLREDG---V TPLPSKKIYD LVGIYAIKLA FGYMVQPFII LDLKPSLMVW GSVYFYVHII
EdLPLAT3_XP_001734903  LRNKYG---E KSIP-IYLYH TFNYLYTPFA QMYCFVSFVL LSFSATFTVY LHTYFIPHII
EdLPLAT4_XP_001733896  IIYLTK---N NKKM-MTVWN VFLIIFTNLS IKYNFCPFFM MTFKASWEMF KQTYFCFHLI
EdLPLAT5_XP_001735834  MKIKFG---E KSIQ-WKLYD ILLRVFNHWH LNYAVYPFMR FELIPSLIVL YRTYFLGYLI
EdLPLAT6_XP_001736261  IIKKYG---K DSLI-YYSYW VLTFIYARIS ISYSFIPFVI YTFIDSWKFF VNTYFCVDVI
AtLPLAT8_NP_176493     PPKMA----- ---MLRNVLV LINFLYTVVV LNYSSVGFMV LSLHETLVAF KSVYYIGTVI
AtLPLAT9_NP_172724     SPKMA----- ---MLRNIMV FINFLYTVLV LNYSAVGFMV LSLHETLTAY GSVYYIGTII
                                       MBOAT motifs: (C) YsmtaF          (D) Y  ksiYFlgH

                       541
DmLPLAT4_NP_995637     WAAMTALGFY LTSQRKA-AE RRKKRAAEKA AG-------- ---------- -----GDGIA
DmLPLAT5_NP_524157     LVLWAAFYHT FNHFFRSSSR KLAGE--DQK LQDSNTDKL- ---------- ---------VE
DmLPLAT6_NP_610546     SLVTIFI--- LPKFIRG-ER RLRTSNGNGN VRLSGSGNTK DAVTTSVEST AALTAGNDLN
HsLPLAT11_NP_077274    FLALAALGLG LALGGGS-PS RRKAASQPTS LA-------- ---------- -----PEKLR
HsLPLAT12_NP_005759    FLSLLFILPY IHKA------ -MVPR--KEK LKKME----- ---------- ----------
HsLPLAT13_NP_620154    GILVLLL--- LPVKKTQ-RR KNTHE----N IQLSQSKKFD EGEN------ --------SLG
HsLPLAT14_NP_001073949 SLLIILF--- LPMKPQA-HT QRRPQ----T LNSINKRKTD ---------- ----------
ScLPLAT2_NP_014818     VAFSFFLFR- GPYAKQV-TE FFKSK-QPKE IFIRKQKKLE KDISASSPN- ----LGGILK
EdLPLAT3_XP_001734903  ALTGWMYLSY FPVYKKPRQE TVETK--PKE VTTGSPATKE ---------- ----------
EdLPLAT4_XP_001733896  ALILFIWLTV APPKIPK-EE KNKKE----- ---------- ---------- ----------
EdLPLAT5_XP_001735834  PLFLYLWLSY YPPHLSY-QN TMNEQ--LKQ QKDSTLLKKE D--------- ----------
EdLPLAT6_XP_001736261  GLLALIILYV WFPKQQK-RD RID------- ---------- ---------- ----------
AtLPLAT8_NP_176493     PIAVLLLSYL VPVKPVR-PK TRKEE----- ---------- ---------- ----------
AtLPLAT9_NP_172724     PVGLILLSYV VPAKPSR-PK PRKEE----- ---------- ---------- ----------

                       601
DmLPLAT4_NP_995637     AAIEKEKAQ- ---------- ---------- ---------- ---------- ----------
DmLPLAT5_NP_524157     EKKPEDKKSE ---------- ---------- ---------- ---------- ----------
DmLPLAT6_NP_610546     EDKEEDKHAQ CKVHTPTQQQ PAAGPHKTTV EQPTEQPNNV NLRSRPQQQQ PHL-EKKAMP
HsLPLAT11_NP_077274    ----EE---- ---------- ---------- ---------- ---------- ----------
HsLPLAT12_NP_005759    ---------- ---------- ---------- ---------- ---------- ----------
HsLPLAT13_NP_620154    QNSFSTTNNV CNQN-----Q EIASRHS-SL KQ-------- ---------- ----------
HsLPLAT14_NP_001073949 ---------- ---------- ---------- ---------- ---------- ----------
ScLPLAT2_NP_014818     AKIEHEKG-- ---------- ---------- --KTAEEEEM NLGIPPIELE KWDNAKEDWE
EdLPLAT3_XP_001734903  ---------- ---------- ---------- ---------- ---------- ----------
EdLPLAT4_XP_001733896  ---------- ---------- ---------- ---------- ---------- ----------
EdLPLAT5_XP_001735834  ---------- ---------- ---------- ---------- ---------- ----------
EdLPLAT6_XP_001736261  ---------- ---------- ---------- ---------- ---------- ----------
AtLPLAT8_NP_176493     ---------- ---------- ---------- ---------- ---------- ----------
AtLPLAT9_NP_172724     ---------- ---------- ---------- ---------- ---------- ----------

                       661
DmLPLAT4_NP_995637     ---------- ---------- ---------- ---------- ---------- ----------
DmLPLAT5_NP_524157     ---------- ---------- ---------- ---------- ---------- ----------
DmLPLAT6_NP_610546     PTCARDAVSV PHDQCEMDQL SSKLKEKIEA ETKNIEEFID KTVTETVSGI VEFKNDLMRD
HsLPLAT11_NP_077274    ---------- ---------- ---------- ---------- ---------- ----------
HsLPLAT12_NP_005759    ---------- ---------- ---------- ---------- ---------- ----------
HsLPLAT13_NP_620154    ---------- ---------- ---------- ---------- ---------- ----------
HsLPLAT14_NP_001073949 ---------- ---------- ---------- ---------- ---------- ----------
ScLPLAT2_NP_014818     ---------- --DFCKDY-K EWRNKNGLEI EEENLSKAFE ---------- -RFKQEF---
EdLPLAT3_XP_001734903  ---------- ---------- ---------- ---------- ---------- ----------
EdLPLAT4_XP_001733896  ---------- ---------- ---------- ---------- ---------- ----------
EdLPLAT5_XP_001735834  ---------- ---------- ---------- ---------- ---------- ----------
EdLPLAT6_XP_001736261  ---------- ---------- ---------- ---------- ---------- ----------
AtLPLAT8_NP_176493     ---------- ---------- ---------- ---------- ---------- ----------
AtLPLAT9_NP_172724     ---------- ---------- ---------- ---------- ---------- ----------
```

```
                          721
DmLPLAT4_NP_995637       ---------- ---------- ---------- ---------- ---------- ----------
DmLPLAT5_NP_524157       ---------- ---------- ---------- ---------- ---------- ----------
DmLPLAT6_NP_610546       IEFPKLKLPG SNGAISLDSS NGGGLRKRNI SSVHDNGTDP GHATADLHPP LEENGAAFLK
HsLPLAT11_NP_077274      ---------- ---------- ---------- ---------- ---------- ----------
HsLPLAT12_NP_005759      ---------- ---------- ---------- ---------- ---------- ----------
HsLPLAT13_NP_620154      ---------- ---------- ---------- ---------- ---------- ----------
HsLPLAT14_NP_001073949   ---------- ---------- ---------- ---------- ---------- ----------
ScLPLAT2_NP_014818       ---------- S------NAA SGSGERVRKM SF---SGYSP KPISK----- ---KEE----
EdLPLAT3_XP_001734903    ---------- ---------- ---------- ---------- ---------- ----------
EdLPLAT4_XP_001733896    ---------- ---------- ---------- ---------- ---------- ----------
EdLPLAT5_XP_001735834    ---------- ---------- ---------- ---------- ---------- ----------
EdLPLAT6_XP_001736261    ---------- ---------- ---------- ---------- ---------- ----------
AtLPLAT8_NP_176493       ---------- ---------- ---------- ---------- ---------- ----------
AtLPLAT9_NP_172724       ---------- ---------- ---------- ---------- ---------- ----------

                          781
DmLPLAT4_NP_995637       ---------- ---------- ----
DmLPLAT5_NP_524157       ---------- ---------- ----
DmLPLAT6_NP_610546       KEIEVINAVV QQAVPAVLSN GHAK
HsLPLAT11_NP_077274      ---------- ---------- ----
HsLPLAT12_NP_005759      ---------- ---------- ----
HsLPLAT13_NP_620154      ---------- ---------- ----
HsLPLAT14_NP_001073949   ---------- ---------- ----
ScLPLAT2_NP_014818       ---------- ---------- ----
EdLPLAT3_XP_001734903    ---------- ---------- ----
EdLPLAT4_XP_001733896    ---------- ---------- ----
EdLPLAT5_XP_001735834    ---------- ---------- ----
EdLPLAT6_XP_001736261    ---------- ---------- ----
AtLPLAT8_NP_176493       ---------- ---------- ----
AtLPLAT9_NP_172724       ---------- ---------- ----
```

Supplement Figure 1B. Sequence alignment utilized in Figure 2B. MBOAT motifs in HsLPLAT12 were shown.

Supplementary Fig. 2A

```
                            1
AqLPLAT1_XP_019853566       ---------- ---------- ---------- ---------- ---------- ----------
AqLPLAT2_XP_011403842       ---------- ---------- ---------- ---------- ---------- ----------
NvLPLAT1_XP_048587027       ---------- ---------- ---------- ---------- ---------- ----------
MgLPLAT1_XP_011449105       ---------- ---------- ---------- ---------- ---------- ----------
MgLPLAT2_XP_011416667       ---------- ---------- ---------- ---------- ---------- ----------
MgLPLAT3_XP_011422898       MDGEFLKLTL YLLCSTVCCV IGLTVVLAAI GKSLGIRKLY TSVLL----S IF----EFGK
MgLPLAT4_XP_011457228       ---------- ---------- ---------- ---------- ---------- ----------
DmLPLAT1_NP_572828          ---------- ---------- ---------- ---------- ---------- ----------
DmLPLAT2_NP_609231          ---------- ---------- ---------- ---------- ---------- ----------
DmLPLAT3_NP_001036265       ---------- ---------- ---------- ---------- ---------- ----------
CeLPLAT1_NP_510606          ---------- ---------- ---------- ---------- ---------- ----------
CeLPLAT2_NP_001256175       ---------- ---------- ---------- ---------- ---------- ----------
CeLPLAT3_NP_001348648       ---------- ---------- ---------- ---------- ---------- ----------
CeLPLAT4_NP_504644          ---------- ---------- ---------- ---------- ---------- ----------
CeLPLAT5_Q20800             ---------- ---------- ---------- ---------- ---------- ----------
CeLPLAT6_NP_491479          ---------- ---------- ---------- ---------- ---------- ----------
CeLPLAT7_NP_509260          ---------- ---------- ---------- ---------- ---------- ----------
CeLPLAT8_NP_502370          ---------- ---------- ---------- ---------- ---------- ----------
CeLPLAT9_NP_001371003       ---------- ---------- ---------- ---------- ---------- ----------
CtLPLAT1_ELT98524           ---------- ---------- ---------- ---------- ---------- ----------
CtLPLAT2_ELU12181           ---------- ---------- ---------- ---------- ---------- ----------
CtLPLAT3_ELU10392           ---------- ---------- ---------- ---------- ---------- ----------
DrLPLAT2_NP_001071200       ---------- ---------- ---------- ---------- ---------- ----------
DrLPLAT3_NP_998590          ---------- ---------- ---------- ---------- ---------- ----------
DrLPLAT4_NP_998157          ---------- ---------- ---------- ---------- ---------- ----------
DrLPLAT5_NP_001070213       ---------- ---------- ---------- ---------- ---------- ----------
DrLPLAT6_NP_998435          ---------- ---------- ---------- ---------- ---------- ----------
DrLPLAT7_NP_001025129       ---------- ---------- ---------- ---------- ---------- ----------
DrLPLAT8_NP_001037806       ---------- ---------- ---------- ---------- ---------- ----------
DrLPLAT9_NP_001018492       ---------- ---------- ---------- ---------- ---------- ----------
DrLPLAT10_NP_991122         ---------- ---------- ---------- ---------- ---------- ----------
XtLPLAT1_XP_012823272       ---------- ---------- ---------- --------ML TTPLV----S ----------
XtLPLAT2_NP_001123741       ---------- ---------- ---------- ---------- ---------- ----------
XtLPLAT3_NP_001008119       ---------- ---------- ---------- ---------- ---------- ----------
XtLPLAT4_NP_001011265       ---------- ---------- ---------- ---------- ---------- ----------
XtLPLAT5_NP_001015964       ---------- ---------- ---------- ---------- ---------- ----------
XtLPLAT6_NP_001135517       ---------- ---------- ---------- ---------- ---------- ----------
XtLPLAT7_XP_017949604       ---------- ---------- ---------- ---------- MRSLPS--PS ----ANSAGG
XtLPLAT8_XP_002933165       ---------- ---------- ---------- ---------- ---------- ----------
XtLPLAT9_XP_012815924       ---------- ---------- ---------- ---------- ---------- ----------
XtLPLAT10_NP_001037863      ---------- ---------- ---------- ---------- ---------- ----------
AcLPLAT1_XP_062829728       ---------- ---------- ---------- ---------- ---------- ----------
AcLPLAT2_XP_062816491       ---------- ---------- ---------- ---------- ---------- ----------
AcLPLAT3_XP_008105662       ---------- ---------- ---------- ---------- ---------- ----------
AcLPLAT4_XP_062832607       ---------- ---------- ---------- ---------- ---------- ----------
AcLPLAT5_XP_003215489       ---------- ---------- ---------- ---------- ---------- ----------
AcLPLAT6_XP_003216108       ---------- ---------- ---------- ---------- ---------- ----------
AcLPLAT7_XP_016846149       ---------- ---------- ---------- ---------- ---------- ----------
AcLPLAT8_XP_016848437       ---------- ---------- ---------- ---------- ---------- ----------
AcLPLAT9_XP_003222971       ---------- ---------- ---------- ---------- ---------- ----------
AcLPLAT10_XP_062813319      ---------- ---------- ---------- ---------- ---------- ----------
GgLPLAT1_XP_040543510       ---------- ---------- ---------- ---------- ---------- ----------
GgLPLAT2_XP_001235300       ---------- ---------- ---------- ---------- ---------- ----------
GgLPLAT3_XP_004934656       ---------- ---------- ---------- ---------- ---------- ----------
GgLPLAT4_XP_040523863       ---------- ---------- ---------- ---------- ---------- ----------
GgLPLAT5_XP_040522440       ---------- ---------- ---------- ---------- ---------- ----------
GgLPLAT6_XP_025004424       ---------- ---------- ---------- ---------- ---------- ----------
GgLPLAT7_NP_001026220       ---------- ---------- ---------- ---------- ---------- ----------
GgLPLAT8_XP_004935228       ---------- ---------- ---------M RRGEGARPRL APPFPAPPPR ILPTDAETGA
GgLPLAT9_NP_001025739       ---------- ---------- ---------- ---------- ---------- ----------
HsLPLAT1_NP_006402          ---------- ---------- ---------- ---------- ---------- ----------
HsLPLAT2_NP_006403          ---------- ---------- ---------- ---------- ---------- ----------
HsLPLAT3_NP_064517          ---------- ---------- ---------- ---------- ---------- ----------
HsLPLAT4_NP_064518          ---------- ---------- ---------- ---------- ---------- ----------
HsLPLAT5_NP_060831          ---------- ---------- ---------- ---------- ---------- ----------
HsLPLAT6_NP_001002257       ---------- ---------- ---------- ---------- ---------- ----------
HsLPLAT7_NP_055688          ---------- ---------- ---------- ---------- ---------- ----------
HsLPLAT8_NP_079106          ---------- ---------- ---------- ---------- ---------- ----------
HsLPLAT9_NP_060309          ---------- ---------- ---------- ---------- ---------- ----------
HsLPLAT10_NP_705841         ---------- ---------- ---------- ---------- ---------- ----------
```

```
                          61
AqLPLAT1_XP_019853566     ---------- ---------- ---------- ---------- ---------- ----------
AqLPLAT2_XP_011403842     ---------- ---------- ---------- ---------- ---------- ----------
NvLPLAT1_XP_048587027     ---------- ---------- ---------- ---------- ---------- ----------
MgLPLAT1_XP_011449105     ---------- ---------- ---------- ---------- ---------- ----------
MgLPLAT2_XP_011416667     ---------- ---------- ---------- ---------- ---------- ----------
MgLPLAT3_XP_011422898     SKIERYEK-Q RYQREPKDSE SHDDSDRSDK ETTENGGDPL IQRDIAID-- ----AY----
MgLPLAT4_XP_011457228     ---------- ---------- ---------- ---------- ---------- ----------
DmLPLAT1_NP_572828        ---------- ---------- ---------- ---------- ---------- ----------
DmLPLAT2_NP_609231        ---------- ---------- ---------- ---------- ---------- ----------
DmLPLAT3_NP_001036265     ------MTTS TIKPTGGDE- ------VPPA GVV--G---- ---------- ----------
CeLPLAT1_NP_510606        ---------- ---------- ---------- ---------- ---------- ----------
CeLPLAT2_NP_001256175     ---------- ---------- ---------- ---------- ---------- ----------
CeLPLAT3_NP_001348648     ---------- ---------- ---------- ---------- ---------- ----------
CeLPLAT4_NP_504644        ---------- --------MS SLNEP----- ---------- ---------- ----------
CeLPLAT5_Q20800           ---------- ---------- ---------- ---------- ---------- ----------
CeLPLAT6_NP_491479        ---------- ---------- ---------- ---------- ---------- ----------
CeLPLAT7_NP_509260        ---------- ---------- ---------- ---------- ---------- ----------
CeLPLAT8_NP_502370        ---------- ---------- ---------- ---------- ---------- ----------
CeLPLAT9_NP_001371003     ---------- MCDEQTQKLL D--------- --LA------ -------N-- GQRFRPP---
CtLPLAT1_ELT98524         ---------- ---------- ---------- ---------- ---------- ----------
CtLPLAT2_ELU12181         ---------- ---------- ---------- ---------- ---------- ----------
CtLPLAT3_ELU10392         ---------- ---------- ---------- ---------- ---------- ----------
DrLPLAT2_NP_001071200     ---------- ---------- ---------- ---------- ---------- ----------
DrLPLAT3_NP_998590        ---------- ---------- ---------- ---------- ---------- ----------
DrLPLAT4_NP_998157        ---------- ---------- ---------- ---------- ---------- ----------
DrLPLAT5_NP_001070213     ---------- ---------- ---------- ---------- ---------- ----------
DrLPLAT6_NP_998435        ---------- ---------- ---------- ---------- ---------- ----------
DrLPLAT7_NP_001025129     ---------- ---------- ---------- ---------- ---------- ----------
DrLPLAT8_NP_001037806     ---------- ---------- ---------- ---------- ---------- ----------
DrLPLAT9_NP_001018492     ---------- ---------- ---------- ---------- ---------- ----------
DrLPLAT10_NP_991122       ---------- ---------- ---------- ---------- ---------- ----------
XtLPLAT1_XP_012823272     --DNRYDA-A SCVRAGVEY- ----TFRAHR V-FFCCSAEI LQKDRRLT-- ----DRPLCR
XtLPLAT2_NP_001123741     ---------- ---------- ---------- ---------- ---------- ----------
XtLPLAT3_NP_001008119     ---------- ---------- ---------- ---------- ---------- ----------
XtLPLAT4_NP_001011265     ---------- ---------- ---------- ---------- ---------- ----------
XtLPLAT5_NP_001015964     ---------- ---------- ---------- ---------- ---------- ----------
XtLPLAT6_NP_001135517     ---------- ---------- ---------- ---------- ---------- ----------
XtLPLAT7_XP_017949604     ------GSQS RCWEEGVEVE EEGRPTPSSS DRVC------ -------H-- TPGYGGP---
XtLPLAT8_XP_002933165     ---------- ---------- ---------- ---------- ----MRLTKN G---------
XtLPLAT9_XP_012815924     ---------- ---------- ---------- ---------- ---------- -------MCL
XtLPLAT10_NP_001037863    ---------- ---------- ---------- ---------- MSE------- A---------
AcLPLAT1_XP_062829728     ---------- ---------- ---------- ---------- ---------- ----------
AcLPLAT2_XP_062816491     ---------- ---------- ---------- ---------- ---------- ----------
AcLPLAT3_XP_008105662     ---------- ---------- ---------- ---------- ---------- ----------
AcLPLAT4_XP_062832607     ---------- ---------- ---------- ---------- ---------- ----------
AcLPLAT5_XP_003215489     ---------- ---------- ---------- ---------- ---------- ----------
AcLPLAT6_XP_003216108     ---------- ---------- ---------- ---------- ---------- ----------
AcLPLAT7_XP_016846149     ---------- ---------- ---------- ---------- ---------- ----------
AcLPLAT8_XP_016848437     ----MRLKGR GCFAPGERG- ------REGG GGGE-GAGEE E--------- ----------
AcLPLAT9_XP_003222971     ---------- ---------- ---------- ---------- ---------- ----------
AcLPLAT10_XP_062813319    ---------- --MEGGGGG- ------RGGG GGGE-GGEGG EGG------- G---------
GgLPLAT1_XP_040543510     ---------- ---------- ---------- ---------- ---------- ----------
GgLPLAT2_XP_001235300     ---------- ---------- ---------- ---------- ---------- ----------
GgLPLAT3_XP_004934656     ---------- ---------- ---------- ---------- ---------- ----------
GgLPLAT4_XP_040523863     ---------- ---------- ---------- ---------- ---------- ----------
GgLPLAT5_XP_040522440     ---------- ---------- ---------- ---------- ---------- ----------
GgLPLAT6_XP_025004424     ---------- --------MK KRGCP----- ---------- ---------- ----------
GgLPLAT7_NP_001026220     ---------- ---------- ---------- ---------- ---------- ----------
GgLPLAT8_XP_004935228     GRAAVAGAQG ACAPPGGRG- ------PAPS GERA-AAAAA ARRRRRLRSS GRRAVPSLCP
GgLPLAT9_NP_001025739     ---------- ---------- ---------- ---------- ---------- ----------
HsLPLAT1_NP_006402        ---------- ---------- ---------- ---------- ---------- ----------
HsLPLAT2_NP_006403        ---------- ---------- ---------- ---------- ---------- ----------
HsLPLAT3_NP_064517        ---------- ---------- ---------- ---------- ---------- ----------
HsLPLAT4_NP_064518        ---------- ---------- ---------- ---------- ---------- ----------
HsLPLAT5_NP_060831        ---------- ---------- ---------- ---------- ---------- ----------
HsLPLAT6_NP_001002257     ---------- ---------- ---------- ---------- ---------- ----------
HsLPLAT7_NP_055688        ---------- ---------- ---------- ---------- ---------- ----------
HsLPLAT8_NP_079106        ---------- ---------- ---------- ---------- ----MRLRGC GPRAAPA---
HsLPLAT9_NP_060309        ---------- ---------- ---------- ---------- ---------- ----------
HsLPLAT10_NP_705841       ---------- ---------- ---------- ---------- MSQ------- G---------
```

```
                        121
AqLPLAT1_XP_019853566   ----MILH-- ---------- ---------- ---------- ----AP---- ----------
AqLPLAT2_XP_011403842   ---------- ---------- ---------- ---------- ---------- ----------
NvLPLAT1_XP_048587027   ---------- ---------- ---------- ---------- ---------- ----------
MgLPLAT1_XP_011449105   ---------- ---------- ---------- ---------- ---------- ----------
MgLPLAT2_XP_011416667   ---------- ---------- ---------- ---------- ---------- -------MSE
MgLPLAT3_XP_011422898   --QGIRIPSN --RADYDLTN LKKEFHMSDI M--YF---AK CGVETI-IED EVTKCFTAEE
MgLPLAT4_XP_011457228   ---------- ---------- ---------- -----MKRPV VVPRQE-SLM YPIVKNPFVH
DmLPLAT1_NP_572828      ---------- ---------- ---------- ---------- ---------- ----------
DmLPLAT2_NP_609231      ---------- ---------- ---------- ---------- ---------- ----------
DmLPLAT3_NP_001036265   ---------- ---------- -----NVADV GVAASLTKRD SEEDTW-ASK GYNYINPFVH
CeLPLAT1_NP_510606      ---------- ---------- ---------- ---------- ---------- ----------
CeLPLAT2_NP_001256175   ---------- ---------- ---------- ---------- ---------- ----------
CeLPLAT3_NP_001348648   ---------- ---------- ---------- ---------- ---------- ----------
CeLPLAT4_NP_504644      ---------- ---------- ---------- ---------P VLGAGQ---- ----------
CeLPLAT5_Q20800         ---------- ---------- ---------- ---------- --MRIP---- ----------
CeLPLAT6_NP_491479      ----MLIS-- ---------- ---------- ---------- ----LT---- ----------
CeLPLAT7_NP_509260      ----MLKS-- ---------- ---------- --------G- -LMDTDDQKV GVRVAN--ID
CeLPLAT8_NP_502370      ---------- ---------- ---------- ---------- --MEAE---- ----------
CeLPLAT9_NP_001371003   -------G-- ---------- ---------- --------S- -RLVIE---- ----------
CtLPLAT1_ELT98524       ---------- ---------- ---------- ---------- ---------- ----------
CtLPLAT2_ELU12181       ---------- ---------- ---------- ---------- ---------- ----------
CtLPLAT3_ELU10392       ---------- ---------- ---------- ---------- ---------- ----------
DrLPLAT2_NP_001071200   ---------- ---------- ---------- ---------- ---------- ----------
DrLPLAT3_NP_998590      ---------- ---------- ---------- ---------- ---------- ----------
DrLPLAT4_NP_998157      ---------- ---------- ---------- ---------- ---------- ----------
DrLPLAT5_NP_001070213   ----MLLS-- ---------- ---------- ---------- ----LV---- ----------
DrLPLAT6_NP_998435      ---------- ---------- ---------- ---------- ---------- ----------
DrLPLAT7_NP_001025129   ---------- ---------- ---------- ---------- --MAPH---- ----------
DrLPLAT8_NP_001037806   -------MRF P--------- -NR------- --KLH----T AVNGGD-SGV STHFRNPFVH
DrLPLAT9_NP_001018492   ---------- ---------- ---------- --MPPPHRVF ALPRQQ-SLL LPAVINPFVH
DrLPLAT10_NP_991122     ---------- ---------- ---------- ---------- -----M-LKN KGQTAHPFIH
XtLPLAT1_XP_012823272   FHAGMSSNVV --KAASPLAS ----AVTGKL Q--FP---WQ CNLQS----- GRPFCNNFAN
XtLPLAT2_NP_001123741   ---------- ---------- ---------- ---------- ---------- ----------
XtLPLAT3_NP_001008119   ---------- ---------- ---------- ---------- ---------- ----------
XtLPLAT4_NP_001011265   ---------- ---------- ---------- ---------- ---------- ----------
XtLPLAT5_NP_001015964   ----MLLS-- ---------- ---------- ---------- ----AV---- ----------
XtLPLAT6_NP_001135517   ---------- ---------- ---------- ---------- ---------- ----------
XtLPLAT7_XP_017949604   -RRGCCRS-- ---------- ---------- --------G- -KMAIT---- ----------
XtLPLAT8_XP_002933165   ---------- ---------- ---------- --RAVSHGLE AGDGRV-EEL ARPVRNPFVH
XtLPLAT9_XP_012815924   LFRPFCADPC SASVQWDVPW LPVALQLQKM ANKVEQQRVP SLPRQK-SFI PPQIPNPFVH
XtLPLAT10_NP_001037863  ---------- ---------- ---------- --EPV---GE KGPAED-DGE ESVPFNPFLH
AcLPLAT1_XP_062829728   ---------- ---------- ---------- ---------- ---------- ----------
AcLPLAT2_XP_062816491   ---------- ---------- ---------- ---------- ---------- -------MEE
AcLPLAT3_XP_008105662   ---------- ---------- ---------- ---------- ---------- ----------
AcLPLAT4_XP_062832607   ---------- ---------- ---------- ---------- ---------- ----------
AcLPLAT5_XP_003215489   ----MLLS-- ---------- ---------- ---------- ----LV---- ----------
AcLPLAT6_XP_003216108   ---------- ---------- ---------- ---------- ---------- ----------
AcLPLAT7_XP_016846149   ---------- ---------- ---------- ---------- --MAVT---- ----------
AcLPLAT8_XP_016848437   ---------- ---------- ---------- ---------Q KRQQGR-RPP PPPLRSPFAH
AcLPLAT9_XP_003222971   ---------- ---------- ------MSSS PPAPSSPLPP PFPRQE-SLA SPTVLNPFVQ
AcLPLAT10_XP_062813319  ---------- ---------- ---------- ---GP---GR APPK---EED PATEPNPFVH
GgLPLAT1_XP_040543510   ---------- ---------- ---------- ---------- ---------- ----------
GgLPLAT2_XP_001235300   ---------- ---------- ---------- ---------- ---------- ----------
GgLPLAT3_XP_004934656   ---------- ---------- ---------- ---------- ---------- ----------
GgLPLAT4_XP_040523863   ---------- ---------- ---------- ---------- ---------- ----------
GgLPLAT5_XP_040522440   ----MLLS-- ---------- ---------- ---------- ----LV---- ----------
GgLPLAT6_XP_025004424   ----LFVS-- ---------- ---------- --------SG CGAENS---- ----------
GgLPLAT7_NP_001026220   ---------- ---------- ---------- ---------- --MAVT---- ----------
GgLPLAT8_XP_004935228   FSLPLSSRPF P--------- -AM------- --KLSSRGRG CCVAGS-RER GPPPRSPFVH
GgLPLAT9_NP_001025739   ---------- ---------- ---------- ----MAQRTL PLPRQQ-SFS PPVVPNPFVY
HsLPLAT1_NP_006402      ---------- ---------- ---------- ---------- ---------- ----------
HsLPLAT2_NP_006403      ---------- ---------- ---------- ---------- ---------- ----------
HsLPLAT3_NP_064517      ---------- ---------- ---------- ---------- ---------- ----------
HsLPLAT4_NP_064518      ---------- ---------- ---------- ---------- ---------- ----------
HsLPLAT5_NP_060831      ----MLLS-- ---------- ---------- ---------- ----LV---- ----------
HsLPLAT6_NP_001002257   ---------- ---------- ---------- ---------- ---------- ----------
HsLPLAT7_NP_055688      ---------- ---------- ---------- ---------- --MAIT---- ----------
HsLPLAT8_NP_079106      ---------- ---------- ---------- --------SS AGASDA-RLL APPGRNPFVH
HsLPLAT9_NP_060309      ------MSRC AQAA------ -EVAATVPGA GVGNVGLRPP MVPRQA-SFF PPPVPNPFVQ
HsLPLAT10_NP_705841     ---------- ---------- ---------- ---SP---GD WAPLDP-TPG PPASPNPFVH
```

```
                              181
AqLPLAT1_XP_019853566     -HLIL-SLRC LVPSVFL-M- -----GS--- ------STSY W--------- ---VAATL--
AqLPLAT2_XP_011403842     --MAS-MLKG VCFVLLIVI- -----TA--- ------VFGL H--------- ----------
NvLPLAT1_XP_048587027     ---------- -MFLECSLSA LLLGVVGFHI LYRISRWFQF YVDY-----F SYNIAMVLL-
MgLPLAT1_XP_011449105     ---------- ---M--L--- IWSLLILVFA LYFLSNTFRY YVKY-----G LYLLFLTVI-
MgLPLAT2_XP_011416667     TENSEVFGIS YSVI------ -LSSLVVGIV TWIFYDKIQF FGKH-----L IANLSWQFF-
MgLPLAT3_XP_011422898     -------LRT WNFLTRTTHG HQYVSVRLTV LWCLGCLLRY LLLPFRLVLL SIGLSWLLI-
MgLPLAT4_XP_011457228     NLQLST---- LDIVRIVF-- --MSITIA-- ------PVRL VSLA-----L LFGLAWLLA-
DmLPLAT1_NP_572828        ---MI-TMTS FIEL--L--- GLFLLLMLPF LYETNHIFRY YFKF-----L MYYGIVSFN-
DmLPLAT2_NP_609231        ---------- -MACTCE--- VIGLACVVAL ILSATAKAPY QMRM-----F FIFFGAGLI-
DmLPLAT3_NP_001036265     RIEIDSH--- IEVAKIYV-- --LTVLLL-- ------PIRV VGCV-----L SLISAWMFA-
CeLPLAT1_NP_510606        ---MT----- FLA------- ILF-VIAVLL LLAQLPVIGF YIRA-----V -YFGMCLII-
CeLPLAT2_NP_001256175     ---MENF--- WSIV------ VFFLLSILFI LYNISTVCHY YMRI-----S FYYFTILLH-
CeLPLAT3_NP_001348648     ---MK-VIKG VTFIVLVFF- -----SS--- ------LLGT V--------- ----------
CeLPLAT4_NP_504644        -QIGT-RLKG WFFGAAMLL- -----SA--- ------FFGV L--------- ----------
CeLPLAT5_Q20800           -CLLR-PLLG WFFGLCILF- -----SA--- ------LFGN Y--------- ----------
CeLPLAT6_NP_491479        -IAVD-ALRP IIPCSLL-S- -----LS--- ------MVPF A--------- ---SCAIV--
CeLPLAT7_NP_509260        MSERT-DNVH LIEIRRIIS- -----LV--- ------GAAY FFF------- ---MTAWVVP
CeLPLAT8_NP_502370        --NDN-KKQG WTLIDNVIS- -----CI--- ------LLVY AGI------- ---VTAFICP
CeLPLAT9_NP_001371003     -QGSF-ASRL WWLVKAPFR- -----TL--- ---------- MCL------- ---SLVTIFF
CtLPLAT1_ELT98524         ---------- ---------- ---------- ---------- ---------- ----------
CtLPLAT2_ELU12181         ---MALG--- WIELTLI--- --GLATLFPI VWRASCSVRY YVKI-----F VYFSFMMLT-
CtLPLAT3_ELU10392         ---MG-GWWG WMKANFLPL- -----HI--- ------LIGY VFV------- ---VSGLI-V
DrLPLAT2_NP_001071200     ------MDAV WMLP------ ---VLLVLPL LLWTSSTFVF YFKK-----C FYVAYMMLL-
DrLPLAT3_NP_998590        ----M-GLVA WLKSLFV-L- -----QL--- ------LIGF VFV------- ---VSGLI-I
DrLPLAT4_NP_998157        -MIRM-GLLK VLKTQLL-C- -----HL--- ------IICY VFL------- ---VSGII-I
DrLPLAT5_NP_001070213     -VHTY-SLRY WLPTAVM-L- -----GT--- ------APAY L--------- ---LSWSV--
DrLPLAT6_NP_998435        ---MV-SPRG VCFLLFLLL- -----GS--- ------VFGS V--------- ----------
DrLPLAT7_NP_001025129     -LDVA-RKLV WILIKSLLR- -----FT--- ---------- FMF------- ---VNNCVAI
DrLPLAT8_NP_001037806     ELRFTT---- LQKLKIAV-- --MTVTLF-- ------PVRL LFAA-----F MMLLAWPFA-
DrLPLAT9_NP_001018492     DLSLST---- ADITKCFL-- --LGIILV-- ------PLRA IFLL-----L VLLVMWPVS-
DrLPLAT10_NP_991122       EVNLT-A--- LQRIQGLV-- --LGVFLF-- ------PVRI TLAV-----L FFLLMWPIA-
XtLPLAT1_XP_012823272     TGAMELSVAQ WLLVVCL--- -----VAFPL LYEWSVSFKY FCKM-----A FYNGWILTL-
XtLPLAT2_NP_001123741     ---MDLW--- WLLW---IS- --LLFLVPFI LMEISSGFKF HFKL-----V YYCAMCLLL-
XtLPLAT3_NP_001008119     ----M-GFWG FLKTQFV-V- -----HL--- ------LIGF VFV------- ---VSGLI-I
XtLPLAT4_NP_001011265     ----M-DIAG FLKSQFL-C- -----HL--- ------FISY VFI------- ---GTGLI-V
XtLPLAT5_NP_001015964     -LYTY-ALRH MVPTAFM-L- -----GT--- ------APTY L--------- ---LAWGC--
XtLPLAT6_NP_001135517     ---MV-SWRG VYFVLALFL- -----GS--- ------FFGS I--------- ----------
XtLPLAT7_XP_017949604     -ADST-GRMG FLILRMVLR- -----FA--- ---------- FMV------- ---VNNMVAI
XtLPLAT8_XP_002933165     DLRFSP---- LQKAKILF-- --MTLTVF-- ------PIRL FFAA-----F MMLLAWPFA-
XtLPLAT9_XP_012815924     EINLSP---- VNKLKMVL-- --CGLFLL-- ------PLRV ILFM-----I VLLVSWLVA-
XtLPLAT10_NP_001037863    EFEPEGF--- WQKARFYI-- --LGFTLF-- ------PLRF LLAA-----I FLFLMWPIA-
AcLPLAT1_XP_062829728     ---MEISLGQ WILILIL--- -----VVVPL LYEWSTTFKY FCKM-----A FYNSYILFL-
AcLPLAT2_XP_062816491     PLSPAVG--- WPWL---SL- --ALLVGLPG VLALSPRLRF RFKV-----G FYCVLCLVC-
AcLPLAT3_XP_008105662     ----M-GLIA FLKTQFI-V- -----HL--- ------LIGF VFV------- ---VSGFI-I
AcLPLAT4_XP_062832607     ----M-NIAG FLKSQFI-F- -----HL--- ------LISY IFI------- ---ASGLI-I
AcLPLAT5_XP_003215489     -LHTY-SMRY VLPAALM-L- -----GA--- ------APTY V--------- ---VAWGA--
AcLPLAT6_XP_003216108     ---MV-SWKG IYFVLALFL- -----GS--- ------FFGS I--------- ----------
AcLPLAT7_XP_016846149     -LEQA-QRAG YVFLKALLR- -----FG--- ---------- FMV------- ---ANNLVAI
AcLPLAT8_XP_016848437     QLRFSA---- LQKAKIAF-- --MTLTLF-- ------PIRL FFAA-----F MMLLAWPFA-
AcLPLAT9_XP_003222971     EARLDP---- QAKTRAIL-- --FGLILV-- ------PLRL ICMF-----L ILAMAWPFA-
AcLPLAT10_XP_062813319    DLRMS-R--- FQRAKFYV-- --LGIILA-- ------PIRV ALAF-----V VLFLIWPFA-
GgLPLAT1_XP_040543510     ---MELL--- --LLHYPFT- --FLLIAVLI LCWRYPTFKY FFKM-----T FYQVWIFTI-
GgLPLAT2_XP_001235300     ---MEPG--- WLLW---GA- --AVLLLLHL LMELSSAVNF VVRI-----G FYYVLCIVC-
GgLPLAT3_XP_004934656     ----M-GFIA FLKTQFI-V- -----HL--- ------LIGF VFV------- ---VSGLI-I
GgLPLAT4_XP_040523863     ----M-DIVG FLKSQFI-F- -----HL--- ------LICY IFI------- ---VSGLI-I
GgLPLAT5_XP_040522440     -LHTY-SMRY FLPAAVM-M- -----GT--- ------APTY V--------- ---LAWGA--
GgLPLAT6_XP_025004424     -RAMV-SWKG IYFVVALFL- -----GS--- ------FFGS I--------- ----------
GgLPLAT7_NP_001026220     -LEQA-QRVG YTFLKALLR- -----FA--- ---------- FMV------- ---ANNLVAI
GgLPLAT8_XP_004935228     QLRFSP---- LEKTKIAF-- --MTLTLF-- ------PIRL FFAA-----F MMLLAWPFA-
GgLPLAT9_NP_001025739     PGRLSA---- GDKLRLVL-- --LGIILL-- ------PLRA ICMV-----I LLLLSWLSA-
HsLPLAT1_NP_006402        ---MDLWPGA WMLLLLLFL- --LLLFLLPT LWFCSPSAKY FFKM-----A FYNGWILFL-
HsLPLAT2_NP_006403        ----MEL--- WPCL---AA- --AL-LLLLL LVQLSRAAEF YAKV-----A LYCALCFTV-
HsLPLAT3_NP_064517        ----M-GLLA FLKTQFV-L- -----HL--- ------LVGF VFV------- ---VSGLV-I
HsLPLAT4_NP_064518        ----M-DLAG LLKSQFL-C- -----HL--- ------VFCY VFI------- ---ASGLI-I
HsLPLAT5_NP_060831        -LHTY-SMRY LLPSVVL-L- -----GT--- ------APTY V--------- ---LAWGV--
HsLPLAT6_NP_001002257     ---MV-SWKG IYFILTLFW- -----GS--- ------FFGS I--------- ----------
HsLPLAT7_NP_055688        -LEEA-PWLG WLLVKALMR- -----FA--- ---------- FMV------- ---VNNLVAI
HsLPLAT8_NP_079106        ELRLSA---- LQKAQVAL-- --MTLTLF-- ------PVRL LVAA-----A MMLLAWPLA-
HsLPLAT9_NP_060309        QTQIGS---- ARRVQIVL-- --LGIILL-- ------PIRV LLVA-----L ILLLAWPFA-
HsLPLAT10_NP_705841       ELHLS-R--- LQRVKFCL-- --LGALLA-- ------PIRV LLAF-----I VLFLLWPFA-
```

```
                            241
AqLPLAT1_XP_019853566      ----LKLISL P---F-GGQR GRSLYHRMDQ RLYSSMMAMF GYFFEHWSGV -QYCFYGDPL
AqLPLAT2_XP_011403842      ----VLFIPA I---L-TFFI SPGLHTVYVS GILYFWTHLV SGLFEL-LGI -RMKLVGDLK
NvLPLAT1_XP_048587027      ----GCL-AV L--VA---LL --RPGNVNNF YRTSR---LL YWISSKYFRV -RVEAKGLEN
MgLPLAT1_XP_011449105      ----STV-TV F--WV---IL TCRPKSTENS RFAN----WA LVKLKYFYGW -EFEIHGKEY
MgLPLAT2_XP_011416667      ----SIF-PI L--AS---LF --YGRIPENR RLFSI---GV QLTNKYLIGT -KYIVRNQKY
MgLPLAT3_XP_011422898      ----STAVIG L--LP---DI --SLRRQLNR YASL----IC HRILCRACSA -VVTYHGREN
MgLPLAT4_XP_011457228      ----AISLAY RTPEE-KKKP LEGWRNSLR- PLVV----FV SRCVFFVGGF HWLTVKGEQH
DmLPLAT1_NP_572828         ----SII-L- ---IP---AF LTRPCDVRNL LWAS----TW CHRVSTLIGL -RWELRGKEH
DmLPLAT2_NP_609231         ----VFL-CV P--FM---IL --RPRDYRNA LFPS----WC FRQLCRLVGI -TMEVRGLEN
DmLPLAT3_NP_001036265      ----CIGLYG MTLDDLKAKP LTGWRKQM-Q YMTA----CG MRMVYTFGSF HYVTMKGRAA
CeLPLAT1_NP_510606         ----GGFLGG L--AS---IP --FGKSPNNH FRMF----KI FQAMTWPMGV -RFELRNSEI
CeLPLAT2_NP_001256175      ----GME-VC VTMIP---SW L-NGKGAD-Y VFHS----FF Y--WCKWTGV -HTTVYGYEK
CeLPLAT3_NP_001348648      ----FLLFPL I---P-LAWF APKLWRTCAD RLVGFWLTFP CSLIEWVFGV -NFRVTGDLI
CeLPLAT4_NP_504644         ----YIVTPL V---V-LLFL KPRLWRQMLD RLVGIWVIMP GALCNYIFGA -NIRIKGDFI
CeLPLAT5_Q20800            ----IITLFL G---L-PILG RHKQWRNLMD RAISYWMTIP MGLLEFLMGV -RIRVSGDEI
CeLPLAT6_NP_491479         ----I----G G---V-SWIV PRHVAQQLDN MLYKSYMRLC LFVFENLSGV -EIYLHGTNE
CeLPLAT7_NP_509260         VACVITVSLL F---P-LMLF STPLFNYL-E HKLCAMVNAH WNAVSVFVGA -TVTEYGTNL
CeLPLAT8_NP_502370         IVFLCFFYTA A---P-IMLF STEFRKFV-M KIMARAGNLQ FITCVRITGI -KVMMSGDGL
CeLPLAT9_NP_001371003      ATYFGFMLPV M---W-ARTV WPRLYWFV-E GKLYRWLQSF IAYWGYTAGY -DVYEYGDDV
CtLPLAT1_ELT98524          ---------- --MIL---LI YPFPFRYRHR IFVKTWATVA INLCRFICGV -RWSIRGKEN
CtLPLAT2_ELU12181          ----GFV-MI P--IV---MW --RPKHPRNL MYVR----NF WNFSLRMFQW -EVEVRDREN
CtLPLAT3_ELU10392          --CGLMALTY L---L-VWPW NRQLYRKIVM NLAYTHWCQF TFLGQWWAGC -NCTLHLDNK
DrLPLAT2_NP_001071200      ----AVI-AI P--IC---IL KSGGRDIENM RVIR----FL VRHVKYFLGL -RYQVSGWEH
DrLPLAT3_NP_998590         --N-FTQLCT C---V-LWPF DKQLYRRINT RLSYSLWSQL VMLLEWWSGT -ECTLYTDQA
DrLPLAT4_NP_998157         --N-LLQLCT L---P-LWPI NKQLARKINC RLGYSIASQL VALLEWWSGT -ECTLYTDPE
DrLPLAT5_NP_001070213      ----WRLLSA ----I----L PARLYHTVDD RAYSVYQSMV LFFFENYTGV -EIVIYGE-I
DrLPLAT6_NP_998435         ----FMLGPL L---P-LMLL SPSRYRWITD RIVATWLTLP VALLELVLGV -KVVVTGDGF
DrLPLAT7_NP_001025129      PSYCLYLIVL Q---P-LRVL DAQTFWYI-E GVMFRWLLAM VASWGWCAGY -TVTEWGDDV
DrLPLAT8_NP_001037806      ----FVATVG RSENA-V-EP LSWWRWLV-D LALK----AI MRAMWFSGGF HWVRVKGRPA
DrLPLAT9_NP_001018492      ----VIITFG QSLKG-VVEP MTGWRRFLHR RVMT----FL GRMYFFGMGF -KVVVKGKKA
DrLPLAT10_NP_991122        ----RLRLAG LPESR-RAEP VRGWRRWLFH HVMV----FL SRAVFFCVGF LWVRVKGRQA
XtLPLAT1_XP_012823272      ----AIL-AI P--IC---AV --RGRNVENM KVLQ----FM LLHIKYLYGI -KIEVRGWEN
XtLPLAT2_NP_001123741      ----SAL-AA P--MC---LL TNGGRTVNNM RIIS----RV VRTLKYFFGV -RFEVKGLEN
XtLPLAT3_NP_001008119      --N-FLQLCT L---P-LWGI NKPLYRRVNC RMSYLLWSQL VMLLEWWSGT -QCTLFTDQE
XtLPLAT4_NP_001011265      --N-FIQLCT L---I-VWPF NKQLYRKINC RLAYCISSQL VMLLEWWSNT -DCTLYTDPE
XtLPLAT5_NP_001015964      ----WRALSA ----V----L PERIYRSVDD RLYTLYQSMV LFFFQNYTGV -EIIIYGD-L
XtLPLAT6_NP_001135517      ----FMLGPF L---P-LMLV SPSWYRWITD RIVATWLTLP VALLELVFGA -KVVITGDGF
XtLPLAT7_XP_017949604      PSYVLYLIAL Q---P-VRVI DRKLFWYI-E GVMFKWLLAM VASWGWYAGY -TVVEWGDNV
XtLPLAT8_XP_002933165      ----FIAAMG RTEKD-LEKP LSWWRKVL-D LLLK----AI MRTMWFAGGF HWVTVKGQQA
XtLPLAT9_XP_012815924      ----AIATCC CPEKN--EKP LEGWRRKVTQ TLIG----SL GRLLFFSMGF -HVRVEGKPA
XtLPLAT10_NP_001037863     ----ALRVAG LTEEE-LSRS IR-HRRTILH HLIY----LL SRTMFFMCGF HWITIRGRRA
AcLPLAT1_XP_062829728      ----AVI-VI P--MC---AV --RGRNVENM KLIR----FM LLHVKYLYGV -KIEVRGSEN
AcLPLAT2_XP_062816491      ----SAL-TA P--AC---LA WNRGRTVHNM TIIK----NV VRSFKYFYGL -RFETKGLEH
AcLPLAT3_XP_008105662      --N-FIQLCT L---I-LWPI NKQFYRRVNC RLSYSLWSQL VMLLEWWSGT -ECTLFSDQD
AcLPLAT4_XP_062832607      --N-FIQLFT L---I-LWPF NKQLFRKVNC KLAYCLSSQL VMLLEWWSGT -TCTLYTDPG
AcLPLAT5_XP_003215489      ----WRLLAA L---L----L PARLYHRVDD RFYTLYQSMV LFFFENYTGV -QIFIYGD-L
AcLPLAT6_XP_003216108      ----FMLAPV L---P-LMFV SPAWYRWITD RVVATWLTLP VALLEIVFGA -KVVITGDGF
AcLPLAT7_XP_016846149      PSYVLYLIAL Q---P-LRLL DSKRFWYI-E GVLFKWLLAM VASWGWWAEY -TVIEWGDDV
AcLPLAT8_XP_016848437      ----FIASIG TAEKE-LEQP LSWWRKVV-D FLLK----AI MRTMWLAGGF HWINVKGRQA
AcLPLAT9_XP_003222971      ----LLATFG APEKG--TMP LQGWKRKVSY SALI----FL GHVLFFVMGF -WVKVKGKVA
AcLPLAT10_XP_062813319     ----LLQVVG LPEET-LQEP FSGWRNTVSH SLVY----LL SRLMFFLLGF MRIRVRGQMA
GgLPLAT1_XP_040543510      ----SFL-VV I--LA---LP --RGRSFENM KILR----TA ILTVKYALGV -KIVVKGKEN
GgLPLAT2_XP_001235300      ----SGL-TA P--VC---LL VNGGRTVKNM RIIK----TV VKSFKYFFGL -RFEVKGLEN
GgLPLAT3_XP_004934656      --N-FIQLCT L---V-LWPI NKQFYRRVNC RLAYSLWSQL VMLLEWWSGT -ECTLFSDEA
GgLPLAT4_XP_040523863      --N-FIQLFT L---I-LWPI NKQLFRRINC RLAYCISSQM VMLLEWWSGT -DCTLYTDPE
GgLPLAT5_XP_040522440      ----WRLLSA ----V----L PARFYREVDD RLYTIYQSMV LFFFENYAGV -QVIIYGD-L
GgLPLAT6_XP_025004424      ----FMLGPF L---P-LMFI SPAWYRWITD RIVATWLTLP VALLEMVFGA -KVVVTGDGF
GgLPLAT7_NP_001026220      PSYVLYLIVL Q---P-LRVL DSESFWYI-E GVLFKWLLAM VASWGWWAGY -TVVEWGDDV
GgLPLAT8_XP_004935228      ----FIASMG CDEQE-LEKP LSWWRKIV-D ILLK----AI MRMMWLAGGF HWINVKGRRA
GgLPLAT9_NP_001025739      ----SVATFR HTGGA--SVP LKGWRRSMIQ ATLS----CL TRTLFFVMGF -RVKVKGKVA
HsLPLAT1_NP_006402         ----AVL-AI P--VC---AV --RGRNVENM KILR----LM LLHIKYLYGI -RVEVRGAHH
HsLPLAT2_NP_006403         ----SAV-AS L--VC---LL RHGGRTVENM SIIG----WF VRSFKYFYGL -RFEVRDPRR
HsLPLAT3_NP_064517         --N-FVQLCT L---A-LWPV SKQLYRRLNC RLAYSLWSQL VMLLEWWSCT -ECTLFTDQA
HsLPLAT4_NP_064518         --N-TIQLFT L---L-LWPI NKQLFRKINC RLSYCISSQL VMLLEWWSGT -ECTIFTDPR
HsLPLAT5_NP_060831         ----WRLLSA ----F----L PARFYQALDD RLYCVYQSMV LFFFENYTGV -QILLYGD-L
HsLPLAT6_NP_001002257      ----FMLSPF L---P-LMFV NPSWYRWINN RLVATWLTLP VALLETMFGV -KVIITGDAF
HsLPLAT7_NP_055688         PSYICYVIIL Q---P-LRVL DSKRFWYI-E GIMYKWLLGM VASWGWYAGY -TVMEWGEDI
HsLPLAT8_NP_079106         ----LVASLG SAEKE-PEQP PALWRKVV-D FLLK----AI MRTMWFAGGF HRVAVKGRQA
HsLPLAT9_NP_060309         ----AISTVC CPEKL--THP ITGWRRKITQ TALK----FL GRAMFFSMGF -IVAVKGKIA
HsLPLAT10_NP_705841        ----WLQVAG LSEEQ-LQEP ITGWRKTVCH NGVL----GL SRLLFFLLGF LRIRVRGQRA
```

```
                         301
AqLPLAT1_XP_019853566    -----PQTPE SVIYISNHQS TVDWLIPE-I LCLKQG--GF GGR--VHYFL KKAVLYIPLY
AqLPLAT2_XP_011403842    ---K-VSKSV PTLIILNHRT RFDWFFLF-T MLRHVG--RL GN---VRIIL KSSLKSVPIF
NvLPLAT1_XP_048587027    -----LPENK NCIIVSNHQS SLDMFPIL-R ICP--P---- YT---T-FIA KRELLFAPFF
MgLPLAT1_XP_011449105    -----LMKDE PCVVLINHQS GLDLIGLM-E IWNWRY---- HC---A-VVG KRSLMYFGMF
MgLPLAT2_XP_011416667    -----LEDDK ACVIVSNHQS SLDGNFMV-Q LGNYKG---- SI---V-ILK KSLLYFSGLL
MgLPLAT3_XP_011422898    -----MVKNG IC--VANHTS PLDVIILS-C -----D---- NC---YSLVG QRH---GGFL
MgLPLAT4_XP_011457228    -----TAKEA PIIALAPHSS FLDALVIVYL NLS------- ------TVVA KQETSRAPVL
DmLPLAT1_NP_572828       -----LAKDQ ACIIVANHQS SLDVLGMF-N IWHVMN---- KC---T-VVA KRELFYAWPF
DmLPLAT2_NP_609231       -----VRKDH GAVVIMNHQS AVDLCVLA-Y LWPVIG---- RA---T-VVS KKEVLYIPFF
DmLPLAT3_NP_001036265    -----TAKEA PILVVAPHSS YVDSILVVAS GPP------- ------SIVA KRETADIPLL
CeLPLAT1_NP_510606       -----LHDKK PYIIIANHQS ALDVLGMS-F AWP--V---- DC---V-VML KSSLKYLPGF
CeLPLAT2_NP_001256175    -----TQVEG PAVVICNHQS SLDILSMA-S IWP--K---- NC---V-VMM KRILAYVPFF
CeLPLAT3_NP_001348648    ------ERDE PAILIMNHRT RLDWLFSW-N ALYKMDPWLL TT---EKISL KAPLKKIPGA
CeLPLAT4_NP_504644       ------NHEE PALIIMNHRT RLDWLFFW-N ALYKMDPWLC TT---EKISL KGMLKYVPGA
CeLPLAT5_Q20800          ------EFGS PAMIVMNHRT RLDWMYMW-C ALYQINPWLI TS---NKISL KAQLKKLPGA
CeLPLAT6_NP_491479       EVVNKTGKPE NAVMISNHQS NVDWIIPV-M LAARHG--DQ GNEQAFRVMV KNSIHLVPMF
CeLPLAT7_NP_509260       AG---YAEEK C-LLLANHLG LLDHFVLM-Q SLNGKGS-IR SR---WMWVI YNIWKY-TPL
CeLPLAT8_NP_502370       ER---LTDCR A-LLLPNHLG LFDHFIFM-T AADSFGVNAV GR---WIFVI YNMWIY-SPL
CeLPLAT9_NP_001371003    TT---YYRDE RVLMMCNHQS TADVPTLM-T VLQNKGV-AS RK---TLWLM DVMFRW-TPF
CtLPLAT1_ELT98524        -----IP-DK TCVVISNHQS TWETFFLQ-T LLT------- PQ---T-QVI KQELLNIPFY
CtLPLAT2_ELU12181        -----VEYMK PAVIVSNHQS ALDMIAMM-Q AMP--E---- RT---T-ALA KKELLFTGAW
CtLPLAT3_ELU10392        DDLK-YIGHE HVVALMNHKY DIDWLMAW-L LSERFA--ML GG---TKIYG KSSLKMVPLI
DrLPLAT2_NP_001071200    -----LQTEG PYVIISNHQS SLDVLGMV-E ILP--D---- RC---T-MIA KKELIWAGTV
DrLPLAT3_NP_998590       T-VD-KFGKE HVIIILNHNF EVDFLCGW-T ICERYG--VL GS---SKVLA KHELLKVPLI
DrLPLAT4_NP_998157       S-FR-LYGKE NAIVVLNHNF EIDFMTGW-T FCERFG--VL GS---SKVLA KKELSFVPVI
DrLPLAT5_NP_001070213    -----PKKKE NVVYLSNHQS TADWIIAD-M LAIRQN--AI G-H--VRYVL KDGLKWLPLY
DrLPLAT6_NP_998435       ------IPGE RSVIIMNHRT RLDWMFLW-C CLLRYS--YL RQ---EKICL KAALKSVPGF
DrLPLAT7_NP_001025129    SP---MTEDE A-MVIVNHQA TGDVCTLM-M CLQDKGT-VV RK---MMWLM DHVFKY-TNF
DrLPLAT8_NP_001037806    -----LPSEA PILTMAPHSS YFDAIPVT-M TMA------- ------SIVM KAESKDIPVW
DrLPLAT9_NP_001018492    -----STLEA PILAVAPHSS FFDAIACIES GLP------- ------STVS RIESLEAPIF
DrLPLAT10_NP_991122      -----GLKEA PVLAVAPHSS FLDMLVLSVT GLP------- ------IVVS RSENAKLPVI
XtLPLAT1_XP_012823272    -----FNIKE PYVVVSNHQS SLDLLGMM-E ILP--S---- RC---V-PIA KRELMYAGTA
XtLPLAT2_NP_001123741    -----FQIDG PCVIISNHQS ILDMMGLM-E ILP--D---- RC---V-QIA KKELMYAGSV
XtLPLAT3_NP_001008119    T-ID-HFGKE HVIIILNHNY EIDFLCGW-T MCERYG--VL GS---SKVLA KKELLMVPLI
XtLPLAT4_NP_001011265    N-YT-KYGKE NAIVVLNHNF EIDFLCGW-N FCERFG--VL GS---SKVLA KKELSYVPVI
XtLPLAT5_NP_001015964    -----PK-KE NILYISNHQC TVDWIVAD-M LAVQQN--AL G-H--VRYVL KDGLKFLPLY
XtLPLAT6_NP_001135517    ------IPGE RSVIIMNHRT RLDWMFLW-N CLLRYS--YL RL---EKICL KSSLKAVPGF
XtLPLAT7_XP_017949604    HS---ISEDE A-VMLVNHQA TGDVCTLM-M CLQDKGT-VV RQ---MMWLM DHIFKY-TNF
XtLPLAT8_XP_002933165    -----LPAEA PILTLAPHSS YFDAIPVT-M TMA------- ------SIVM KAESKDIPVW
XtLPLAT9_XP_012815924    -----TPSDA PILLVAPHSS FFDAIAVIVS GMP------- ------SSVS RAENISVPIF
XtLPLAT10_NP_001037863   -----PASEA PLLVVAPHST FFDPIVTVVC DLP------- ------SVVS RVENLNIPVI
AcLPLAT1_XP_062829728    -----FNIKE PYVIVSNHQS SLDLLGMM-E VLP--D---- RC---V-PIA KKELMYMGTV
AcLPLAT2_XP_062816491    -----FEIEG PCVIVSNHQS ILDMMGLM-E ILP--E---- RC---V-QIG KRELLYFGSV
AcLPLAT3_XP_008105662    T-IN-KFGKE HVIIILNHNF EIDFLCGW-T MTERFG--VL GS---SKVLA KKELLYVPLI
AcLPLAT4_XP_062832607    H-YH-KYGKE NAIVILNHNF EIDFLCGW-S FCDRFG--VL GS---SKVLA KKELSYMPII
AcLPLAT5_XP_003215489    -----PKTKE NVIYISNHQC TVDWIVAD-M LAIRQN--AL G-H--VRYVL KDGLKWLPLY
AcLPLAT6_XP_003216108    ------IPGE RSVIIMNHRT RMDWMFLW-N CLLRYS--YL RL---EKICL KSSLKGIPGF
AcLPLAT7_XP_016846149    KT---ISEDE A-VMLVNHQA TGDICTLM-M CLQDKGT-VV RQ---MMWLM DHIFKY-TNF
AcLPLAT8_XP_016848437    -----LPTEA AILTLAPHSS YFDAIPVT-M TMA------- ------SIVM KAESKDIPVW
AcLPLAT9_XP_003222971    -----RPEEA PIFAVAPHSS FFDGIVCIVA GLP------- ------STVS RQENLMAPFV
AcLPLAT10_XP_062813319   -----SRLEA PILVAAPHST FFDPIILLPC DLP------- ------KVVS RTENLHVPVI
GgLPLAT1_XP_040543510    -----LRTKK PCVLVLNHQN SLDVMVIM-Q MIP--N---- RC---V-PIG KKEILYMGPF
GgLPLAT2_XP_001235300    -----FEVEG PAIIVSNHQS ILDMMGLM-E VLP--D---- GC---V-QVG KKELMYAGTV
GgLPLAT3_XP_004934656    T-VN-TFGKE HVIIILNHNF EIDFLCGW-T MTERFG--VL GS---SKVLA KKELLYVPLI
GgLPLAT4_XP_040523863    N-YH-KYGKE NAIVILNHNF EIDFLCGW-N FCERFG--VL GS---SKVLA KKELSYMPII
GgLPLAT5_XP_040522440    -----PKNKE NIIYLSNHQC TVDWIIAD-M LAIRQN--AL G-H--VRYVL KDGLKWLPLY
GgLPLAT6_XP_025004424    ------IPGE RSVIIMNHRT RMDWMFLW-N CLLRYS--YL RL---EKICL KSSLKSIPGF
GgLPLAT7_NP_001026220    KA---VSEDE A-VVLVNHQA TGDVCTLM-M CLQDKGT-VV RQ---MMWLM DHIFKY-TNF
GgLPLAT8_XP_004935228    -----LPAEA AILTVAPHSS YFDAIPVT-M TFA------- ------SIVM KAESKDIPVW
GgLPLAT9_NP_001025739    -----SLQEA PVFVAAPHSS FFDAIVSALT GMP------- ------SIVS RAENLSTPVF
HsLPLAT1_NP_006402       -----FPPSQ PYVVVSNHQS SLDLLGMM-E VLP--G---- RC---V-PIA KRELLWAGSA
HsLPLAT2_NP_006403       -----LQEAR PCVIVSNHQS ILDMMGLM-E VLP--E---- RC---V-QIA KRELLFLGPV
HsLPLAT3_NP_064517       T-VE-RFGKE HAVIILNHNF EIDFLCGW-T MCERFG--VL GS---SKVLA KKELLYVPLI
HsLPLAT4_NP_064518       A-YL-KYGKE NAIVVLNHKF EIDFLCGW-S LSERFG--LL GG---SKVLA KKELAYVPII
HsLPLAT5_NP_060831       -----PKNKE NIIYLANHQS TVDWIVAD-I LAIRQN--AL G-H--VRYVL KEGLKWLPLY
HsLPLAT6_NP_001002257    ------VPGE RSVIIMNHRT RMDWMFLW-N CLMRYS--YL RL---EKICL KASLKGVPGF
HsLPLAT7_NP_055688       KA---VSKDE A-VMLVNHQA TGDVCTLM-M CLQDKGL-VV AQ---MMWLM DHIFKY-TNF
HsLPLAT8_NP_079106       -----LPTEA AILTLAPHSS YFDAIPVT-M TMS------- ------SIVM KAESRDIPIW
HsLPLAT9_NP_060309       -----SPLEA PVFVAAPHST FFDGIACVVA GLP------- ------SMVS RNENAQVPLI
HsLPLAT10_NP_705841      -----SRLQA PVLVAAPHST FFDPIVLLPC DLP------- ------KVVS RAENLSVPVI
```

AGPAT motif: (1) HssyfD

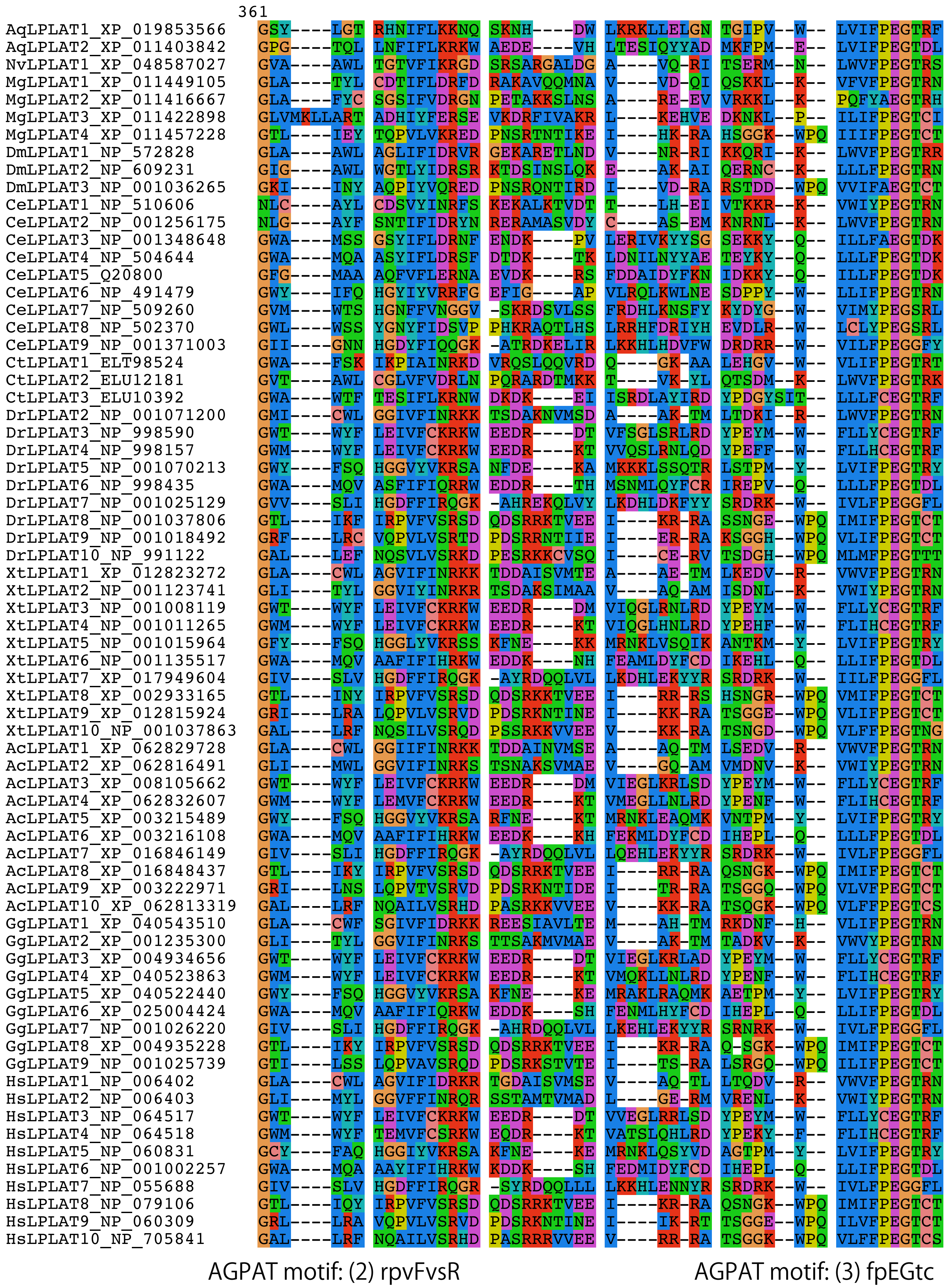
361
AqLPLAT1_XP_019853566 GSY----LGT RHNIFLKKNQ SKNH----DW LKRKLLELGN TGIPV--W-- LVIFPEGTRF
AqLPLAT2_XP_011403842 GPG----TQL LNFIFLKRKW AEDE----VH LTESIQYYAD MKFPM--E-- LVIFPEGTDL
NvLPLAT1_XP_048587027 GVA----AWL TGTVFIKRGD SRSARGALDG A----VQ-RI TSERM--N-- LWVFPEGTRS
MgLPLAT1_XP_011449105 GLA----TYL CDTIFLDRFD RAKAVQQMNA V----VD-QI QSKKL--K-- VFVFPEGTRN
MgLPLAT2_XP_011416667 GLA----FYC SGSIFVDRGN PETAKKSLNS A----RE-EV VRKKL--K-- PQFYAEGTRH
MgLPLAT3_XP_011422898 GLVMKLLART ADHIYFERSE VKDRFIVAKR L----KEHVE DKNKL--P-- ILIFPEGTCI
MgLPLAT4_XP_011457228 GTL----IEY TQPVLVKRED PNSRTNTIKE I----HK-RA HSGGK--WPQ IIIFPEGTCT
DmLPLAT1_NP_572828 GLA----AWL AGLIFIDRVR GEKARETLND V----NR-RI KKQRI--K-- LWVFPEGTRR
DmLPLAT2_NP_609231 GIG----AWL WGTLYIDRSR KTDSINSLQK E----AK-AI QERNC--K-- LLLFPEGTRN
DmLPLAT3_NP_001036265 GKI----INY AQPIYVQRED PNSRQNTIRD I----VD-RA RSTDD--WPQ VVIFAEGTCT
CeLPLAT1_NP_510606 NLC----AYL CDSVYINRFS KEKALKTVDT T----LH-EI VTKKR--K-- VWIYPEGTRN
CeLPLAT2_NP_001256175 NLG----AYF SNTIFIDRYN RERAMASVDY C----AS-EM KNRNL--K-- LWVFPEGTRN
CeLPLAT3_NP_001348648 GWA----MSS GSYIFLDRNF ENDK----PV LERIVKYYSG SEKKY--Q-- ILLFAEGTDK
CeLPLAT4_NP_504644 GWA----MQA ASYIFLDRSF DTDK----TK LDNILNYYAE TEYKY--Q-- LLLFPEGTDK
CeLPLAT5_Q20800 GFG----MAA AQFVFLERNA EVDK----RS FDDAIDYFKN IDKKY--Q-- ILLFPEGTDK
CeLPLAT6_NP_491479 GWY----IFQ HGYIYVRRFG EFIG----AP VLRQLKWLNE SDPPY--W-- LLIFPEGTRN
CeLPLAT7_NP_509260 GVM----WTS HGNFFVNGGV -SKRDSVLSS FRDHLKNSFY KYDYG--W-- VIMYPEGSRL
CeLPLAT8_NP_502370 GWL----WSS YGNYFIDSVP PHKRAQTLHS LRRHFDRIYH EVDLR--W-- LCLYPEGSRL
CeLPLAT9_NP_001371003 GII----GNN HGDYFIQQGK -ATRDKELIR LKKHLHDVFW DRDRR--W-- VILFPEGGFY
CtLPLAT1_ELT98524 GWA----FSK IKPIAINRKD VRQSLQQVRD Q----GK-AA LEHGV--W-- VLIFPEGTRT
CtLPLAT2_ELU12181 GVT----AWL CGLVFVDRLN PQRARDTMKK T----VK-YL QTSDM--K-- LWVFPEGTRK
CtLPLAT3_ELU10392 GWA----WTF TESIFLKRNW DKDK----EI ISRDLAYIRD YPDGYSIT-- LLLFCEGTRF
DrLPLAT2_NP_001071200 GMI----CWL GGIVFINRKK TSDAKNVMSD A----AK-TM LTDKI--R-- LWVFPEGTRN
DrLPLAT3_NP_998590 GWT----WYF LEIVFCKRKW EEDR----DT VFSGLSRLRD YPEYM--W-- FLLYCEGTRF
DrLPLAT4_NP_998157 GWM----WYF LEIVFCKRKW EEDR----KT VVQSLRNLQD YPEFF--W-- FLLHCEGTRF
DrLPLAT5_NP_001070213 GWY----FSQ HGGVYVKRSA NFDE----KA MKKKLSSQTR LSTPM--Y-- LVIFPEGTRY
DrLPLAT6_NP_998435 GWA----MQV ASFIFIQRRW EDDR----TH MSNMLQYFCR IREPV--Q-- LLLFPEGTDL
DrLPLAT7_NP_001025129 GVV----SLI HGDFFIRQGK -AHREKQLVY LKDHLDKFYY SRDRK--W-- IVLFPEGGFL
DrLPLAT8_NP_001037806 GTL----IKF IRPVFVSRSD QDSRRKTVEE I----KR-RA SSNGE--WPQ IMIFPEGTCT
DrLPLAT9_NP_001018492 GRF----LRC VQPVLVSRTD PDSRRNTIIE I----ER-RA KSGGH--WPQ VLIFPEGTCT
DrLPLAT10_NP_991122 GAL----LEF NQSVLVSRKD PESRKKCVSQ I----CE-RV TSDGH--WPQ MLMFPEGTTT
XtLPLAT1_XP_012823272 GLA----CWL AGVIFINRKK TDDAISVMTE A----AE-TM LKEDV--R-- VWVFPEGTRN
XtLPLAT2_NP_001123741 GLI----TYL GGVIYINRKR TSDAKSIMAA V----AQ-AM ISDNL--K-- VWIYPEGTRN
XtLPLAT3_NP_001008119 GWT----WYF LEIVFCKRKW EEDR----DM VIQGLRNLRD YPEYM--W-- FLLYCEGTRF
XtLPLAT4_NP_001011265 GWM----WYF LEIVFCKRKW EEDR----KT VIQGLHNLRD YPEHF--W-- FLIHCEGTRF
XtLPLAT5_NP_001015964 GFY----FSQ HGGLYVKRSS KFNE----KK MRNKLVSQIK ANTKM--Y-- LVIFPEGTRY
XtLPLAT6_NP_001135517 GWA----MQV AAFIFIHRKW EDDK----NH FEAMLDYFCD IKEHL--Q-- LLIFPEGTDL
XtLPLAT7_XP_017949604 GIV----SLV HGDFFIRQGK -AYRDQQLVL LKDHLEKYYR SRDRK--W-- IILFPEGGFL
XtLPLAT8_XP_002933165 GTL----INY IRPVFVSRSD QDSRKKTVEE I----RR-RS HSNGR--WPQ VMIFPEGTCT
XtLPLAT9_XP_012815924 GRI----LRA LQPVLVSRVD PDSRKNTINE I----KK-RA TSGGE--WPQ VLIFPEGTCT
XtLPLAT10_NP_001037863 GAL----LRF NQSILVSRQD PSSRKKVVEE V----KK-RA TSNGD--WPQ VLFFPEGTNG
AcLPLAT1_XP_062829728 GLA----CWL GGIIFINRKK TDDAINVMSE A----AQ-TM LSEDV--R-- VWVFPEGTRN
AcLPLAT2_XP_062816491 GLI----MWL GGVIFINRKS TSNAKSVMAE V----GQ-AM VMDNV--K-- VWIYPEGTRN
AcLPLAT3_XP_008105662 GWT----WYF LEIVFCKRKW EEDR----DM VIEGLKRLSD YPEYM--W-- FLLYCEGTRF
AcLPLAT4_XP_062832607 GWM----WYF LEMVFCKRKW EEDR----KT VMEGLLNLRD YPENF--W-- FLIHCEGTRF
AcLPLAT5_XP_003215489 GWY----FSQ HGGVYVKRSA RFNE----KT MRNKLEAQMK VNTPM--Y-- LVIFPEGTRY
AcLPLAT6_XP_003216108 GWA----MQV AAFIFIHRKW EEDK----KH FEKMLDYFCD IHEPL--Q-- LLLFPEGTDL
AcLPLAT7_XP_016846149 GIV----SLI HGDFFIRQGK -AYRDQQLVL LQEHLEKYYR SRDRK--W-- IVLFPEGGFL
AcLPLAT8_XP_016848437 GTL----IKY IRPVFVSRSD QDSRRKTVEE I----RR-RA QSNGK--WPQ IMIFPEGTCT
AcLPLAT9_XP_003222971 GRI----LNS LQPVTVSRVD PDSRKNTIDE I----TR-RA TSGGQ--WPQ VLVFPEGTCT
AcLPLAT10_XP_062813319 GAL----LRF NQAILVSRHD PASRKKVVEE V----KK-RA TSQGK--WPQ VLFFPEGTCS
GgLPLAT1_XP_040543510 GLA----CWF SGIVFIDRKK REESIAVLTE M----AH-TM RKDNF--H-- VLIFPEGTRN
GgLPLAT2_XP_001235300 GLI----TYL GGVIFINRKS TTSAKMVMAE V----AK-TM TADKV--K-- VWVYPEGTRN
GgLPLAT3_XP_004934656 GWT----WYF LEIVFCKRKW EEDR----DT VIEGLKRLAD YPEYM--W-- FLLYCEGTRF
GgLPLAT4_XP_040523863 GWM----WYF LEIVFCKRKW EEDR----KT VMQKLLNLRD YPENF--W-- FLIHCEGTRF
GgLPLAT5_XP_040522440 GWY----FSQ HGGVYVKRSA KFNE----KE MRAKLRAQMK AETPM--Y-- LVIFPEGTRY
GgLPLAT6_XP_025004424 GWA----MQV AAFIFIQRKW EDDK----SH FENMLHYFCD IHEPL--Q-- LLIFPEGTDL
GgLPLAT7_NP_001026220 GIV----SLI HGDFFIRQGK -AHRDQQLVL LKEHLEKYYR SRNRK--W-- IVLFPEGGFL
GgLPLAT8_XP_004935228 GTL----IKY IRPVFVSRSD QDSRRKTVEE I----KR-RA Q-SGK--WPQ IMIFPEGTCT
GgLPLAT9_NP_001025739 GTI----LSS LQPVAVSRQD PDSRKSTVTE I----TS-RA LSRGQ--WPQ ILIFPEGTCT
HsLPLAT1_NP_006402 GLA----CWL AGVIFIDRKR TGDAISVMSE V----AQ-TL LTQDV--R-- VWVFPEGTRN
HsLPLAT2_NP_006403 GLI----MYL GGVFFINRQR SSTAMTVMAD L----GE-RM VRENL--K-- VWIYPEGTRN
HsLPLAT3_NP_064517 GWT----WYF LEIVFCKRKW EEDR----DT VVEGLRRLSD YPEYM--W-- FLLYCEGTRF
HsLPLAT4_NP_064518 GWM----WYF TEMVFCSRKW EQDR----KT VATSLQHLRD YPEKY--F-- FLIHCEGTRF
HsLPLAT5_NP_060831 GCY----FAQ HGGIYVKRSA KFNE----KE MRNKLQSYVD AGTPM--Y-- LVIFPEGTRY
HsLPLAT6_NP_001002257 GWA----MQA AAYIFIHRKW KDDK----SH FEDMIDYFCD IHEPL--Q-- LLIFPEGTDL
HsLPLAT7_NP_055688 GIV----SLV HGDFFIRQGR -SYRDQQLLL LKKHLENNYR SRDRK--W-- IVLFPEGGFL
HsLPLAT8_NP_079106 GTL----IQY IRPVFVSRSD QDSRRKTVEE I----KR-RA QSNGK--WPQ IMIFPEGTCT
HsLPLAT9_NP_060309 GRL----LRA VQPVLVSRVD PDSRKNTINE I----IK-RT TSGGE--WPQ ILVFPEGTCT
HsLPLAT10_NP_705841 GAL----LRF NQAILVSRHD PASRRRVVEE V----RR-RA TSGGK--WPQ VLFFPEGTCS
AGPAT motif: (2) rpvFvsR
AGPAT motif: (3) fpEGtc

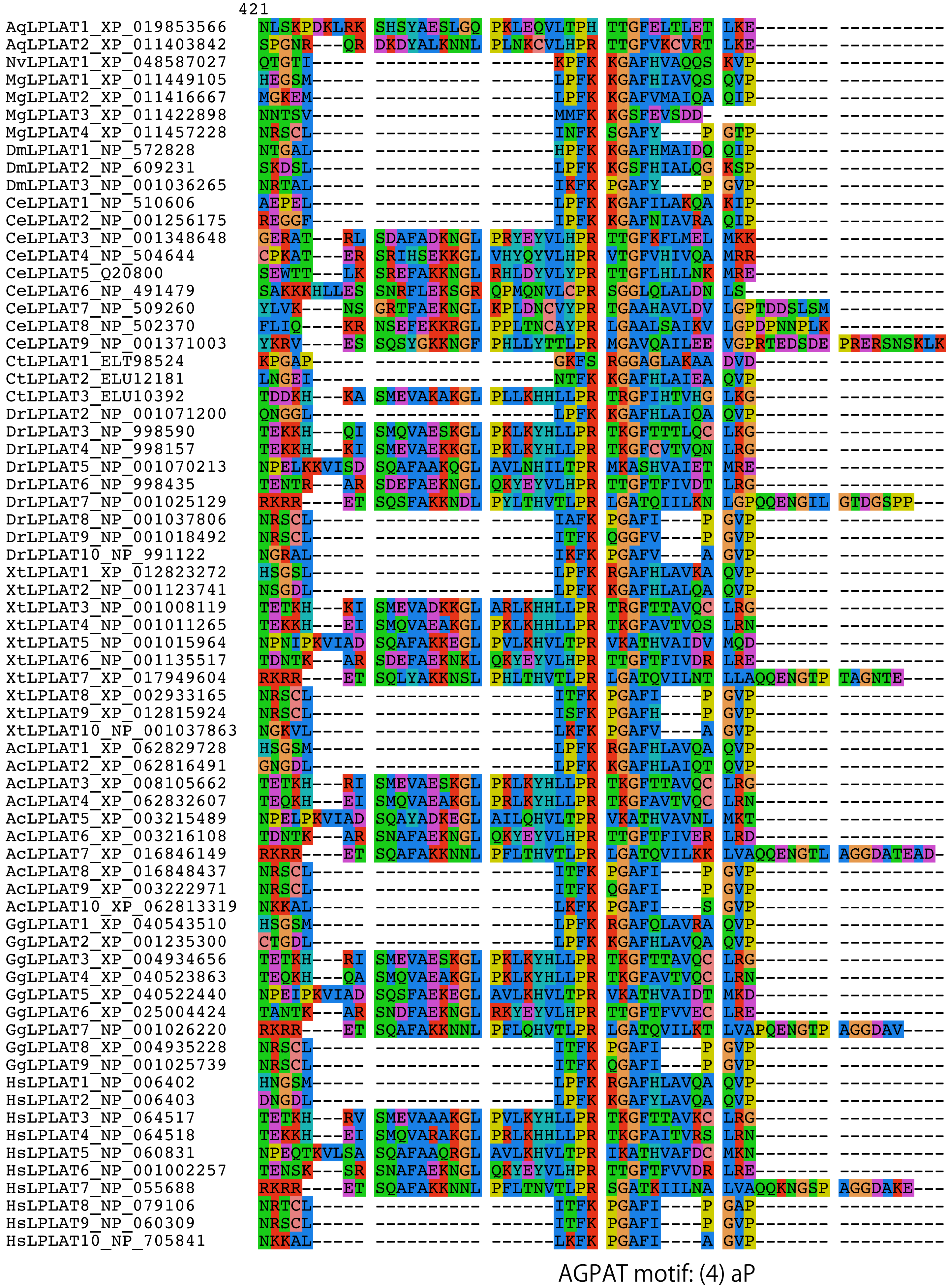
421
AqLPLAT1_XP_019853566 NLSKPDKLRK SHSYAESLGQ PKLEQVLTPH TTGFELTLET LKE------- ----------
AqLPLAT2_XP_011403842 SPGNR---QR DKDYALKNNL PLNKCVLHPR TTGFVKCVRT LKE------- ----------
NvLPLAT1_XP_048587027 QTGTI----- ---------- ------KPFK KGAFHVAQQS KVP------- ----------
MgLPLAT1_XP_011449105 HEGSM----- ---------- ------LPFK KGAFHIAVQS QVP------- ----------
MgLPLAT2_XP_011416667 MGKEM----- ---------- ------LPFK KGAFVMAIQA QIP------- ----------
MgLPLAT3_XP_011422898 NNTSV----- ---------- ------MMFK KGSFEVSDD- ---------- ----------
MgLPLAT4_XP_011457228 NRSCL----- ---------- ------INFK SGAFY----P GTP------- ----------
DmLPLAT1_NP_572828 NTGAL----- ---------- ------HPFK KGAFHMAIDQ QIP------- ----------
DmLPLAT2_NP_609231 SKDSL----- ---------- ------LPFK KGSFHIALQG KSP------- ----------
DmLPLAT3_NP_001036265 NRTAL----- ---------- ------IKFK PGAFY----P GVP------- ----------
CeLPLAT1_NP_510606 AEPEL----- ---------- ------LPFK KGAFILAKQA KIP------- ----------
CeLPLAT2_NP_001256175 REGGF----- ---------- ------IPFK KGAFNIAVRA QIP------- ----------
CeLPLAT3_NP_001348648 GERAT---RL SDAFADKNGL PRYEYVLHPR TTGFKFLMEL MKK------- ----------
CeLPLAT4_NP_504644 CPKAT---ER SRIHSEKKGL VHYQYVLHPR VTGFVHIVQA MRR------- ----------
CeLPLAT5_Q20800 SEWTT---LK SREFAKKNGL RHLDYVLYPR TTGFLHLLNK MRE------- ----------
CeLPLAT6_NP_491479 SAKKKHLLES SNRFLEKSGR QPMQNVLCPR SGGLQLALDN LS-------- ----------
CeLPLAT7_NP_509260 YLVK----NS GRTFAEKNGL KPLDNCVYPR TGAAHAVLDV LGPTDDSLSM ----------
CeLPLAT8_NP_502370 FLIQ----KR NSEFEKKRGL PPLTNCAYPR LGAALSAIKV LGPDPNNPLK ----------
CeLPLAT9_NP_001371003 YKRV----ES SQSYGKKNGF PHLLYTTLPR MGAVQAILEE VGPRTEDSDE PRERSNSKLK
CtLPLAT1_ELT98524 KPGAP----- ---------- ------GKFS RGGAGLAKAA DVD------- ----------
CtLPLAT2_ELU12181 LNGEI----- ---------- ------NTFK KGAFHLAIEA QVP------- ----------
CtLPLAT3_ELU10392 TDDKH---KA SMEVAKAKGL PLLKHHLLPR TRGFIHTVHG LKG------- ----------
DrLPLAT2_NP_001071200 QNGGL----- ---------- ------LPFK KGAFHLAIQA QVP------- ----------
DrLPLAT3_NP_998590 TEKKH---QI SMQVAESKGL PKLKYHLLPR TKGFTTTLQC LKG------- ----------
DrLPLAT4_NP_998157 TEKKH---KI SMEVAEKKGL PKLKYHLLPR TKGFCVTVQN LRG------- ----------
DrLPLAT5_NP_001070213 NPELKKVISD SQAFAAKQGL AVLNHILTPR MKASHVAIET MRE------- ----------
DrLPLAT6_NP_998435 TENTR---AR SDEFAEKNGL QKYEYVLHPR TTGFTFIVDT LRG------- ----------
DrLPLAT7_NP_001025129 RKRR----ET SQSFAKKNDL PYLTHVTLPR LGATQIILKN LGPQQENGIL GTDGSPP---
DrLPLAT8_NP_001037806 NRSCL----- ---------- ------IAFK PGAFI----P GVP------- ----------
DrLPLAT9_NP_001018492 NRSCL----- ---------- ------ITFK QGGFV----P GVP------- ----------
DrLPLAT10_NP_991122 NGRAL----- ---------- ------IKFK PGAFV----A GVP------- ----------
XtLPLAT1_XP_012823272 HSGSL----- ---------- ------LPFK RGAFHLAVKA QVP------- ----------
XtLPLAT2_NP_001123741 NSGDL----- ---------- ------LPFK KGAFHLALQA QVP------- ----------
XtLPLAT3_NP_001008119 TETKH---KI SMEVADKKGL ARLKHHLLPR TRGFTTAVQC LRG------- ----------
XtLPLAT4_NP_001011265 TEKKH---EI SMQVAEAKGL PKLKHHLLPR TKGFAVTVQS LRN------- ----------
XtLPLAT5_NP_001015964 NPNIPKVIAD SQAFAKKEGL PVLKHVLTPR VKATHVAIDV MQD------- ----------
XtLPLAT6_NP_001135517 TDNTK---AR SDEFAEKNKL QKYEYVLHPR TTGFTFIVDR LRE------- ----------
XtLPLAT7_XP_017949604 RKRR----ET SQLYAKKNSL PHLTHVTLPR LGATQVILNT LLAQQENGTP TAGNTE----
XtLPLAT8_XP_002933165 NRSCL----- ---------- ------ITFK PGAFI----P GVP------- ----------
XtLPLAT9_XP_012815924 NRSCL----- ---------- ------ISFK PGAFH----P GVP------- ----------
XtLPLAT10_NP_001037863 NGKVL----- ---------- ------LKFK PGAFV----A GVP------- ----------
AcLPLAT1_XP_062829728 HSGSM----- ---------- ------LPFK RGAFHLAVQA QVP------- ----------
AcLPLAT2_XP_062816491 GNGDL----- ---------- ------LPFK KGAFHLAIQT QVP------- ----------
AcLPLAT3_XP_008105662 TETKH---RI SMEVAESKGL PKLKYHLLPR TKGFTTAVQC LRG------- ----------
AcLPLAT4_XP_062832607 TEQKH---EI SMQVAEAKGL PRLKYHLLPR TKGFAVTVQC LRN------- ----------
AcLPLAT5_XP_003215489 NPELPKVIAD SQAYADKEGL AILQHVLTPR VKATHVAVNL MKT------- ----------
AcLPLAT6_XP_003216108 TDNTK---AR SNAFAEKNGL QKYEYVLHPR TTGFTFIVER LRD------- ----------
AcLPLAT7_XP_016846149 RKRR----ET SQAFAKKNNL PFLTHVTLPR LGATQVILKK LVAQQENGTL AGGDATEAD-
AcLPLAT8_XP_016848437 NRSCL----- ---------- ------ITFK PGAFI----P GVP------- ----------
AcLPLAT9_XP_003222971 NRSCL----- ---------- ------ITFK QGAFI----P GVP------- ----------
AcLPLAT10_XP_062813319 NKKAL----- ---------- ------LKFK PGAFI----S GVP------- ----------
GgLPLAT1_XP_040543510 HSGSM----- ---------- ------LPFK RGAFQLAVRA QVP------- ----------
GgLPLAT2_XP_001235300 CTGDL----- ---------- ------LPFK KGAFHLAVQA QVP------- ----------
GgLPLAT3_XP_004934656 TETKH---RI SMEVAESKGL PKLKYHLLPR TKGFTTAVQC LRG------- ----------
GgLPLAT4_XP_040523863 TEQKH---QA SMQVAEAKGL PKLKYHLLPR TKGFAVTVQC LRN------- ----------
GgLPLAT5_XP_040522440 NPEIPKVIAD SQSFAEKEGL AVLKHVLTPR VKATHVAIDT MKD------- ----------
GgLPLAT6_XP_025004424 TANTK---AR SNDFAEKNGL RKYEYVLHPR TTGFTFVVEC LRE------- ----------
GgLPLAT7_NP_001026220 RKRR----ET SQAFAKKNNL PFLQHVTLPR LGATQVILKT LVAPQENGTP AGGDAV----
GgLPLAT8_XP_004935228 NRSCL----- ---------- ------ITFK PGAFI----P GVP------- ----------
GgLPLAT9_NP_001025739 NRSCL----- ---------- ------ITFK QGAFI----P GVP------- ----------
HsLPLAT1_NP_006402 HNGSM----- ---------- ------LPFK RGAFHLAVQA QVP------- ----------
HsLPLAT2_NP_006403 DNGDL----- ---------- ------LPFK KGAFYLAVQA QVP------- ----------
HsLPLAT3_NP_064517 TETKH---RV SMEVAAAKGL PVLKYHLLPR TKGFTTAVKC LRG------- ----------
HsLPLAT4_NP_064518 TEKKH---EI SMQVARAKGL PRLKHHLLPR TKGFAITVRS LRN------- ----------
HsLPLAT5_NP_060831 NPEQTKVLSA SQAFAAQRGL AVLKHVLTPR IKATHVAFDC MKN------- ----------
HsLPLAT6_NP_001002257 TENSK---SR SNAFAEKNGL QKYEYVLHPR TTGFTFVVDR LRE------- ----------
HsLPLAT7_NP_055688 RKRR----ET SQAFAKKNNL PFLTNVTLPR SGATKIILNA LVAQQKNGSP AGGDAKE---
HsLPLAT8_NP_079106 NRTCL----- ---------- ------ITFK PGAFI----P GAP------- ----------
HsLPLAT9_NP_060309 NRSCL----- ---------- ------ITFK PGAFI----P GVP------- ----------
HsLPLAT10_NP_705841 NKKAL----- ---------- ------LKFK PGAFI----A GVP------- ----------
AGPAT motif: (4) aP

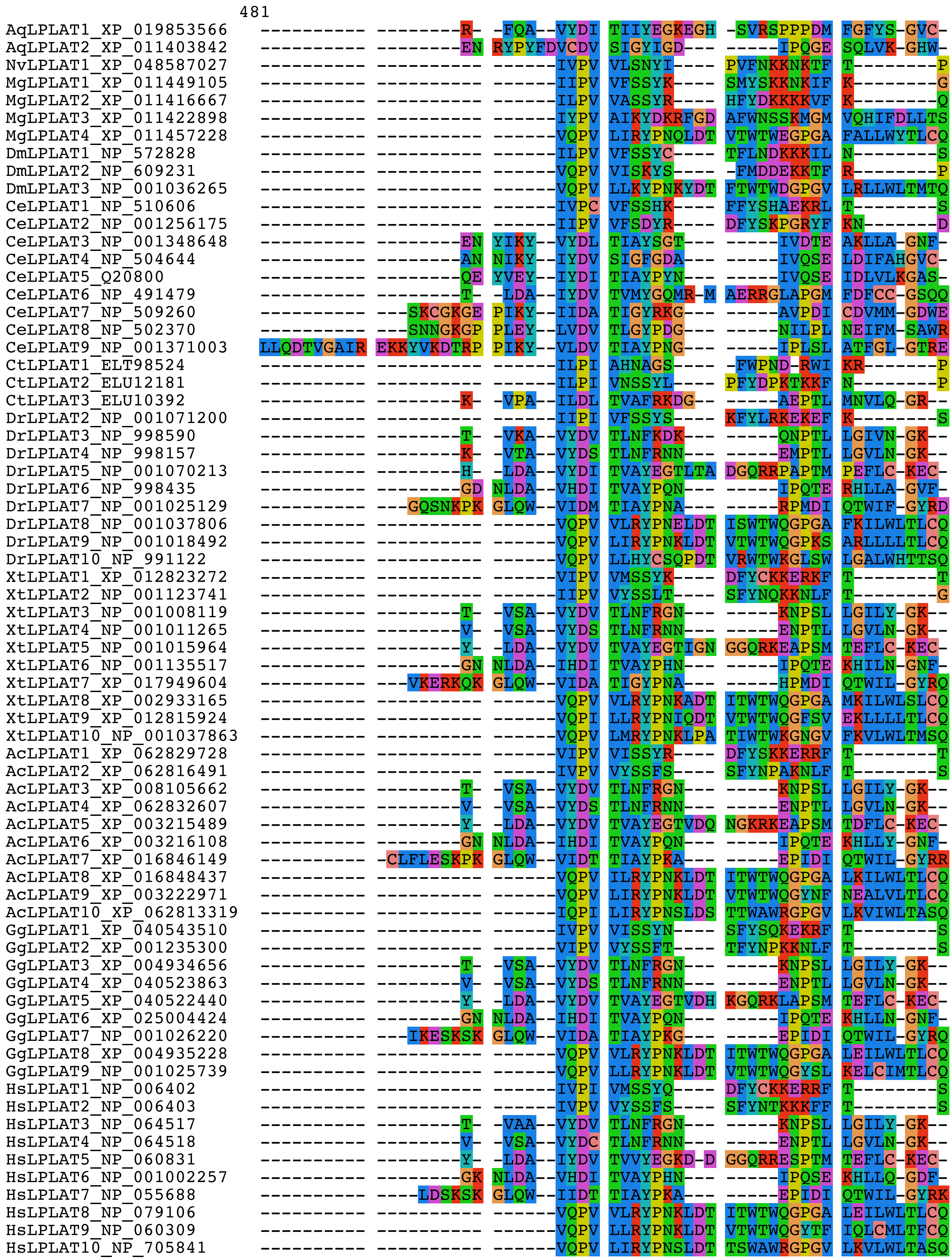

AGPAT motif: (4) vqPvv

```
                           541
AqLPLAT1_XP_019853566      ----QKVHIH IKRTEVSDIP NT-VEEQRQW LFDSFKDK-- -DKLITDFIS SGI-------
AqLPLAT2_XP_011403842      ---PKEIHFH IKYHPSSSLP SS-DEELGEW LKKRWREK-- -DELLTQFYE TNS-------
NvLPLAT1_XP_048587027      G--IIKVTIL PEVPTDGLTP SD-VTSLTER VWHDMLKV-- FHEGQPIPNN MDQDKPKEQ-
MgLPLAT1_XP_011449105      G--KIVITCL PPVSTSGLSK DN-IPELLES VRNSMIET-- YNRTSDVKDK SE--------
MgLPLAT2_XP_011416667      G--TVIVTCL PPIPTTGLTM SD-LNDLIDR VWKQMQET-- IDNTTEEVEN SL--------
MgLPLAT3_XP_011422898      WALVAEVWYL PPMHRNEN-- ES-AVDFANR VKKEIARQGG LVDLSWDGQL ----KRMKVK
MgLPLAT4_XP_011457228      FRTNVEIEFL PVYTPSEEEK KN-AALFASN VRAKMAEALG VPVTDHSYDD -CRLMQKAAK
DmLPLAT1_NP_572828         G--RIVITTL PPVSTEGLTK DD-IDVLMER VRSQMIET-- FKVTSAEALH RYKPIKKVGI
DmLPLAT2_NP_609231         G--HALIHIL PEVSTEKYKR ED-VQLLIDE CQSIMQTE-- YTKLSKEGQA LSSKKQA---
DmLPLAT3_NP_001036265      FYNRCEIEYL PVYTPSEDEV AD-ANLYANN VREVMAKALG VPTSDYSFED -VIVMSRARD
CeLPLAT1_NP_510606         G--NCIIDIL PEVDSSKF-- DS-IDDLSAH CRKIMQAH-- REKLDAEAAN LNI-------
CeLPLAT2_NP_001256175      G--EVVIRVL DAIPTKGLTL DD-VSELSDM CRDVMLAA-- YKEVTLEAQQ RNATRRGETK
CeLPLAT3_NP_001348648      ---PDKVHLD VKKYKLDEIP TG--EGCEKW LTDLWATK-- -EKRLKKFYE QEERLEASGD
CeLPLAT4_NP_504644         ---PKEVFYQ VIKYPIEAIP QT-DEALGQW LVNLWRNK-- -EEKLKRFYE MPRNVRQ---
CeLPLAT5_Q20800            ---PREVHFH IRKIPISQVP LN-EQDASRW LTDRWTIK-- -EQLLHDFYS EEQPINRQ--
CeLPLAT6_NP_491479         ---FKQLHIH LDRIPIDEVP KA-KLELRTW TIERFTKK-- -ERIIDEFYS EKPST-GS--
CeLPLAT7_NP_509260         SVEASQFAVH YDVIPVKPE- WSDENLLKEF LYERYIIK-- -DKLLAEFYK TGH-------
CeLPLAT8_NP_502370         NGGGKPFAIH YEIFKMDPK- WKDEEELKNF LFDRYQKK-- -DKLLESYYK TGS-------
CeLPLAT9_NP_001371003      ---KCDIAVN YKIYDAAEVP FEDEEKLRDW MYEVYKEK-- -DEMLARYYS TGE-------
CtLPLAT1_ELT98524          G--TIQIEIG ATIKTKNLSV AE-ANDATRN WIASSLHA-- MN-------- ----------
CtLPLAT2_ELU12181          G--KIITVCL PAISTEGMTS DD-VNQVTED TRQKMAVV-- FEKISREVAQ EIGSKET---
CtLPLAT3_ELU10392          ---ACKAEMY CRRIPLDSVP TNTDQECADW VQKVFREK-- -DEVYDDFYR NGK-------
DrLPLAT2_NP_001071200      G--TITLKVL PKIETKGLTA DD-VTTLSDQ AFGVMRSA-- FMEISGQSAQ SNGPSTH---
DrLPLAT3_NP_998590         ---KYKADLS VRRFSVEEIP DD-EKECADW LHKLYQEK-- -DALQEYYEK EGC-------
DrLPLAT4_NP_998157         ---KYHADLY VRRIPLDSIP ED-ESECAAW LHKLYQEK-- -DEFQEHYRQ TGR-------
DrLPLAT5_NP_001070213      ----PRVHIH ISRVDIKDIP AE-PDVFRGW LHQRFEIK-- -DKLLTTFYE SEDPAKKS--
DrLPLAT6_NP_998435         ---PREIHFH VQRFTVASVP AG-AAGLQAW CQERWREK-- -ERRLQRFYE TVPRRFDAPA
DrLPLAT7_NP_001025129      ---PTVTHVH YRTYPIKDVP VDS-EALTDW LYQRFVEK-- -EKLLAHFYE TGA-------
DrLPLAT8_NP_001037806      LHNFVEIEYL PTYTPSEEEK KD-PALFASN VRRIMAKALG LPIIDYSFED -CQLAMAKGP
DrLPLAT9_NP_001018492      LCTTVEVEFL PPQVPTEMEK KC-PLKFAQS VRAVMAKSLK LPVTDHTYED -CRLMIAAGE
DrLPLAT10_NP_991122        IYSSITVEFL PVYTPSAEEK QN-PELYAEN VQKLMARALG VPATDYMM-E -GFPVQKLGN
XtLPLAT1_XP_012823272      G--KCTVRIL PGVHTKGLSS DD-VPELADR VRGMMLEA-- FSQMSSERPG KG-----PGG
XtLPLAT2_NP_001123741      G--TVKVEIL PKIDTSGLTE ND-ITDLTER CHQTMRQV-- FFRLSNKPYE SNGQSNCAQ-
XtLPLAT3_NP_001008119      ---KYEADMC VRRFPLEEIP ED-EQKAADW LHKLYQEK-- -DALQEQYIQ EGT-------
XtLPLAT4_NP_001011265      ---KYHADLC VRRIPLEEIP ED-EQECASW LHKLYQEK-- -DAFQEEYYK TGA-------
XtLPLAT5_NP_001015964      ----PKIHIL LERISVKDIP KE-QTFMRRW LHERFEVK-- -DKVLTEFYD STDPAKKN--
XtLPLAT6_NP_001135517      ---PKEIHFH VCRYPVSSLP VS-KEELQLW CQQRWKEK-- -EDRLRAFYQ GER-------
XtLPLAT7_XP_017949604      ---PTVTHVY YRIYPIKDVP MET-EALTDW LYQRFVEK-- -EDLLAHFYE TGA-------
XtLPLAT8_XP_002933165      FHNYVEIEFL PIYTPSEEEK KN-PALYANN VRRVMAKALG VSVTDYTFED -CQLALAEGQ
XtLPLAT9_XP_012815924      ICTNVTVEFL PVYVPSEEEK KD-PFLYAHN VRHTMAQALG LPVTDHTYED -CRLMMTAGE
XtLPLAT10_NP_001037863     FYINLEIEFL PVYHPTAEER AD-PTLYASK VQKIMADALA KPATEFELIG -DTPVTPVGH
AcLPLAT1_XP_062829728      G--RCTVEIL PPMPTKGLSA DD-VPALTER VRQAMLVA-- FDGLSVELGA PQ--------
AcLPLAT2_XP_062816491      G--KIRVEVL PAIPTKGLGP SD-VDDLTSQ CYDTMRET-- LFRLSGRPGE ANGGAPPRVP
AcLPLAT3_XP_008105662      ---KYEADMC VRRFPLEDIP LD-EKEAAKW LHTLYQEK-- -DALQEKYNQ EGI-------
AcLPLAT4_XP_062832607      ---KYHADLY VRRIPLGEVP ED-EQECSNW LHKLYQEK-- -DSFQQEYYK TGV-------
AcLPLAT5_XP_003215489      ----PRIHIY VDRIELKDIP EE-QMYMRRW LHERFEIK-- -DKLLIEFYD SKDPKRRN--
AcLPLAT6_XP_003216108      ---PKEIHFH VQRYPVEVLP AS-REELQLW CQQRWEEK-- -EERLRLFYE GKK-------
AcLPLAT7_XP_016846149      ---PTITHVH YRIFPIKDVP LEP-EALTAW LYQRFIEK-- -EDLLSHFYK TGA-------
AcLPLAT8_XP_016848437      FHNFVEIEFL PVYIPSEEER KN-PSLYANN VRRIMAKALG VSVTDYTFED -CQLALAEGQ
AcLPLAT9_XP_003222971      LFTKMEVEFL PVHIPTDEEK HD-PALFANR VRSKMSSALG VPVTDHTYED -CRLMISAGN
AcLPLAT10_XP_062813319     PCTTVEVEFL PVYHPSAEEA VN-PTLYANN VQKVMAKALG IPATECEFLG -NLPVTVVGR
GgLPLAT1_XP_040543510      G--TSTIQIL PPVETVGLSS DD-VPKLTEQ VRDSMMNA-- YQRISGMTNG GSHE------
GgLPLAT2_XP_001235300      G--KIKVEVL PPIETKGLTS DD-VSDLTDR CYNLMRET-- LFRLSGRPGE GKDSS-----
GgLPLAT3_XP_004934656      ---KYEADMC VRRFPLEDIP QD-EKEAANW LHKLYQEK-- -DALQEMYNQ EGT-------
GgLPLAT4_XP_040523863      ---KYHADLY VRRIPLEEIP ED-EQECSNW LHRLYQEK-- -DAFQEEYYR TGT-------
GgLPLAT5_XP_040522440      ----PRVHIF IDRIELKDIP EE-QMYMRRW LHERFEIK-- -DKLLIEFYD AKDSKRRN--
GgLPLAT6_XP_025004424      ---PKEIHFH VQRYPIETVP TS-KEELQLW CQKRWEEK-- -EERLRRFYE GGK-------
GgLPLAT7_NP_001026220      ---PTVTHVH YRIFPIKDVP AEP-EALSQW LYQRFIEK-- -EDLLTHFYE TGA-------
GgLPLAT8_XP_004935228      FHNSVEIEFL PVYIPSEEER KN-PVLYANN VRRVMAEALG VPVTDYTFED -CQLALAEGQ
GgLPLAT9_NP_001025739      LFTRVEVEFL PVYVPTEEER ND-PILFANR VRQTMATALK VPVTDHTFED -CRLMISAGQ
HsLPLAT1_NP_006402         G--QCQVRVL PPVPTEGLTP DD-VPALADR VRHSMLTV-- FREISTDGRG GGDYLKKPGG
HsLPLAT2_NP_006403         G--TVTVQVL EAIPTSGLTA AD-VPALVDT CHRAMRTT-- FLHISKTPQE NGATAGSGVQ
HsLPLAT3_NP_064517         ---KYEADMC VRRFPLEDIP LD-EKEAAQW LHKLYQEK-- -DALQEIYNQ KGM-------
HsLPLAT4_NP_064518         ---KYHADLY VRRIPLEDIP ED-DDECSAW LHKLYQEK-- -DAFQEEYYR TGT-------
HsLPLAT5_NP_060831         ----PKIHIH IDRIDKKDVP EE-QEHMRRW LHERFEIK-- -DKMLIEFYE SPDPERRK--
HsLPLAT6_NP_001002257      ---PREIHFH VHRYPIDTLP TS-KEDLQLW CHKRWEEK-- -EERLRSFYQ GEK-------
HsLPLAT7_NP_055688         ---PTVTHVH YRIFPIKDVP LET-DDLTTW LYQRFVEK-- -EDLLSHFYE TGA-------
HsLPLAT8_NP_079106         FHNQVEIEFL PVYSPSEEEK RN-PALYASN VRRVMAEALG VSVTDYTFED -CQLALAEGQ
HsLPLAT9_NP_060309         LFTKVEVEFM PVQVPNDEEK ND-PVLFANK VRNLMAEALG IPVTDHTYED -CRLMISAGQ
HsLPLAT10_NP_705841        PCSIVDVEFL PVYHPSPEES RD-PTLYANN VQRVMAQALG IPATECEFVG -SLPVIVVGR
```

```
                           601
AqLPLAT1_XP_019853566     ---------V RGQT-----R SLPLPLSYT- ---------L PPV-MAYGSV LLLMAT----
AqLPLAT2_XP_011403842     ---------F PGPV----LR ETLMSRLKIL ALLVGWLVLC TAVIYYWIQ- ATLC--MTII
NvLPLAT1_XP_048587027     ---------- ---------- ---------- ---------- ---------- ----------
MgLPLAT1_XP_011449105     ---------- ---------- ---------- ---------- ---------- ----------
MgLPLAT2_XP_011416667     ---------- ---------- ---------- ---------- ---------- ----------
MgLPLAT3_XP_011422898     -------DSW KTKT------ -----QD-D- -----FSKIL K--------V D---------
MgLPLAT4_XP_011457228     -------LKL PKSA------ -----GIVE- -----FMKLN KKLGLKFDDA N---------
DmLPLAT1_NP_572828        -------PAA SSAS------ -----TSST- -----TTTTL ASPGTAVTPV A---------
DmLPLAT2_NP_609231        ---------- ---------- ---------- ---------- ---------- ----------
DmLPLAT3_NP_001036265     -------MKI PFPG------ -----DIVE- -----IERTI EKLGLNESQR D---------
CeLPLAT1_NP_510606        ---------- ---------- ---------- ---------- ---------- ----------
CeLPLAT2_NP_001256175     -------DGK KSE------- ---------- ---------- ---------- ----------
CeLPLAT3_NP_001348648     ---RF---EW PET-TTGIGY ---------- ---------Y VAF-AFWVLA SLIW--MGAI
CeLPLAT4_NP_504644        ---------F PDT-PDGVEY ELDNNTDRA- --------QK GLI-GFWCFT TVFW--MFMF
CeLPLAT5_Q20800           ---------F PVERGDGVWR S-WKEPRRHF ------YVKL TSL-MFWTLV ISFC--SYHI
CeLPLAT6_NP_491479        ---------- ---------A LPCVPISQT- ---------L PST-LFFSAA LLAPFF----
CeLPLAT7_NP_509260        ---------F PGDKTK---- --VI-PN-NY EMM-FAQVFW G------CLY ----Y-AHY-
CeLPLAT8_NP_502370        ---------F SSESKE---- --AQFPS-DM ILIGFHILIM GLF-ALLVIF ----L-LSF-
CeLPLAT9_NP_001371003     ---------F NPGEKG---- --TRI----- --AFSWAKII GMY-GFWFGS FYAQY-NVYS
CtLPLAT1_ELT98524         ---------- ---------- ---------- ---------- ---------- ----------
CtLPLAT2_ELU12181         ---------- ---------- ---------- ---------- ---------- ----------
CtLPLAT3_ELU10392         ---------F TRGVK----- --TEIPLRVN D-MVVW---- ----FMWTIV LCVPLYYYIV
DrLPLAT2_NP_001071200     ---------- ---------- ---------- ---------- ---------- ----------
DrLPLAT3_NP_998590        ---------Y P-GPT----- --IIPKRRVW T-LLNF---- ----LFWATI LLSPLINFAW
DrLPLAT4_NP_998157        ---------F P-GPI----- --TNPPRRLW A-LVNW---- ----LFWVCV LVYPICVLLL
DrLPLAT5_NP_001070213     --------RF PGEG-----R KSPLSLSKT- ---------L PSL-LILSGL TFPMLL----
DrLPLAT6_NP_998435        VGVCVREPCC QSGQ--CVCV PRCKSEGRVR ------IILV ASL-LYWSVF ITAA--CASL
DrLPLAT7_NP_001025129     ---------F PPLDGQ---- --KEMVSREM TLDNAWLFLV QTF-AFASGY --MWY-SILH
DrLPLAT8_NP_001037806     -------LRL PKHT------ -----CLLE- -----FARLV RLLGLKTKVT D---------
DrLPLAT9_NP_001018492     -------LTL PMEA------ -----GLVE- -----FTKIS RKLELKWDNV K---------
DrLPLAT10_NP_991122       -------LSL PLEP------ -----PAQE- -----TLRLL HQQGFSKSRV E---------
XtLPLAT1_XP_012823272     -------VH- ---------- ---------- ---------- ---------- ----------
XtLPLAT2_NP_001123741     ---------- ---------- ---------- ---------- ---------- ----------
XtLPLAT3_NP_001008119     ---------F P-GTQ----- --IVPPRRPW T-LLNF---- ----LFWATL LLSPLFSFAI
XtLPLAT4_NP_001011265     ---------Y P-GTA----- --VVPPRRPW T-LINW---- ----IFWASL LSYPLFKVLA
XtLPLAT5_NP_001015964     --------TF PGVG-----H SAKLDLKRT- ---------L PSF-LLLTGV TAGMLC----
XtLPLAT6_NP_001135517     --------YF DATR--RSRI PPCKSELRVH ------LIKM ASL-LYWTLF PLAM--IVLL
XtLPLAT7_XP_017949604     ---------F PPLKGQ---- --SKASSREM ALSNSWLFLV QSL-AFLSGY --MWY-CLLQ
XtLPLAT8_XP_002933165     -------LRL PSDT------ -----SLLE- -----FARLV RSLGLKPEKL E---------
XtLPLAT9_XP_012815924     -------LTL PMET------ -----GLVE- -----FTKIS KKLNLKWDSI K---------
XtLPLAT10_NP_001037863    -------LKV ALDP------ -----KIWE- -----LGNIL EKAGFSLDSV Q---------
AcLPLAT1_XP_062829728     ---------- ---------- ---------- ---------- ---------- ----------
AcLPLAT2_XP_062816491     -------Q-- ---------- ---------- ---------- ---------- ----------
AcLPLAT3_XP_008105662     ---------F P-GQQ----- --FKPPRRPW T-LLNF---- ----LFWATV LLSPLFKFGF
AcLPLAT4_XP_062832607     ---------Y P-GTP----- --IVPSRRPW T-LLNW---- ----LFWALL LLYPLFKLLI
AcLPLAT5_XP_003215489     --------KF PGKC-----V YSKLSLKKT- ---------L PSF-LFLSGL TVSMLL----
AcLPLAT6_XP_003216108     --------YF DASR--RSKI PPCKSELRVM ------VVKC ISL-LYWTFF TLAA--FVLL
AcLPLAT7_XP_016846149     ---------F PPIQGQ---- --TEVVSREM TLSNLWLVLV QSF-AFLSGG --MWY-CILQ
AcLPLAT8_XP_016848437     -------LRL PSNT------ -----CLLE- -----FAKLV RSLGLKPEKL E---------
AcLPLAT9_XP_003222971     -------LTL PMEA------ -----GLVE- -----FTKIS KKLNLKWDNI R---------
AcLPLAT10_XP_062813319    -------LRV ALEP------ -----RIWE- -----LGKVL RKARFLTGGA K---------
GgLPLAT1_XP_040543510     ---------- ---------- ---------- ---------- ---------- ----------
GgLPLAT2_XP_001235300     ---------- ---------- ---------- ---------- ---------- ----------
GgLPLAT3_XP_004934656     ---------F P-GQQ----- --FKPPRRPW T-LVNF---- ----LFWATV LLSPLFTFGF
GgLPLAT4_XP_040523863     ---------Y P-AVP----- --IVPPRRPW T-LLNW---- ----LFWALL LLYPLFKLLI
GgLPLAT5_XP_040522440     --------KF PGKS-----V HSKLSLKKT- ---------L PSL-LFLGGL TASMLL----
GgLPLAT6_XP_025004424     --------CF DETG--QSII PPCKSELRVL ------AVKC ISL-LYWTVF PMGT--FALL
GgLPLAT7_NP_001026220     ---------F PPLQGQ---- --TKAVSREM TLSNLWLVGI QSL-AFLSGG --MWY-CILQ
GgLPLAT8_XP_004935228     -------LRL PSDT------ -----CLLE- -----FAKLV RSLGLKPETL E---------
GgLPLAT9_NP_001025739     -------LTL PMEA------ -----GLVE- -----FTKIS KKLNLKWNHV R---------
HsLPLAT1_NP_006402        -------GG- ---------- ---------- ---------- ---------- ----------
HsLPLAT2_NP_006403        -------PAQ ---------- ---------- ---------- ---------- ----------
HsLPLAT3_NP_064517        ---------F P-GEQ----- --FKPARRPW T-LLNF---- ----LSWATI LLSPLFSFVL
HsLPLAT4_NP_064518        ---------F P-ETP----- --MVPPRRPW T-LVNW---- ----LFWASL VLYPFFQFLV
HsLPLAT5_NP_060831        --------RF PGKS-----V NSKLSIKKT- ---------L PSM-LILSGL TAGMLM----
HsLPLAT6_NP_001002257     --------NF YFTG--QSVI PPCKSELRVL ------VVKL LSI-LYWTLF SPAM--CLLI
HsLPLAT7_NP_055688        ---------F PPSKGH---- --KEAVSREM TLSNLWIFLI QSF-AFLSGY --MWY-NIIQ
HsLPLAT8_NP_079106        -------LRL PADT------ -----CLLE- -----FARLV RGLGLKPEKL E---------
HsLPLAT9_NP_060309        -------LTL PMEA------ -----GLVE- -----FTKIS RKLKLDWDGV R---------
HsLPLAT10_NP_705841       -------LKV ALEP------ -----QLWE- -----LGKVL RKAGLSAGYV D---------
```

```
                        661
AqLPLAT1_XP_019853566   --TKHGRFFW LGALI----- ----FYSLCG YIAIQQK--- --------LI D---------
AqLPLAT2_XP_011403842   MG-------- -----IPFVI HYVVNKRFDG WDKLMLR--- --------KW KV--KKRD--
NvLPLAT1_XP_048587027   ---------- ---------- ---------- ---------- ---------- ----------
MgLPLAT1_XP_011449105   ---------- ---------- ---------- ---------- ---------- ----------
MgLPLAT2_XP_011416667   ---------- ---------- ---------- ---------- ---------- ----------
MgLPLAT3_XP_011422898   ---------- ---------- ---------- ---------- ---------- ----------
MgLPLAT4_XP_011457228   --------DL LDKYYAIAKD ----SGGDIT YDEFAKYLHL PKS-DALEEV FNLYDRNGSG
DmLPLAT1_NP_572828      --------DA AVAAAAAVAS SSSAYGQPPG YEAGAAKA-- ----SLLKTL ----------
DmLPLAT2_NP_609231      ---------- ---------- ---------- ---------- ---------- ----------
DmLPLAT3_NP_001036265   --------AE LC--KGFLRL ---SNTDRLD IITFGELLQV DLKNTDLHKL FALLDHRRSG
CeLPLAT1_NP_510606      ---------- ---------- ---------- ---------- ---------- ----------
CeLPLAT2_NP_001256175   ---------- ---------- ---------- ---------- ---------- ----------
CeLPLAT3_NP_001348648   YS-----LLW VKIYVSIAII FYLASLRFYN GAEFVFL--- --------RW FE--ARRDCD
CeLPLAT4_NP_504644      FE-----SAF MFYWAIIACV FYAAVHKFYG GLEFLAI--- --------DR FN--HSKQYG
CeLPLAT5_Q20800         FFVRTLQLGF LYFF---VIS FY-LSWRYGG IDKYIIF--- --------KW QE--SRKSLQ
CeLPLAT6_NP_491479      --SRTIGRIY LLTIAS---- ----SPLLIA WLH--IR--- --------KC V---------
CeLPLAT7_NP_509260      --VYWLRPLI VHSWTSFLSI F--------- ---------- ---------- ----------
CeLPLAT8_NP_502370      --VY------ ---------- ---------- ---------- ---------- ----------
CeLPLAT9_NP_001371003   YLITSIAKFL LFPFSS---- ---------- ---------- ---------- ----------
CtLPLAT1_ELT98524       ---------- ---------- ---------- ---------- ---------- ----------
CtLPLAT2_ELU12181       ---------- ---------- ---------- ---------- ---------- ----------
CtLPLAT3_ELU10392       SLIMTGSYAT LFWAIVIIAV FAIILRLMIA VSEIKSH--- --------G- ------SSYG
DrLPLAT2_NP_001071200   ---------- ---------- ---------- ---------- ---------- ----------
DrLPLAT3_NP_998590      DVFVSGSPLL IIGFMIFLIV ASVAVRRLIG VTEVKKT--- --------G- ------SSYG
DrLPLAT4_NP_998157      QLLLSGSTFT IVCTFVFCLA VSAGVRWMIG QTEIDKG--- --------S- -------NYG
DrLPLAT5_NP_001070213   --TESGRKLY LNTWL----- ----YGTLIG WIWVGLS--- --------P- ----------
DrLPLAT6_NP_998435      CL-----CPP AQFYFLFMVV FFLCQQRFTG GLELMEL--- --------AC HRYWSRRSAD
DrLPLAT7_NP_001025129   HIYFWL---- -F-------- ---------- ---------- ---------- ----------
DrLPLAT8_NP_001037806   --------EV LREEASSARQ ---LCGRRLD MEGFAQYLHQ PMT-EAVQDI FSLFEE--HG
DrLPLAT9_NP_001018492   --------KE LESFANIACS ---CKGGRIT VEEFASFLKL PIS-PALQQL FALFDRNGDG
DrLPLAT10_NP_991122     --------SV MKDMIDSCHS ---GEITMVT ADQLTTVLGL EEK-QTAAKI CSLYSKD--D
XtLPLAT1_XP_012823272   ---------- ---------- ---------- ---------- ---------- ----------
XtLPLAT2_NP_001123741   ---------- ---------- ---------- ---------- ---------- ----------
XtLPLAT3_NP_001008119   GIFASGSPLL ILSFMGFMWT ASFAVRRLIG VTEIER---- --------G- ------SSYG
XtLPLAT4_NP_001011265   NMISSGSYFT ISVFALLMVI VSVGARRMIR VTEIDRG--- --------S- -------AYG
XtLPLAT5_NP_001015964   --TETGRKVY LNAWI----- ----YGTLIG IMWVSIK--- --------A- ----------
XtLPLAT6_NP_001135517   YL-----YSP VRWYFLVTVV IFVLQEKIFG GLELIEL--- --------AC HRYYTRRYKR
XtLPLAT7_XP_017949604   YFYRSL---- -F-------- ---------- ---------- ---------- ----------
XtLPLAT8_XP_002933165   --------KE LDAFCESARK ---RNGKKIT LHEFAAYLYV PDT-EKLADL FALFDENEEG
XtLPLAT9_XP_012815924   --------KQ LEVFASIAGS ---CKGGRIG IEEFANHLKL PVS-DVLREL FSLFDRNKDG
XtLPLAT10_NP_001037863  ---------- --GLIDLCLE ---GVCSRVG LDELAEKLGV TQH-DVISRV FNYFHKDASG
AcLPLAT1_XP_062829728   ---------- ---------- ---------- ---------- ---------- ----------
AcLPLAT2_XP_062816491   ---------- ---------- ---------- ---------- ---------- ----------
AcLPLAT3_XP_008105662   GIFASGSPLL ILAFLGFVGA ASFGVRRLIG VTEIEK---- --------G- ------SSYG
AcLPLAT4_XP_062832607   NMISSGSSLT LATFVFVFVA AIQ-----LG VVTVDTS--- --------GF L-IYIAAYYP
AcLPLAT5_XP_003215489   --TESGRKLY VKTWI----- ----YGTLLG CLWVSIK--- --------A- ----------
AcLPLAT6_XP_003216108   YL-----YSF VRWYFVFVVV IFFMQHKLFG GLELIEL--- --------AC HNFFSKKTVH
AcLPLAT7_XP_016846149   YFYQCL---- -F-------- ---------- ---------- ---------- ----------
AcLPLAT8_XP_016848437   --------KE LDVYSGSAKR ---MKGKRIS IKKFAEYLEV PVS-DTLKNM FALFDEKEDG
AcLPLAT9_XP_003222971   --------EQ LSQFAAIANA ---SKGGRIG IEEFANYLKL PVS-EVLGEL FALFDRNGDG
AcLPLAT10_XP_062813319  ---------- --CQLDPLPD ---GTLGKLK PAELAQWLGL TCP-EAVEGL GTYFQKDSEG
GgLPLAT1_XP_040543510   ---------- ---------- ---------- ---------- ---------- ----------
GgLPLAT2_XP_001235300   ---------- ---------- ---------- ---------- ---------- ----------
GgLPLAT3_XP_004934656   GVFASGSPLL ILAFLGFVGA ASFGVRRLIG VTEIEK---- --------G- ------SSYG
GgLPLAT4_XP_040523863   NMINSGSSLT LASFGFVIVM ASVGVRWMIG VTEINKG--- --------S- -------TYG
GgLPLAT5_XP_040522440   --TESGRKLY VKTWI----- ----YGTLIG CLWVSIK--- --------P- ----------
GgLPLAT6_XP_025004424   YL-----YSF ARWYFAAMII IFVAQQKIFG GLELIEL--- --------AC HQYFKKQQKH
GgLPLAT7_NP_001026220   YFYHCL---- -F-------- ---------- ---------- ---------- ----------
GgLPLAT8_XP_004935228   --------DD LDKYATRAMK ---MKKEKAG LKQFSAYLEF PVS-HTLESM FALFDENEDG
GgLPLAT9_NP_001025739   --------EQ LDTFAAIASA ---SKGGQIG IEEFAEYLKL PIS-DVLREL FLLFDRNGDG
HsLPLAT1_NP_006402      ---------- ---------- ---------- ---------- ---------- ----------
HsLPLAT2_NP_006403      ---------- ---------- ---------- ---------- ---------- ----------
HsLPLAT3_NP_064517      GVFASGSPLL ILTFLGFVGA ASFGVRRLIG VTEIEK---- --------G- ------SSYG
HsLPLAT4_NP_064518      SMIRSGSSLT LASFILVFFV ASVGVRWMIG VTEIDKG--- --------S- -------AYG
HsLPLAT5_NP_060831      --TDAGRKLY VNTWI----- ----YGTLLG CLWVTIK--- --------A- ----------
HsLPLAT6_NP_001002257   YL-----YSL VKWYFIITIV IFVLQERIFG GLEIIEL--- --------AC YRLLHKQPHL
HsLPLAT7_NP_055688      YFYHCL---- -F-------- ---------- ---------- ---------- ----------
HsLPLAT8_NP_079106      --------KD LDRYSERARM ---KGGEKIG IAEFAASLEV PVS-DLLEDM FSLFDESGSG
HsLPLAT9_NP_060309      --------KH LDEYASIASS ---SKGGRIG IEEFAKYLKL PVS-DVLRQL FALFDRNHDG
HsLPLAT10_NP_705841     ---------- --AGAEP--- ---GRSRMIS QEEFARQLQL SDP-QTVAGA FGYFQQDTKG
```

```
                       721
AqLPLAT1_XP_019853566  ---------- ---------- ---------- ---------- ---------- ----------
AqLPLAT2_XP_011403842  ---------- ---------- ---------- ---------- ---------- ----------
NvLPLAT1_XP_048587027  ---------- ---------- ---------- ---------- ---------- ----------
MgLPLAT1_XP_011449105  ---------- ---------- ---------- ---------- ---------- ----------
MgLPLAT2_XP_011416667  ---------- ---------- ---------- ---------- ---------- ----------
MgLPLAT3_XP_011422898  ---------- ---------- ---------- ---------- ---------- ----------
MgLPLAT4_XP_011457228  TIDFREYVIG LSLVSSPENT ED--TIKFAF QLFDEGN--- -KGYITKEEL GIILHKAFN-
DmLPLAT1_NP_572828     ---------- ---------- ---------- ---------- ---------- ----------
DmLPLAT2_NP_609231     ---------- ---------- ---------- ---------- ---------- ----------
DmLPLAT3_NP_001036265  TVSLKSFLLC -SLFCKLKNS DLLTFLRALI HLYSE-S--- -SQQIDRESF VRLMRHAGGK
CeLPLAT1_NP_510606     ---------- ---------- ---------- ---------- ---------- ----------
CeLPLAT2_NP_001256175  ---------- ---------- ---------- ---------- ---------- ----------
CeLPLAT3_NP_001348648  KTSKKE---- ---------- ---------- ---------- ---------- ----------
CeLPLAT4_NP_504644     VVGQEPEV-- ---------- ---------- ---------- ---------- ----------
CeLPLAT5_Q20800        KSPSSSSI-- ---------- ---------- ---------- ---------- ----------
CeLPLAT6_NP_491479     ---------- ---------- ---------- ---------- ---------- ----------
CeLPLAT7_NP_509260     ---------- ---------- ---------- ---------- ---------- ----------
CeLPLAT8_NP_502370     ---------- ---------- ---------- ---------- ---------- ----------
CeLPLAT9_NP_001371003  ---------- ---------- ---------- ---------- ---------- ----------
CtLPLAT1_ELT98524      ---------- ---------- ---------- ---------- ---------- ----------
CtLPLAT2_ELU12181      ---------- ---------- ---------- ---------- ---------- ----------
CtLPLAT3_ELU10392      KKDD------ ---------- ---------- ---------- ---------- ----------
DrLPLAT2_NP_001071200  ---------- ---------- ---------- ---------- ---------- ----------
DrLPLAT3_NP_998590     NVEAKKEN-- ---------- ---------- ---------- ---------- ----------
DrLPLAT4_NP_998157     VKDVQMNN-- ---------- ---------- ---------- ---------- ----------
DrLPLAT5_NP_001070213  ---------- ---------- ---------- ---------- ---------- ----------
DrLPLAT6_NP_998435     KQE------- ---------- ---------- ---------- ---------- ----------
DrLPLAT7_NP_001025129  ---------- ---------- ---------- ---------- ---------- ----------
DrLPLAT8_NP_001037806  MMDVREYVIA LSVVCRPFRY LD--TVKLAF RMFEAQE--- -DGAIVEDEL TVILKTALG-
DrLPLAT9_NP_001018492  TIDFREYVIG VTVLCRPANN EE--VIQTAF KLFDIDE--- -DNCITQEEF SSLLRSALG-
DrLPLAT10_NP_991122    MVDLRQICLS VSAVSGFWSL ES--LIHTAF MWFDTDA--- -DGQLSAEDL SSLMGALLG-
XtLPLAT1_XP_012823272  ---------- ---------- ---------- ---------- ---------- ----------
XtLPLAT2_NP_001123741  ---------- ---------- ---------- ---------- ---------- ----------
XtLPLAT3_NP_001008119  NHEVKKSI-- ---------- ---------- ---------- ---------- ----------
XtLPLAT4_NP_001011265  NRENKLKQ-- ---------- ---------- ---------- ---------- ----------
XtLPLAT5_NP_001015964  ---------- ---------- ---------- ---------- ---------- ----------
XtLPLAT6_NP_001135517  G--------- ---------- ---------- ---------- ---------- ----------
XtLPLAT7_XP_017949604  ---------- ---------- ---------- ---------- ---------- ----------
XtLPLAT8_XP_002933165  TIDLREYVIA LSVVCRPSKT LE--TIKLAF KMYQSED--- -GKTIVEEDL AVILKTALG-
XtLPLAT9_XP_012815924  TIDFREYVIG VAILCNPANT EE--TIKMAF KLFDVDE--- -DGSITEDEF SSLLQSSLG-
XtLPLAT10_NP_001037863 MIDFREVSLV LAAQDATRSA EE--LAKLAF DLFSTCDADG -RFLLSADGF AAILRSVVG-
AcLPLAT1_XP_062829728  ---------- ---------- ---------- ---------- ---------- ----------
AcLPLAT2_XP_062816491  ---------- ---------- ---------- ---------- ---------- ----------
AcLPLAT3_XP_008105662  NQEFKKKK-- ---------- ---------- ---------- ---------- ----------
AcLPLAT4_XP_062832607  FLGKHCKT-- ---------- ---------- ---------- ---------- ----------
AcLPLAT5_XP_003215489  ---------- ---------- ---------- ---------- ---------- ----------
AcLPLAT6_XP_003216108  RSN------- ---------- ---------- ---------- ---------- ----------
AcLPLAT7_XP_016846149  ---------- ---------- ---------- ---------- ---------- ----------
AcLPLAT8_XP_016848437  LIDLREYVIA LSVVCRPSKT LQ--TIQLAF KMYQS-D--- -YGTITEDDL TCILKTAMG-
AcLPLAT9_XP_003222971  SIDFREYVIG LSVLCNPANT EQ--TIQMAF QLFDIDE--- -DGIISEDEF ALIIRSSLG-
AcLPLAT10_XP_062813319 NMDSREVALA LEVLEGEHDP HS--LTSHAF ELFSELDAGT GDHRLCQDGF CTILRLLFG-
GgLPLAT1_XP_040543510  ---------- ---------- ---------- ---------- ---------- ----------
GgLPLAT2_XP_001235300  ---------- ---------- ---------- ---------- ---------- ----------
GgLPLAT3_XP_004934656  NQEFKKKE-- ---------- ---------- ---------- ---------- ----------
GgLPLAT4_XP_040523863  NNDNKQKQ-- ---------- ---------- ---------- ---------- ----------
GgLPLAT5_XP_040522440  ---------- ---------- ---------- ---------- ---------- ----------
GgLPLAT6_XP_025004424  DDTKMKKK-- ---------- ---------- ---------- ---------- ----------
GgLPLAT7_NP_001026220  ---------- ---------- ---------- ---------- ---------- ----------
GgLPLAT8_XP_004935228  IIDVREFVIA LSVVCKPSKT LE--TIQLAF QLYQS-E--- -NGTITEEDL AQILKTAMG-
GgLPLAT9_NP_001025739  TIDFREYVIG LSILCNPANT EE--TIHMAF KLFDQDD--- -DGTITEKEF ACIIQSALG-
HsLPLAT1_NP_006402     ---------- ---------- ---------- ---------- ---------- ----------
HsLPLAT2_NP_006403     ---------- ---------- ---------- ---------- ---------- ----------
HsLPLAT3_NP_064517     NQEFKKKE-- ---------- ---------- ---------- ---------- ----------
HsLPLAT4_NP_064518     NSDSKQKL-- ---------- ---------- ---------- ---------- ----------
HsLPLAT5_NP_060831     ---------- ---------- ---------- ---------- ---------- ----------
HsLPLAT6_NP_001002257  NSKKNE---- ---------- ---------- ---------- ---------- ----------
HsLPLAT7_NP_055688     ---------- ---------- ---------- ---------- ---------- ----------
HsLPLAT8_NP_079106     EVDLRECVVA LSVVCRPART LD--TIQLAF KMYGAQE--- -DGSVGEGDL SCILKTALG-
HsLPLAT9_NP_060309     SIDFREYVIG LAVLCNPSNT EE--IIQVAF KLFDVDE--- -DGYITEEEF STILQASLG-
HsLPLAT10_NP_705841    LVDFRDVALA LAALDGGRSL EE--LTRLAF ELFAEEQAEG PNRLLYKDGF STILHLLLG-
```

Supplement Figure 2A. Sequence alignment utilized in Figure 3A.
AGPAT motifs in HsLPLAT8 were shown.

```
                     781
AqLPLAT1_XP_019853566   ---------- ---------- ---------- ---------- ---------- ----------
AqLPLAT2_XP_011403842   ---------- ---------- ---------- ---------- ---------- ----------
NvLPLAT1_XP_048587027   ---------- ---------- ---------- ---------- ---------- ----------
MgLPLAT1_XP_011449105   ---------- ---------- ---------- ---------- ---------- ----------
MgLPLAT2_XP_011416667   ---------- ---------- ---------- ---------- ---------- ----------
MgLPLAT3_XP_011422898   ---------- ---------- ---------- ---------- ---------- ----------
MgLPLAT4_XP_011457228   MDGLDADALF EEVDLNQDGR IC-------- ---------- ------FDEF SQHAMKKPEY
DmLPLAT1_NP_572828      ---------- ---------- ---------- ---------- ---------- ----------
DmLPLAT2_NP_609231      ---------- ---------- ---------- ---------- ---------- ----------
DmLPLAT3_NP_001036265   LNEQKAQALF YALDTDNLGY VS-------- ---------- ------FDSF VELTEKQKSS
CeLPLAT1_NP_510606      ---------- ---------- ---------- ---------- ---------- ----------
CeLPLAT2_NP_001256175   ---------- ---------- ---------- ---------- ---------- ----------
CeLPLAT3_NP_001348648   ---------- ---------- ---------- ---------- ---------- ----------
CeLPLAT4_NP_504644      ---------- ---------- ---------- ---------- ---------- ----------
CeLPLAT5_Q20800         ---------- ---------- ---------- ---------- ---------- ----------
CeLPLAT6_NP_491479      ---------- ---------- ---------- ---------- ---------- ----------
CeLPLAT7_NP_509260      ---------- ---------- ---------- ---------- ---------- ----------
CeLPLAT8_NP_502370      ---------- ---------- ---------- ---------- ---------- ----------
CeLPLAT9_NP_001371003   ---------- ---------- ---------- ---------- ---------- ----------
CtLPLAT1_ELT98524       ---------- ---------- ---------- ---------- ---------- ----------
CtLPLAT2_ELU12181       ---------- ---------- ---------- ---------- ---------- ----------
CtLPLAT3_ELU10392       ---------- ---------- ---------- ---------- ---------- ----------
DrLPLAT2_NP_001071200   ---------- ---------- ---------- ---------- ---------- ----------
DrLPLAT3_NP_998590      ---------- ---------- ---------- ---------- ---------- ----------
DrLPLAT4_NP_998157      ---------- ---------- ---------- ---------- ---------- ----------
DrLPLAT5_NP_001070213   ---------- ---------- ---------- ---------- ---------- ----------
DrLPLAT6_NP_998435      ---------- ---------- ---------- ---------- ---------- ----------
DrLPLAT7_NP_001025129   ---------- ---------- ---------- ---------- ---------- ----------
DrLPLAT8_NP_001037806   VGDLAVSELF RAIDSQDKGK IT-------- ---------- ------FDDL CSFMEKCPDL
DrLPLAT9_NP_001018492   VCDLDVHSLF REIDADGSGH IT-------- ---------- ------YDEF RSFALNHPEY
DrLPLAT10_NP_991122     IPQRSITEMY SALTSRGQPT EAS-L----- ---------- ---------- RDILLTHPTY
XtLPLAT1_XP_012823272   ---------- ---------- ---------- ---------- ---------- ----------
XtLPLAT2_NP_001123741   ---------- ---------- ---------- ---------- ---------- ----------
XtLPLAT3_NP_001008119   ---------- ---------- ---------- ---------- ---------- ----------
XtLPLAT4_NP_001011265   ---------- ---------- ---------- ---------- ---------- ----------
XtLPLAT5_NP_001015964   ---------- ---------- ---------- ---------- ---------- ----------
XtLPLAT6_NP_001135517   ---------- ---------- ---------- ---------- ---------- ----------
XtLPLAT7_XP_017949604   ---------- ---------- ---------- ---------- ---------- ----------
XtLPLAT8_XP_002933165   VSELNVSDLF RVIDEENMGK IT-------- ---------- ------YDEF NSYAMMNPTF
XtLPLAT9_XP_012815924   VPDLDVSKLF RDMDADNSGK IS-------- ---------- ------YEEF RNFSLNHPEY
XtLPLAT10_NP_001037863  SPPAESGKVF SELCTYTELH GLTQD----- ---------- --------GF VRFAIRHPCY
AcLPLAT1_XP_062829728   ---------- ---------- ---------- ---------- ---------- ----------
AcLPLAT2_XP_062816491   ---------- ---------- ---------- ---------- ---------- ----------
AcLPLAT3_XP_008105662   ---------- ---------- ---------- ---------- ---------- ----------
AcLPLAT4_XP_062832607   ---------- ---------- ---------- ---------- ---------- ----------
AcLPLAT5_XP_003215489   ---------- ---------- ---------- ---------- ---------- ----------
AcLPLAT6_XP_003216108   ---------- ---------- ---------- ---------- ---------- ----------
AcLPLAT7_XP_016846149   ---------- ---------- ---------- ---------- ---------- ----------
AcLPLAT8_XP_016848437   VADLNVANLF RAIDVEGRGK VT-------- ---------- ------YDDF HRFTEMYPDF
AcLPLAT9_XP_003222971   LPDLDVSNLF KEIDADKTNK LS-------- ---------- ------YEEF KNFALKHPEY
AcLPLAT10_XP_062813319  TPAADGAELY SRLSAEESSQ NELTLAEIFW HCTQCAHRHR DHLHWFLPES LKFNLEVLIA
GgLPLAT1_XP_040543510   ---------- ---------- ---------- ---------- ---------- ----------
GgLPLAT2_XP_001235300   ---------- ---------- ---------- ---------- ---------- ----------
GgLPLAT3_XP_004934656   ---------- ---------- ---------- ---------- ---------- ----------
GgLPLAT4_XP_040523863   ---------- ---------- ---------- ---------- ---------- ----------
GgLPLAT5_XP_040522440   ---------- ---------- ---------- ---------- ---------- ----------
GgLPLAT6_XP_025004424   ---------- ---------- ---------- ---------- ---------- ----------
GgLPLAT7_NP_001026220   ---------- ---------- ---------- ---------- ---------- ----------
GgLPLAT8_XP_004935228   VSQINVTHLF RAVDEEEKGK IT-------- ---------- ------YDDF YRFAELQPHF
GgLPLAT9_NP_001025739   LPDLDVSNLF KEIDADETGK LS-------- ---------- ------YDEF KNFALEHPEY
HsLPLAT1_NP_006402      ---------- ---------- ---------- ---------- ---------- ----------
HsLPLAT2_NP_006403      ---------- ---------- ---------- ---------- ---------- ----------
HsLPLAT3_NP_064517      ---------- ---------- ---------- ---------- ---------- ----------
HsLPLAT4_NP_064518      ---------- ---------- ---------- ---------- ---------- ----------
HsLPLAT5_NP_060831      ---------- ---------- ---------- ---------- ---------- ----------
HsLPLAT6_NP_001002257   ---------- ---------- ---------- ---------- ---------- ----------
HsLPLAT7_NP_055688      ---------- ---------- ---------- ---------- ---------- ----------
HsLPLAT8_NP_079106      VAELTVTDLF RAIDQEEKGK IT-------- ---------- ------FADF HRFAEMYPAF
HsLPLAT9_NP_060309      VPDLDVSGLF KEIAQG--DS IS-------- ---------- ------YEEF KSFALKHPEY
HsLPLAT10_NP_705841     SPHPAATALH AELCQAGSSQ GL-SLCQF-- -------Q-- ---------- -NFSLHDPLY
```

```
                     841
AqLPLAT1_XP_019853566  ---------- ---------- ---------- ---------- ---------- ----------
AqLPLAT2_XP_011403842  ---------- ---------- ---------- ---------- ---------- ----------
NvLPLAT1_XP_048587027  ---------- ---------- ---------- ---------- ---------- ----------
MgLPLAT1_XP_011449105  ---------- ---------- ---------- ---------- ---------- ----------
MgLPLAT2_XP_011416667  ---------- ---------- ---------- ---------- ---------- ----------
MgLPLAT3_XP_011422898  ---------- ---------- ---------- ---------- ---------- ----------
MgLPLAT4_XP_011457228  AK-LFKTYQD TKMNGHEKQE ---------- ---------- ---------- ----------
DmLPLAT1_NP_572828     ---------- ---------- ---------- ---------- ---------- ----------
DmLPLAT2_NP_609231     ---------- ---------- ---------- ---------- ---------- ----------
DmLPLAT3_NP_001036265  YKFLYHKS-E HIRRPKAV-- ---------- ---------- -VT----TAE N---------
CeLPLAT1_NP_510606     ---------- ---------- ---------- ---------- ---------- ----------
CeLPLAT2_NP_001256175  ---------- ---------- ---------- ---------- ---------- ----------
CeLPLAT3_NP_001348648  ---------- ---------- ---------- ---------- ---------- ----------
CeLPLAT4_NP_504644     ---------- ---------- ---------- ---------- --------LN T---------
CeLPLAT5_Q20800        ---------- ---------- ---------- ---------- ---------- ----------
CeLPLAT6_NP_491479     ---------- ---------- ---------- ---------- ---------- ----------
CeLPLAT7_NP_509260     ---------- ---------- ---------- ---------- ---------- ----------
CeLPLAT8_NP_502370     ---------- ---------- ---------- ---------- ---------- ----------
CeLPLAT9_NP_001371003  ---------- ---------- ---------- ---------- ---------- ----------
CtLPLAT1_ELT98524      ---------- ---------- ---------- ---------- ---------- ----------
CtLPLAT2_ELU12181      ---------- ---------- ---------- ---------- ---------- ----------
CtLPLAT3_ELU10392      ---------- ---------- ---------- ---------- ---------- ----------
DrLPLAT2_NP_001071200  ---------- ---------- ---------- ---------- ---------- ----------
DrLPLAT3_NP_998590     ---------- ---------- ---------- ---------- ---------- ----------
DrLPLAT4_NP_998157     ---------- ---------- ---------- ---------- ------N--- ----------
DrLPLAT5_NP_001070213  ---------- ---------- ---------- ---------- ---------- ----------
DrLPLAT6_NP_998435     ---------- ---------- ---------- ---------- ---------- ----------
DrLPLAT7_NP_001025129  ---------- ---------- ---------- ---------- ---------- ----------
DrLPLAT8_NP_001037806  VE-QYHCL-C ESISQ--H-- ---------- ---------- --SRESTSSS NGFCADFSPR
DrLPLAT9_NP_001018492  AK-LFTTYIE LQRYQGLQGD ETDFDS---- ---------- --------AF SHCCTAAYDE
DrLPLAT10_NP_991122    RK-VITEYLH FSGLEDG--N H--------- ---------- ------NGLP NGKTNGNNNG
XtLPLAT1_XP_012823272  ---------- ---------- ---------- ---------- ---------- ----------
XtLPLAT2_NP_001123741  ---------- ---------- ---------- ---------- ---------- ----------
XtLPLAT3_NP_001008119  ---------- ---------- ---------- ---------- ---------- ----------
XtLPLAT4_NP_001011265  ---------- ---------- ---------- ---------- ------K--- ----------
XtLPLAT5_NP_001015964  ---------- ---------- ---------- ---------- ---------- ----------
XtLPLAT6_NP_001135517  ---------- ---------- ---------- ---------- ---------- ----------
XtLPLAT7_XP_017949604  ---------- ---------- ---------- ---------- ---------- ----------
XtLPLAT8_XP_002933165  AE-DYLYP-D LVDMDSCM-- ---------- ---------- --ESHSPTTP NGICPDFTPE
XtLPLAT9_XP_012815924  AK-LFTTYLE HQRYYAYMLE CEEKE----- ---------- DYNVMVQSAA NKLCPISN-E
XtLPLAT10_NP_001037863 RH-LFLFYLR PPSSGRRKPP H--------- ---------- ------IQQN GGCSGKNNPR
AcLPLAT1_XP_062829728  ---------- ---------- ---------- ---------- ---------- ----------
AcLPLAT2_XP_062816491  ---------- ---------- ---------- ---------- ---------- ----------
AcLPLAT3_XP_008105662  ---------- ---------- ---------- ---------- ---------- ----------
AcLPLAT4_XP_062832607  ---------- ---------- ---------- ---------- ------TSCN TGSCNTGSYI
AcLPLAT5_XP_003215489  ---------- ---------- ---------- ---------- ---------- ----------
AcLPLAT6_XP_003216108  ---------- ---------- ---------- ---------- ---------- ----------
AcLPLAT7_XP_016846149  ---------- ---------- ---------- ---------- ---------- ----------
AcLPLAT8_XP_016848437  AE-EYLYP-D QMETGSSL-- ---------- ---------- --EA---PAP NGLCTDFSPE
AcLPLAT9_XP_003222971  AK-LFTTYIE LQRNQMCIEA E--------- -FEPAATPNT SVSEKSSVPR NKVCPQTS-E
AcLPLAT10_XP_062813319 AE-FFLLCCC ---CCCCCCC ---------- ---------- --------CC CCCTPGSNPR
GgLPLAT1_XP_040543510  ---------- ---------- ---------- ---------- ---------- ----------
GgLPLAT2_NP_001235300  ---------- ---------- ---------- ---------- ---------- ----------
GgLPLAT3_XP_004934656  ---------- ---------- ---------- ---------- ---------- ----------
GgLPLAT4_XP_040523863  ---------- ---------- ---------- ---------- ------K--- ----------
GgLPLAT5_XP_040522440  ---------- ---------- ---------- ---------- ---------- ----------
GgLPLAT6_XP_025004424  ---------- ---------- ---------- ---------- ---------- ----------
GgLPLAT7_NP_001026220  ---------- ---------- ---------- ---------- ---------- ----------
GgLPLAT8_XP_004935228  AE-DYLYP-D QMVAESGL-- ---------- ---------- --ETSSLSAP NGICTDFSPE
GgLPLAT9_NP_001025739  AK-LFTTYLE LQRHQLGISE EDDNIQSSEA KHTPIRKPAV LVSETRSTAR NKVCPADC-E
HsLPLAT1_NP_006402     ---------- ---------- ---------- ---------- ---------- ----------
HsLPLAT2_NP_006403     ---------- ---------- ---------- ---------- ---------- ----------
HsLPLAT3_NP_064517     ---------- ---------- ---------- ---------- ---------- ----------
HsLPLAT4_NP_064518     ---------- ---------- ---------- ---------- ------ND-- ----------
HsLPLAT5_NP_060831     ---------- ---------- ---------- ---------- ---------- ----------
HsLPLAT6_NP_001002257  ---------- ---------- ---------- ---------- ---------- ----------
HsLPLAT7_NP_055688     ---------- ---------- ---------- ---------- ---------- ----------
HsLPLAT8_NP_079106     AE-EYLYP-D QTHFESCA-- ---------- ---------- --ETSPAPIP NGFCADFSPE
HsLPLAT9_NP_060309     AK-IFTTYLD LQTCHVFSLP KEV------- ---------- --QTTPSTAS NKVSP----E
HsLPLAT10_NP_705841    GK-LFSTYLR PPHTSRGTSQ ---------- ---------- --------TP NASSPG-NPT
```

```
                              901
AqLPLAT1_XP_019853566      ---------- ------
AqLPLAT2_XP_011403842      ---------- ------
NvLPLAT1_XP_048587027      ---------- ------
MgLPLAT1_XP_011449105      ---------- ------
MgLPLAT2_XP_011416667      ---------- ------
MgLPLAT3_XP_011422898      ---------- ------
MgLPLAT4_XP_011457228      ---------- ------
DmLPLAT1_NP_572828         ---------- ------
DmLPLAT2_NP_609231         ---------- ------
DmLPLAT3_NP_001036265      ---------- ------
CeLPLAT1_NP_510606         ---------- ------
CeLPLAT2_NP_001256175      ---------- ------
CeLPLAT3_NP_001348648      ---------- ------
CeLPLAT4_NP_504644         ---------- ------
CeLPLAT5_Q20800            ---------- ------
CeLPLAT6_NP_491479         ---------- ------
CeLPLAT7_NP_509260         ---------- ------
CeLPLAT8_NP_502370         ---------- ------
CeLPLAT9_NP_001371003      ---------- ------
CtLPLAT1_ELT98524          ---------- ------
CtLPLAT2_ELU12181          ---------- ------
CtLPLAT3_ELU10392          ---------- ------
DrLPLAT2_NP_001071200      ---------- ------
DrLPLAT3_NP_998590         ---------- ------
DrLPLAT4_NP_998157         ---------- ------
DrLPLAT5_NP_001070213      ---------- ------
DrLPLAT6_NP_998435         ---------- ------
DrLPLAT7_NP_001025129      ---------- ------
DrLPLAT8_NP_001037806      NHSKKQ-D-- ------
DrLPLAT9_NP_001018492      YQEDSA-S-- -DKKDD
DrLPLAT10_NP_991122        IY---HGYRT TNRKTD
XtLPLAT1_XP_012823272      ---------- ------
XtLPLAT2_NP_001123741      ---------- ------
XtLPLAT3_NP_001008119      ---------- ------
XtLPLAT4_NP_001011265      ---------- ------
XtLPLAT5_NP_001015964      ---------- ------
XtLPLAT6_NP_001135517      ---------- ------
XtLPLAT7_XP_017949604      ---------- ------
XtLPLAT8_XP_002933165      EIERPE-NHN KQKKLN
XtLPLAT9_XP_012815924      EERISV-S-- -DKKED
XtLPLAT10_NP_001037863     NQSKMD---- ------
AcLPLAT1_XP_062829728      ---------- ------
AcLPLAT2_XP_062816491      ---------- ------
AcLPLAT3_XP_008105662      ---------- ------
AcLPLAT4_XP_062832607      NFNAA----- ------
AcLPLAT5_XP_003215489      ---------- ------
AcLPLAT6_XP_003216108      ---------- ------
AcLPLAT7_XP_016846149      ---------- ------
AcLPLAT8_XP_016848437      TVEERR-T-R LQKKLD
AcLPLAT9_XP_003222971      DDHSSV-S-- -DKKED
AcLPLAT10_XP_062813319     LSGNGDSYNP VQS-R-
GgLPLAT1_XP_040543510      ---------- ------
GgLPLAT2_XP_001235300      ---------- ------
GgLPLAT3_XP_004934656      ---------- ------
GgLPLAT4_XP_040523863      ---------- ------
GgLPLAT5_XP_040522440      ---------- ------
GgLPLAT6_XP_025004424      ---------- ------
GgLPLAT7_NP_001026220      ---------- ------
GgLPLAT8_XP_004935228      NAEDKN-K-P LQKKWN
GgLPLAT9_NP_001025739      EDNSST-S-- -DKKDD
HsLPLAT1_NP_006402         ---------- ------
HsLPLAT2_NP_006403         ---------- ------
HsLPLAT3_NP_064517         ---------- ------
HsLPLAT4_NP_064518         ---------- ------
HsLPLAT5_NP_060831         ---------- ------
HsLPLAT6_NP_001002257      ---------- ------
HsLPLAT7_NP_055688         ---------- ------
HsLPLAT8_NP_079106         NSDAGR-K-P VRKKLD
HsLPLAT9_NP_060309         KHEEST-S-- -DKKDD
HsLPLAT10_NP_705841        ALANGTVQAP KQK-GD
```

Supplementary Fig. 2B

```
                          1
AqLPLAT3_XP_019855423     ---------- ---------- ---------- ---------M FFEYFSDLLG FPVDRVRYVT
NvLPLAT2_XP_048575357     ---------- ---------- ---------- ---------- ---MGGGGGR ITPGEYFFFG
MgLPLAT5_XP_034333714     ---------- ---------- ---------- ---------- ---------M ELTDDVVYSL
MgLPLAT6_XP_011416786     ---------- ---------- --------MV HRGEFYMGSG LLQPLT-VIG LPLDQINFLV
DmLPLAT4_NP_995637        ---------- ---------- ---------- ---------- ---------- MSIDDVIYVI
DmLPLAT5_NP_524157        ---------- ---------- --------MA EFEEDLPHNG LMDGIASGVG VPVEALRLLL
DmLPLAT6_NP_610546        ---------- ---------- MLEPPKFIEN D---CYNGSR TFTWLADMVG LSVDLVNFLI
CeLPLAT10_NP_001370561    ---------- ---------- --------MP GIHTFYDGSK ILEPLAETIG IEADRVNFVL
CeLPLAT11_NP_001022735    ---------- ---------- ---------- --------MG VVGALSEVTS ASEDALRLLI
CeLPLAT12_NP_001255544    ---------- ---------- MSSLNEDVDE SNHIFYDPVT LLRPVSRAIS MNLDQLNFVT
CtLPLAT4_ELT92151         ---------- ---------- ---------- ---------- ---------- MSPADFLYAG
CtLPLAT5_ELT92172         ---------- ---------- ---------- --MEELPDLG YVERLAATLG ASESALRLLI
DrLPLAT11_NP_956831       ---------- ---------- ---------- ---------- ---------- MSPDELVYLG
DrLPLAT12_XP_073781106    ---------- ---------- ---------- ------MAAP FMEKLAEYLD SPEPAVRLIL
DrLPLAT13_XP_073783893    ---------- ---------- ---------- ---------- ---------- ----------
DrLPLAT14_NP_001007397    ---------- --------MD KKKNI----- DIAFKTTGCT WLSPVSDQVG VPLDQVNFIA
DrLPLAT15_NP_001265531    ---------- ---------- -------MAT ESISACTGSN LLQPISEMTN LPLDQVNFVV
XtLPLAT11_XP_012822891    ---------- ---------- ---------- ---------- ---------- MSPDELTYLA
XtLPLAT12_XP_002941254    ---------- ---------- ---------- ---MAATGGS VLYQVAEALG ASEPALRLIL
XtLPLAT13_NP_001096155    ---------- ---------- ---------- -MATSTTGST LLLPLSRAVQ LPVDQVNFVA
XtLPLAT14_XP_002940346    ---------- ---------- MVAAS----G NIVPQPTGSA LLQPISQLLH TPLEQVNFVA
AcLPLAT11_XP_062840076    ---------- ---------- ---------- ---------- ---------- MSPEEIIYLS
AcLPLAT12_XP_003223803    MAAAEAGS-- ---------- ---------- -GGGAFRIGT SLAEIAASLG SSEQALRLIL
AcLPLAT13_XP_008115909    ---------- ---------- ---------- ---MATTGST LLQPLSSAVL LPIDQVNFVV
AcLPLAT14_XP_003219829    ---------- ---------- MAEGSRIPYR T-TG----ST LLHPVSLLLG IPLDQVNFFA
GgLPLAT12_XP_040517541    MGAERDESGA GAAVLVPLLG FGSNRPAGPA EKMAVAGSGW SLARVAEALG SSEQALRLIV
GgLPLAT13_NP_001026261    ---------- ---------- ---------- -MATSTTGST LLQPLSNAVR LPVDQVNFVV
GgLPLAT14_XP_046767333    MFYS------ ---VCCNKVE EIKCRQMIWR QQLGTSRTYS LIYIQFLMAR CFASTINFVA
HsLPLAT11_NP_077274       ---------- ---------- ---------- ---------- ---------- MSPEEWTYLV
HsLPLAT12_NP_005759       MASSAEGD-E GTV--VALAG ---------- -VLQSGFQEL SLNKLATSLG ASEQALRLII
HsLPLAT13_NP_620154       ---------- ---------- ---------- MATTSTTGST LLQPLSNAVQ LPIDQVNFVV
HsLPLAT14_NP_001073949    ---------- -------MAA EPQPSSLSYR T-----TGST YLHPLSELLG IPLDQVNFVV

                          61
AqLPLAT3_XP_019855423     LFLVSFPLAS LLRVVLHYSY TSPTIRHTVS ALLGLL-FGW ICF-QWQVFY LI--GFVSVG
NvLPLAT2_XP_048575357     KC-----IDF FFFL------ ---------- --LQLVAVNI FFS-G-FFFH FLVECSIIAG
MgLPLAT5_XP_034333714     ILLLSVPVGS AVKLI----- PSPELKKAVC TIIGIV-AAF LTI-GGHILH PL--FTSVAS
MgLPLAT6_XP_011416786     CQLTALFLGF YFRKNWSPQK ISVVHRHALE MAVGFL-LTF FCF-GYQVIH VV--LMGWLC
DmLPLAT4_NP_995637        CLLGCIGAGS YVKKI----- ADEGQRKLVS TGLGVL-VVV IVS-GLHSLH CFVSLAL-GT
DmLPLAT5_NP_524157        TILAGYPVAA LYQKFISV-I ADKTVHHMFF AGCGAG-LCY FNY-GLDTYH SL--IAILTT
DmLPLAT6_NP_610546        CQISALFLAS LFRSMLHPSK VSSKLRHTFA LSIGLA-FGY FCF-GQQAIH IA--GLPAIC
CeLPLAT10_NP_001370561    SLFACFGISY VYRKTCSLKQ VNRQVRTVVP AVVGIG-IVF FCF-GRAIKH LL--ANALGS
CeLPLAT11_NP_001022735    SVLAGYPLAV VHRTF-FY-N KPAQHQHLFF VIVGLS-LWM FNC-GSSVIH PI--LSIFGA
CeLPLAT12_NP_001255544    VLLLSFGLGY WFRVQF--CD ASRTTRAAVT TSVGLI-FTY FCY-GNAIAH LF--INGFGS
CtLPLAT4_ELT92151         LLFFSVAFGQ VVKIN----- GSPEKRRLIS TSLGVV-III FVG-GFGCFH AI--VAGVVN
CtLPLAT5_ELT92172         SLFAGYPLAV IHRLV-FY-G QAPNVQHLFF TLASLA-IGY FNF-GWEIAH SF--INIVLC
DrLPLAT11_NP_956831       ILAATIPVGF LFRY------ LSPPVKQGAA LLLGLI-ISI ATC-GIHTLH SL--CTVLGT
DrLPLAT12_XP_073781106    SILIGYPFAL AYRWF-LF-H QSSTVLHLFH TITGLA-LAT FNFAGSQVYH SL--LCVVIQ
DrLPLAT13_XP_073783893    ---------- ---------- ---------- ---------- ---------- ----------
DrLPLAT14_NP_001007397    CQLFGLAAAF WFRLFLSPQR ATPEVRHAVA AILGAS-SVM FCF-GWYAVH IF--ILVLAC
DrLPLAT15_NP_001265531    CQLCALLTAV WFRLYLHPSK TSPFVRHVVA TLLGIY-FAL FCF-GWYALH FL--LQSGIT
XtLPLAT11_XP_012822891    ILLCSAPLGF LFKN------ GSPQVKQRGS AAVGLA-LTL ITC-HIHTLH SV--IAILGT
XtLPLAT12_XP_002941254    SIFLGYPLAL FQRYF-QF-K KEPSHIHLFN TLTGLG-IAY FNF-GSQLYH SL--LCVVLN
XtLPLAT13_NP_001096155    CQVFALLAAI WFRIYLHSSK TSPLIRHVVA TLLGLY-LAL FCF-GWYALH FI--VQSGIA
XtLPLAT14_XP_002940346    CQLVALLVAF WFRIYLNPSN AHPAVRHAFA TFVGVY-FAV FCF-GWYSLH IF--TLVLLC
AcLPLAT11_XP_062840076    VLLVSIPIGF LFKK------ GGLKTKQFGG AAAGLV-LTL ATC-HIHTLH SL--ITILGT
AcLPLAT12_XP_003223803    SILMGYPFAL FQRYF-LF-Q KELYLIHLSN ILMGIS-VAY FNF-GTMFCH SL--ICVLVQ
AcLPLAT13_XP_008115909    CQLFALLAAV WFRTYLHSSK TSPFIRHLVA TLLGLY-LAL FCF-GWYALH FV--IQSGIS
AcLPLAT14_XP_003219829    CQLFALLAAF WFRSYLSPSH VSSGVRHAFA TIFGLY-FAV FCF-GWYSIH IF--VLVMIS
GgLPLAT12_XP_040517541    SILMGYPFAL FQRYF-LF-Q KETYLIHLYN VFTGLS-IAY FNF-GMQFFH SL--LCVLIQ
GgLPLAT13_NP_001026261    CQLFALLAAI WFRTYLHSSK TSPFIRHVVA TLLGLY-LAL FCF-GWYALH FV--IQSGIS
GgLPLAT14_XP_046767333    CQLFALLAAF WFRIYLGPSH ASSAVRHAFA TLFGIY-FAV FCF-GWYSIH LF--VLVLMN
HsLPLAT11_NP_077274       VLLISIPIGF LFKK------ AGPGLKRWGA AAVGLG-LTL FTC-GPHTLH SL--VTILGT
HsLPLAT12_NP_005759       SIFLGYPFAL FYRHY-LF-Y KETYLIHLFH TFTGLS-IAY FNF-GNQLYH SL--LCIVLQ
HsLPLAT13_NP_620154       CQLFALLAAI WFRTYLHSSK TSSFIRHVVA TLLGLY-LAL FCF-GWYALH FL--VQSGIS
HsLPLAT14_NP_001073949    CQLVALFAAF WFRIYLRPGT TSSDVRHAVA TIFGIY-FVI FCF-GWYSVH LF--VLVLMC
```

```
                       121
AqLPLAT3_XP_019855423  YM--LLITL- P-PTIVQRYS MIYALIGIST GHIYR-LITD YGGYTLDFTG ----PLMLLV
NvLPLAT2_XP_048575357  FHVTSPGSL- F-VRRCQWVA FLFVFGYLFF FRTCT--YFG FKKPPMQSNA ----VQLLVT
MgLPLAT5_XP_034333714  IL--AIKIV- G-PRKCHIFL FFGCFGYLAF FRTCH--YFG IPKVSPISNA ----VQLFIT
MgLPLAT6_XP_011416786  YA--LILHA- P-RDKFHVLV FIFGMGYLCI MHIYR-QYYD YGGYSLDITG ----PLMILT
DmLPLAT4_NP_995637     A---AVLLV- H-PSKGHLVT FAVMFGYLVF FRIFD-FYFG IPG---HTNM ----IQMILT
DmLPLAT5_NP_524157     YF--LVLLLR KKTQIFLAIN FVFHMSYLLL GY----FYTS SNDYDILWTM ----PHCILV
DmLPLAT6_NP_610546     YI--VIRTQ- D-PRIVQRAV LLVAMSYLLC VHLMR-QLYD YGSYALDITG ----PLMIIT
CeLPLAT10_NP_001370561 YA--IMYFA- P-PTQVHKIV LLFSMGYLFF IHCYRWAIL- -ESYSLDVTG ----PIMVAV
CeLPLAT11_NP_001022735 FF--ITNFM- AGTDASIYAA HIVFLGHLLI GY----WFHE TDTYDITWTT ----PFCIMT
CeLPLAT12_NP_001255544 YL--LMVSV- P-PQHVHKSV FAFAMGYLVL IHSYRWMYQ- -KTYCLDVTG ----SMMVAV
CtLPLAT4_ELT92151      AL--IIGFI- D-PSICHIAS FIWGFGYIAF FRTCH--FIG LPMPSPYSNA ----VQLLLT
CtLPLAT5_ELT92172      HV--LMRSL- GGSLLCIVLA FTLNVMYLLV GY----YSVD TFNVTVCWTT ----PQCVLT
DrLPLAT11_NP_956831    WI--IIK-I- N-WRSAPALS LAWTFLYLLF FRLVT--WFG LPQPTPFANA ----IQLLLT
DrLPLAT12_XP_073781106 FL--LLRLM- GRTVTCVLTS FIFQMIYLLT GY----YYTA TDEYDIKWTM ----PHCVLT
DrLPLAT13_XP_073783893 ---------- ---------- ---------- ---------- ---------- ----------
DrLPLAT14_NP_001007397 YG--IMIKA- S-VRNVHRYT MVASIGYLTA CQVSRVFIFD YGILSTDFSG ----PLMMIT
DrLPLAT15_NP_001265531 YG--IMILT- G-VEHMHKYC LVVALGYLSF CQITRVYVFD YGMYSADFTG ----PMMVIT
XtLPLAT11_XP_012822891 WL--IIK-I- T-PRACHFPT LGWTFAYLLF FRTVT--YFD LPAPTPFTNA ----VQLLLT
XtLPLAT12_XP_002941254 FL--ILRLM- GRTMTAVFTS FCLQMGYLLC GY----YYTA TDNYDIKWTM ----PHCVLT
XtLPLAT13_NP_001096155 YY--IMIAG- R-LENMHKFC FVFALGYLTL CQIARVYIFD YGQYSADFSG ----PMMIMT
XtLPLAT14_XP_002940346 YC--IMITA- S-VSNVHRYS FIMAMGYLTL CQINRVYIFN YGILSTDFSG ----PLMIIT
AcLPLAT11_XP_062840076 WL--IIN-I- S-PRACHYLT LGWTFSYLLF FRTVT--FFD FPEPTPFTNA ----VQLLLT
AcLPLAT12_XP_003223803 FL--ILRLM- GRTISAIITT FCFQMVYLMA GY----YYTA TENYDIKWTM ----PHCVLT
AcLPLAT13_XP_008115909 YY--IMIII- G-VENMHKYC FVFALGYLTV CQISRVYIFD YGQYSADFSG ----PMMIIT
AcLPLAT14_XP_003219829 YS--IMVLA- S-IPNIHRYS FMVAMGYLTL CHVSRIYIFS YGILTTDFSG ----PLMIIT
GgLPLAT12_XP_040517541 FL--ILRLM- GRTVTAVFTT FVFQMTYLMA GY----YFTA TEHYDIKWTM ----PHCVLT
GgLPLAT13_NP_001026261 YY--LMIII- G-VENMHKYC FVFALGYLTV CQITRVYIFD YGQYSADFSG ----PMMIIT
GgLPLAT14_XP_046767333 YG--IMNMA- S-IPNIHRYS FVVAMGYLTL CHISRIYIFH YGILTTDFSG GLCCPLMIIT
HsLPLAT11_NP_077274    WA--LIQ-A- Q-PCSCHALA LAWTFSYLLF FRALS--LLG LPTPTPFTNA ----VQLLLT
HsLPLAT12_NP_005759    FL--ILRLM- GRTITAVLTT FCFQMAYLLA GY----YYTA TGNYDIKWTM ----PHCVLT
HsLPLAT13_NP_620154    YC--IMIII- G-VENMHNYC FVFALGYLTV CQVTRVYIFD YGQYSADFSG ----PMMIIT
HsLPLAT14_NP_001073949 YA--IMVTA- S-VSNIHRYS FFVAMGYLTI CHISRIYIFH YGILTTDFSG ----PLMIVT

                       181
AqLPLAT3_XP_019855423  QRVSYVAYAY HDGVARDEKD LSDDQKKL-- ---------- -----KISSI PSLLEYFSYN
NvLPLAT2_XP_048575357  LRLCTLPFEI FDPEISRNKA A--------- ---------- ------ATSK PSFYEFLSYS
MgLPLAT5_XP_034333714  LRLIGLGFEI HDSYREL--- SSQEGSSEER HLR------- ---KQYVCID PSSVDMFQYA
MgLPLAT6_XP_011416786  QKVTSIAFAM HDGMQKKDQD LNPDQKIM-- ---------- -----KIKKT PTVLEYFSYL
DmLPLAT4_NP_995637     LKVSGIAFEK TAAWKRLQAH D--EQKKNDQ RDVHQESPIE ITDYDVELQS LSAAEILHYS
DmLPLAT5_NP_524157     LRMIGYGFDI TDGL-KEESE LSKDQKET-- ---------- -----ALKKP PSLLELLAFS
DmLPLAT6_NP_610546     QKVTSLAFSI HDGFVRGDEE LTKAQQYH-- ---------- -----AIRKM PSALEYFSYV
CeLPLAT10_NP_001370561 EKATMMAFNL KDGKAKDQSK LTEEQKRE-- ---------- -----SLKEI PSLLEFMSFM
CeLPLAT11_NP_001022735 LRFIGLVMDV YDGA-QKPEH LKPDQKLT-- ---------- -----AISDK PGLLEIAAFG
CeLPLAT12_NP_001255544 GKITLLASAI TDGMGRDPKA LNSGQKRD-- ---------- -----AVKEV PSLLDFASYM
CtLPLAT4_ELT92151      LKMVGIAFEV HDTDKRKRNI PSDEEEKAKH LLK------- ---CKYQDIH VSVVDIICYS
CtLPLAT5_ELT92172      LRLTALAFNL YDGS-KNEDE LSAEQKET-- ---------- -----ALKDV PSLLEMTGFS
DrLPLAT11_NP_956831    LKMVSLANEV QSYHLERKKE V-STFTK--- SPV------- ---VAGLSHE PSLYDIISYS
DrLPLAT12_XP_073781106 LKLIGLALDY YDGG-KDQSK LSAEQKKS-- ---------- -----ALGST PSLLEVFGFS
DrLPLAT13_XP_073783893 ---------- ---MLH-ILC EPIIRKRT-- ---------- -----EKRKM PSLLEYLSYN
DrLPLAT14_NP_001007397 QKITSLAFQV YDGMGRKQEE LKDDQRQL-- ---------- -----AVHSK PSLLEYLSYN
DrLPLAT15_NP_001265531 QKITSVAFEI HDGMARKVEQ LNQGQKLL-- ---------- -----AIRRM PSLLEYFSYN
XtLPLAT11_XP_012822891 LKLVSLANEV QDFYRTKKQE V-ASFSK--- SSV------- ---ISTIPSV PSLREMFYYS
XtLPLAT12_XP_002941254 LKLIGLTFDY YDSS-KDKES MTEEQMRY-- ---------- -----AVPGV PSLMEVCGFS
XtLPLAT13_NP_001096155 QKITSLAFEI RDGIFCKEEE LTPSQRRL-- ---------- -----AVRRM PSLLEYLSYN
XtLPLAT14_XP_002940346 QKITTLAFQL HDGFGRSFKV LSKDQQQY-- ---------- -----SIKKK PSFLEYLSYH
AcLPLAT11_XP_062840076 LKMVSLANEV QEFTQAKKQE V-TSFSK--- SSV------- ---IGVIPKM PGLVEILCYS
AcLPLAT12_XP_003223803 LKLIGLVIDY YDGR-KEVES LTPEQQKY-- ---------- -----VVQGV PTLLEVAGFS
AcLPLAT13_XP_008115909 QKITSLAYEI HDGMFRKDEE LTPSQQCL-- ---------- -----AVRRM PSLLEYLSYN
AcLPLAT14_XP_003219829 QKITILAFQV HDGIGRKLEE LTKEQKEL-- ---------- -----AIKTK PSLLEYLSYQ
GgLPLAT12_XP_040517541 LKLIGLAIDY YDGG-KDPEL LTPEQRRF-- ---------- -----AVRGV PTLLEVSGFS
GgLPLAT13_NP_001026261 QKITSLAFEI HDGMFRKNED LTPSQRCL-- ---------- -----AVRRM PSLLEYLSYN
GgLPLAT14_XP_046767333 QKITTLACQL HDGIGRQAEE LTAEQNRL-- ---------- -----AVKTR PSLLEYLSYL
HsLPLAT11_NP_077274    LKLVSLASEV QDLHLAQRKE MASGFSK--- GPT------- ---LGLLPDV PSLMETLSYS
HsLPLAT12_NP_005759    LKLIGLAVDY FDGG-KDQNS LSSEQQKY-- ---------- -----AIRGV PSLLEVAGFS
HsLPLAT13_NP_620154    QKITSLACEI HDGMFRKDEE LTSSQRDL-- ---------- -----AVRRM PSLLEYLSYN
HsLPLAT14_NP_001073949 QKITTLAFQV HDGLGRRAED LSAEQHRL-- ---------- -----AIKVK PSFLEYLSYL
```

```
                       241
AqLPLAT3_XP_019855423  FSFMTFLAGP TVAYKNYDDF ITGTNFTQA- ---------- ANNKTTTEPS PLIPTVKTFL
NvLPLAT2_XP_048575357  YCYCGLLTGP YYRYKTYKDF LEQENPEK-- ---------- --------IA SLWPALRRLK
MgLPLAT5_XP_034333714  FCFIGQFAGP YYKYRTYQDM IYSPRTSE-- ---------- --------IP SMGPLIPRLK
MgLPLAT6_XP_011416786  FSFHNVMVGP TVFFNDYIKF IEGSDGS--- --------YE SPDKTPTEPN PEAAVREKLV
DmLPLAT4_NP_995637     FNYIGVLTGP YYRYRTYRDY FEMPFKTY-- ---------- --------AP TVEATLEKLK
DmLPLAT5_NP_524157     YFPSGFLVGP QFPFRRYKAF VDGEFRQ--- ---------- -------HEG NVEAGVRRFG
DmLPLAT6_NP_610546     WHFQSILAGP LVFYKDYIEF VEGYNLLST- -PPGNGNLDS SKREVVLEPS PTKAVIRKVV
CeLPLAT10_NP_001370561 FNFQTVLTGP ANNYYDYIKF LDEKHLVA-- ---------- -D-KHGKLPS PTGAAMKKFY
CeLPLAT11_NP_001022735 LFFQGTLVGP QFTLSKFRSF VNGDWLDS-- ---------- ---DGQPPKS AFLPSIGRFL
CeLPLAT12_NP_001255544 FNFQTVIVGP MNHYSTWSAF LELKHVPK-- ---------- -NEKTGKPYD PTSTAMKKFE
CtLPLAT4_ELT92151      FCYIGILTGP YYKYRTYHDM LHNSNAKN-- ---------- --------ID TIVPLWRRIR
CtLPLAT5_ELT92172      YFFGAFLTGP QFSFKRYMNF VNGAYSDS-- ---------- ---ITGGPPR SVMPALQRLM
DrLPLAT11_NP_956831    YCYVGIMTGP FFRYQTYADW LQQSSPLS-- ---------- --------LP GKEPCLQRLK
DrLPLAT12_XP_073781106 YFYGGFLVGP QFTLRNYQML VSREMTD--- ---------- ---VPGQPPN SVVPALKRFC
DrLPLAT13_XP_073783893 CNFMGILAGP TCSYNDYIDF IEGRGYESKH LET---NGKE NGKFKQIEPS PKRDVIQKVC
DrLPLAT14_NP_001007397 INFLSVLVGP CSNYKDYEEF IKGQHVQHRL KRVKDGSLQQ NGWDKVPEPS PVRTVITKVL
DrLPLAT15_NP_001265531 CNFMGILAGP TCSYNDYIAF IEGTPYRHRD LEP---KGKV NGKSRLNDPS PNTEVIRKLS
XtLPLAT11_XP_012822891 YCYIGLMTGP FYRYKTYYDW LHQIDSLS-- ---------- --------IP SWKPLVTRLK
XtLPLAT12_XP_002941254 YYYGGFLVGP QFSMCSYLKL VRGELTD--- ---------- ---VPGQRPN SIEPAMKRLS
XtLPLAT13_NP_001096155 CNFMGILAGP LCSYKDYIAF IEGRSYQLKQ FEA---NGKE DPKHEQKEPC PNSAVIHKVI
XtLPLAT14_XP_002940346 LNFMSVLAGP CSNFQDYIAF IEGRHIQSKL LN-----SKG NGYTKLPNPS PNEVVIYKLC
AcLPLAT11_XP_062840076 FCYVGLMTGP FFRYRTHYDW LNQPDSSA-- ---------- --------IP SWQPLLSRAK
AcLPLAT12_XP_003223803 YFYGAFMVGP QFSMTRYQKL VKGELTD--- ---------- ---VPGQRPN SFLPALNRMM
AcLPLAT13_XP_008115909 CNFMGILAGP LCSYKDYITF IEGRSYQPAQ SEA---NGKE EIKYEQTDPS PNIAVAQKLI
AcLPLAT14_XP_003219829 LNFLSILAGP CSNYKDYIAF IEGRHVQMKL LEV---NWKE RNCERLPDPS PVGAVLHKLG
GgLPLAT12_XP_040517541 YFYGAFMVGP QFSMTDYQKL AKGEMTD--- ---------- ---VPGQRPN SFVPALKRLS
GgLPLAT13_NP_001026261 CNFMGILAGP LCSYKDYITF IEGRSYQLQQ SEA---NGKE DTKYEQTDPS PNIAVAQKLL
GgLPLAT14_XP_046767333 LNFMSIIAGP CSNYKDYIAF IEGRHVHMKL LEV---NWKQ KGYDRLPDPS PTGAVMYKLC
HsLPLAT11_NP_077274    YCYVGIMTGP FFRYRTYLDW LEQPFPGA-- ---------- --------VP SLRPLLRRAW
HsLPLAT12_NP_005759    YFYGAFLVGP QFSMNHYMKL VQGELID--- ---------- ---IPGKIPN SIIPALKRLS
HsLPLAT13_NP_620154    CNFMGILAGP LCSYKDYITF IEGRSYHITQ SGE---NGKE ETQYERTEPS PNTAVVQKLL
HsLPLAT14_NP_001073949 LNFMSVIAGP CNNFKDYIAF IEGKHIHMKL LEV---NWKR KGFHSLPEPS PTGAVIHKLG

                       301
AqLPLAT3_XP_019855423  LSMTALSIVI LSSSWTDTSL LLDPDL---- Y-ILRRLINM LMF---CLAM RSQYFFTFLL
NvLPLAT2_XP_048575357  FAPLYGGLYL LMNTYFPTKH LMSEEFFKHP WGIPYQLLYL VPA---FNGF RWRFYIGWLL
MgLPLAT5_XP_034333714  QLPLIGVTYL FFSYFFNIQY TQTEEFYEHP F--WFRLFYM VPM---FTIF RTRLYMAWIL
MgLPLAT6_XP_011416786  VAAISGAVMV LAPLFIPVNY MTDEEFLYGT N-WVYRNLYV LIC---VSLV RGKYYCAWKL
DmLPLAT4_NP_995637     YAVFYCALYL ATNYMWPLDY ALSDEFFNDR S-FVYRLLYV WPT---FFTF RARIYTGLTL
DmLPLAT5_NP_524157     AGAFYLIVCQ VGLRYLPDSY FLTPEFAQVS --FVKRIYLL GFW---AKFS LYKYISCWLL
DmLPLAT6_NP_610546     GSLVCAFIFM KFVKIYPVKD MKEDDFMNNT S-MVYKYWYA MMA---TTCI RFKYYHAWLL
CeLPLAT10_NP_001370561 QSLFFLAIVV TLGGKYKVED VGTQPYFALP --FFQWFFWW FITIF---FI RCAYYFAWVF
CeLPLAT11_NP_001022735 AGCTYMVLHQ WGQFWIPDQY FNSDAYNNLS --FFWRWSWV TLW---FRLT MYKYCAMWLI
CeLPLAT12_NP_001255544 AAIAFSVVYT ILG-SYLPMS LTNDP--SIN --EYNLLIWW LITVAASTVH RLPYYFAWTI
CtLPLAT4_ELT92151      VLPLYFLPYL IGSQFFSLDY IRRGDLFADP F--WFRLLYM AAL---SFIF RLRFYCGWIL
CtLPLAT5_ELT92172      AGVLYTLVYQ VSDLIIPDSF LLSQSFADMT --IITKCLVI TLW---GKLM LSKYVGCWLI
DrLPLAT11_NP_956831    MVPVYGLLFI AVNSVFPLSY VRTEDFLEHN Y--FYRFFYM VAI---FFVF RMRFYSAWCG
DrLPLAT12_XP_073781106 LGLITLSIFT IGGPRYSDSY YLTDEFAAHP --FWYKCVYI LLW---VKIN LYKYISCWLI
DrLPLAT13_XP_073783893 TCALCLTIYL VIHTACPTER VTHESFMASN P-FYIQVLFL YIS---MLSL RPKYYFVWTL
DrLPLAT14_NP_001007397 VCVCCLLWFF IITRAFPIKY NVDPQFITEA S-FISRLAYA FVS---IQAA RPKFYFAWTL
DrLPLAT15_NP_001265531 TCFISLLVFL SVCKVFPVER NVDDDFIATS P-FYVQVVYL YLS---MLTT RPKYYFVWTL
XtLPLAT11_XP_012822891 PAPVFGVLFL IASKYFPLEY VKTDDFYEQA F--LYRLFYM VPT---FFIF RMRFYVAWIF
XtLPLAT12_XP_002941254 LGLFCLVIYT VFGPMLPDSY FLTDEYANKP --FWYRCAYV PIW---GKVM LYKYVTCWLV
XtLPLAT13_NP_001096155 VSAVSLAFYM TATRILPVEY NIDEQFQETS S-VLNRIIYL YLS---LMAA RPKYYFAWTL
XtLPLAT14_XP_002940346 IAAASLAVFM TFTKAFPILY VVDETFMNTV P-FLKRLGYF YIA---TQAC KPKYYFAWTL
AcLPLAT11_XP_062840076 YIPVFGALFL AVSHFFPLNY VRSDAFYERA L--PFRLFYM VPI---FFVF RMRFYVAWLC
AcLPLAT12_XP_003223803 LGLFFMVVYT VAGPYFPEEY MVSDDYLNQP --FWFRCVYI LMW---GKVM LNKYVTCWLI
AcLPLAT13_XP_008115909 VCGLSLLFHM TVSKLLPVEY NIDDQFRATA S-LPTRVIYL YLS---LMAA RPKYYFAWTL
AcLPLAT14_XP_003219829 ITLVSLLLFL TLSKNFPATS LVDDQFINKS S-FLSRLGFL YLV---MQAS KPKYYFAWTL
GgLPLAT12_XP_040517541 LGLLFLVTYT LSSPYISEEY LISDDYMEKP --FWFRCGYI LVW---GKII LYKYVTCWLV
GgLPLAT13_NP_001026261 ICGLSLLFHM TITKTLPVEY NIDENFRATA S-WPVRVFYL YLS---LMAA RPKYYFAWTL
GgLPLAT14_XP_046767333 ITLISLVLFL TLTKNFPMAY IIDNEFLEKT P-FLTRLGYL YVV---TQSA KPKYYFAWTL
HsLPLAT11_NP_077274    PAPLFGLLFL LSSHLFPLEA VREDAFYARP L--PARLFYM IPV---FFAF RMRFYVAWIA
HsLPLAT12_NP_005759    LGLFYLVGYT LLSPHITEDY LLTEDYDNHP --FWFRCMYM LIW---GKFV LYKYVTCWLV
HsLPLAT13_NP_620154    VCGLSLLFHL TICTTLPVEY NIDEHFQATA S-WPTKIIYL YIS---LLAA RPKYYFAWTL
HsLPLAT14_NP_001073949 ITLVSLLLFL TLTKTFPVTC LVDDWFVHKA S-FPARLCYL YVV---MQAS KPKYYFAWTL
```

```
                       361
AqLPLAT3_XP_019855423  TESISNAGGL GFTGYD---- ---------- ---------- ---KE-GREV WDLAKNIDLR
NvLPLAT2_XP_048575357  AESSCIMLGL GAYPFETDPK PALGPSKPMP AENGHVSEVA DTKENGDTHS FMTVYNIDIF
MgLPLAT5_XP_034333714  SESMCMTSGL GAYPEASQPK CGQGPTDL-K KL----HESY ES-KDSVDYN YETIHSLSIY
MgLPLAT6_XP_011416786  GEAVNNAAGL GFNGYD---- ---------- ---------- ---DK-GNAK WDLLNNVKIY
DmLPLAT4_NP_995637     SECVCTMAGF GAYPDESDPN NGEGPRKRYQ HL----K--- -RDADKHTYN FTTIVNTRVL
DmLPLAT5_NP_524157     TEGALICIGL TYKGED---- ---------- ---------- ---KN-GQPD WSGCSNVKLK
DmLPLAT6_NP_610546     ADAICNNSGL GFTGYD---- ---------- ---------- ---KD-GNSK WDLISNINVL
CeLPLAT10_NP_001370561 ADAICNMSGF GFSGYD---- ---------- ---------- ---KE-GNAE WKLCTNVLPY
CeLPLAT11_NP_001022735 TEGASILSGL GHNGKD---- ---------- ---------- ---AE-GNDR WDGVRDLHII
CeLPLAT12_NP_001255544 SDSICNISGF GYDGLA---- ---------- ---------- ---DETLEPK WSRTTNVKPL
CtLPLAT4_ELT92151      AECGCMTAGL GAYPAVTKPR CGAGPTDF-E AF----EECY EMAPEGIAYN FETIHNIDEY
CtLPLAT5_ELT92172      TEGVCIMAGL TYNGRD---- ---------- ---------- ---EN-GNCL WNGCANIHAV
DrLPLAT11_NP_956831    AEAGCISAGL GCYPQGALSK PGGGPTVKYS PD----P--- ---ETAVEYD FKTIQNIDCY
DrLPLAT12_XP_073781106 TEGVCVLAGL GYNGKN---- ---------- ---------- ---EK-GEHQ WDACANVRVW
DrLPLAT13_XP_073783893 ADAINNAAGF GFNGYN---- ---------- ---------- ---TD-GTPK WDRISNLRIL
DrLPLAT14_NP_001007397 ADAVHNAAGY GVSGVN---- ---------- ---------- ---ER-GEVS WDLVSNVRIW
DrLPLAT15_NP_001265531 ADAINNAAGF GFNGYD---- ---------- ---------- ---SD-GSPR WDLLSNIRIL
XtLPLAT11_XP_012822891 AECGCIAAAF GAYPVTAKSR PGGGPTVEYS PL----ERNP DGAKVELEYD YETIKNIDSY
XtLPLAT12_XP_002941254 TEGVCILSGL GYNGKD---- ---------- ---------- ---DT-GRVR WDACANMKVW
XtLPLAT13_NP_001096155 ADAINNAAGF GFQGYD---- ---------- ---------- ---KN-GEEN WDLVSNLNIK
XtLPLAT14_XP_002940346 ADAVNNAAGY GFNGVD---- ---------- ---------- ---EK-GKFR WDLISNLNIW
AcLPLAT11_XP_062840076 AECACIAAAF GAYPTAAKAR SGGGPTLEYE PL----SKSA DGEASEVTYD YETIKNIDPH
AcLPLAT12_XP_003223803 TEGVCILTGL GYNGKD---- ---------- ---------- ---EK-GKPL WNACANMKVW
AcLPLAT13_XP_008115909 ADAINNAAGF GFRGYD---- ---------- ---------- ---KN-GVSH WDSISNLRIL
AcLPLAT14_XP_003219829 ADAIHNAAGY GFSGVD---- ---------- ---------- ---EK-GNFH WDLLSNLNIR
GgLPLAT12_XP_040517541 TEGVCILVGL GYNGND---- ---------- ---------- ---QN-GKPV WDACANMKVW
GgLPLAT13_NP_001026261 ADAINNAAGF GFRGYD---- ---------- ---------- ---KN-GVTR WDLISNLRIQ
GgLPLAT14_XP_046767333 ADAVNNAAGY GFSGVD---- ---------- ---------- ---ER-GAFR WDLLSNLNIW
HsLPLAT11_NP_077274    AECGCIAAGF GAYPVAAKAR AGGGPTLQCP PP----SSP- -EKAASLEYD YETIRNIDCY
HsLPLAT12_NP_005759    TEGVCILTGL GFNGFE---- ---------- ---------- ---EK-GKAK WDACANMKVW
HsLPLAT13_NP_620154    ADAINNAAGF GFRGYD---- ---------- ---------- ---EN-GAAR WDLISNLRIQ
HsLPLAT14_NP_001073949 ADAVNNAAGF GFSGVD---- ---------- ---------- ---KN-GNFC WDLLSNLNIW
```

MBOAT motif: (A) WD

```
                       421
AqLPLAT3_XP_019855423  GFELGANPRE MTSSWNMKTV QWLRRVCYDR SHS---V--- -LLTNMLSAL WHGFYPGYYL
NvLPLAT2_XP_048575357  KVEFAPSMRS IMKDWNMSVQ WWIAQYVHRK LP-FSNRNIR MSVTLLVSAF WHGVAPGYYL
MgLPLAT5_XP_034333714  GCELGRTTKE GLRSWNMTVQ YWLATYCHRR VP-NSLKAYR VAITMTVSAF WHGIYPGYYL
MgLPLAT6_XP_011416786  ELEVSNSLKV NIDCWNITTL VWLRRVIYDR VPPKYRT--- -VSTFMVSAL WHGFYPGYYI
DmLPLAT4_NP_995637     EVERCWTFRE GMKHWNVCVQ YWLAVNVYKL FP---SKKYR TGATLLCSAY WHGFRPGHYF
DmLPLAT5_NP_524157     LLETGNTMEH YVQSFNVNTN QWVGQYIYKR LKFLNNRTIS YGAALGFLAV WHGYHSGYYM
DmLPLAT6_NP_610546     SFEFSTNMRD AINNWNCGTN RWLRTLVYER VPQQYGT--- -LLTFALSAV WHGFYPGYYL
CeLPLAT10_NP_001370561 QVEMAQSLKE TLDGWNIQTG FWLRKVGYER APKSIRT--- -VATYTLSAV WHGVSIGYYM
CeLPLAT11_NP_001022735 KWETGHDYNS VVESFNCGTN TFAKNHIHRR LRWVNNKLAS HVITLSYLAI WHGYHLGYFL
CeLPLAT12_NP_001255544 LVEFGQNYKE MVDNWNIWTV AWLRRVVYER VEGPYRT--- -LAVYVTGAA WHGLAVGYYF
CtLPLAT4_ELT92151      GCETAVTNRR KMHHWNKSVQ YWLSSNIYKR LTLVKSGALK VAITMTVSAF WHGIHPGYFF
CtLPLAT5_ELT92172      EFEKASTCQE YIQAFNVNTN KWMAKYIFKR LKWLGNRHIS QFATLFFLAL WHGLHSGYYN
DrLPLAT11_NP_956831    NTDFCVKVRH GMRYWNMTVQ WWLHHYIYPN AP-FRAYALR AGWTMLISAY WHGLHAGYYL
DrLPLAT12_XP_073781106 LFETTPLFTG TINSFNINTN AWVARHVFKR LRFLGNKLVS QMFTLLFLAI WHGLHSGYLI
DrLPLAT13_XP_073783893 NIEFATSFKM FLDNWNIQTA LWLKRVCYER CPY-NPT--- -AATFLLSAM WHGVYPGYYL
DrLPLAT14_NP_001007397 EIETATSFKS FIDNWNIQTG LWLKRVCYDR APF-YRT--- -PLTFVLSAI WHGVYPGYYF
DrLPLAT15_NP_001265531 NIELATSFKV FLDNWNIQTA HWLKRVCYER CPY-NPT--- -AATFLLSAM WHGAYPGYYL
XtLPLAT11_XP_012822891 GADFCVKVKD GMRCWNMSVQ WWLAQYIYKN SP-VKSFVFG SAWTMLISAY WHGIHPGYYM
XtLPLAT12_XP_002941254 QYETTPLFTG TIASFNINTN AWVARYVFKR LRFLGNKAIS QATALFFLAI WHGLHSGYFV
XtLPLAT13_NP_001096155 QIEFSTSFKM FIDNWNIQTA LWLKRVCYER ATY-SAT--- -AKTFLLSAI WHGVYPGYYM
XtLPLAT14_XP_002940346 NIETATSFKM YIDNWNIQTA AWLKRVCYDR APK-YRT--- -GLTFILSGV WHGVYPGYYF
AcLPLAT11_XP_062840076 GTDFCVKVKD GMRYWNMSVQ WWLAQYIYKN AP-VRSYVMR SAWTMLISAY WHGIHPGYYL
AcLPLAT12_XP_003223803 LFETTPYFTG TIASFNTNTN DWVARYIYKR LKFLGNKLLS QALSLLFLAI WHGLHCGYLV
AcLPLAT13_XP_008115909 HIEFSTSFKM FLDNWNIQTA FWLKRVCYER ASF-SPT--- -IQTFILSAI WHGVYPGYYL
AcLPLAT14_XP_003219829 NIEMATSFKM YIENWNIQTT AWLKRVCYDR APQ-YRT--- -ALTFLLSAI WHGVYPGYYF
GgLPLAT12_XP_040517541 LYETTPLFTG TIASFNINTN AWVARYVFKR LKFLGNKLLS QALALFFLAI WHGLHSGYLV
GgLPLAT13_NP_001026261 QIESSTSFKM FLDNWNIQTA LWLKRVCYER ATF-SPT--- -IQTFILSAI WHGVYPGYYL
GgLPLAT14_XP_046767333 NIETATSFKM YIENWNIQTA AWLKRVCYDR APW-YPT--- -ALTFVLSAL WHGIYPGYYF
HsLPLAT11_NP_077274    STDFCVRVRD GMRYWNMTVQ WWLAQYIYKS AP-ARSYVLR SAWTMLLSAY WHGLHPGYYL
HsLPLAT12_NP_005759    LFETNPRFTG TIASFNINTN AWVARYIFKR LKFLGNKELS QGLSLLFLAL WHGLHSGYLV
HsLPLAT13_NP_620154    QIEMSTSFKM FLDNWNIQTA LWLKRVCYER TSF-SPT--- -IQTFILSAI WHGVYPGYYL
HsLPLAT14_NP_001073949 KIETATSFKM YLENWNIQTA TWLKCVCYQR VPW-YPT--- -VLTFILSAL WHGVYPGYYF
```

MBOAT motif: (B) WHGlhsGYlv

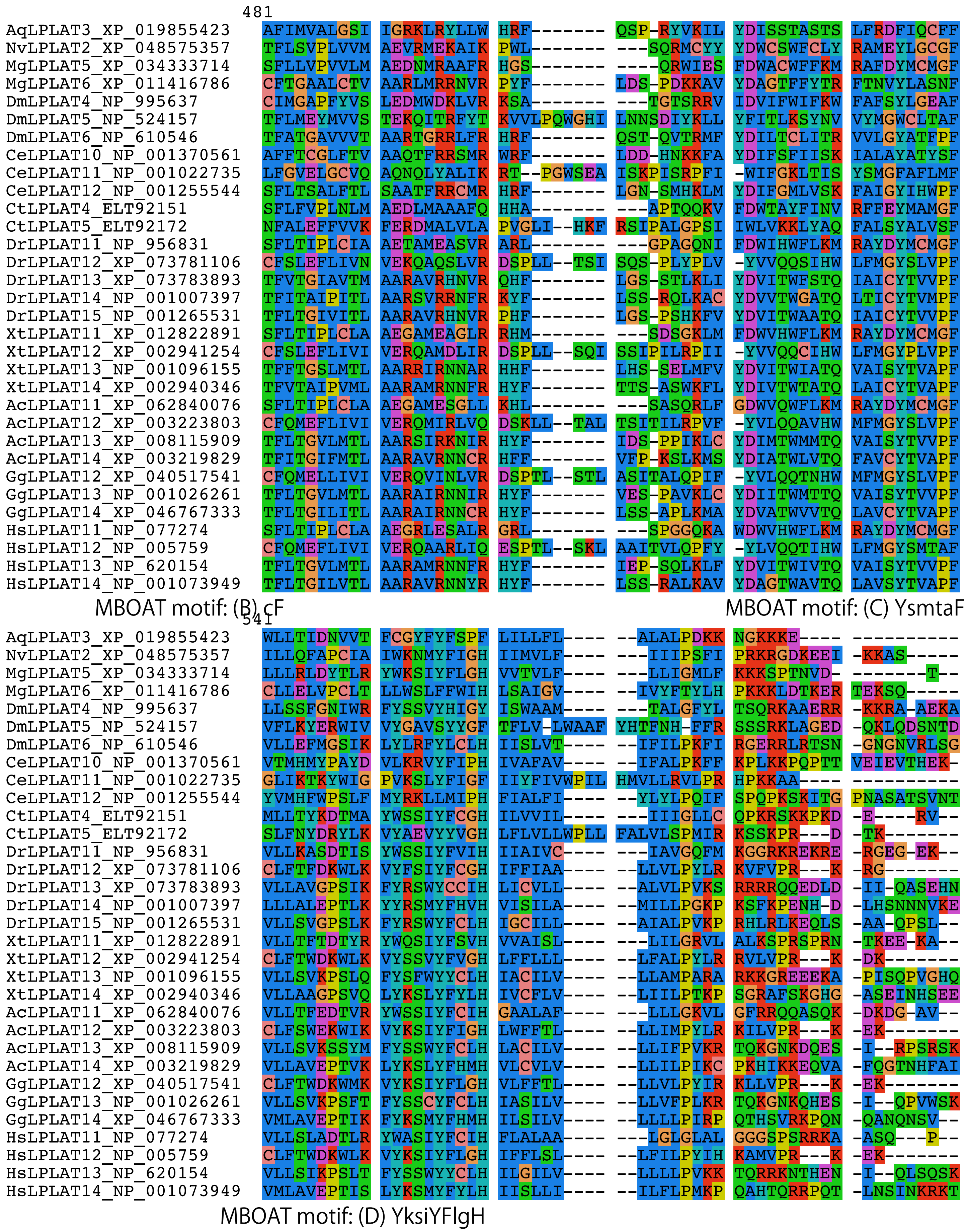
481
AqLPLAT3_XP_019855423
NvLPLAT2_XP_048575357
MgLPLAT5_XP_034333714
MgLPLAT6_XP_011416786
DmLPLAT4_NP_995637
DmLPLAT5_NP_524157
DmLPLAT6_NP_610546
CeLPLAT10_NP_001370561
CeLPLAT11_NP_001022735
CeLPLAT12_NP_001255544
CtLPLAT4_ELT92151
CtLPLAT5_ELT92172
DrLPLAT11_NP_956831
DrLPLAT12_XP_073781106
DrLPLAT13_XP_073783893
DrLPLAT14_NP_001007397
DrLPLAT15_NP_001265531
XtLPLAT11_XP_012822891
XtLPLAT12_XP_002941254
XtLPLAT13_NP_001096155
XtLPLAT14_XP_002940346
AcLPLAT11_XP_062840076
AcLPLAT12_XP_003223803
AcLPLAT13_XP_008115909
AcLPLAT14_XP_003219829
GgLPLAT12_XP_040517541
GgLPLAT13_NP_001026261
GgLPLAT14_XP_046767333
HsLPLAT11_NP_077274
HsLPLAT12_NP_005759
HsLPLAT13_NP_620154
HsLPLAT14_NP_001073949
MBOAT motif: (B) cF
MBOAT motif: (C) YsmtaF
541
MBOAT motif: (D) YksiYFlgH

```
                          601
AqLPLAT3_XP_019855423     ---------- ---------- ---------- ---------- ---------- ----------
NvLPLAT2_XP_048575357     ---------- ---------- ---------- ---------- ---------- ----------
MgLPLAT5_XP_034333714     ---------- ---------- -------SSK MKEKVKSN-- ---------- ----------
MgLPLAT6_XP_011416786     ---------- ---------- ---------- ---------- ---------- ----------
DmLPLAT4_NP_995637        A--------- ---------- -GGDGIAAAI EKEKAQ---- ---------- ----------
DmLPLAT5_NP_524157        KL-------- ---------- -----V-EEK KPEDKKSE-- ---------- ----------
DmLPLAT6_NP_610546        SGNTKDA--V TTSVESTAAL TAGNDL-NED KEEDKHAQCK VHTPTQQQPA AGPHKTTVEQ
CeLPLAT10_NP_001370561    -----PIKAE ---------- ---------- ---------- ---------- ----------
CeLPLAT11_NP_001022735    ---------- ---------- --------AE KPEEVKKEL- ---------- ----------
CeLPLAT12_NP_001255544    DR-ISEISQR SSSRTS---- --AIDV-STR TALEK----- ---------- ----------
CtLPLAT4_ELT92151         ---------- ---------- -------SEE AKKKL----- ---------- ----------
CtLPLAT5_ELT92172         ---------- ---------- ---------- --EAKKTE-- ---------- ----------
DrLPLAT11_NP_956831       ---------- ---------- -------EDA VREKAE---- ---------- ----------
DrLPLAT12_XP_073781106    ---------- ---------- ---------- --SEKKED-- ---------- ----------
DrLPLAT13_XP_073783893    CLSS------ ---------- -PDSNC-NQK Q----KSS-- ---------- ----------
DrLPLAT14_NP_001007397    D--------- ---------- ---------- ---------- ---------- ----------
DrLPLAT15_NP_001265531    --ES------ ---------- -TDSN---QK Q----KAT-- ---------- ----------
XtLPLAT11_XP_012822891    ---------- ---------- -------ETK QENRLQE--- ---------- ----------
XtLPLAT12_XP_002941254    ---------- ---------- ---------- --QKSQ---- ---------- ----------
XtLPLAT13_NP_001096155    RT-EDNI-SE QNSH-ST--- -TNNNS-NQR E----ETAWR RPSAAQ---- ----------
XtLPLAT14_XP_002940346    K--QYSAKHA NNHM-S---- ---------- ---------- ---------- ----------
AcLPLAT11_XP_062840076    ---------- ---------- -------ESL QHME------ ---------- ----------
AcLPLAT12_XP_003223803    ---------- ---------- ---------- --KAE----- ---------- ----------
AcLPLAT13_XP_008115909    KPEDNDV-EE KSSM-GHNSH FLANSF-NQK T----EFTYR NTATKQ---- ----------
AcLPLAT14_XP_003219829    N--RKQK--- ---------- ---------- ---------- ---------- ----------
GgLPLAT12_XP_040517541    ---------- ---------- ---------- --LKKAE--- ---------- ----------
GgLPLAT13_NP_001026261    KLEEENL-LQ KNSY-ST--- -TNNSF-SQK E----EITCR YQALKQ---- ----------
GgLPLAT14_XP_046767333    K--SKDK--- ---------- ---------- ---------- ---------- ----------
HsLPLAT11_NP_077274       ---------- ---------- -------TSL APEKLREE-- ---------- ----------
HsLPLAT12_NP_005759       ---------- ---------- ---------- --LKKME--- ---------- ----------
HsLPLAT13_NP_620154       KFDEGENSLG QNSF-ST--- -TNNVC-NQN Q----EIASR HSSLKQ---- ----------
HsLPLAT14_NP_001073949    D--------- ---------- ---------- ---------- ---------- ----------

                          661
AqLPLAT3_XP_019855423     ---------- ---------- ---------- ---------- ---------- ----------
NvLPLAT2_XP_048575357     ---------- ---------- ---------- ---------- ---------- ----------
MgLPLAT5_XP_034333714     ---------- ---------- ---------- ---------- ---------- ----------
MgLPLAT6_XP_011416786     ---------- ---------- ---------- ---------- ---------- ----------
DmLPLAT4_NP_995637        ---------- ---------- ---------- ---------- ---------- ----------
DmLPLAT5_NP_524157        ---------- ---------- ---------- ---------- ---------- ----------
DmLPLAT6_NP_610546        PTEQPNNVNL RSRPQQQQPH LEKKAMPPTC ARDAVSVPHD QCEMDQLSSK LKEKIEAETK
CeLPLAT10_NP_001370561    ---------- ---------- ---------- ---------- ---------- ----------
CeLPLAT11_NP_001022735    ---------- ---------- ---------- ---------- ---------- ----------
CeLPLAT12_NP_001255544    ---------- ---------- ---------- ---------- ---------- ----------
CtLPLAT4_ELT92151         ---------- ---------- ---------- ---------- ---------- ----------
CtLPLAT5_ELT92172         ---------- ---------- ---------- ---------- ---------- ----------
DrLPLAT11_NP_956831       ---------- ---------- ---------- ---------- ---------- ----------
DrLPLAT12_XP_073781106    ---------- ---------- ---------- ---------- ---------- ----------
DrLPLAT13_XP_073783893    ---------- ---------- ---------- ---------- ---------- ----------
DrLPLAT14_NP_001007397    ---------- ---------- ---------- ---------- ---------- ----------
DrLPLAT15_NP_001265531    ---------- ---------- ---------- ---------- ---------- ----------
XtLPLAT11_XP_012822891    ---------- ---------- ---------- ---------- ---------- ----------
XtLPLAT12_XP_002941254    ---------- ---------- ---------- ---------- ---------- ----------
XtLPLAT13_NP_001096155    ---------- ---------- ---------- ---------- ---------- ----------
XtLPLAT14_XP_002940346    ---------- ---------- ---------- ---------- ---------- ----------
AcLPLAT11_XP_062840076    ---------- ---------- ---------- ---------- ---------- ----------
AcLPLAT12_XP_003223803    ---------- ---------- ---------- ---------- ---------- ----------
AcLPLAT13_XP_008115909    ---------- ---------- ---------- ---------- ---------- ----------
AcLPLAT14_XP_003219829    ---------- ---------- ---------- ---------- ---------- ----------
GgLPLAT12_XP_040517541    ---------- ---------- ---------- ---------- ---------- ----------
GgLPLAT13_NP_001026261    ---------- ---------- ---------- ---------- ---------- ----------
GgLPLAT14_XP_046767333    ---------- ---------- ---------- ---------- ---------- ----------
HsLPLAT11_NP_077274       ---------- ---------- ---------- ---------- ---------- ----------
HsLPLAT12_NP_005759       ---------- ---------- ---------- ---------- ---------- ----------
HsLPLAT13_NP_620154       ---------- ---------- ---------- ---------- ---------- ----------
HsLPLAT14_NP_001073949    ---------- ---------- ---------- ---------- ---------- ----------
```

```
                          721
AqLPLAT3_XP_019855423   ---------- ---------- ---------- ---------- ---------- ----------
NvLPLAT2_XP_048575357   ---------- ---------- ---------- ---------- ---------- ----------
MgLPLAT5_XP_034333714   ---------- ---------- ---------- ---------- ---------- ----------
MgLPLAT6_XP_011416786   ---------- ---------- ---------- ---------- ---------- ----------
DmLPLAT4_NP_995637      ---------- ---------- ---------- ---------- ---------- ----------
DmLPLAT5_NP_524157      ---------- ---------- ---------- ---------- ---------- ----------
DmLPLAT6_NP_610546      NIEEFIDKTV TETVSGIVEF KNDLMRDIEF PKLKLPGSNG AISLDSSNGG GLRKRNISSV
CeLPLAT10_NP_001370561  ---------- ---------- ---------- ---------- ---------- ----------
CeLPLAT11_NP_001022735  ---------- ---------- ---------- ---------- ---------- ----------
CeLPLAT12_NP_001255544  ---------- ---------- ---------- ---------- ---------- ----------
CtLPLAT4_ELT92151       ---------- ---------- ---------- ---------- ---------- ----------
CtLPLAT5_ELT92172       ---------- ---------- ---------- ---------- ---------- ----------
DrLPLAT11_NP_956831     ---------- ---------- ---------- ---------- ---------- ----------
DrLPLAT12_XP_073781106  ---------- ---------- ---------- ---------- ---------- ----------
DrLPLAT13_XP_073783893  ---------- ---------- ---------- ---------- ---------- ----------
DrLPLAT14_NP_001007397  ---------- ---------- ---------- ---------- ---------- ----------
DrLPLAT15_NP_001265531  ---------- ---------- ---------- ---------- ---------- ----------
XtLPLAT11_XP_012822891  ---------- ---------- ---------- ---------- ---------- ----------
XtLPLAT12_XP_002941254  ---------- ---------- ---------- ---------- ---------- ----------
XtLPLAT13_NP_001096155  ---------- ---------- ---------- ---------- ---------- ----------
XtLPLAT14_XP_002940346  ---------- ---------- ---------- ---------- ---------- ----------
AcLPLAT11_XP_062840076  ---------- ---------- ---------- ---------- ---------- ----------
AcLPLAT12_XP_003223803  ---------- ---------- ---------- ---------- ---------- ----------
AcLPLAT13_XP_008115909  ---------- ---------- ---------- ---------- ---------- ----------
AcLPLAT14_XP_003219829  ---------- ---------- ---------- ---------- ---------- ----------
GgLPLAT12_XP_040517541  ---------- ---------- ---------- ---------- ---------- ----------
GgLPLAT13_NP_001026261  ---------- ---------- ---------- ---------- ---------- ----------
GgLPLAT14_XP_046767333  ---------- ---------- ---------- ---------- ---------- ----------
HsLPLAT11_NP_077274     ---------- ---------- ---------- ---------- ---------- ----------
HsLPLAT12_NP_005759     ---------- ---------- ---------- ---------- ---------- ----------
HsLPLAT13_NP_620154     ---------- ---------- ---------- ---------- ---------- ----------
HsLPLAT14_NP_001073949  ---------- ---------- ---------- ---------- ---------- ----------

                          781
AqLPLAT3_XP_019855423   ---------- ---------- ---------- ---------- ---------- -
NvLPLAT2_XP_048575357   ---------- ---------- ---------- ---------- ---------- -
MgLPLAT5_XP_034333714   ---------- ---------- ---------- ---------- ---------- -
MgLPLAT6_XP_011416786   ---------- ---------- ---------- ---------- ---------- -
DmLPLAT4_NP_995637      ---------- ---------- ---------- ---------- ---------- -
DmLPLAT5_NP_524157      ---------- ---------- ---------- ---------- ---------- -
DmLPLAT6_NP_610546      HDNGTDPGHA TADLHPPLEE NGAAFLKKEI EVINAVVQQA VPAVLSNGHA K
CeLPLAT10_NP_001370561  ---------- ---------- ---------- ---------- ---------- -
CeLPLAT11_NP_001022735  ---------- ---------- ---------- ---------- ---------- -
CeLPLAT12_NP_001255544  ---------- ---------- ---------- ---------- ---------- -
CtLPLAT4_ELT92151       ---------- ---------- ---------- ---------- ---------- -
CtLPLAT5_ELT92172       ---------- ---------- ---------- ---------- ---------- -
DrLPLAT11_NP_956831     ---------- ---------- ---------- ---------- ---------- -
DrLPLAT12_XP_073781106  ---------- ---------- ---------- ---------- ---------- -
DrLPLAT13_XP_073783893  ---------- ---------- ---------- ---------- ---------- -
DrLPLAT14_NP_001007397  ---------- ---------- ---------- ---------- ---------- -
DrLPLAT15_NP_001265531  ---------- ---------- ---------- ---------- ---------- -
XtLPLAT11_XP_012822891  ---------- ---------- ---------- ---------- ---------- -
XtLPLAT12_XP_002941254  ---------- ---------- ---------- ---------- ---------- -
XtLPLAT13_NP_001096155  ---------- ---------- ---------- ---------- ---------- -
XtLPLAT14_XP_002940346  ---------- ---------- ---------- ---------- ---------- -
AcLPLAT11_XP_062840076  ---------- ---------- ---------- ---------- ---------- -
AcLPLAT12_XP_003223803  ---------- ---------- ---------- ---------- ---------- -
AcLPLAT13_XP_008115909  ---------- ---------- ---------- ---------- ---------- -
AcLPLAT14_XP_003219829  ---------- ---------- ---------- ---------- ---------- -
GgLPLAT12_XP_040517541  ---------- ---------- ---------- ---------- ---------- -
GgLPLAT13_NP_001026261  ---------- ---------- ---------- ---------- ---------- -
GgLPLAT14_XP_046767333  ---------- ---------- ---------- ---------- ---------- -
HsLPLAT11_NP_077274     ---------- ---------- ---------- ---------- ---------- -
HsLPLAT12_NP_005759     ---------- ---------- ---------- ---------- ---------- -
HsLPLAT13_NP_620154     ---------- ---------- ---------- ---------- ---------- -
HsLPLAT14_NP_001073949  ---------- ---------- ---------- ---------- ---------- -
```

Supplement Figure 2B. Sequence alignment utilized in Figure 3B.
MBOAT motifs in HsLPLAT12 were shown.

Supplementary Fig. 3A

```
                       1
EckLPLAT1_AIZ52813     ---------- ---------- ---------- ---------- ---------- ----------
EckLPLAT2_AKK15969     ---------- ---------- ---------- ----MRFFSA L----SMANL LNKFIMTRI-
EcoLPLAT1_EFX09666     ---------- ---------- ---------- ---------- ---------- ----------
EcoLPLAT2_EFX08826     ---------- ---------- ---------- ---------- ------MANL LNKFIMTRI-
EcoLPLAT3_EFX09355     ---------- ---------- ---------- ------MS-- --KLMSRLEW TWRLVMTGLC
EdLPLAT1_XP_001739767  MILYFRQPSK RVKKMIR--- ---------- GESSERMVDT TGIKDKIILI LKYYFAYC--
EdLPLAT2_XP_001734633  ---------- ---------- ---------- -------MKC NELINKICWG LKIYFAYG--
EhLPLAT1_XP_655766     ---------- ---------- ---------- ------MVDT TNITHKIMLI LKYYFAYC--
EhLPLAT2_XP_649422     ---------- ---------- ---------- -------MKY SELINKICWG LKLYFAYG--
HsLPLAT1_NP_006402     ---------- ---------- ---------- ---------- ----MDLWPG AWMLLLL---
HsLPLAT2_NP_006403     ---------- ---------- ---------- ---------- ----MELWPC LAAALLL---
HsLPLAT3_NP_064517     ---------- ---------- ---------- ---------- ----MGLLAF LKTQFVLHL-
HsLPLAT4_NP_064518     ---------- ---------- ---------- ---------- ----MDLAGL LKSQFLCHL-
HsLPLAT5_NP_060831     ---------- ---------- ---------- ---------- ----MLLSLV LHTYSMRY--
HsLPLAT6_NP_001002257  ---------- ---------- ---------- ---------- ------MVSW KGIYFILTL-
HsLPLAT7_NP_055688     ---------- ---------- ---------- ---------- MAITLEEAPW LGWLLVKA--
HsLPLAT8_NP_079106     MRLRGCGPRA AP-------- ------ASSA GASDARLL-- --APPGRNPF VHELRLSALQ
HsLPLAT9_NP_060309     --MSRCAQAA EVAATVPGAG VGNVGLRPPM VPRQASFF-- --PPPVPNPF VQQTQIGSAR
HsLPLAT10_NP_705841    ---------- ---------- -MSQGSPGDW APLDPTPG-- --PPASPNPF VHELHLSRLQ

                       61
EckLPLAT1_AIZ52813     ---------- ---------- -MLYIFRLII TVIYSILVCV FGSIYCLF-- -------SPR
EckLPLAT2_AKK15969     -----LAAIT LLLSIVLT-- -----ILVTI FCSVPIIIAG IVKLLLPVPV IW---RK-VS
EcoLPLAT1_EFX09666     ---------- ---------- -MLYIFRLII TVIYSILVCV FGSIYCLF-- -------SPR
EcoLPLAT2_EFX08826     -----LAAIT LLLSIVLT-- -----ILVTI FCSVPIIIAG IVKLLLPVPV IW---RK-VS
EcoLPLAT3_EFX09355     FA-LFGLGGL LLSVVWF--- ---------- ---------- -NILLVLVWD -----TSRRR
EdLPLAT1_XP_001739767  ---------- FVASVF---- -----L-Q-- ----ITILLI MDVICLPFYF IY---RPIYQ
EdLPLAT2_XP_001734633  ---------- FLMSIF---- -----I-Q-- ----ITILLI INVIILPIYI LY---RPLYQ
EhLPLAT1_XP_655766     ---------- FVASVF---- -----L-Q-- ----ITVLLI MDVICLPLYF IY---RPIYQ
EhLPLAT2_XP_649422     ---------- FLMSIF---- -----I-Q-- ----ITILLI INVIILPTYI LY---RPIYQ
HsLPLAT1_NP_006402     ---LFLLLLF LLPTLWFCSP SAKYFFKMAF YNGWILFLAV LAIPVCAV-- -------RGR
HsLPLAT2_NP_006403     ---LLLLVQ- -------LSR AAEFYAKVAL YCALCFTVSA VASLVCLLRH -------GGR
HsLPLAT3_NP_064517     -----LVG-- ---FVF---- -----VVS-- ----GLVINF VQLCTLALWP VS---KQLYR
HsLPLAT4_NP_064518     -----VFC-- ---YVF---- -----IAS-- ----GLIINT IQLFTLLLWP IN---KQLFR
HsLPLAT5_NP_060831     ---------- LLPSVV---- -----LLGT- ---APTYVLA WGVWRLLSAF LP---ARFYQ
HsLPLAT6_NP_001002257  -----FWG-S FFGSIF---- -----MLS-- ----P----- ----FLPLMF VN---PSWYR
HsLPLAT7_NP_055688     ---------- LMRFAF---- -----MVVNN LVAIPSYICY VI-ILQPLRV L----DSKRF
HsLPLAT8_NP_079106     KAQVALMTLT LFPVRLLV-- -----AAAMM LLAWPLA--L VASLGS-AEK EPEQPPALWR
HsLPLAT9_NP_060309     RVQIVLLGII LLPIRVLL-- -----VALIL LLAWPFA--A ISTVCC-PEK LTHPITGWRR
HsLPLAT10_NP_705841    RVKFCLLGAL LAPIRVLL-- -----AFIVL FLLWPFA--W LQVAGLSEEQ LQEPITGWRK

                       121
EckLPLAT1_AIZ52813     NP---KHVAT FGHMFGRLAP LFGLKVECRK P---TDAESY GNAIYIANHQ NNYDMVTASN
EckLPLAT2_AKK15969     RFCDFMMYCW CEGLAVLLHL NPHLQWEVHG L---EGLSKK NWYLLICNHR SWADIVVLCV
EcoLPLAT1_EFX09666     NP---KHVAT FGHMFGRLAP LFGLKVECRK P---ADAESY GNAIYIANHQ NNYDMVTASN
EcoLPLAT2_EFX08826     RFCDFMMYCW CEGLAVLLHL NPHLQWEVHG L---EGLSKK NWYLLICNHR SWADIVVLCV
EcoLPLAT3_EFX09355     RLARRSIAAS FRLFLTVAKG LGVLDYRIDG A---EILHQE RGCLVVANHP TLIDYVLLAS
EdLPLAT1_XP_001739767  WIFQRVTEVY MMYFPLIFYY IIGNRIIETG D----ELVPN ENALFLCNHT HFYDFLPIVI
EdLPLAT2_XP_001734633  WIFQRVTEVY MMYFPLVFYY INGNRIYVSG D----KMIEN ANAIWISNHS HWVDFVPVCL
EhLPLAT1_XP_655766     WIFQRVTEVY MMYFPLIFYY IIGNRIIETG D----ELIPN ENALFLCNHT HFYDFLPIVI
EhLPLAT2_XP_649422     WIFQRVTEVY MMYFPLVFYY INGNRIYISG D----KFIEN VNAIWISNHS HWVDFIPVCL
HsLPLAT1_NP_006402     NV---ENMKI LRLMLLHIKY LYGIRVEVRG A---HHFPPS QPYVVVSNHQ SSLDLLGMME
HsLPLAT2_NP_006403     TV---ENMSI IGWFVRSFKY FYGLRFEVRD P---RRLQEA RPCVIVSNHQ SILDMMGLME
HsLPLAT3_NP_064517     RLNCRLAYSL WSQLVMLLEW WSCTECTLFT DQATVERFGK EHAVIILNHN FEIDFLCGWT
HsLPLAT4_NP_064518     KINCRLSYCI SSQLVMLLEW WSGTECTIFT DPRAYLKYGK ENAIVVLNHK FEIDFLCGWS
HsLPLAT5_NP_060831     ALDDRLYCVY QSMVLFFFEN YTGVQILLYG D----LPKNK ENIIYLANHQ STVDWIVADI
HsLPLAT6_NP_001002257  WINNRLVATW LTLPVALLET MFGVKVIITG D----AFVPG ERSVIIMNHR TRMDWMFLWN
HsLPLAT7_NP_055688     WYIEGIMYKW LLGMVASWGW YAGYTVMEWG ED--IKAVSK DEAVMLVNHQ ATGDVCTLMM
HsLPLAT8_NP_079106     KV---VDFLL KAIMRTMWFA GGFHRVAVKG R---QALPTE AAILTLAPHS SYFDAIPVTM
HsLPLAT9_NP_060309     KI---TQTAL KFLGRAMFFS MGFI-VAVKG K---IASPLE APVFVAAPHS TFFDGIACVV
HsLPLAT10_NP_705841    TV---CHNGV LGLSRLLFFL LGFLRIRVRG Q---RASRLQ APVLVAAPHS TFFDPIVLLP
```

AGPAT motif: (1) HssyfD

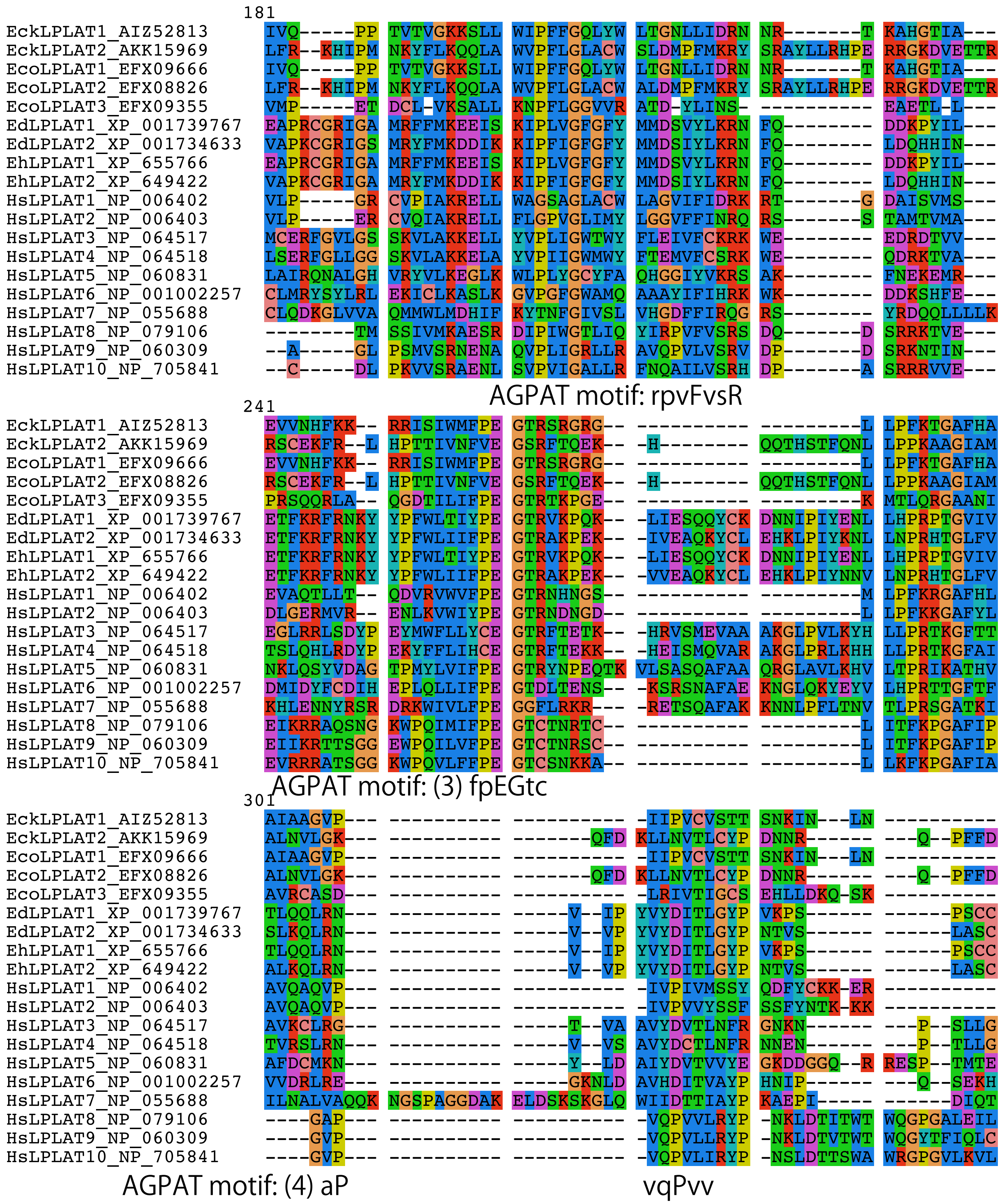
181
EckLPLAT1_AIZ52813 IVQ-----PP TVTVGKKSLL WIPFFGQLYW LTGNLLIDRN NR-------T KAHGTIA---
EckLPLAT2_AKK15969 LFR--KHIPM NKYFLKQQLA WVPFLGLACW SLDMPFMKRY SRAYLLRHPE RRGKDVETTR
EcoLPLAT1_EFX09666 IVQ-----PP TVTVGKKSLL WIPFFGQLYW LTGNLLIDRN NR-------T KAHGTIA---
EcoLPLAT2_EFX08826 LFR--KHIPM NKYFLKQQLA WVPFLGLACW ALDMPFMKRY SRAYLLRHPE RRGKDVETTR
EcoLPLAT3_EFX09355 VMP-----ET DCL-VKSALL KNPFLGGVVR ATD-YLINS- ---------- EAETL-L---
EdLPLAT1_XP_001739767 EAPRCGRIGA MRFFMKEEIS KIPLVGFGFY MMDSVYLKRN FQ-------- DDKPYIL---
EdLPLAT2_XP_001734633 VAPKCGRIGS MRYFMKDDIK KIPFIGFGFY MMDSIYLKRN FQ-------- LDQHHIN---
EhLPLAT1_XP_655766 EAPRCGRIGA MRFFMKEEIS KIPLVGFGFY MMDSVYLKRN FQ-------- DDKPYIL---
EhLPLAT2_XP_649422 VAPKCGRIGA MRYFMKDDIK KIPFIGFGFY MMDSIYLKRN FQ-------- LDQHHIN---
HsLPLAT1_NP_006402 VLP-----GR CVPIAKRELL WAGSAGLACW LAGVIFIDRK RT-------G DAISVMS---
HsLPLAT2_NP_006403 VLP-----ER CVQIAKRELL FLGPVGLIMY LGGVFFINRQ RS-------S TAMTVMA---
HsLPLAT3_NP_064517 MCERFGVLGS SKVLAKKELL YVPLIGWTWY FLEIVFCKRK WE-------- EDRDTVV---
HsLPLAT4_NP_064518 LSERFGLLGG SKVLAKKELA YVPIIGWMWY FTEMVFCSRK WE-------- QDRKTVA---
HsLPLAT5_NP_060831 LAIRQNALGH VRYVLKEGLK WLPLYGCYFA QHGGIYVKRS AK-------- FNEKEMR---
HsLPLAT6_NP_001002257 CLMRYSYLRL EKICLKASLK GVPGFGWAMQ AAAYIFIHRK WK-------- DDKSHFE---
HsLPLAT7_NP_055688 CLQDKGLVVA QMMWLMDHIF KYTNFGIVSL VHGDFFIRQG RS-------- YRDQQLLLLK
HsLPLAT8_NP_079106 --------TM SSIVMKAESR DIPIWGTLIQ YIRPVFVSRS DQ-------D SRRKTVE---
HsLPLAT9_NP_060309 --A-----GL PSMVSRNENA QVPLIGRLLR AVQPVLVSRV DP-------D SRKNTIN---
HsLPLAT10_NP_705841 --C-----DL PKVVSRAENL SVPVIGALLR FNQAILVSRH DP-------A SRRRVVE---
AGPAT motif: rpvFvsR
241
EckLPLAT1_AIZ52813 EVVNHFKK-- RRISIWMFPE GTRSRGRG-- ---------- ---------L LPFKTGAFHA
EckLPLAT2_AKK15969 RSCEKFR--L HPTTIVNFVE GSRFTQEK-- -H-------- QQTHSTFQNL LPPKAAGIAM
EcoLPLAT1_EFX09666 EVVNHFKK-- RRISIWMFPE GTRSRGRG-- ---------- ---------L LPFKTGAFHA
EcoLPLAT2_EFX08826 RSCEKFR--L HPTTIVNFVE GSRFTQEK-- -H-------- QQTHSTFQNL LPPKAAGIAM
EcoLPLAT3_EFX09355 PRSQQRLA-- QGDTILIFPE GTRTKPGE-- ---------- ---------K MTLQRGAANI
EdLPLAT1_XP_001739767 ETFKRFRNKY YPFWLTIYPE GTRVKPQK-- -LIESQQYCK DNNIPIYENL LHPRPTGVIV
EdLPLAT2_XP_001734633 ETFKRFRNKY YPFWLIIFPE GTRAKPEK-- -IVEAQKYCL EHKLPIYKNL LNPRHTGLFV
EhLPLAT1_XP_655766 ETFKRFRNKY YPFWLTIYPE GTRVKPQK-- -LIESQQYCK DNNIPIYENL LHPRPTGVIV
EhLPLAT2_XP_649422 ETFKRFRNKY YPFWLIIFPE GTRAKPEK-- -VVEAQKYCL EHKLPIYNNV LNPRHTGLFV
HsLPLAT1_NP_006402 EVAQTLLT-- QDVRVWVFPE GTRNHNGS-- ---------- ---------M LPFKRGAFHL
HsLPLAT2_NP_006403 DLGERMVR-- ENLKVWIYPE GTRNDNGD-- ---------- ---------L LPFKKGAFYL
HsLPLAT3_NP_064517 EGLRRLSDYP EYMWFLLYCE GTRFTETK-- -HRVSMEVAA AKGLPVLKYH LLPRTKGFTT
HsLPLAT4_NP_064518 TSLQHLRDYP EKYFFLIHCE GTRFTEKK-- -HEISMQVAR AKGLPRLKHH LLPRTKGFAI
HsLPLAT5_NP_060831 NKLQSYVDAG TPMYLVIFPE GTRYNPEQTK VLSASQAFAA QRGLAVLKHV LTPRIKATHV
HsLPLAT6_NP_001002257 DMIDYFCDIH EPLQLLIFPE GTDLTENS-- -KSRSNAFAE KNGLQKYEYV LHPRTTGFTF
HsLPLAT7_NP_055688 KHLENNYRSR DRKWIVLFPE GGFLRKR--- -RETSQAFAK KNNLPFLTNV TLPRSGATKI
HsLPLAT8_NP_079106 EIKRRAQSNG KWPQIMIFPE GTCTNRTC-- ---------- ---------L ITFKPGAFIP
HsLPLAT9_NP_060309 EIIKRTTSGG EWPQILVFPE GTCTNRSC-- ---------- ---------L ITFKPGAFIP
HsLPLAT10_NP_705841 EVRRRATSGG KWPQVLFFPE GTCSNKKA-- ---------- ---------L LKFKPGAFIA
AGPAT motif: (3) fpEGtc
301
EckLPLAT1_AIZ52813 AIAAGVP--- ---------- ---------- -IIPVCVSTT SNKIN---LN ----------
EckLPLAT2_AKK15969 ALNVLGK--- ---------- -------QFD KLLNVTLCYP DNNR------ ---Q--PFFD
EcoLPLAT1_EFX09666 AIAAGVP--- ---------- ---------- -IIPVCVSTT SNKIN---LN ----------
EcoLPLAT2_EFX08826 ALNVLGK--- ---------- -------QFD KLLNVTLCYP DNNR------ ---Q--PFFD
EcoLPLAT3_EFX09355 AVRCASD--- ---------- ---------- -LRIVTIGCS EHLLDKQ-SK ----------
EdLPLAT1_XP_001739767 TLQQLRN--- ---------- -----V--IP YVYDITLGYP VKPS------ ------PSCC
EdLPLAT2_XP_001734633 SLKQLRN--- ---------- -----V--VP YVYDITLGYP NTVS------ ------LASC
EhLPLAT1_XP_655766 TLQQLRN--- ---------- -----V--IP YVYDITLGYP VKPS------ ------PSCC
EhLPLAT2_XP_649422 ALKQLRN--- ---------- -----V--VP YVYDITLGYP NTVS------ ------LASC
HsLPLAT1_NP_006402 AVQAQVP--- ---------- ---------- -IVPIVMSSY QDFYCKK-ER ----------
HsLPLAT2_NP_006403 AVQAQVP--- ---------- ---------- -IVPVVYSSF SSFYNTK-KK ----------
HsLPLAT3_NP_064517 AVKCLRG--- ---------- -----T--VA AVYDVTLNFR GNKN------ ---P--SLLG
HsLPLAT4_NP_064518 TVRSLRN--- ---------- -----V--VS AVYDCTLNFR NNEN------ ---P--TLLG
HsLPLAT5_NP_060831 AFDCMKN--- ---------- -----Y--LD AIYDVTVVYE GKDDGGQ--R RESP--TMTE
HsLPLAT6_NP_001002257 VVDRLRE--- ---------- -----GKNLD AVHDITVAYP HNIP------ ---Q--SEKH
HsLPLAT7_NP_055688 ILNALVAQQK NGSPAGGDAK ELDSKSKGLQ WIIDTTIAYP KAEPI----- ------DIQT
HsLPLAT8_NP_079106 ----GAP--- ---------- ---------- -VQPVVLRYP -NKLDTITWT WQGPGALEIL
HsLPLAT9_NP_060309 ----GVP--- ---------- ---------- -VQPVLLRYP -NKLDTVTWT WQGYTFIQLC
HsLPLAT10_NP_705841 ----GVP--- ---------- ---------- -VQPVLIRYP -NSLDTTSWA WRGPGVLKVL
AGPAT motif: (4) aP
vqPvv

```
                       361
EckLPLAT1_AIZ52813     RLHNG----- -LVIVEMLPP IDVSQ--Y-- GKDQVREL-A AHCRSIMEQK IAELDK----
EckLPLAT2_AKK15969     -MLSGK-LTR IVVHVDLQPI ADELHGDYIN DKSFK----- RHFQ------ ----------
EcoLPLAT1_EFX09666     RLHNG----- -LVIVEMLPP IDVSQ--Y-- GKDQVREL-A AHCRSIMEQK IAELDK----
EcoLPLAT2_EFX08826     -MLSGK-LTR IVVHVDLQPI ADELHGDYIN DKSFK----- RHFQ------ ----------
EcoLPLAT3_EFX09355     WYDVPPDRP- -FFTVAVHGR VEINK--FYD ATSQEPAL-A ARQL--NRHL LLQLQQ----
EdLPLAT1_XP_001739767  -FFPGE-GIT IHMNIHKISI KDIPE----- ---DE----- ESLK------ ----------
EdLPLAT2_XP_001734633  -FCPGE-GVN IHMNVNRIDV KDIPE----- ---DE----- NEFK------ ----------
EhLPLAT1_XP_655766     -FFPGE-GIT IHMNIHKINI KDIPE----- ---DE----- ESLK------ ----------
EhLPLAT2_XP_649422     -FCPGE-GVN IHMYVNRIDV KEIPE----- ---DE----- TEFK------ ----------
HsLPLAT1_NP_006402     RFTSG----- -QCQVRVLPP VPTEG--L-- TPDDVPAL-A DRVRHSMLTV FREIST----
HsLPLAT2_NP_006403     FFTSG----- -TVTVQVLEA IPTSG--L-- TAADVPAL-V DTCHRAMRTT FLHISK----
HsLPLAT3_NP_064517     -ILYGK-KYE ADMCVRRFPL EDIPL----- ---DE----- KEAA------ ----------
HsLPLAT4_NP_064518     -VLNGK-KYH ADLYVRRIPL EDIPE----- ---DD----- DECS------ ----------
HsLPLAT5_NP_060831     -FLCKE-CPK IHIHIDRIDK KDVPE----- ---EQ----- EHMR------ ----------
HsLPLAT6_NP_001002257  -LLQGDFPRE IHFHVHRYPI DTLPT----- ---SK----- EDLQ------ ----------
HsLPLAT7_NP_055688     WILGYRKPTV THVHYRIFPI KDVPL----- ---ET----- DDLT------ ----------
HsLPLAT8_NP_079106     WLTLCQFHN- -QVEIEFLPV YSPSE----- EEKRNPALYA SNVRRVMAEA LGVSVTDYTF
HsLPLAT9_NP_060309     MLTFCQLFT- -KVEVEFMPV QVPND----- EEKNDPVLFA NKVRNLMAEA LGIPVTDHTY
HsLPLAT10_NP_705841    WLTASQPCS- -IVDVEFLPV YHPSP----- EESRDPTLYA NNVQRVMAQA LGIPATECEF

                       421
EckLPLAT1_AIZ52813     ------E--- ---------- ----VAEREA AGK------- ---------- ----V-----
EckLPLAT2_AKK15969     ---------- ---------- -------QWL NSLWQEKDRL LTSLMSSQRQ NK--------
EcoLPLAT1_EFX09666     ------E--- ---------- ----VAEREA AGK------- ---------- ----V-----
EcoLPLAT2_EFX08826     ---------- ---------- -------QWL NSLWQEKDRL LTSLMSSQRQ DK--------
EcoLPLAT3_EFX09355     -GCLPLS--- ---------- ----GINNAS AL-------- ---------- SGN-------
EdLPLAT1_XP_001739767  ---------- ---------- -------RWL DDLWVEKDKL MSYFKEH--- ---KEFPGEP
EdLPLAT2_XP_001734633  ---------- ---------- -------QWL SNIWKHKDEL VDYYKEN--- ---GHFPGHE
EhLPLAT1_XP_655766     ---------- ---------- -------RWL DDLWVEKDKL MSYFKEH--- ---KEFPGEP
EhLPLAT2_XP_649422     ---------- ---------- -------QWL CTIWKHKDEL VGYYKEN--- ---GHFPGIE
HsLPLAT1_NP_006402     ------D--- ---------- ----GRGGGD YLK------- --------KP GGGG------
HsLPLAT2_NP_006403     ------T--- ---------- ----PQENGA T--------- ---------- AGSGVQPAQ-
HsLPLAT3_NP_064517     ---------- ---------- -------QWL HKLYQEKDAL QEIYNQKG-- ----MFPGEQ
HsLPLAT4_NP_064518     ---------- ---------- -------AWL HKLYQEKDAF QEEYYRTG-- ----TFPETP
HsLPLAT5_NP_060831     ---------- ---------- -------RWL HERFEIKDKM LIEFYESPDP ERRKRFPGKS
HsLPLAT6_NP_001002257  ---------- ---------- -------LWC HKRWEEKEER LRSFYQGE-- -KNFYFTGQS
HsLPLAT7_NP_055688     ---------- ---------- -------TWL YQRFVEKEDL LSHFYETG-- ----AFPPSK
HsLPLAT8_NP_079106     EDCQLALAEG QLRLPADTCL LEFARLVRGL GLKPEKLEKD LDRYSERARM KGGEKIGIAE
HsLPLAT9_NP_060309     EDCRLMISAG QLTLPMEAGL VEFTKISRKL KLDWDGVRKH LDEYASIASS SKGGRIGIEE
HsLPLAT10_NP_705841    VGSLPVIVVG RLKVALEPQL WELGKVLRKA GLSAGYVDAG A-------EP GRSRMISQEE

                       481
EckLPLAT1_AIZ52813     ---------- ---------- ---------- ---------- ---------- ----------
EckLPLAT2_AKK15969     ---------- ---------- ---------- ---------- ---------- ----------
EcoLPLAT1_EFX09666     ---------- ---------- ---------- ---------- ---------- ----------
EcoLPLAT2_EFX08826     ---------- ---------- ---------- ---------- ---------- ----------
EcoLPLAT3_EFX09355     ---------- ---------- ---------- ---------- ---------- ----------
EdLPLAT1_XP_001739767  RKP-QFKFTW A--------- ---------D ---FTGYMT- --P------- ----------
EdLPLAT2_XP_001734633  ELY-PLKFTW A--------- ---------D ---FTSYMS- --Q------- ----------
EhLPLAT1_XP_655766     RKP-PFKFTW A--------- ---------D ---FTGYMT- --P------- ----------
EhLPLAT2_XP_649422     ELY-PLKFTW A--------- ---------D ---FTGYMS- --Q------- ----------
HsLPLAT1_NP_006402     ---------- ---------- ---------- ---------- ---------- ----------
HsLPLAT2_NP_006403     ---------- ---------- ---------- ---------- ---------- ----------
HsLPLAT3_NP_064517     FKP-ARRPW- -----TLLNF LSWATILLSP LFSFVLGVF- ---ASGSPLL ILTFLGFVGA
HsLPLAT4_NP_064518     MVP-PRRPW- -----TLVNW LFWASLVLYP FFQFLVSMI- ---RSGSSLT LASFILVFFV
HsLPLAT5_NP_060831     VNS-KLSIKK T-----LPSM LI-----LSG ---LTAGMLM --TDAGRKLY VN--------
HsLPLAT6_NP_001002257  VIP-PCKSEL RVLVVKLLSI LYWTLF--SP AMCLLIYLY- ---SLVKWYF IITIVIFV--
HsLPLAT7_NP_055688     GHKEAVSREM -----TLSN- -LWIFL-IQS FAFLSGYMW- --------YN IIQ---YFY-
HsLPLAT8_NP_079106     FAA-SLEVP- ------VSD- ---------- ---LLEDMFS LFDESGSGE- -VDLRECVVA
HsLPLAT9_NP_060309     FAK-YLKLP- ------VSD- ---------- ---VLRQLFA LFDRNHDGS- -IDFREYVIG
HsLPLAT10_NP_705841    FAR-QLQLS- ------DPQ- ---------- ---TVAGAFG YFQQDTKGL- -VDFRDVALA
```

```
                         541
EckLPLAT1_AIZ52813       ---------- ---------- ---------- ---------- ---------- ----------
EckLPLAT2_AKK15969       ---------- ---------- ---------- ---------- ---------- ----------
EcoLPLAT1_EFX09666       ---------- ---------- ---------- ---------- ---------- ----------
EcoLPLAT2_EFX08826       ---------- ---------- ---------- ---------- ---------- ----------
EcoLPLAT3_EFX09355       ---------- ---------- ---------- ---------- ---------- ----------
EdLPLAT1_XP_001739767    ---------- ---------- ---------- --------EC -----FTKP- ----------
EdLPLAT2_XP_001734633    ---------- ---------- ---------- --------EC -----FSLP- ----------
EhLPLAT1_XP_655766       ---------- ---------- ---------- --------EC -----FTKP- ----------
EhLPLAT2_XP_649422       ---------- ---------- ---------- --------EC -----FSLP- ----------
HsLPLAT1_NP_006402       ---------- ---------- ---------- ---------- ---------- ----------
HsLPLAT2_NP_006403       ---------- ---------- ---------- ---------- ---------- ----------
HsLPLAT3_NP_064517       ASFG------ ------VRRL IGVTEI---- -----EKGSS -----YGNQE FKKK-----E
HsLPLAT4_NP_064518       ASVG------ ------VRWM IGVTEI---- -----DKGSA -----YGNSD SKQK-----L
HsLPLAT5_NP_060831       ---------- -------TWI YGTLL----- --------GC -----LWVTI KA--------
HsLPLAT6_NP_001002257    ---L------ ------QERI FGGLEI---- -----IELAC -----YRLLH KQPH-----L
HsLPLAT7_NP_055688       ---------- ---------- ---------- --------HC -----LF--- ----------
HsLPLAT8_NP_079106       LSVVCRPART LDTIQLAFKM YGAQED---- GSVGEGDLSC ILKTALGVAE LTVTDLFRAI
HsLPLAT9_NP_060309       LAVLCNPSNT EEIIQVAFKL FDVDED---- GYITEEEFST ILQASLGVPD LDVSGLFKEI
HsLPLAT10_NP_705841      LAALDGGRSL EELTRLAFEL FAEEQAEGPN RLLYKDGFST ILHLLLGSPH PAATALHAEL

                         601
EckLPLAT1_AIZ52813       ---------- ---------- ---------- ---------- ---------- ----------
EckLPLAT2_AKK15969       ---------- ---------- ---------- ---------- ---------- ----------
EcoLPLAT1_EFX09666       ---------- ---------- ---------- ---------- ---------- ----------
EcoLPLAT2_EFX08826       ---------- ---------- ---------- ---------- ---------- ----------
EcoLPLAT3_EFX09355       ---------- ---------- ---------- ---------- ---------- ----------
EdLPLAT1_XP_001739767    ---------- ---------- ---------- ---------- ---------- ----------
EdLPLAT2_XP_001734633    ---------- ---------- ---------- ---------- ---------- ----------
EhLPLAT1_XP_655766       ---------- ---------- ---------- ---------- ---------- ----------
EhLPLAT2_XP_649422       ---------- ---------- ---------- ---------- ---------- ----------
HsLPLAT1_NP_006402       ---------- ---------- ---------- ---------- ---------- ----------
HsLPLAT2_NP_006403       ---------- ---------- ---------- ---------- ---------- ----------
HsLPLAT3_NP_064517       ---------- ---------- ---------- ---------- ---------- ----------
HsLPLAT4_NP_064518       N---D----- ---------- ---------- ---------- ---------- ----------
HsLPLAT5_NP_060831       ---------- ---------- ---------- ---------- ---------- ----------
HsLPLAT6_NP_001002257    N---SKKNE- ---------- ---------- ---------- ---------- ----------
HsLPLAT7_NP_055688       ---------- ---------- ---------- ---------- ---------- ----------
HsLPLAT8_NP_079106       DQE-EKGKIT FADFHRFAEM YPAFAEEYLY PDQTHFESCA ETSP----AP IPNGFCADFS
HsLPLAT9_NP_060309       AQG-D--SIS YEEFKSFALK HPEYAKIFT- -TYLDLQTCH VFSLPKEVQT TPSTASNKVS
HsLPLAT10_NP_705841      CQAGSSQGLS LCQFQNFSLH DPLYGKLFS- -TYLRPPHTS RG-----TSQ TPNASSPG-N

                         661
EckLPLAT1_AIZ52813       ---------- -------
EckLPLAT2_AKK15969       ---------- -------
EcoLPLAT1_EFX09666       ---------- -------
EcoLPLAT2_EFX08826       ---------- -------
EcoLPLAT3_EFX09355       ---------- -------
EdLPLAT1_XP_001739767    ---------- -------
EdLPLAT2_XP_001734633    ---------- -------
EhLPLAT1_XP_655766       ---------- -------
EhLPLAT2_XP_649422       ---------- -------
HsLPLAT1_NP_006402       ---------- -------
HsLPLAT2_NP_006403       ---------- -------
HsLPLAT3_NP_064517       ---------- -------
HsLPLAT4_NP_064518       ---------- -------
HsLPLAT5_NP_060831       ---------- -------
HsLPLAT6_NP_001002257    ---------- -------
HsLPLAT7_NP_055688       ---------- -------
HsLPLAT8_NP_079106       PENSDAGRK- PVRKKLD
HsLPLAT9_NP_060309       PEKHEESTS- DKKD--D
HsLPLAT10_NP_705841      PTALANGTVQ APKQKGD
```

Supplement Figure 3A. Sequence alignment utilized in Figure 4A.
AGPAT motifs in HsLPLAT8 were shown.

Supplementary Fig. 3B

```
                            1
HsLPLAT11_NP_077274         ---------- ---------- ---------- --------MS PEEWTY-LVV LLISIPIGF-
HsLPLAT12_NP_005759         -MASSAEGDE GTVVALAGVL QSGFQELSLN KLATSL-GAS EQALRL-IIS IFLGYPFALF
HsLPLAT13_NP_620154         ---------- --MATTSTT- ----GSTLLQ PLSNAV-QLP IDQVNF-VVC QLFALLAAIW
HsLPLAT14_NP_001073949      -MAAEP--QP S-SLSYRTT- ----GSTYLH PLSELL-GIP LDQVNF-VVC QLVALFAAFW
EdLPLAT3_XP_001734903       MKEKIS--DS R-SYKFKRM- ----LDSFVT TSAQLI-GFP EDMFRF-LFA FILEVPISI-
EdLPLAT4_XP_001733896       ---------- ------MSV- ----IDFILE PISQIV-HVP VYLLKY-FSS FVLSLPMTY-
EdLPLAT5_XP_001735834       ---------- --MFSNYSV- ----VDEWVQ PISKGL-NVP IDQVKY-FTN AIIAAPICF-
EdLPLAT6_XP_001736261       ---------- ---------- ----MFTVIE SLAEKV-HVP ADQIRY-LLA FFIELPLCY-
EhLPLAT3_XP_653096          ---------- --------M- ----LDSFVT TSAQLI-GFP EDVFRF-LFA FIVEVPISI-
EhLPLAT4_XP_652418          ---------- ------MSV- ----IDFILE PISQIV-HVP VYLLKY-FSS FVLSLPMTY-
EhLPLAT5_XP_651748          ---------- --MFSNYSL- ----ADDWVE PLSSAL-NVP LDQVKY-FSN AVLAAPICF-
EhLPLAT6_XP_655691          ---------- ---------- ----MYSIIE SFSEKV-YVP SDQMRY-FLS FFIELPLCY-
EhLPLAT7_XP_648994          ---------- ---------- --------ME TIQKLCHSIP VISPHYYTVA LLFLAPIPF-

                           61
HsLPLAT11_NP_077274         -----LFKKA GPGLKRWGAA AVGLGLTLFT CGPHTLHSLV TILGTWALIQ AQPCSCHA--
HsLPLAT12_NP_005759         YRHYLFYKE- -TYLIHLFHT FTGLSIAYFN FGNQLYHSLL CIVLQFLILR LMGRTITAVL
HsLPLAT13_NP_620154         FRTYLHSSKT SSFIRHVVAT LLGLYLALFC FGWYALHFLV QSGISYCIMI IIGVENMH-N
HsLPLAT14_NP_001073949      FRIYLRPGTT SSDVRHAVAT IFGIYFVIFC FGWYSVHLFV LVLMCYAIMV TASVSNIH-R
EdLPLAT3_XP_001734903       ---ICRYISM STDMKYFIYG IIGIFISFFV YSTMTYCVLI TMIPVYIIMK YMPTRKGA-Y
EdLPLAT4_XP_001733896       ---YCRYLPN NPSLKHKVYG SIGITMSLIV FGPITFSIIF LGLPIYYIAK TYPTRIMA-I
EdLPLAT5_XP_001735834       ---GLRYLPN NKNIKHIVYG TLGMIFSYLL FGQESITVIF TTIPVYLIMK YNKTQKAA-N
EdLPLAT6_XP_001736261       ---ILRYLPS NPRLKHFVYS TVGVIMLLFV FGINSLIIMI PALITYYFMK WKKSLHMA-L
EhLPLAT3_XP_653096          ---ICRYIPL STDMKYLIYG IIGIFISFFV YSTMTYCVLI TMIPVYLIMK YVPTRKGA-Y
EhLPLAT4_XP_652418          ---YCRYLPN NPSLKHKVYG SIGILMSLIV FGPITFSIIF LGLPIYYIAK TYPTRIMA-T
EhLPLAT5_XP_651748          ---GLRYLPN NQNIKHIVYG TLGMVFSYLL FGMEFITVFF TSIPVYLIMK YNKTQKAA-N
EhLPLAT6_XP_655691          ---ILRYLPS NPRLKHFVYS IIGVIILLFV FGINSMIIII PGLITYYAMK WKKSLHAA-F
EhLPLAT7_XP_648994          -GLISKFIKN -RTARVWYII IISFICQWVC FQEYMLHGTF AILMTYIFMK IIPFPQAH-Y

                          121
HsLPLAT11_NP_077274         LALAWTFSYL LFFRALS--- LLGLPTPTPF TNAVQLLLTL KLVSLASEVQ DLHLAQRKEM
HsLPLAT12_NP_005759         TTFCFQMAYL LAGY----YY TATGNYDIKW -TMPHCVLTL KLIGLAVDYF DGGKDQ-NSL
HsLPLAT13_NP_620154         YCFVFALGYL TVCQVTRVYI FDYGQYSADF -SGPMMIITQ KITSLACEIH DGMFRKDEEL
HsLPLAT14_NP_001073949      YSFFVAMGYL TICHISRIYI FHYGILTTDF -SGPLMIVTQ KITTLAFQVH DGLGRRAEDL
EdLPLAT3_XP_001734903       LCFGISLGYL LTIHIKR-MI DNYLGYDLDF -SSVQMVLTI KFTTFAFSVC NAYDKEY-KC
EdLPLAT4_XP_001733896       ITTTMLILHL ACIHLYK-IL HTGMVYNLSF -STVHMMYVI KFSTFVWSVV DAHENNT-KL
EdLPLAT5_XP_001735834       ICFIITFGYL LYRHIYL-YF NKYLIWTVEF -TTTHMILTL KLTGFAFSVA NSNKKE---L
EdLPLAT6_XP_001736261       LMFSMNLIFL TLLQIYK-YI NYYLQWYLDI -TTLQMIVVI KLGNFVFNVA KGSNTNIIRQ
EhLPLAT3_XP_653096          ACFGISLGYL LTIHIKR-MI DNYLGYDLDF -SSVQMVLTI KFTTFAFSVC NAQDKEY-KC
EhLPLAT4_XP_652418          ITTIVLILHL ACIHLYK-IL HTGVVYNLSF -STVHMMYVI KFSTFVWSVV DAHENNI-KL
EhLPLAT5_XP_651748          ICFIITFGYL LYRHIYL-YF NMYLVWTLEF -TTTHMIITL KLTAFAFSVA NSFKKE---L
EhLPLAT6_XP_655691          FIFSLNLMFL TLLQIHK-YI NYYLQWYLDI -TTLQMIVVI KLGNFAFNVA KGSNTNTIKQ
EhLPLAT7_XP_648994          INLIFQFAYV LYGHAWR-MY NYYLVYSTDW -TIIQMLLVV RLTQISWYKN LSYQPI-ESI

                          181
HsLPLAT11_NP_077274         ASGFSKGPTL GLLPDVPSLM ETLSYSYCYV GIMTGPFFRY RTYLDWLEQP FPGAVP----
HsLPLAT12_NP_005759         SSEQ----QK YAIRGVPSLL EVAGFSYFYG AFLVGPQFSM NHYMKLVQGE LIDIPG----
HsLPLAT13_NP_620154         TSSQ----RD LAVRRMPSLL EYLSYNCNFM GILAGPLCSY KDYITFIEGR SYHITQSGEN
HsLPLAT14_NP_001073949      SAEQ----HR LAIKVKPSFL EYLSYLLNFM SVIAGPCNNF KDYIAFIEGK HIHMKLLEVN
EdLPLAT3_XP_001734903       SEHT----EK HKIYNYPSIL QFFGYTFFFP AFFSGPSMEY TDFVSFIDMS MFKEFN----
EdLPLAT4_XP_001733896       CEHR----KQ MMIKTYPSLL EFFGYTFFFP GIFSGPSLEY IDYIKFITLE QFKDYP----
EdLPLAT5_XP_001735834       TKYL----EI HKINKYPNII EYYGFIFFYP GLFSGPTLEY NDYMSFIDMS RFKEIK----
EdLPLAT6_XP_001736261       TDYN--KMNN IPRDQFPSLL EFIGYFYFFP SIFSGPCIEY QTYKKFVTLE LFESYP----
EhLPLAT3_XP_653096          SEHT----EK HNIYNYPSIL QFFGYTFFFP AFFSGPSMEY TDFVSFIDMS MFKEFN----
EhLPLAT4_XP_652418          CEHR----KQ MMIKTYPSLL EFFGYTFFFP GIFSGPSLEY IDYIKFITLE QFKNYP----
EhLPLAT5_XP_651748          TPYL----EA HKINKYPSII EFYGFIFFYP GLFSGPSLEY RDYMSFIDMS RFNLIK----
EhLPLAT6_XP_655691          TDYN--KMNN IPNDQFPSLL EFIGYFYFFP SIFSGPCIEY QTYKKFVTLE LFESYP----
EhLPLAT7_XP_648994          KPRH----RN YILKDDISLF DLFAYCYHLP GMLIGPNVDY PTFLKFLDLS MFPNKQI---
```

```
                        241
HsLPLAT11_NP_077274     --------SL RPLLRRAWPA PLFGLL---- -FLLSSHLFP LEA-----VR EDAFYARPLP
HsLPLAT12_NP_005759     --------KI PNSIIPA-LK RLSLGLF-YL V--GYTLLSP HITEDYL-LT ED-YDNHPFW
HsLPLAT13_NP_620154     GKEETQYERT EPSPNTAVVQ KLLVCGLSLL FHLTICTTLP VE---YN-ID EHFQATASWP
HsLPLAT14_NP_001073949  WKRKGFHSLP EPSPTGAVIH KLGITLVSLL LFLTLTKTFP VT---CL-VD DWFVHKASFP
EdLPLAT3_XP_001734903   -------NKL PPINMKAVGK VTLRLIVIIV FYLISVKYSI FELCEYY-IL GH-KEESTCW
EdLPLAT4_XP_001733896   -KD-KMEGDV PKIPMNLFYK RIGLGIAFYM LV-WAISIFE PLLADYY-FL EA-PELHGFF
EdLPLAT5_XP_001735834   -G-----NQL PKIPILKFIE HYFGGIILYF IY-AYINSNP LPDPEYY-IL KH-PETCNIG
EdLPLAT6_XP_001736261   -NN-HLKNKI PPIDWKQFFI VFGQGIILLG LT-SIVIMIP LKAYFYEIVI NN-PNDYSFI
EhLPLAT3_XP_653096      -------NKL PPINMKAVGT VTLRLILIIV FHLVSIKCSI FEFCEYY-IL GH-KEESTCW
EhLPLAT4_XP_652418      -KD-KMEGDV PQIPMKMFYK RIGLGITFYM LV-WAISIFE PLLADYY-FL EA-PESHGFF
EhLPLAT5_XP_651748      -G-----KEL PKIPILKFLE HYIGGIIIYF IY-AYINSNP LPNPEYY-IL EH-PETCSIW
EhLPLAT6_XP_655691      -NN-HLKNKI PPIDWKQFFI VFSQGIILLL LT-SIVIIVP LKVYFYEIVI NN-PNDYSFI
EhLPLAT7_XP_648994      ---------P KTVNTKAFYT RLIELFIIYM FGV----TDP L-DFKYM-IT PE-FMEHSFF

                        301
HsLPLAT11_NP_077274     ARLFYMIPVF FAFRMRFYVA WIAAECGCIA AGFGAYPVAA KARAGGGPTL QCPPPSSPEK
HsLPLAT12_NP_005759     FRCMYMLIWG KFVLYKYVTC WLVTEGVCIL TGLGFNGFEE KG-------- ----------
HsLPLAT13_NP_620154     TKIIYLYISL LAARPKYYFA WTLADAINNA AGFGFRGYDE NG-------- ----------
HsLPLAT14_NP_001073949  ARLCYLYVVM QASKPKYYFA WTLADAVNNA AGFGFSGVDK NG-------- ----------
EdLPLAT3_XP_001734903   FKIFMIYLYV EFIKLRYYFV WEFGELLSIL MGFGYSG-MK DG-------- ----------
EdLPLAT4_XP_001733896   FKMFAIWYTG ELALAKYIGT WYVADAMSVI GGFGFTT-IK NG-------- ----------
EdLPLAT5_XP_001735834   WKLLTIWWTV STLRLKYYGT WKLTESLGEI SGCGYTGTTK EG-------- ----------
EdLPLAT6_XP_001736261   QRMGMVMIFI YSIVFRYFAT WKMAECMGIL FGFGYSG-IQ EN-------- ----------
EhLPLAT3_XP_653096      FKLLMILLYV EFIKLRYYFV WEFGELLSIL MGFGYSG-MK DG-------- ----------
EhLPLAT4_XP_652418      FKMFAIWYTG EMALTKYIGT WYVADAMSII GGFGFTT-IK NG-------- ----------
EhLPLAT5_XP_651748      WKLLTIWWTV STIRLKYYGT WKLTESLGEI SGCGYTGTTK EG-------- ----------
EhLPLAT6_XP_655691      QKMGMVMIFI YSIVFRYFAT WKMAECMGIL FGFGYSG-VK EN-------- ----------
EhLPLAT7_XP_648994      YRWGYLMVAH YTNMSKYLFM FVAGELACVG CGISYYI-NE KG-------- ----------

                        361
HsLPLAT11_NP_077274     AASLEYDYET IRNIDCYSTD FCVRVRDGMR YWNMTVQWWL AQYIYKSAPA RS---YVLRS
HsLPLAT12_NP_005759     ----KAKWDA CANMKVWLFE TNPRFTGTIA SFNINTNAWV ARYIFKRLKF LGNK--ELSQ
HsLPLAT13_NP_620154     ----AARWDL ISNLRIQQIE MSTSFKMFLD NWNIQTALWL KRVCYERTS- ------FSPT
HsLPLAT14_NP_001073949  ----NFCWDL LSNLNIWKIE TATSFKMYLE NWNIQTATWL KCVCYQRVP- ------WYPT
EdLPLAT3_XP_001734903   ----ETTWEG FKNVDIWKME TADSIRQIVS NWNIQTERWL RYYVYERLN- QNKTLAPYKS
EdLPLAT4_XP_001733896   ----KEVWDK FTNIDFPVFY LSTSFRNSIV LWNRYVQNWL SNYVYLN--L EGTKLDAFKT
EdLPLAT5_XP_001735834   ----KDQFLL YRNVSILEFE ICRSCKVNMD LWNTYVQKWL KNYVYVS--M IGTPLEKYKT
EdLPLAT6_XP_001736261   ----KPMWYG FRNVKLNDFF FSNSAKLVID SWNIYIQTFM KNHIYESFEL FGGVLNKYKQ
EhLPLAT3_XP_653096      ----ETTWEG FKNVDIWKME TSDSVRQIVS NWNIQTERWL RYYVYERLN- QNKTLAPYKS
EhLPLAT4_XP_652418      ----KEIWDK FTNIDFPVFY LSTSFRNSIV LWNRYVQNWL SNYIYLN--L EGTKLDAFKT
EhLPLAT5_XP_651748      ----KDEFLL YRNVSIIEFE VCRSCKVNMD LWNTYVQKWL KNYVYVS--M IGTPLERYKT
EhLPLAT6_XP_655691      ----KPTWYG FRNVKLNDFF FSNSAKLVID SWNIYIQTFM KNHIYESFEL FGGILNEYKQ
EhLPLAT7_XP_648994      ----EEKFCK YRNIRMMKIL TARGLRMFAS NWNLGIVQWI TSVVNDIVP- -RSWPELARH
```

MBOAT motif: (A) WD

```
                        421
HsLPLAT11_NP_077274     AWTMLLSAYW HGLHPGYYLS FLTIPLCLAA E----G---- RLESALRGRL SPG--GQKAW
HsLPLAT12_NP_005759     GLSLLFLALW HGLHSGYLVC FQMEFLIVIV ERQAARLIQE S--PTLS-KL AAITVLQPFY
HsLPLAT13_NP_620154     IQTFILSAIW HGVYPGYYLT FLTGVLMTLA A----R---- AMRNNFRHYF IEPSQLKLFY
HsLPLAT14_NP_001073949  VLTFILSALW HGVYPGYYFT FLTGILVTLA A----R---- AVRNNYRHYF LSSRALKAVY
EdLPLAT3_XP_001734903   ILTNMVSAFW HGLYPGYYIS FGTMTLQQKF Q----YMLYT QVLPLLRNKY GEKSIPIYLY
EdLPLAT4_XP_001733896   VITNLVSAFW HGFLPGYYIS FGTLALHTEL S----KLIYK KLTPYIIYLT KNNKKMMTVW
EdLPLAT5_XP_001735834   TLTMVVCAFW HGVYPGYYIS FLILGFDKDL S----NLIYK RLDPFMKIKF GEKSIQWKLY
EdLPLAT6_XP_001736261   SLTNLISAFW HGIYPGYYLS FGMLALHHDV S----CGFHS RIEPIIIKKY GKDSLIYYSY
EhLPLAT3_XP_653096      LLTNMVSAFW HGLYPGYYIA FGTMTLQQKF Q----YMLYT QVLPLLRNKY GEKSIPIYLY
EhLPLAT4_XP_652418      VITNLVSAFW HGFLPGYYIS FGTLALHTEL S----KLIYK KLTPYIAYLT KNNNKMMTLW
EhLPLAT5_XP_651748      SLTMAVCAFW HGVYPGYYMS FFVLGFDKDL S----NLIYK RLDPYMRMKF GEGSIVWNGY
EhLPLAT6_XP_655691      SLTNLVSAFW HGIYPGYYLS FGMLALHHDV S----CGFHS RIEPIIIKKY GKDSLIYYSY
EhLPLAT7_XP_648994      ALTYIVNAFW HGFYPGYLLF FFCCGIPYEF C----S---K GFLKIINTYI NKKSKLYYVY
```

MBOAT motif: (B) W HGlhsGY lvc F

```
                        481
HsLPLAT11_NP_077274     DWVHWFLKMR AYDYMCMGFV LLSLADTLRY WASIYFCIHF LALAALGLGL ALGGGSPS--
HsLPLAT12_NP_005759     YLVQQTIHWL FMGYSMTAFC LFTWDKWLKV YKSIYFLGHI FFLSLLFILP YIHKAMVP--
HsLPLAT13_NP_620154     DVITWIVTQV AISYTVVPFV LLSIKPSLTF YSSWYYCLHI LGILVLLLLP VKKTQRRK--
HsLPLAT14_NP_001073949  DAGTWAVTQL AVSYTVAPFV MLAVEPTISL YKSMYFYLHI ISLLIILFLP MKPQAHTQ--
EdLPLAT3_XP_001734903   HTFNYLYTPF AQMYCFVSFV LLSFSATFTV YLHTYFIPHI IALTGWMYLS YFPVYKKP--
EdLPLAT4_XP_001733896   NVFLIIFTNL SIKYNFCPFF MMTFKASWEM FKQTYFCFHL IALILFIWLT VAPPKIPK--
EdLPLAT5_XP_001735834   DILLRVFNHW HLNYAVYPFM RFELIPSLIV LYRTYFLGYL IPLFLYLWLS YYPPHLSY--
EdLPLAT6_XP_001736261   WVLTFIYARI SISYSFIPFV IYTFIDSWKF FVNTYFCVDV IGLLALIILY VWFPKQQK--
EhLPLAT3_XP_653096      HTFNYIYTPF AQMYCFVSFV LLSFSATFTV YSYTYFIPHI IALSGWMYLS YFPVYKKP--
EhLPLAT4_XP_652418      NIFLIIFTNL SIKYNFCPFF MMTFKASWEM FKQTYFCFHL IALVLFIWLT VAPPKIPK--
EhLPLAT5_XP_651748      DILLRIFNHW HLNYAVYPFM RFELIPSLIV VYRTYFLGYL IPLILYLWLT YYPPHLTQ--
EhLPLAT6_XP_655691      LVLTFIYARI SISYSFIPFV IYTFSDSWKF FVNTYFCIDI IGLLLLIILY IWFPKQQK--
EhLPLAT7_XP_648994      QVLGFFFSLI LMQFCHMAFN LMAFEYVWKC WSNFHFYFYV ILAGMYVLQF IIPPLPADYW
```

MBOAT motifs: (C) YsmtaF (D) YksiYFlgH

```
                             541
HsLPLAT11_NP_077274          -RRKAAS--- --QPTSLAPE KLREE----- ---------- ----------
HsLPLAT12_NP_005759          --RKEKLKKM E--------- ---------- ---------- ----------
HsLPLAT13_NP_620154          -NTHENIQLS QSKKFDEGEN SLGQNSFSTT NNVCNQNQEI ASRHSSLKQ
HsLPLAT14_NP_001073949       -RRPQTLNSI NKRKTD---- ---------- ---------- ----------
EdLPLAT3_XP_001734903        --RQETVETK PKEVTTGSPA TKE------- ---------- ----------
EdLPLAT4_XP_001733896        -EEKNKKE-- ---------- ---------- ---------- ----------
EdLPLAT5_XP_001735834        -QNTMNEQLK QQ--KDSTLL KKED------ ---------- ----------
EdLPLAT6_XP_001736261        -RDRID---- ---------- ---------- ---------- ----------
EhLPLAT3_XP_653096           -SRQETVETK PKEVSTGSPA TKET-TKSE- ---------- ----------
EhLPLAT4_XP_652418           -EEKEKKNN- ---------- ---------- ---------- ----------
EhLPLAT5_XP_651748           -EKIKKEE-- ---------- ---------- ---------- ----------
EhLPLAT6_XP_655691           -RDRID---- ---------- ---------- ---------- ----------
EhLPLAT7_XP_648994           DDKKETKESK PFEPVNPQQQ ELEKPKK--- EEV------- KMD------
```

Supplement Figure 3B. Sequence alignment utilized in Figure 4B.
MBOAT motifs in HsLPLAT12 were shown.
